# Boryl substitution of functionalized aryl-, heteroaryl- and alkenyl halides with silylborane and an alkoxy base: expanded scope and mechanistic studies†
†Electronic supplementary information (ESI) available. See DOI: 10.1039/c5sc00384a



**DOI:** 10.1039/c5sc00384a

**Published:** 2015-03-02

**Authors:** Eiji Yamamoto, Satoshi Ukigai, Hajime Ito

**Affiliations:** a Division of Chemical Process Engineering and Frontier Chemistry Center , Faculty of Engineering , Hokkaido University , Sapporo 060-8628 , Japan . Email: hajito@eng.hokudai.ac.jp

## Abstract

A transition-metal-free method has been developed for the boryl substitution of functionalized aryl-, heteroaryl- and alkenyl halides with a silylborane in the presence of an alkali-metal alkoxide. The base-mediated boryl substitution of organohalides with a silylborane was recently reported to provide the corresponding borylated products in good to high yields, and exhibit good functional group compatibility and high tolerance to steric hindrance. In this study, the scope of this transformation has been extended significantly to include a wide variety of functionalized aryl-, heteroaryl- and alkenyl halides. In particular, the boryl substitution of (*E*)- and (*Z*)-alkenyl halides proceeded smoothly to afford the corresponding alkenyl boronates in good to high yields with retention of the configuration using modified reaction conditions. The results of the mechanistic studies suggest that this boryl substitution proceeds *via* a carbanion-mediated mechanism.

## Abstract 2

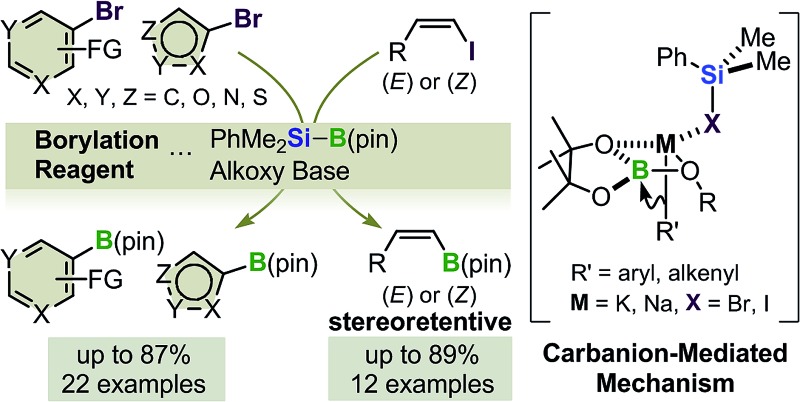
A transition-metal-free method has been developed for the boryl substitution of functionalized aryl-, heteroaryl- and alkenyl halides using a silylborane/alkoxy-base reagent. Borylation of (*Z*)-alkenyl halides proceeded in a stereoretentive manner.

## Introduction

**Fig. 1:**
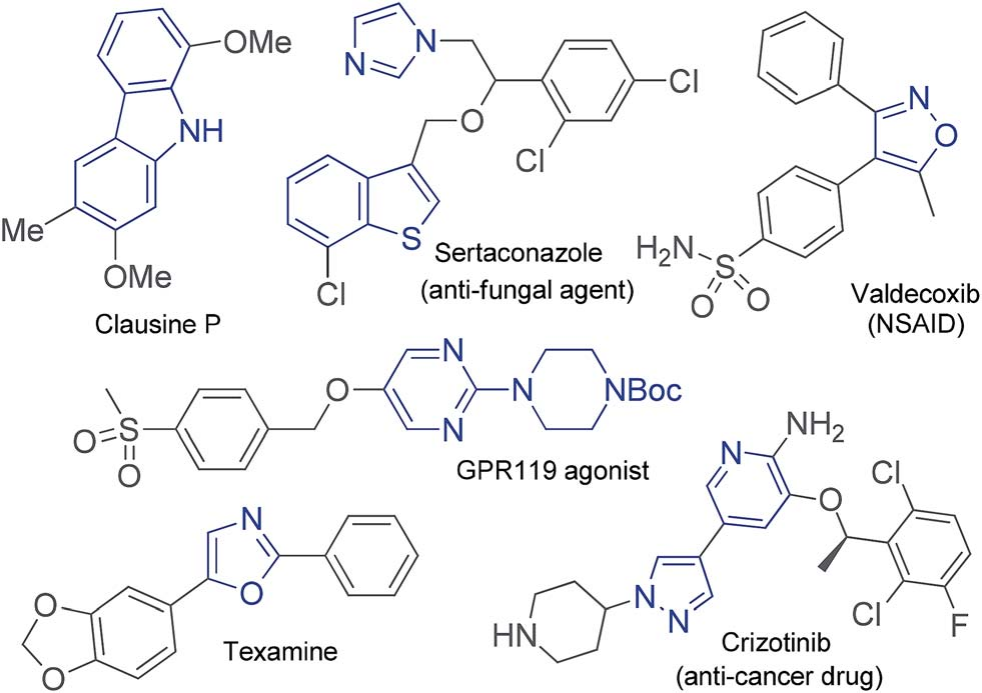
Natural products and pharmaceuticals containing five- and six-membered heterocyclic rings.

Functionalized aryl-, heteroaryl- and alkenyl boronate esters are incredibly useful and versatile building blocks in organic synthesis because they can be readily converted into a wide variety of different functional groups. Heteroaryl boronates are especially important for pharmaceutical applications because heteroaryl moieties such as oxazole, carbazole, pyridine, pyrazole and pyrimidine groups are often found in a large number of pharmaceuticals and natural products, as shown in Fig. 1.^1^

Considerable research effort has been devoted to the development of versatile methods for the preparation of organoboronate esters.^2^ Despite considerable progress in this area, the existing synthetic methods are still in need of further improvement. One of the most conventional procedures for the synthesis of organoboronate esters involves the reaction of organolithium or Grignard reagents with boron electrophiles.^3^ However, these methods are limited by the availability of the organolithium or Grignard reagents, which have low functional group compatibility and require additional preparation steps.

Several transition-metal-catalyzed methods have been developed during the course of the past two decades for the borylation of aryl and alkenyl halides,^4,5^ C(sp^2^)–H borylation^6^ and the hydroboration of alkynes,^7^ and all of these methods show a broad functional group compatibility. However, the application of these methods to the preparation of pharmaceuticals has been limited by the costs associated with the use of expensive transition-metal catalysts and the potential for contamination of the product with residual transition-metal impurities.^8,9^ Furthermore, many transition-metal-catalyzed borylation reactions are susceptible to steric constraints imposed by the substrate. For example, reaction of bulky 2,4,6-triisopropylbromobenzene requires exquisite catalysts.^4e–g^

Based on these limitations, the development of functional group and steric-bulk tolerant, transition-metal-free synthetic routes for the construction of organoboronates is strongly desired. Several transition-metal-free borylation methods have been developed, including radical-mediated and electrophilic borylation^10,11^ reactions. However, these reactions still have issues in terms of their reactivity, regioselectivity and functional group compatibility. Furthermore, to the best of our knowledge, there are currently no transition-metal-free boryl substitution reactions available for the synthesis of disubstituted (*Z*)-alkenyl boronates, which are important building blocks in organic chemistry.

**Fig. 2:**
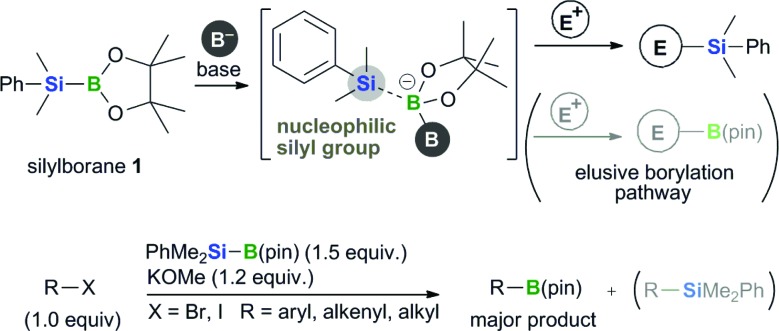
Boryl substitution of organohalides with silylborane and an alkoxy base.

We previously reported the boryl substitution of organohalides with a commercially available silylborane, PhMe_2_Si–B(pin) (**1**), in the presence of an alkoxide base [*i.e.*, base-mediated borylation with silylborane (BBS method), Fig. 2].^12^ This reaction proceeds smoothly in the absence of transition-metal catalysts and shows good functional group compatibility as well as high tolerance toward sterically hindered substrates. Most notably, the silylborane reagent used in this reaction performs a formal nucleophilic boryl substitution reaction with halide electrophiles. This boryl substitution is a counterintuitive reaction, in that the silylborane substrate should react with the base to generate the corresponding silyl nucleophile, as seen in the base-mediated activation of B–Si^13,14^ bonds in the absence of a transition-metal.

In our previous study,^12^ aryl-, alkenyl- and alkyl halide substrates were investigated. However, the substrate scope and limitations of the reaction were not thoroughly investigated, with a heteroaryl bromide bearing a 1-methylindol moiety and a tetrasubstituted alkenyl bromide being shown as the only examples of heteroaryl and alkenyl substrates, respectively. With regard to the reaction mechanism, the results of several experimental studies^12^ and a DFT-based theoretical study^15^ led to the proposal of a carbanion-mediated mechanism. However, further mechanistic studies are still necessary to assess the validity of the proposed mechanism.

Herein, we report the results of our recent efforts towards expanding the substrate scope of this transition-metal-free boryl substitution reaction to functionalized aryl-, heteroaryl- and alkenyl halides, and our mechanistic investigation of the BBS method. Notably, extensive investigation of the substrate scope revealed that a variety of functionalized aryl-, heteroaryl- and alkenyl halides could be successfully applied to this borylation. Furthermore, (*E*)- and (*Z*)-alkenyl iodides reacted to afford the corresponding alkenyl boronates in good to high yields with retention of the configuration, under modified reaction conditions. The results of our mechanistic experiments supported the existence of a carbanion-mediated mechanism and discounted the possibility of a radical anion-mediated mechanism.

## Results and discussion

We previously reported that the BBS method tolerates various functional groups, including chloro, fluoro, ester, amide, ether and dialkyl amino groups.^12^ In this study, we initially examine extending the functional group compatibility of the boryl substrate to include various aryl bromides, using our previously reported borylation conditions^12^ to investigate the scope of the method (Table 1). An aryl bromide bearing an epoxy group underwent the borylation reaction to give the desired product **3a** in 84% yield. *p*-Methylthiophenyl boronate **3b** was also formed in good yield (78%). Pleasingly, aryl bromides bearing an aromatic alkene moiety reacted smoothly to afford the desired products in high yields (**3c**: 85%, **3d**: 87%). Unfortunately, *p*-nitrobromobenzene (**2e**) performed poorly as a substrate and provided the desired product **3e** in low yield (8%). *p*-Bromophenylacetylene (**2f**) and *p*-bromobenzophenone (**2g**) were even less effective as substrates and gave complex mixtures. The failure of these substrates was attributed to nucleophilic attack of the silyl moiety on the keto group or the abstraction of a proton from the terminal alkyne of the substrate. The borylation of heteroaryl bromides was also investigated, and the reactions of 4-bromodibenzofuran (**2h**), 3-bromo-9-ethylcarbazole (**2i**), 2-bromodibenzothiophene (**2j**) and 5-bromobenzothiophene (**2k**) all proceeded smoothly to give the desired products in good to high yields (**3h**: 77%, **3i**: 85%, **3j**: 75%, **3k**: 51%). Substrates containing a 5-membered heterocycle, such as a thiophene, pyrazole, isoxazole, oxazole or thiazole group, also reacted efficiently to provide the corresponding heteroaryl boronates in good yields (**3l–q**: 87, 68, 67, 74, 63 and 68% yields, respectively). The application of the reaction conditions to 5-bromopyrimidine (**2r**) did not afford any of the borylated product **3r**. However, 5-bromopyrimidine derivatives bearing a methoxy or piperidyl group at their 2-position successfully underwent the borylation reaction, to give the desired products in moderate to good yield (**3s**: 38%, **3t**: 71%). This difference in the reactivity of these substrates could be attributed to the reactivity of their 2-positions towards the silyl nucleophile. 3-Bromopyridine (**2u**) and 3-bromoquinoline (**2v**) both reacted smoothly under the standard conditions to afford the borylated products in moderate to good yields (68 and 58%, respectively). Disappointingly, however, the products **3u** and **3v** could not be isolated because they decomposed during purification by silica-gel column chromatography.

**Table 1:** Boryl substitution of aryl- and heteroaryl bromides with the PhMe_2_Si–B(pin)/base reagent^*a*^ ^,^ ^*b*^

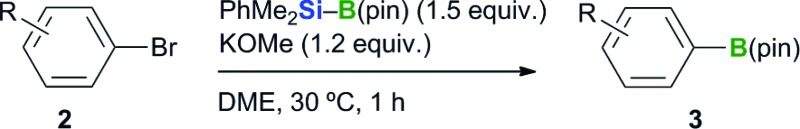
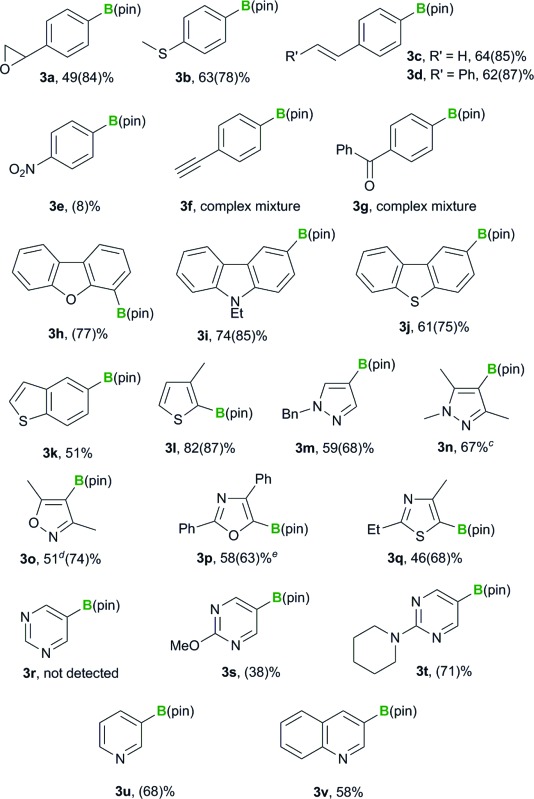

^*a*^Reaction conditions: a mixture of PhMe_2_Si–B(pin) (0.75 mmol) and KOMe (0.6 mmol) in DME (5 mL) was stirred for 10 min at 30 °C. Aryl bromide **2** (0.5 mmol) was then added, and the resulting mixture was stirred for 1 h.

^*b*^Isolated yields of product **3**, yields of **3** based on ^1^H NMR analysis are shown in parentheses.

^*c*^Isolated yield of product **3n** containing a small amount of the corresponding silylated by-product (total yield: 82%; B/Si = 81 : 19).

^*d*^Isolated yield of the borylated product **3o** containing a small amount of the corresponding silylated by-product (total yield: 57%; B/Si = 90 : 10).

^*e*^PhMe_2_Si–B(pin) (1.5 mmol) and KOMe (1.2 mmol) were used.

We then conducted sequential boryl substitution/Suzuki–Miyaura coupling procedures to demonstrate the utility of this borylation reaction (Table 2). After being quenched with TBAF to allow for the decomposition of any unreacted silylborane, the reaction mixture was subjected to an aqueous work-up procedure to give the crude borylation product, which was progressed directly into a cross-coupling reaction under conventional conditions. This reaction sequence provided facile access to compounds **4h**, **4t** and **4w** in high yields (84, 78 and 74%, respectively). However, substrate **2x** containing a nitrile group afforded the corresponding coupled product **4x** in a much lower yield (36%), which was attributed to a low yield during the initial borylation step. For substrates **2u** and **2v**, the sequential boryl substitution/Suzuki–Miyaura coupling procedure was performed in one pot without an aqueous work-up, to avoid loss of the products during the work-up process. In both cases, the sequential reaction process proceeded smoothly to afford the corresponding coupled products **4u** and **4v** in moderate to good yields (64 and 58%, respectively).

**Table 2:** Sequential boryl substitution/Suzuki–Miyaura coupling^*a*^

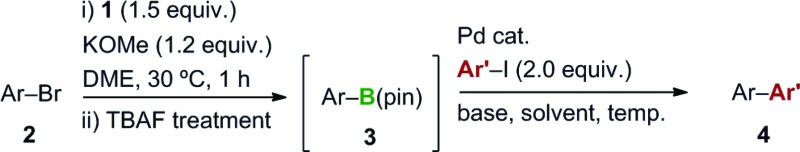
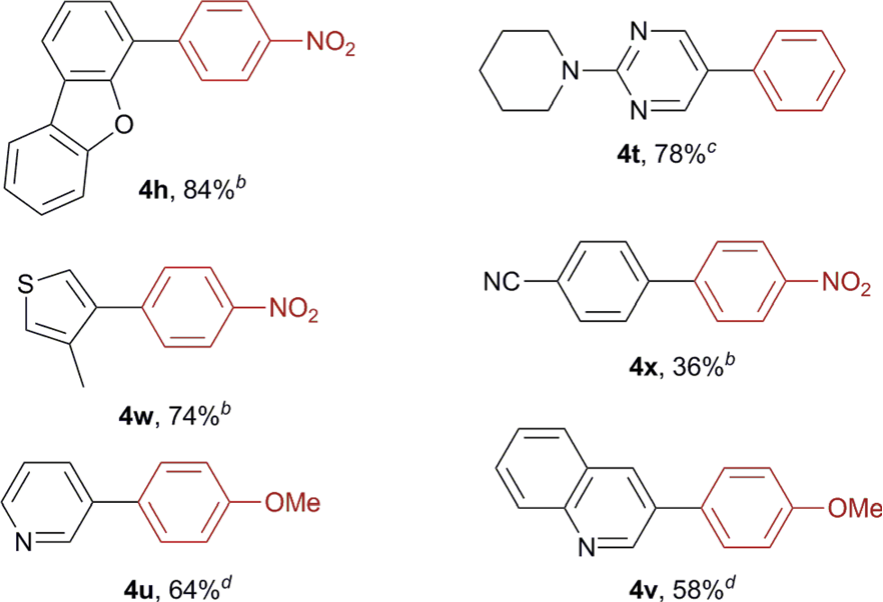

^*a*^Isolated yields.

^*b*^1-Iodo-4-nitrobenzene, K_2_CO_3_ (2.0 equiv.) and Pd(PPh_3_)_4_ (10 mol%) were used in a 10 : 1 (v/v) mixture of DMF and H_2_O at 100 °C.

^*c*^Iodobenzene, K_2_CO_3_ (2.0 equiv.) and Pd(PPh_3_)_4_ (10 mol%) were used in a 10 : 1 (v/v) mixture of DMF and H_2_O at 100 °C.

^*d*^4-Iodoanisole, K_3_PO_4_ (2.55 equiv.), PCy_3_ (3.6 mol%) and Pd_2_(dba)_3_·CHCl_3_ (1.5 mol%) were used in a 3 : 1 (v/v) mixture of 1,4-dioxane and H_2_O at 100 °C.

**Scheme 1:**
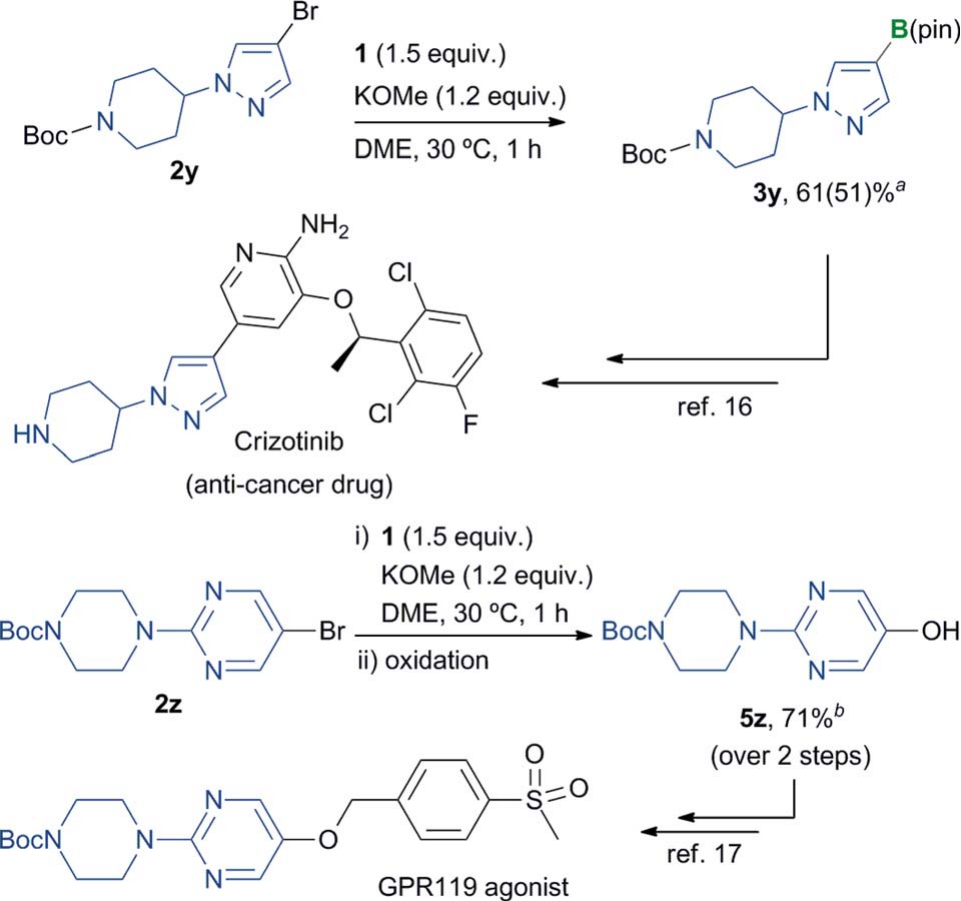
Synthetic application of the borylation reaction to the synthesis of precursors of Crizotinib and a GPR 119 agonist. ^*a*^Isolated yield of the derivatized product obtained from sequential borylation/NaBO_3_·4H_2_O oxidation of **2y** over two steps. For details, see the ESI.† Yield of **3y** based on ^1^H NMR analysis is shown in parentheses. ^*b*^Isolated yield of product **5z**.

The overall utility of this borylation process was further demonstrated through the synthesis of key precursors in the production of Crizotinib^16^ and a GPR119 agonist^17^ (Scheme 1). The borylation of pyrazolyl bromide **2y** proceeded smoothly to give the corresponding borylated product **3y** in good yield. Borylation of the 5-bromopyrimidine derivative **2z** followed by oxidation of the resulting product gave **5z**, which is a precursor of the GPR 119 agonist, in good yield (71% yield, over 2 steps).

**Scheme 2:**
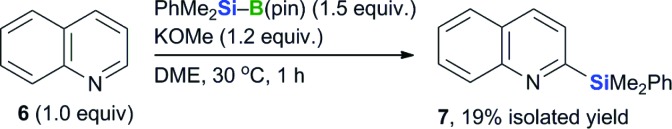
Reaction of quinoline under the boryl substitution conditions.

Results obtained during the course of our investigation into the substrate scope of the borylation reaction with heteroaryl halides indicated that quinoline (**6**) undergoes silyl addition under the conditions used in the BBS method to give 2-silylated quinoline **7** in an isolated yield of 19% (Scheme 2). It is noteworthy that reports pertaining to silyl substitution reactions with quinolines or pyridines using a silyl nucleophile *via* a transition-metal-free dearomatization are scarce.^18–20^ This silylation reaction therefore represents a possible pathway for the formation of by-products when heteroaryl halides are used as substrates in the borylation reaction. These results also suggest that a silyl nucleophile is formed under the conditions used in the BBS method.

With regard to the preparation of alkenyl boronates, the thermal hydroboration of alkynes is a common and straightforward method for the synthesis of (*E*)-alkenyl borates.^21^ In contrast, (*Z*)-alkenyl boronates are usually prepared *via* an indirect *trans*-hydroboration reaction using alkynyl bromides, which requires highly reactive nucleophilic reagents such as alkenyl lithium compounds.^22^ With this in mind, we proceeded to investigate the borylation of (*Z*)-alkenyl iodide **8a** under a variety of different conditions (Table 3). Our initial efforts in this area focused on applying our previously determined optimum conditions for the boryl substitution of aryl bromides to an alkenyl halide substrate.^12^ The reaction of (*Z*)-**8a** with PhMe_2_Si–B(pin) (1.5 equiv.) in the presence of KOMe (1.2 equiv.) in DME at 30 °C afforded the (*Z*)-product in moderate yield and with high levels of both (*Z*)- and borylation/silylation (B/Si) selectivity (Table 3, entry 1, 53%, *Z*/*E* = 92 : 8, B/Si = 95 : 5). In addition, vinylcyclohexane, which is a protonated product of **8a**, was observed as the major by-product under these reaction conditions. The use of NaOMe instead of KOMe successfully suppressed the formation of the by-product and led to an improvement in the yield, as well as in the *Z*/*E* and B/Si selectivities (Table 3, entry 2, 72%, *Z*/*E* = 96 : 4, B/Si = 96 : 4). Furthermore, the use of NaOEt as the base led to a further increase in the yield of the desired (*Z*)-borylated product compared with NaOMe (Table 3, entry 3, 79%, *Z*/*E* = 97 : 3, B/Si = 97 : 3). Finally, increasing the amount of silylborane **1** to 2.0 equivalents relative to the substrate afforded the highest yield of the product boronate observed during this optimization process (Table 3, entry 4, 89%, *Z*/*E* = 97 : 3, B/Si = 96 : 4). Several other bases were also investigated (Table 3, entries 5–8). No reaction occurred when LiOMe was used as the base (Table 3, entry 5), and the use of bulky alkoxide bases, such as K(O-*t*-Bu) and Na(O-*t*-Bu), afforded much lower yields of the product than NaOEt, as well as lower selectivities (Table 3, entries 6 and 7). The use of a weaker base (*i.e.*, NaOTMS) did not lead to significant improvements in the yield or selectivity of the reaction (Table 3, entry 8). The reaction also proceeded when THF or 1,4-dioxane was used instead of DME, although the products were formed in lower yields in both cases (Table 3, entries 9 and 10). The use of CH_2_Cl_2_ or toluene as the solvent afforded only trace amounts of the borylated product (Table 3, entries 11 and 12).

**Table 3:** Boryl substitution of (*Z*)-alkenyl iodide with the PhMe_2_Si–B(pin)/base reagent^*a*^

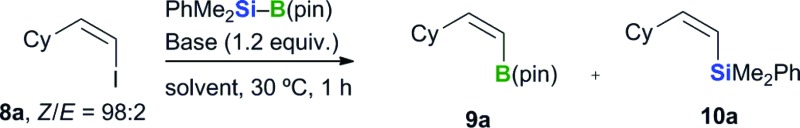						
Entry	Base	Si–B (equiv.)	Solvent	Yield of **9a** ^*b*^ (%)	*Z*/*E* of **9a** ^*c*^ (%)	B/Si^*d*^
1	KOMe	1.5	DME	53	92 : 8	95 : 5
2	NaOMe	1.5	DME	72	96 : 4	96 : 4
3	NaOEt	1.5	DME	79	97 : 3	97 : 3
4	NaOEt	2.0	DME	89(71)	97 : 3	96 : 4
5	LiOMe	1.5	DME	0		
6	K(O-*t*-Bu)	1.5	DME	27	83 : 17	68 : 32
7	Na(O-*t*-Bu)	1.5	DME	41	95 : 5	74 : 26
8	NaOTMS	1.5	DME	65	89 : 11	92 : 8
9	NaOEt	2.0	THF	81	96 : 4	97 : 3
10	NaOEt	2.0	1,4-Dioxane	73	98 : 2	99 : 1
11	NaOEt	2.0	CH_2_Cl_2_	2		
12	NaOEt	2.0	Toluene	0		

^*a*^Reaction conditions: a mixture of PhMe_2_Si–B(pin) and base (0.6 mmol) in solvent (5 mL) was stirred for 10 min at 30 °C. (*Z*)-(2-Iodovinyl)cyclohexane **8a** (*Z*/*E* = 98 : 2, 0.5 mmol) was then added to the reaction, and the resulting mixture was stirred for 1 h.

^*b*^GC yields. Isolated yields are shown in parentheses.

^*c*^ *Z*/*E* ratios were determined based on GC analysis.

^*d*^Ratios of the borylated (**9a**) and silylated (**10a**) products.

With the optimized conditions in hand (Table 3, entry 4), we proceeded to investigate the substrate scope for various alkenyl halide substrates (Table 4). In most cases, the *Z*/*E* ratios of the substrates employed for investigating the substrate scope were >98.5 : 1.5 (for details, see the ESI†), and only less than trace amounts of geometric isomers (<5%) were detected in the borylated products based on GC and ^1^H NMR analysis. The reaction of alkenyl iodide (*E*)-**8a** proceeded in high yield with retention of the configuration [(*E*)-**9a**, 86%]. Several other (*Z*)-alkenyl iodides bearing *n*-pentyl or phenethyl groups also reacted smoothly under these conditions, to provide the corresponding borylated products (*Z*)-**9b** and (*Z*)-**9c** in 83 and 80% yields, respectively. The boryl substitution reactions of α,α-disubstituted alkenyl bromide **8d** and trisubstituted alkenyl iodide (*Z*)-**8e** proceeded as anticipated to give the corresponding borylated products in moderate to good yields [**9d**: 43%, (*E*)-**9e**: 64%]. The tetrasubstituted alkenyl bromide **8f** also reacted effectively, albeit under modified conditions, to provide the corresponding alkenyl boronate **9f**, although the yield was similar to that achieved under the previously reported conditions [*i.e.*, **1** (1.5 equiv.) and KOMe (1.2 equiv.) in DME at 30 °C for 1 h, 58%].^12^ Functionalized (*Z*)-alkenyl iodides containing a benzoyl or acetal group also successfully underwent the boryl substitution reaction to afford the desired products in good yields [(*Z*)-**9g**: 70%, (*Z*)-**9h**: 74%]. A cyclic alkenyl boronate containing a MOM group **9i** was also formed in good yield under the optimized conditions (58%). For cyclic alkenyl iodides and pseudohalides containing a dihydropyranyl moiety, the C=C double bond can undergo positional isomerisation under the Pd-catalyzed borylation conditions, which can lead to a decrease in the yield of the desired alkenyl boronate.^5a,23^ However, under the current borylation conditions, substrate **8j** bearing a dihydropyranyl moiety gave the desired oxacyclic alkenyl boronate **9j** in good yield (71%). The sterically hindered cyclic alkenyl bromide **8k** bearing a butyl ester moiety also provided the desired borylated product **9k** in good yield (53%).

**Table 4:** Substrate scope of the boryl substitution reaction with the PhMe_2_Si–B(pin)/base reagent^*a*^ ^,^ ^*b*^

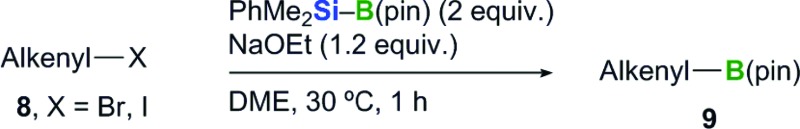
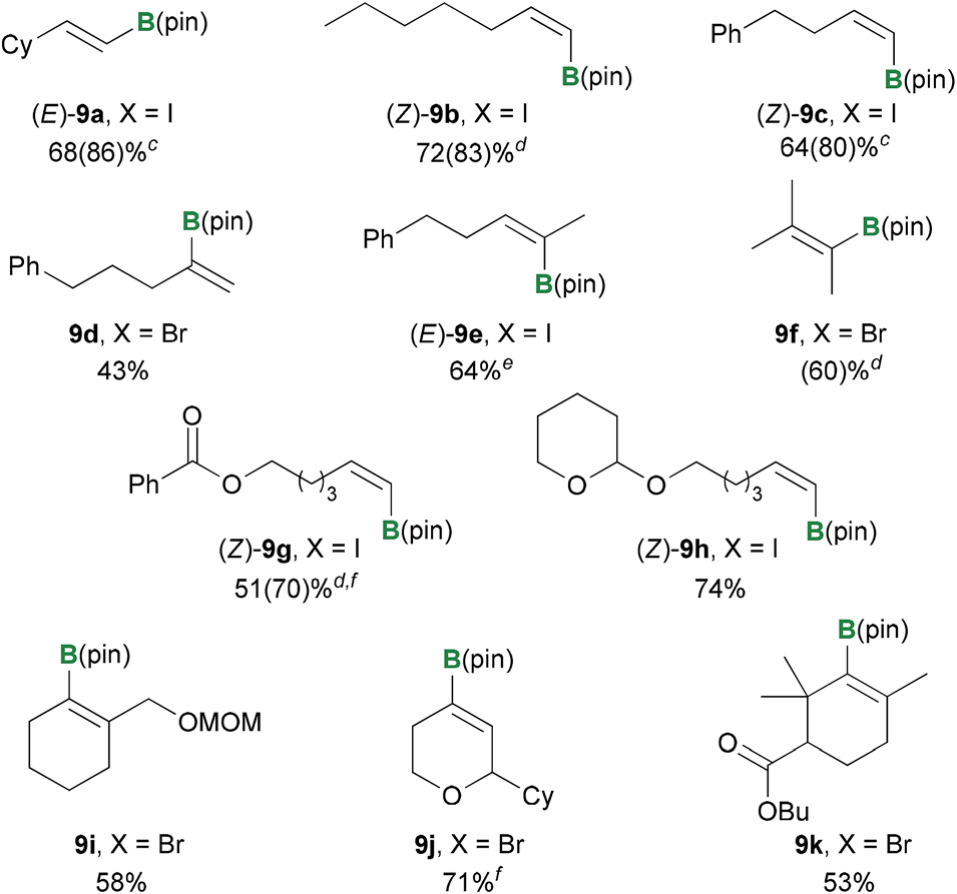

^*a*^Reaction conditions: a mixture of PhMe_2_Si–B(pin) (1.0 mmol) and base (0.6 mmol) in solvent (5 mL) was stirred for 10 min at 30 °C. Alkenyl halide **8** (0.5 mmol) was then added to the reaction, and the resulting mixture was stirred for 1 h.

^*b*^Isolated yields.

^*c*^Yield based on GC analysis is shown in parentheses.

^*d*^Yield based on ^1^H NMR analysis is shown in parentheses.

^*e*^Substrate **8e** had a *Z*/*E* ratio of 6 : 94, and no significant amount of (*Z*)-**9e** was contained in the isolated product based on NMR analysis.

^*f*^Isolated yield was determined after Suzuki–Miyaura cross coupling. Details are described in the ESI.

### Mechanistic studies

**Scheme 3:**
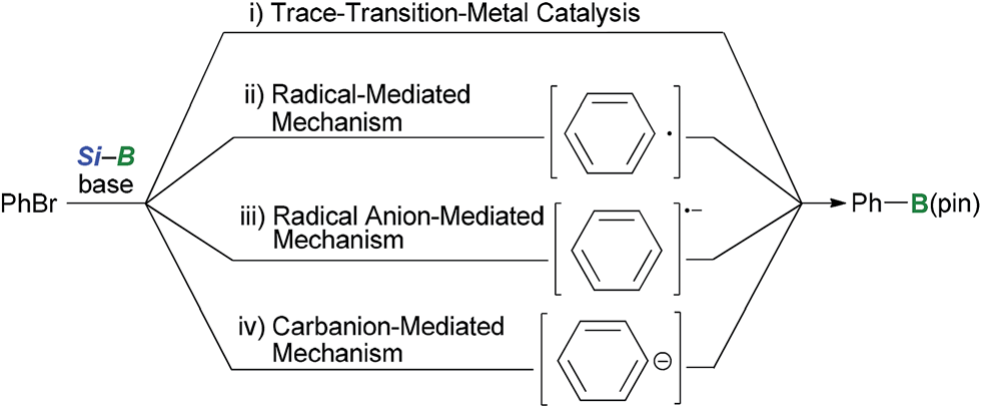
Four possible reaction pathways for the BBS method.

It was initially assumed that the boryl substitution reaction could proceed according to one of four possible reaction pathways, including: (1) trace-transition-metal catalysis, (2) a radical-mediated mechanism, (3) a radical-anion-mediated mechanism or (4) a carbanion-mediated mechanism (Scheme 3). Several experiments were conducted in our previous paper to investigate the reaction mechanism.^12^ The possibility of trace-transition-metal catalysis was investigated by analyzing the alkoxide base for the presence of various transition-metals (*i.e.*, Ni, Pd, Pt, Rh, Au, Ag, Ir, Ru and Co) using ICP-AES. The results of this analysis revealed that the contamination resulting from trace-transition metals was significantly low (*e.g.*, 3.0 ppm for Co, Ni, Ir and Pd, and 2.0 ppm for Ag, Pt, Rh, Ru and Au). Furthermore, the borylation reaction was repeated in the presence of transition-metal salts, which showed no acceleration in the yield or selectivity of the reaction. Several experiments were also conducted to probe the possibility of a radical-mediated mechanism, such as reaction of *o*-butenylbromobenzene under the optimized conditions and a borylation in the presence of a radical scavenger. The results of our previous study^12^ suggested that the borylation reaction does not involve trace-transition-metal catalysis or a radical-mediated mechanism. However, the possibility of a radical-mediated mechanism was not completely ruled out by the results of our previous study. Furthermore, the possibility of the reaction occurring *via* a radical-anion- or carbanion-mediated mechanism has still not been investigated. Thus, further mechanistic studies were needed to elucidate the mechanism of this reaction.

The occurrence of a radical-mediated mechanism can be discounted based on the results of the stereoretentive borylation of (*Z*)-alkenyl halides. This borylation reaction could proceed *via* a radical mechanism if a radical species can be generated from an organohalide *via* a SET process involving a silylborane/alkoxy-base ate complex under basic conditions. For example, Strohmann *et al.* ^24^ reported that a radical-mediated mechanism was involved in the silyl substitution reaction of aryl iodides with an α-aminosilyllithium reagent. Further investigation of the borylation of (*Z*)-alkenyl halides revealed that the reaction proceeded in a perfectly stereoretentive manner, as shown in Tables 3 and 4. It was therefore envisaged that the generation of a corresponding vinyl radical species would lead to the *E*/*Z*-isomerization of the intermediate, which would subsequently lead to a lower *E*/*Z* ratio of the product (eqn (1)).^25^ These experimental results therefore suggest that a carbanion-mediated mechanism is more likely than a radical-mediated mechanism.
1

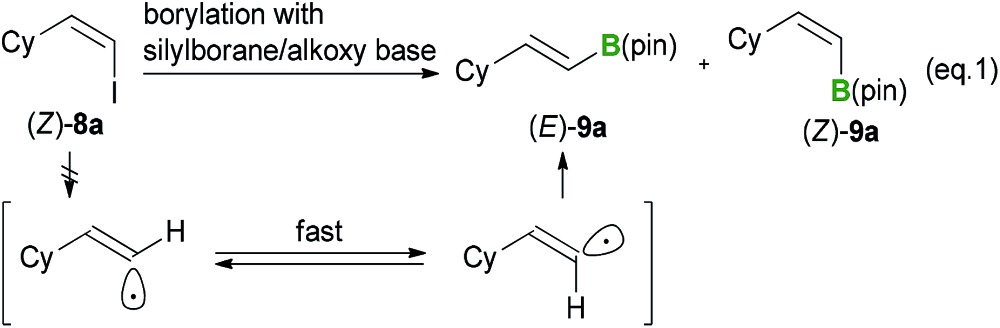




**Scheme 4:**
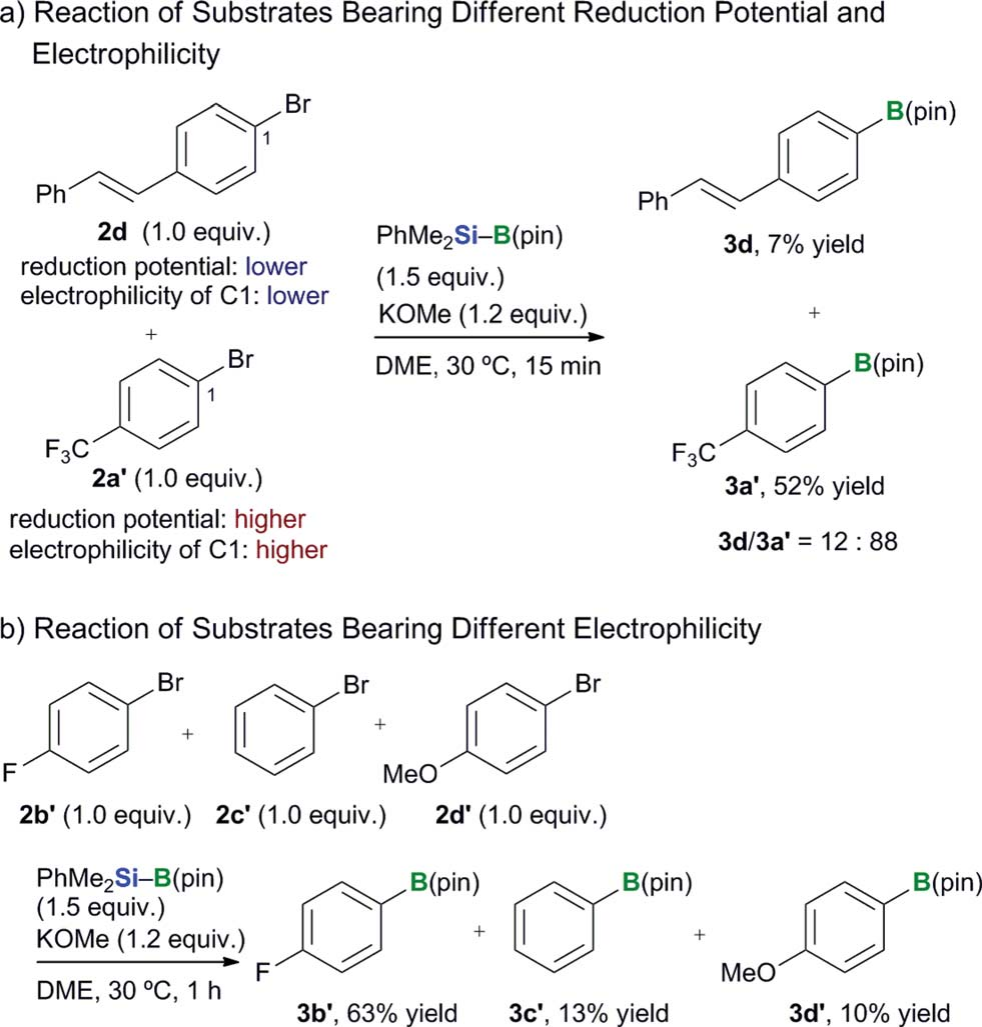
Competition reactions between aryl bromides.

Several competition experiments were conducted to develop further mechanistic insight into this transformation. We initially performed a competition reaction with two different aryl bromides to investigate the involvement of a radical- or radical-anion-mediated mechanism (Scheme 4a). (*E*)-*p*-Bromostilbene (**2d**) has a lower reduction potential than 4-bromo(trifluoromethyl)benzene (**2a′**), and the carbon atom bound to the bromine in the latter of these two substrates is much more electrophilic than that of the former.^26^ The competition reaction with these two halides afforded the borylated products **3d** and **3a′** in a ratio of 12 : 88. A further competition reaction was performed using three aryl bromides (**2b′–d′**) with different levels of electrophilicity (Scheme 4b). The borylation of 4-bromo(fluoro)benzene (**2b′**) proceeded much more rapidly than for the other two substrates, which were less electrophilic. These observations therefore support the existence of a carbanion-mediated mechanism rather than a radical- or radical-anion-mediated mechanism, and are also in good agreement with the results of the DFT study reported in our other paper.^15^

**Scheme 5:**
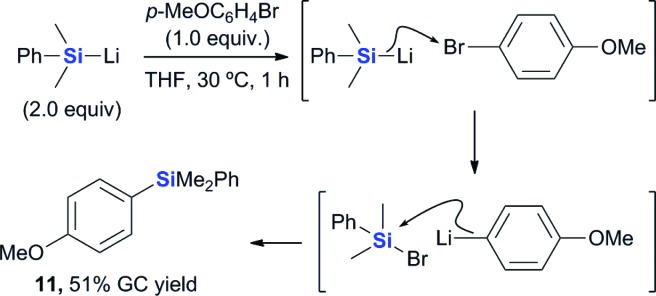
Reaction of *p*-bromoanisole with a silyl nucleophile.

We then proceeded to investigate the possibility of a carbanion-mediated mechanism using a silyl nucleophile. Consideration of the carbanion-mediated mechanism revealed that the halogenophilic attack by the silyl nucleophile would be the key reaction in the borylation process. It is noteworthy, however, that this reaction has not yet been studied in great detail.^27,28^ With this in mind, we performed the silyl substitution reaction of an aryl bromide with (dimethylphenylsilyl)lithium, which is a common silyllithium reagent (Scheme 5). The silyl substitution of *p*-bromoanisole (**2d′**) with the (dimethylphenyl)silyl lithium proceeded smoothly to provide the corresponding silylated product **11** in 51% yield. This reaction most probably involved an initial halogenophilic attack by the silyl nucleophile on the bromine atom, followed by reaction of the resultant silyl bromide with the aryl lithium species to provide the silyl substitution product **11**. This result provides further evidence in support of the formation of an aryl anion intermediate from the reaction of aryl halides with the silyl nucleophile generated by the reaction between PhMe_2_Si–B(pin) and an alkoxide base.

**Scheme 6:**
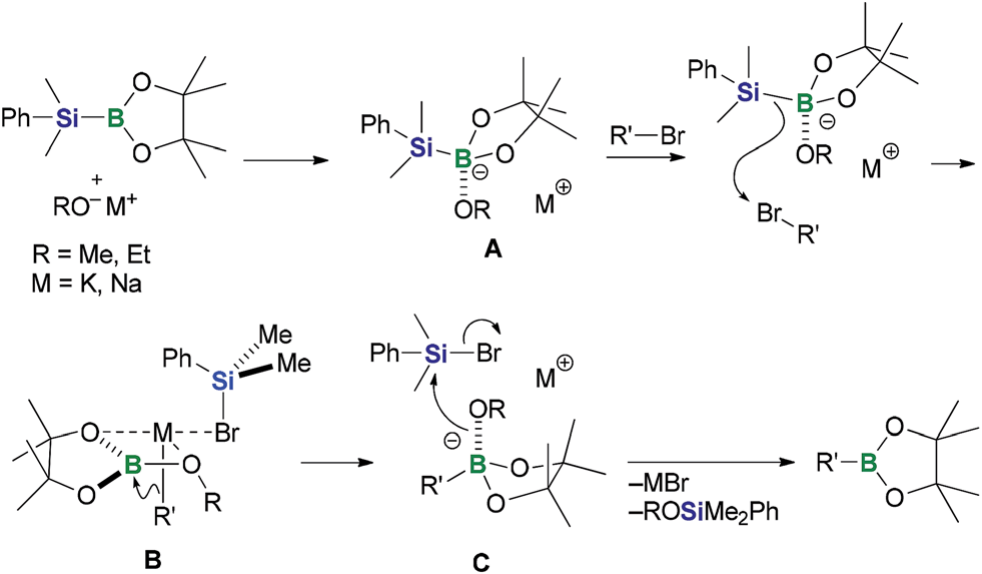
Plausible reaction mechanism.

Based on the experimental findings presented above, we have proposed a plausible carbanion-mediated mechanism for the BBS method, involving a halogenophilic attack by the silyl anion on the bromine atom of the substrate (Scheme 6).^12,15^ PhMe_2_Si–B(pin) would initially react with the alkoxide base to give the silylborane/alkoxy-base complex **A**. Subsequent nucleophilic attack by the nucleophilic silyl moiety of complex **A** on the bromine atom of an organobromine substrate would lead to the formation of complex **B**, which contains a carbanion species. The carbanion species would then attack the boron electrophile rather than the PhMe_2_SiBr generated *in situ* to give the corresponding organoborate intermediate **C**, which would then react with the silyl bromide to give the corresponding organoboronate ester together with ROSiMe_2_Ph and a bromide salt, which would be formed as by-products. Based on this mechanism, the nature of the counter cation and the bulkiness of the base activator would be expected to have a significant impact on the yield and B/Si selectivity. For the alkenyl halide substrate **8a**, sodium alkoxides such as NaOEt and NaOMe were found to be more suitable as base activators than KOMe, which afforded the best results when aryl bromides were used as substrates. Furthermore, the use of KOMe resulted in the production of vinylcyclohexane as a major by-product. These results could be attributable to the basicity of the carbanion^29^ in complex **B**: the high basicity of the vinyl potassium species in complex **B** would lead to the formation of the by-product through the abstraction of a proton. With regard to the effect of the bulkiness of the base, the results of the reactions presented in Table 3 with a *t*-butoxide base indicate that the presence of a bulky substituent on the base lowers the B/Si selectivity by increasing the kinetic stability of the boron electrophile in complex **B**, which would prevent this complex from being attacked by the carbanion species.

## Conclusions

In summary, we have successfully expanded the scope of our borylation reaction using silylborane and an alkoxide base to a variety of functionalized aryl-, heteroaryl- and alkenyl halides. It is noteworthy that the reactions of the (*Z*)-alkenyl halides also proceeded in a stereoretentive manner. Furthermore, we have demonstrated the overall utility of this reaction for the borylation of heteroaryl substrates by synthesizing the precursors involved in the preparation of Crizotinib and a GPR119 antagonist. This reaction has also been applied to the development of a sequential boryl substitution/Suzuki–Miyaura coupling reaction. Furthermore, the results of competition experiments and the reaction of a silyllithium reagent with an aryl bromide support the occurrence of a carbanion-mediated mechanism, rather than a radical- or radical-anion-mediated mechanism. It is envisaged that the results of this study will lead to the development of new strategies for the preparation of organoboron and organosilane compounds, as well as to a deeper understanding of boron and silicon chemistry.
