# Supplementary material for: Boryl substitution of functionalized aryl-, heteroaryl- and alkenyl halides with silylborane and an alkoxy base: expanded scope and mechanistic studies
Source: Chem Sci. 2015 Mar 2;6(5):2943–51. doi: 10.1039/c5sc00384a (PMC5656038; doi:10.1039/c5sc00384a)
Supplement: Supplementary file 1 [file SC-006-C5SC00384A-s001.pdf]

Electronic Supplementary Material (ESI) for Chemical Science.
This journal is © The Royal Society of Chemistry 2015

Boryl Substitution of Functionalized Aryl-, Heteroaryl- and Alkenyl Halides with Silylborane and Alkoxy Base: Expanded Scope and Mechanistic Studies

Eiji Yamamoto, Satoshi Ukigai and Hajime Ito*

Division of Chemical Process Engineering and Frontier Chemistry Center, Faculty of Engineering, Hokkaido University, Sapporo 060-8628, Japan, FAX: +81-(0)11-706-6561
e-mail: hajito@eng.hokudai.ac.jp

Table of Contents

1. General and Materials
2. Typical Procedures
3. Preparation of Aryl- and Alkenyl Halides
4. Characterization of Boryl Substitution Products
5. References
6. ^{1}H and ^{13}C NMR Spectra

1. General and Materials

Materials were obtained from commercial suppliers and purified by standard procedures unless otherwise noted. Solvents for the reaction were also purchased from commercial suppliers. 1,2-Dimethoxyethane (DME) was distilled from sodium benzophenone ketyl, and further dried over molecular sieves (MS 4A). KOMe (95%) was purchased from Aldrich and used as received. NaOEt (95%) was purchased from Tokyo Chemical Industry (TCI) and used as received. $PhMe_2Si$−B(pin) was prepared according to reported procedures[1] or provided by Frontier Scientific, Inc. NMR spectra were recorded on JEOL JNM-ECX400P and ECS-400 spectrometer (^{1}H: 400 MHz and ^{13}C: 100 MHz). Tetramethylsilane (^{1}H) and $CDCl_3$ (^{13}C) were employed as external standards, respectively. Multiplicity was reported as follows: s = singlet, brs = broad singlet, d = doublet, t = triplet, q = quartet, quint = quintet, m = multiplet. Mesitylene was used as the internal standard for determining NMR yield. GLC analyses were conducted with a Shimadzu GC-2014 or GC-2025 equipped with ULBON HR-1 glass capillary column (Shinwa Chemical Industries) and a FID detector. 1,4-Diisopropylbenzene was used as the internal standard for determining GC yield. NMR yields were determined from ^{1}H NMR analysis of the crude mixture. Recycle preparative gel permeation chromatography (GPC) was conducted with a JAI LC-9101 using $CHCl_3$ as the eluent.

High-resolution mass spectra were recorded at the Center for Instrumental Analysis, Hokkaido University.

2. Typical Procedures

2-1. Typical Procedures for Boryl Substitution of Aryl Halide 2c: Procedure A (Table 1)

Potassium methoxide (42.1 mg, 0.60 mmol, 1.2 equiv) was placed in a vial with a screw cap containing a silicon-coated rubber septum in a glove box under argon atmosphere. After the reaction vial was removed from the glove box, DME (5 mL) and (dimethylphenylsilyl)boronic acid pinacol ester (196.7 mg, 0.75 mmol, 1.5 equiv) were added to the vial through the septum with a syringe, then stirred for 10 min at 30 ºC. Aryl halide **2c** (92.0 mg, 0.50 mmol, 1.0 equiv) was added dropwise with a syringe. After 1 h, the reaction mixture was analyzed by GC to check completeness of the reaction and NMR yield was determined by ^{1}H NMR analysis of the crude reaction mixture. Then to remove unreacted (dimethylphenylsilyl)boronic acid pinacol ester and a byproduct, methoxydimethylphenylsilane, the solution was cooled to below −5 ºC followed by addition of TBAF (0.5 M THF solution, 1.6 mL). The resultant solution was stirred for 2 h at the same temperature. After that, H_2O was added to the mixture, then extracted three times with Et_2O. The organic layer was washed with water. The combined organic layer was then dried over $MgSO_4$ followed by filtration and evaporation. The crude product was purified by silica-gel column chromatography with 0−3% hexane/Et_2O eluent to give the borylated product **3c** (73.5 mg, 0.32 mmol, 64% isolated yield) as a colorless oil.

2-2. Typical Procedures for Sequential Boryl Substitution and Suzuki-Miyaura Coupling: Procedure B (Table 2)

Potassium methoxide (42.1 mg, 0.60 mmol, 1.2 equiv) was placed in a vial with a screw cap containing a silicon-coated rubber septum in a glove box under argon atmosphere. After the reaction vial was removed from the glove box, DME (5 mL) and (dimethylphenylsilyl)boronic acid pinacol ester (197.5 mg, 0.75 mmol, 1.5 equiv) were added to the vial through the septum with a syringe, then stirred for 10 min at 30 ºC. Aryl halide **2h** (124.0 mg, 0.50 mmol) was added dropwise with a syringe. After 1 h, the reaction mixture was analyzed by GC to check completeness of the reaction and NMR yield was determined by ^{1}H NMR analysis of the crude reaction mixture. Then to remove unreacted (dimethylphenylsilyl)boronic acid pinacol ester and a byproduct, methoxydimethylphenylsilane, the solution was cooled to −7 ºC followed by addition of TBAF (0.5 M THF solution, 1.6 mL). The resultant solution was stirred for 2 h at the same temperature. After

that, H_2O was added to the mixture, then extracted three times with Et_2O. The organic layer was washed with water. The combined organic layer was then dried over $MgSO_4$ followed by filtration and evaporation. The resultant reaction mixture was transferred to a 20 mL-Schlenk flask with a magnetic stirrer bar and the solvent was removed under a reduced pressure. Then, the flask was connected to a vacuum-nitrogen manifold, and it was evacuated and refilled with nitrogen three times. DMF (4 mL), N_2-bubbled H_2O (0.4 mL), K_2CO_3 (138.8 mg, 1.00 mmol, 2.0 equiv), 1-iodo-4-nitrobenzene (249.5 mg, 1.00 mmol, 2.0 equiv) and $Pd(PPh_3)_4$ (58.0 mg, 0.05 mmol, 10 mol %) were successively added to the flask. The solution was heated to 100 ºC and stirred for 2 h. After that, the reaction mixture was cooled to room temperature, and H_2O was added to the mixture and extracted three times with EtOAc. The combined organic layer was dried over $MgSO_4$ followed by filtration and evaporation. The crude product was purified by silica-gel column chromatography with 0–50% hexane/EtOAc eluent to give the corresponding coupling product **4h** in 84% isolated yield over two steps [122.1 mg, 0.422 mmol, (77% NMR yield of **3h** in the crude mixture)].

2-3. Typical Procedures for Boryl Substitution of Alkenyl Halides: Procedure C (Table 4)

Sodium ethoxide (40.8 mg, 0.60 mmol) was placed in a vial with a screw cap containing a silicon-coated rubber septum in a glove box under argon atmosphere. After the vial was removed from the glove box, DME (5 mL) and (dimethylphenylsilyl)boronic acid pinacol ester (263.7 mg 1.00 mmol) were added to the vial through the septum with a syringe, then stirred for 10 min at 30 ºC. Alkenyl halide **8a** (119.7 mg, 0.507 mmol) was added dropwise with a syringe. After 1 h, the reaction mixture was analyzed by GC and ^{1}H NMR spectroscopy to check the progress of the reaction. Then, the solution was cooled to below −5 ºC followed by addition of TBAF (0.5 M THF solution, 2.0 mL) to remove unreacted (dimethylphenylsilyl)boronic acid pinacol ester and a byproduct, methoxydimethylphenylsilane. The resultant solution was stirred for 2 h at the same temperature. After that, the mixture was passed through a thin-pad of silica-gel to give a crude product followed by evaporation. The resulting crude product was purified by silica-gel column chromatography with 0−3% hexane/Et_2O eluent to give the borylated product **9a** [84.7 mg, 0.359 mmol, 71% isolated yield (89% GC yield)] as a colorless oil.

2-4. Procedures for Competition Reaction between Aryl Bromides (Scheme 4a)

Potassium methoxide (0.60 mmol, 42.2 mg) was placed in a vial with a screw cap containing a silicon-coated rubber septum in a glove box under argon atmosphere. After the reaction vial was removed from the glove box, a solution of (*E*)-4-bromostilbene **2d** (129.6 mg, 0.500 mmol) and

4-bromo(trifluoromethyl)benzene **2a'** (113.5 mg, 0.504 mmol) in DME (4 mL) was added to the vial and washed twice with 0.5 mL of DME. Then stirred for 10 min at 30 ºC, (dimethylphenylsilyl)boronic acid pinacol ester (197.9 mg, 0.755 mmol) was added. During the reaction, 1,4-diisopropylbenzene was added to the reaction mixture as an internal standard. After 15 min later, the reaction mixture was analyzed by GC to determine the yields of the borylated products **3d** and **3a'** (7%, 52%, respectively).

2-5. Procedures for Competition Reaction of Aryl Halides (Scheme 4b)

Potassium methoxide (42.1 mg, 0.60 mmol) was placed in a vial with a screw cap containing a silicon-coated rubber septum in a glove box under argon atmosphere. After the reaction vial was removed from the glove box, DME (5.0 mL) and (dimethylphenylsilyl)boronic acid pinacol ester (194.7 mg, 0.74 mmol) were added to the vial through the septum with a syringe and stirred at 30 ºC. After 10 min, a mixture of *p*-bromoanisole (93.6 mg, 0.5 mmol), *p*-fluorobromobenzene (87.5 mg, 0.5 mmol) and bromobenzene (78.6 mg, 0.50 mmol) in DME (0.25 mL) was added to the vial and washed three times with 0.25 mL of DME. The resultant mixture was stirred at the same temperature. During the reaction, 1,4-diisopropylbenzene was added to the reaction mixture as an internal standard. After 1 h, the reaction mixture was analyzed by GC to determine the yields of the borylated products **3b'**, **3c'** and **3d'** (63%, 13%, 10%, respectively).

2-6. Procedures for Reaction of *p*-Bromoanisole with Silyl Nucleophile (Scheme 5)

An oven-dried 20 mL-Schlenk flask was connected to a vacuum-nitrogen manifold, and it was evacuated and refilled with nitrogen three times.. $PhMe_2SiLi$ solution (2.0 mL, 0.815 M in THF) prepared according to reported procedures[2] was added to the flask. Then, *p*-bromoanisole (152.0 mg, 0.813 mmol) was added to the solution through the septum with a syringe at 30 ºC and stirred. Increase of temperature of the reaction mixture was observed during the addition of *p*-bromoanisole. After 1 h, the reaction was quenched with water, then extracted ten times with Et_2O. The combined organic layer was dried over $MgSO_4$ followed by filtration and evaporation. Then, 1,4-diisopropylbenzene (43.3 mg) was added to the resultant crude mixture as an internal standard, and the sample was analyzed by GC to determine the yield of silylated product **11** (51%).

3. Preparation of Aryl- and Alkenyl Halides

Aryl halides **2d,**[3] **2j,**[4] **2p,**[5] **2q,**[6] **2y,**[7] **2z**[8] and (*Z*)-6-Iodohex-5-en-1-ol,[9] 2-bromo-1-cyclohexenemethanol,[10] 3-bromo-2,4-dimethyl-penta-1,3-diene[11] and alkenyl halides (*E*)-

and (*Z*)-**8a**,[12, 13] (*Z*)-**8b**,[14] (*Z*)-**8c**,[15] **8d**,[16] **8e**,[17] and **8j**[18] were synthesized according to literature procedures. Aryl halides **2u** and **2v** were distilled from CaH_2 and further dried over molecular sieves (MS 4A) prior to use.

Synthesis of (*Z*)-8g.

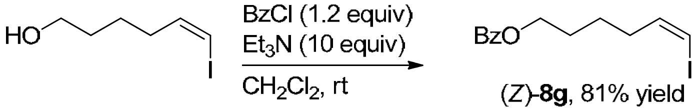

A solution of (*Z*)-6-iodohex-5-en-1-ol (678.2 mg, 3 mmol) and benzoyl chloride (506 mg, 3.6 mmol) in dry CH_2Cl_2 (10 mL) was cooled to 0 °C, then Et_3N (4.18 mL, 30 mmol) was added dropwise to the solution. The resultant mixture was allowed to warm to room temperature and stirred for 16 h. The progress of the reaction was monitored by TLC. The reaction mixture was concentrated and passed through a thin-pad of silica-gel. The resultant solution was evaporated and purified by silica-gel column chromatography with hexane/Et_2O eluent to give alkenyl iodide (*Z*)-**8g** in 81% yield [*Z*/*E* = 100 : 0 (based on GC analysis)].

^{1}H NMR (392 MHz, $CDCl_3$, δ): 1.56−1.68 (m, 2H), 1.76−1.86 (m, 2H), 2.23 (q, *J* = 7.1 Hz, 2H), 4.35 (t, *J* = 6.4 Hz, 2H), 6.15−6.27 (m, 2H), 7.41−7.47 (m, 2H), 7.56 (tt, *J* = 1.6, 7.4 Hz, 1H), 8.03−8.08 (m, 2H). ^{13}C NMR (99 MHz, $CDCl_3$, δ): 24.5 (*C*H_2), 28.1 (*C*H_2), 34.2 (*C*H_2), 64.7 (*C*H_2), 83.0 (*C*H), 128.3 (*C*H), 129.5 (*C*H), 130.3 (*C*), 132.8 (*C*H), 140.6 (*C*H), 166.6 (*C*). HRMS-ESI (*m*/*z*): $[M+Na]^+$ calcd for $C_{13}H_{15}O_2INa$, 353.00089; found, 353.00109.

Synthesis of (*Z*)-8h.

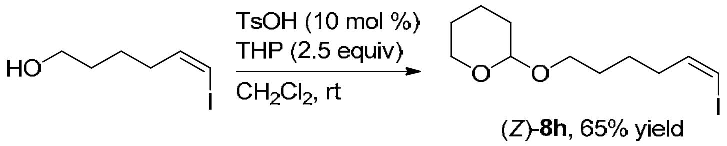

A solution of (*Z*)-6-iodohex-5-en-1-ol (1.13 g, 5 mmol) and TsOH (90 mg, 0.5 mmol) in dry CH_2Cl_2 (10 mL) was cooled to 0 °C, then dihydropyrane (1.05 g, 12.5 mmol) was added dropwise to the solution. The resultant mixture was allowed to warm to room temperature and stirred for 1 h. The progress of the reaction was monitored by TLC. The reaction was quenched with water, then extracted three times with CH_2Cl_2. The combined organic layer was dried over Na_2SO_4 followed by filtration and evaporation. The crude product was purified by silica-gel column chromatography with 1−3% hexane/EtOAc eluent, then further purified by Kugelrohr distillation under a reduced pressure to give alkenyl iodide (*Z*)-**8h** in 65% yield [*Z*/*E* = 100 : 0 (based on GC analysis)].

^{1}H NMR (392 MHz, $CDCl_3$, δ): 1.46−1.76 (m, 9H), 1.76−1.90 (m, 1H), 2.12−2.23 (m, 2H), 3.41 (dt, *J* = 6.4, 9.6 Hz, 1H), 3.47−3.55 (m, 1H), 3.76 (dt, *J* = 6.5, 9.7 Hz, 1H), 3.83−3.91 (m, 1H), 4.55−4.61 (m, 1H), 6.14−6.22 (m, 2H). ^{13}C NMR (99 MHz, $CDCl_3$, δ): 19.6 (CH_2), 24.7 (CH_2), 25.4 (CH_2), 29.1 (CH_2), 30.7 (CH_2), 34.5 (CH_2), 62.3 (CH_2), 67.2 (CH_2), 82.5 (*C*H), 98.8 (*C*H), 141.1 (*C*H). HRMS-ESI (*m*/*z*): $[M]^+$ calcd for $C_{11}H_{19}O_2INa$, 333.03219; found, 333.03228.

Synthesis of 8j.

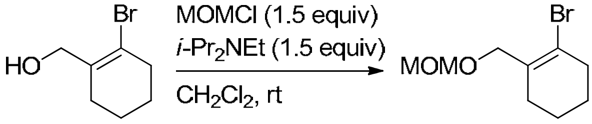

8j, 94% yield

A solution of 2-bromo-1-cyclohexenemethanol (955 mg, 5.0 mmol) and methoxymethyl chloride (80% purity, 755 mg, 7.5 mmol) in CH_2Cl_2 (20 mL) was cooled to 0 °C. Diisopropylethyl amine (1.31 mL, 7.5 mmol) was added dropwise to the mixture. The resultant solution was allowed to warm to ambient temperature and stirred for 13 h. The progress of the reaction was confirmed by TLC. The reaction mixture was quenched with water, extracted three times with CH_2Cl_2. Then, the combined organic layer was dried over $MgSO_4$ followed by filtration and evaporation. The resultant crude mixture was purified by silica-gel column chromatography with 0−10% hexane/EtOAc eluents. The product was further purified by Kugelrohr distillation under a reduced pressure to give alkenyl bromide **8j** in 94% yield.

^{1}H NMR (392 MHz, $CDCl_3$, δ): 1.64−1.76 (m, 4H), 2.18−2.27 (m, 2H), 2.49−2.57 (m, 2H), 3.40 (s, 3H), 4.19 (s, 2H), 4.64 (s, 2H). ^{13}C NMR (99 MHz, $CDCl_3$, δ): 22.1 (CH_2), 24.6 (CH_2), 29.2 (CH_2), 36.7 (CH_2), 55.3 (CH_3), 70.5 (CH_2), 96.0 (CH_2), 122.4 (*C*), 132.7 (*C*). HRMS-EI (*m*/*z*): $[M]^+$ calcd for $C_9H_{15}BrO_2$, 234.02554; found, 234.02495.

Synthesis of (*Z*)-8k.

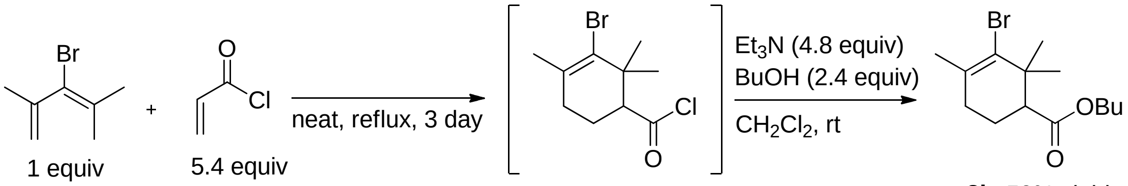

8k, 50% yield

3-Bromo-2,4-dimethyl-penta-1,3-diene (926 mg, 5.0 mmol) and acryloyl chloride (2.43 g, 26.8 mmol) were added to an oven-dried 20 mL-Schlenk flask under nitrogen. The reaction mixture was stirred and refluxed for 3 days. The consumption of the alkenyl bromide was checked by GC

analysis. After 3 days, the residual acryloyl chloride was removed from the reaction mixture under a reduced pressure. CH_2Cl_2 (4 mL) and Et_3N (2.42g, 24 mmol) were added to the resulting mixture, then the solution was cooled to 0 °C. BuOH was added dropwise to the reaction mixture and the resultant solution was allowed to warm to room temperature. After stirred for 5 h, the reaction mixture was passed through a thin-pad of silica-gel, and the resulting solution was concentrated by evaporation. The crude product was purified by silica-gel column chromatography with 0−1% hexane/Et_2O eluent, then further purified by Kugelrohr distillation under a reduced pressure to give alkenyl bromide **8k** in 50% yield.

^{1}H NMR (396 MHz, $CDCl_3$, δ): 0.94 (t, *J* = 7.4 Hz, 3H), 1.12 (s, 3H), 1.31 (s, 3H), 1.34−1.45 (m, 2H), 1.58−1.67 (m, 2H), 1.82 (s, 3H), 1.75−1.94 (m, 2H), 2.13 (dd, *J* = 5.0, 7.7 Hz, 2H), 2.55 (dd, *J* = 3.2, 11.8 Hz, 1H), 4.06 (dt, *J* = 6.6, 10.8 Hz, 1H), 4.12 (dt, *J* = 6.4, 10.8 Hz, 1H). ^{13}C NMR (100 MHz, $CDCl_3$, δ): 13.7 (CH_3), 19.2 (CH_2), 22.0 (CH_2), 23.4 (CH_3), 24.6 (CH_3), 29.4 (CH_3), 30.7 (CH_2), 32.2 (CH_2), 40.7 (*C*), 51.8 (*C*H), 64.2 (CH_2), 130.9 (*C*), 131.9 (*C*), 173.8 (*C*). HRMS-ESI (*m*/*z*): $[M+Na]^+$ calcd for $C_{14}H_{23}BrO_2Na$, 325.07736; found, 325.07775.

4. Characterization of Boryl Substitution Products

4,4,5,5-Tetramethyl-2-(4-(oxiran-2-yl)phenyl)-1,3,2-dioxaborolane (3a).

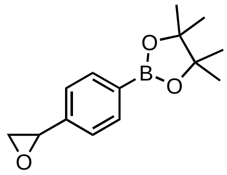

The reaction was performed according to the typical procedure **A** with **2a** (100.5 mg, 0.505 mmol). After the borylation reaction completed, the reaction mixture was directly filtered through a short silica-gel column with AcOEt as an eluent. After removal of the solvents under a reduced pressure, the crude product was purified by silica-gel column chromatography (treated with 3% Et_3N, hexane/EtOAc = 0/50 to 8/50). Then, a volatile byproduct, dimethyl(phenyl)silanol, was removed by keeping the sample under a reduced pressure (approx. 70 Pa) at 30 ºC for several hours to give **3a** in 49% isolated yield [61.3 mg, 0.249 mmol, (84% NMR yield in the crude mixture)]. This product contains a small amount of impurity.

^{1}H NMR (392 MHz, $CDCl_3$, δ): 1.34 (s, 12H), 2.78 (dd, *J* =2.7, 5.6 Hz, 1H), 3.15 (dd, *J* = 4.1, 5.6 Hz, 1H), 3.86 (dd, *J* = 2.9, 3.9 Hz, 1H), 7.28 (d, *J* = 7.9 Hz, 2H), 7.79 (d, *J* = 8.2 Hz, 2H). ^{13}C NMR (99 MHz, $CDCl_3$, δ): 24.8 (CH_3), 51.3 (CH_2), 52.3 (*C*H), 83.8 (*C*), 124.7 (*C*H), 134.9 (*C*H), 140.7 (*C*). The carbon directly attached to the boron atom was not detected, likely due to quadropolar

relaxation. HRMS-EI (*m*/*z*): $[M+H]^+$ calcd for $C_{14}H_{19}O_3{}^{11}B$, 246.14298; found, 246.14331.

4,4,5,5-Tetramethyl-2-(4-(methylthio)phenyl)-1,3,2-dioxaborolane (3b).

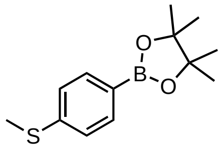

The reaction was performed according to the typical procedure **A** with **2b** (102.1 mg, 0.503 mmol). After the borylation reaction completed, the reaction mixture was cooled to −7 ºC followed by addition of TBAF (0.5 M THF solution, 1.6 mL) to remove unreacted (dimethylphenylsilyl)boronic acid pinacol ester and a byproduct, methoxydimethylphenylsilane. The title compound was purified by silica-gel column chromatography (Et_2O/hexane = 0/50 to 3/47) to give **3b** in 63% isolated yield [79.2 mg, 0.317 mmol, (78% NMR yield in the crude mixture)].

^{1}H and ^{13}C NMR spectra were in agreement with the literature.[19] ^{1}H NMR (392 MHz, $CDCl_3$, δ): 1.33 (s, 12H), 2.48 (s, 3H), 7.22 (d, *J* = 8.2 Hz, 2H), 7.71 (d, *J* = 7.9 Hz, 2H). ^{13}C NMR (99 MHz, $CDCl_3$, δ): 14.9 (*C*H_3), 24.8 (*C*H_3), 83.6 (*C*), 124.9 (*C*H), 135.0 (*C*H) 142.5 (*C*). The carbon directly attached to the boron atom was not detected, likely due to quadropolar relaxation. HRMS-EI (*m*/*z*): $[M]^+$ calcd for $C_{13}H_{19}{}^{11}BO_2S$, 250.12013; found, 250.11939.

4,4,5,5-Tetramethyl-2-(4-vinylphenyl)-1,3,2-dioxaborolane (3c).

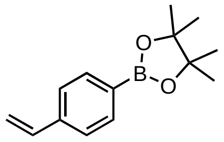

The reaction was performed according to the typical procedure **A** with **2c** (0.502 mmol, 92.0 mg). After the borylation reaction completed, the reaction mixture was cooled to −7 ºC followed by addition of TBAF (0.5 M THF solution, 1.6 mL) to remove unreacted (dimethylphenylsilyl)boronic acid pinacol ester and a byproduct, methoxydimethylphenylsilane. The title compound was purified by silica-gel column chromatography (Et_2O/hexane = 0/50 to1.5/50) to give **3c** in 64% isolated yield [73.5 mg, 0.32 mmol, (85% NMR yield in the crude mixture)].

^{1}H and ^{13}C NMR spectra were in agreement with the literature.[20] ^{1}H NMR (392 MHz, $CDCl_3$, δ): 1.33 (s, 12H), 5.28 (dd, *J* = 0.7, 10.8 Hz, 1H), 5.80 (dd, *J* = 0.7, 17.6 Hz, 1H), 6.72 (dd, *J* = 10.9, 17.8 Hz, 1H), 7.40 (d, *J* = 8.3 Hz, 2H), 7.77 (d, *J* = 8.2 Hz, 2H). ^{13}C NMR (99 MHz, $CDCl_3$, δ): 24.8 (*C*H_3), 83.7 (*C*), 114.8 (*C*H_2), 125.5 (*C*H), 135.0 (*C*H), 136.8 (*C*H), 140.1 (*C*). The carbon directly

attached to the boron atom was not detected, likely due to quadropolar relaxation. HRMS-EI (*m*/*z*): $[M]^+$ calcd for $C_{14}H_{19}{}^{10}BO_2$, 229.15144; found, 229.15054.

(*E*)-4,4,5,5-Tetramethyl-2-(4-styrylphenyl)-1,3,2-dioxaborolane (3d).

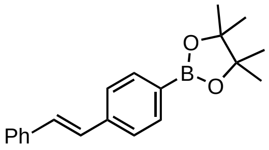

The reaction was performed according to the typical procedure **A** with **2d** (129.6 mg, 0.500 mmol). The title compound was purified by silica-gel column chromatography (Et_2O/hexane = 0/50 to 2.5/50) to give **3d** in 62% isolated yield [95.3 mg, 0.311 mmol, (87% NMR yield in the crude mixture)].

^1H and ^{13}C NMR spectra were in agreement with the literature.[21] ^1H NMR (392 MHz, $CDCl_3$, δ): 1.35 (s, 12H), 7.07−7.20 (m, 2H), 7.21−7.28 (m, 1H), 7.35 (t, *J* = 7.5 Hz, 2H), 7.51 (d, *J* = 7.9 Hz, 4H), 7.80 (d, *J* = 8.3 Hz, 2H). ^{13}C NMR (99 MHz, $CDCl_3$, δ): 24.8 (CH_3), 83.7 (*C*), 125.8 (*C*H), 126.6 (*C*H), 127.8 (*C*H), 128.6 (*C*H), 128.7 (*C*H), 129.6 (*C*H), 135.1 (*C*H), 137.1 (*C*), 140.0 (*C*). The carbon directly attached to the boron atom was not detected, likely due to quadropolar relaxation. HRMS-EI (*m*/*z*): $[M]^+$ calcd for $C_{20}H_{23}{}^{10}BO_2$, 305.18274; found, 305.18300.

4-(4-Nitrophenyl)dibenzo[*b*,*d*]furan (4h).

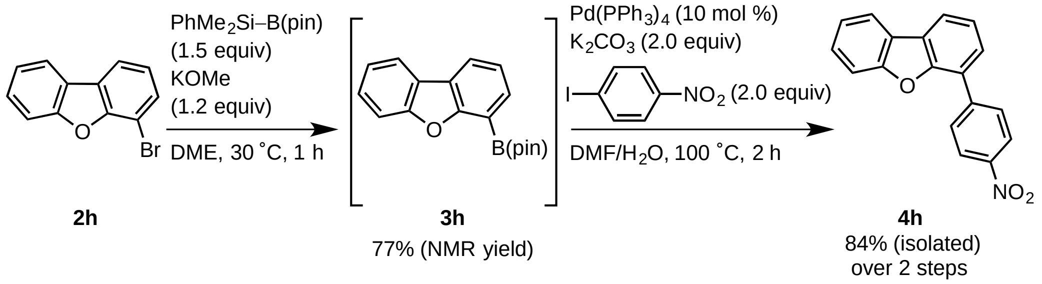

The reaction was performed according to the typical procedure **B** with **2h** (124.0 mg, 0.502 mmol). After the borylation reaction completed, the reaction mixture was cooled to −7 ºC followed by addition of TBAF (0.5 M THF solution, 1.6 mL) to remove unreacted (dimethylphenylsilyl)boronic acid pinacol ester and a byproduct, methoxydimethylphenylsilane. The resultant solution was stirred for 2 h at same temperature. After that, H_2O was added to the mixture, then extracted three times with Et_2O. The resultant reaction mixture was transferred to a 20 mL-Schlenk flask with a magnetic stirrer bar. Then DMF (4 mL), N_2-bubbled H_2O (0.4 mL), K_2CO_3 (138.8 mg, 1.00 mmol), 1-iodo-4-nitrobenzene (249.5 mg, 1.00 mmol) and $Pd(PPh_3)_4$ (58.0 mg, 0.0502 mmol) were

successively added to the flask. The solution was heated to 100 ºC and stirred for 2 h. After that, H_2O was added to the mixture and extracted three times with AcOEt. The title compound was purified by silica-gel column chromatography (AcOEt/hexane = 0/100 to 50/50) to give **4h** in 84% isolated yield over two steps [122.1 mg, 0.422 mmol, (77% NMR yield of **3h** in the crude mixture)].

^{1}H and ^{13}C NMR spectra were in agreement with the literature.[22] ^{1}H NMR (392 MHz, $CDCl_3$, δ): 7.37−7.44 (m, 1H), 7.46−7.55 (m, 2H), 7.60−7.68 (m, 2H), 7.95−8.05 (m, 2H), 8.09−8.15 (m, 2H), 8.37−8.43 (m, 2H). ^{13}C NMR (99 MHz, $CDCl_3$, δ): 111.8 (*C*H), 120.8 (*C*H), 121.4 (*C*H), 123.2 (*C*H), 123.3 (*C*), 123.4 (*C*H), 123.8 (*C*), 123.9 (*C*H), 125.4 (*C*), 126.8 (*C*H), 127.7 (*C*H), 129.4 (*C*H), 143.0 (*C*), 147.1 (*C*), 153.2 (*C*), 156.1 (*C*). HRMS-EI (*m*/*z*): $[M]^+$ calcd for $C_{18}H_{11}O_3N$, 289.07389; found, 289.07343.

9-Ethyl-3-(4,4,5,5-tetramethyl-1,3,2-dioxaborolan-2-yl)-9*H*-carbazole (3i).

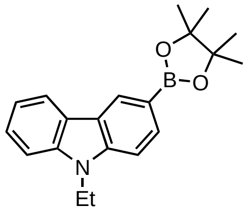

The reaction was performed according to the typical procedure **A** with **2i** (137.1 mg, 0.500 mmol). After the borylation reaction completed, the reaction mixture was filtrated to remove insoluble material that was formed during the reaction. The resultant solution was evaporated followed by the purification of silica-gel column chromatography (Et_2O/hexane = 0/50 to 5/50) to give **3i** in 74% isolated yield [119.4 mg, 0.372 mmol, (85% NMR yield in the crude mixture)].

^{1}H NMR spectrum was in agreement with the literature.[23] ^{1}H NMR (392 MHz, $CDCl_3$, δ): 1.40 (s, 12H), 1.41 (t, *J* = 7.2 Hz, 3H), 4.35 (q, *J* = 7.3 Hz, 2H), 7.24 (t, *J* = 7.4 Hz, 1H), 7.40 (d, *J* = 8.2 Hz, 2H), 7.43−7.49 (m, 1H), 7.93 (dd, *J* = 0.7, 8.3 Hz, 1H), 8.14 (d, *J* = 7.9 Hz, 1H), 8.61 (s, 1H). ^{13}C NMR (99 MHz, $CDCl_3$, δ): 13.7 (CH_3), 24.9 (CH_3), 37.5 (CH_2) 83.5 (*C*), 107.8 (*C*H), 108.4 (*C*H), 119.2 (*C*H), 120.6 (*C*H), 122.6 (*C*), 123.2 (*C*), 125.6 (*C*H), 127.8 (*C*H), 132.1 (*C*H), 140.0 (*C*), 142.0 (*C*). The carbon directly attached to the boron atom was not detected, likely due to quadropolar relaxation. HRMS-ESI (*m*/*z*): $[M+H]^+$ calcd for $C_{20}H_{25}O_2N^{10}B$, 321.20092; found, 321.20145.

2-(Dibenzo[*b*,*d*]thiophen-2-yl)-4,4,5,5-tetramethyl-1,3,2-dioxaborolane (3j).

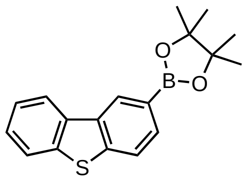

The reaction was performed according to the typical procedure **A** with **2j** (131.5 mg, 0.500 mmol). After the borylation reaction completed, the reaction mixture was cooled to −7 ºC followed by addition of TBAF (0.5 M THF solution, 1.6 mL) to remove unreacted (dimethylphenylsilyl)boronic acid pinacol ester and a byproduct, methoxydimethylphenylsilane. The title compound was purified by silica-gel column chromatography (Et_2O/hexane = 0/100 to 2.5/50) to give **3j** in 61% isolated yield [94.2 mg, 0.607 mmol, (75% NMR yield in the crude mixture)].

^{1}H NMR (392 MHz, $CDCl_3$, δ): 1.39 (s, 12H), 7.40−7.48 (m, 2H), 7.80−7.90 (m, 3H), 8.21−8.27 (m, 1H), 8.62 (s, 1H). ^{13}C NMR (99 MHz, $CDCl_3$, δ): 24.9 (*C*H_3), 83.9 (*C*), 121.8 (*C*H), 122.1 (*C*H), 122.7 (*C*H), 124.4 (*C*H), 126.7 (*C*H), 128.2 (*C*H), 132.5 (*C*H), 135.0 (*C*), 135.5 (*C*), 139.1 (*C*), 142.7 (*C*). The carbon directly attached to the boron atom was not detected, likely due to quadropolar relaxation. HRMS-EI (*m*/*z*): $[M]^+$ calcd for $C_{18}H_{19}O_2{}^{11}BS$, 310.12021; found, 310.11915.

2-(Benzo[b]thiophen-5-yl)-4,4,5,5-tetramethyl-1,3,2-dioxaborolane (3k).

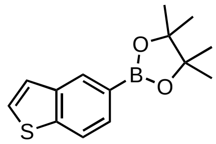

The reaction was performed according to the typical procedure **A** with **2k** (107.0 mg, 0.502 mmol). After the borylation reaction completed, the reaction mixture was diluted by EtOAc (20 mL). Then, the solution was cooled to −5 ºC followed by addition of TBAF (1.0 M THF solution, 0.5 mL) to remove unreacted (dimethylphenylsilyl)boronic acid pinacol ester and a byproduct, methoxydimethylphenylsilane. The title compound was purified by silica-gel column chromatography (Et_2O/hexane = 0/100 to 0.75/50) to give **3k** in 51% isolated yield (67.0 mg, 0.258 mmol).

^{1}H NMR spectrum was in agreement with the literature.[24] ^{1}H NMR (392 MHz, $CDCl_3$, δ): 1.37 (s, 12H), 7.34 (d, *J* = 5.0 Hz, 1H), 7.40 (d, *J* = 5.4 Hz, 1H), 7.75 (dd, *J* = 0.9, 8.1 Hz, 1H), 7.89 (d, *J* = 8.3 Hz, 1H), 8.31 (s, 1H). ^{13}C NMR (99 MHz, $CDCl_3$, δ): 24.9 (*C*H_3), 83.8 (*C*), 121.8 (*C*H), 124.1 (*C*H), 126.0 (*C*H), 129.7 (*C*H), 130.7 (*C*H), 139.1 (*C*), 142.7 (*C*). The carbon directly attached to the boron atom was not detected, likely due to quadropolar relaxation. HRMS-EI (*m*/*z*): $[M]^+$ calcd for

$C_{14}H_{17}{}^{10}BO_2S$, 259.10786; found, 259.10830.

4,4,5,5-Tetramethyl-2-(3-methylthiophen-2-yl)-1,3,2-dioxaborolane (3l).

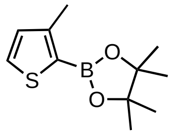

The reaction was performed according to the typical procedure **A** with **2l** (89.1 mg, 0.503 mmol). After the borylation reaction completed, the reaction mixture was cooled to −7 ºC followed by addition of TBAF (0.5 M THF solution, 2.0 mL) to remove unreacted (dimethylphenylsilyl)boronic acid pinacol ester and a byproduct, methoxydimethylphenylsilane. The title compound was purified by silica-gel column chromatography (Et_2O/hexane = 0/50 to 3/50) to give **3l** in 82% isolated yield [92.2 mg, 0.411 mmol, (87% NMR yield in the crude mixture)].

^1H NMR spectrum was in agreement with the literature.[25] ^1H NMR (392 MHz, $CDCl_3$, δ): 1.33 (s, 12H), 2.48 (s, 3H), 6.97 (d, J = 4.7 Hz, 1H), 7.47 (d, J = 4.7 Hz, 1H). ^{13}C NMR (99 MHz, $CDCl_3$, δ): 16.0 (CH$_3$) 24.8 (CH$_3$), 83.5 (C), 131.28 (CH), 131.33 (CH), 149.0 (C). The carbon directly attached to the boron atom was not detected, likely due to quadropolar relaxation. HRMS-EI (m/z): $[M]^+$ calcd for $C_{11}H_{17}{}^{10}BO_2S$, 223.10786; found, 223.10748.

1-Benzyl-4-(4,4,5,5-tetramethyl-1,3,2-dioxaborolan-2-yl)-1*H*-pyrazole (3m).

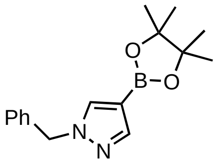

The reaction was performed according to the typical procedure **A** with **2m** (119.0 mg, 0.501 mmol). After the borylation reaction completed, the reaction mixture was filtrated to remove insoluble material that was formed during the reaction. The resultant solution was evaporated followed by the purification of silica-gel column chromatography (EtOAc/hexane = 3/100 to 7.5/50) to give **3m** in 59% isolated yield [84.2 mg, 0.296 mmol, (68% NMR yield in the crude mixture)].

^1H NMR (392 MHz, $CDCl_3$, δ): 1.30 (s, 12H), 5.30 (s, 2H), 7.24–7.34 (m, 5H), 7.67 (s, 1H), 7.82 (s, 1H). ^{13}C NMR (99 MHz, $CDCl_3$, δ): 24.7 (CH$_3$), 55.8 (CH$_2$) 83.2 (C), 127.9 (CH), 128.1 (CH), 128.8 (CH), 136.0 (C), 136.1 (CH), 145.7 (CH). The carbon directly attached to the boron atom was not detected, likely due to quadropolar relaxation. HRMS-ESI (m/z): $[M+H]^+$ calcd for $C_{16}H_{22}O_2N_2{}^{10}B$, 284.18052; found, 284.18133.

1,3,5-Trimethyl-4-(4,4,5,5-tetramethyl-1,3,2-dioxaborolan-2-yl)-1*H*-pyrazole (3n).

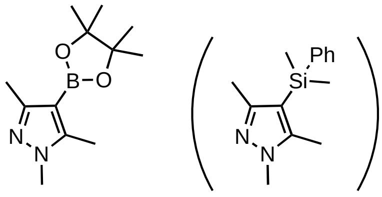

The reaction was performed according to the typical procedure **A** with **2n** (95.4 mg, 0.504 mmol). After the borylation reaction completed, the reaction mixture was filtrated to remove insoluble material that formed during the reaction. The resultant solution was evaporated followed by the purification of silica-gel column chromatography (EtOAc/hexane = 0/50 to 8/50) to give the inseparable mixture of **3n** and silyl substituted product **3n'** in 82% yield and a B/Si ratio of 81:19 (97.9 mg, 0.411 mmol, the yield of borylated product **3n**: 67%). The B/Si ratio was determined by ^{1}H NMR spectrum.

^{1}H NMR spectrum of **3n** was in agreement with the literature.[26] ^{1}H NMR spectrum of the minor silyl substituted product **3n'** was in agreement with the literature.[27] ^{1}H NMR (392 MHz, $CDCl_3$, δ): 1.29 (s, 12H), 2.33 (s, 3H), 2.37 (s, 3H), 3.69 (s, 3H). ^{13}C NMR (99 MHz, $CDCl_3$, δ): 11.2 (CH_3), 13.7 (CH_3), 24.8 (CH_3), 35.2 (CH_3), 82.3 (*C*), 127.7 (*C*), 133.7 (*C*). The carbon directly attached to the boron atom was not detected, likely due to quadropolar relaxation. HRMS-ESI (*m*/*z*): $[M+H]^+$ calcd for $C_{12}H_{22}O_2N_2{}^{10}B$, 236.18052; found, 236.18085.

3,5-Dimethyl-4-(4,4,5,5-tetramethyl-1,3,2-dioxaborolan-2-yl)isoxazole (3o).

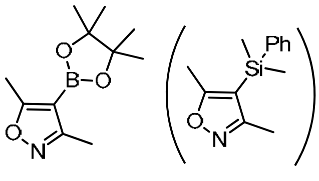

The reaction was performed according to the typical procedure **A** with **2o** (88.1 mg, 0.501 mmol). After the borylation reaction completed, the reaction mixture was filtrated to remove insoluble material that was formed during the reaction. The resultant solution was evaporated followed by the purification of silica-gel column chromatography (Et_2O/hexane = 0/50 to 2.5/50) to give the inseparable mixture of **3o** and silyl substituted product **3o'** in 57% yield and a B/Si ratio of 90:10

[64.0 mg, 0.286 mmol, the yield of borylated product **3o**: 51%, (NMR yield of **3o** in the crude mixture: 74%)]. The B/Si ratio was determined by ^{1}H NMR spectrum.

^{1}H and ^{13}C NMR spectra of **3o** were in agreement with the literature.[28] ^{1}H NMR spectrum of the minor silyl substituted product **3o'** was in agreement with the literature.[27] ^{1}H NMR (392 MHz, $CDCl_3$, δ): 1.30 (s, 12H), 2.33 (s, 3H), 2.51 (s, 3H). ^{13}C NMR (99 MHz, $CDCl_3$, δ): 11.7 (CH_3), 12.8 (CH_3), 24.8 (CH_3), 83.2 (*C*), 163.8 (*C*), 177.9 (*C*). The carbon directly attached to the boron atom was not detected, likely due to quadropolar relaxation. HRMS-ESI (*m*/*z*): $[M+H]^+$ calcd for $C_{11}H_{19}O_3N^{10}B$, 223.14888; found, 223.14951.

2,4-Diphenyl-5-(4,4,5,5-tetramethyl-1,3,2-dioxaborolan-2-yl)oxazole (3p).

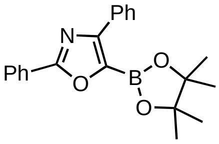

The reaction was performed according to the typical procedure **A** with **2p** (150.2 mg, 0.500 mmol), (dimethylphenylsilyl)boronic acid pinacol ester (3.0 equiv, 391.3 mg, 1.49 mmol) and potassium methoxide (2.4 equiv, 84.2 mg, 1.20 mmol). After the borylation reaction completed, H_2O was added to the mixture, then extracted three times with Et_2O. The organic layer was washed with water. The combined organic layer was then dried over $MgSO_4$ followed by filtration and evaporation. The crude product was purified by silica-gel column chromatography (EtOAc/hexane = 0/50 to 4/46). A volatile byproduct, dimethyl(phenyl)silanol, was removed by keeping the sample under a reduced pressure (4.4×10^{-1} hPa) at 40 ºC for 3 h to give **3p** in 58% isolated yield [101.0 mg, 0.291 mmol, (63% NMR yield in the crude mixture)].

^{1}H NMR (392 MHz, $CDCl_3$, δ): 1.40 (s, 12H), 7.34−7.40 (m, 1H), 7.41−7.49 (m, 5H), 8.15−8.19 (m, 2H), 8.20−8.26 (m, 2H). ^{13}C NMR (99 MHz, $CDCl_3$, δ): 24.8 (CH_3), 84.6 (*C*), 127.3 (*C*H), 128.2 (*C*H), 128.3 (*C*H), 128.6 (*C*H), 130.7 (*C*H), 131.8 (*C*), 153.5 (C), 164.6 (C). The carbon directly attached to the boron atom was not detected, likely due to quadropolar relaxation. HRMS-ESI (*m*/*z*): $[M+H]^+$ calcd for $C_{21}H_{23}O_3N^{10}B$, 347.18018; found, 347.18061.

2-Ethyl-4-methyl-5-(4,4,5,5-tetramethyl-1,3,2-dioxaborolan-2-yl)thiazole (3q).

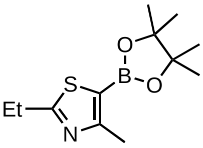

The reaction was performed according to the typical procedure **A** with **2q** (102.5 mg, 0.497

mmol). After the borylation reaction completed, the reaction mixture was filtrated to remove insoluble material that was formed during the reaction. The resultant solution was evaporated followed by the purification of silica-gel column chromatography (AcOEt/hexane = 10/90 to 20/30). A volatile byproduct, dimethyl(phenyl)silanol, was removed by keeping the sample under a reduced pressure (approx. 70 Pa) at 30 ºC for several hours to give **3q** in 46% isolated yield [58.2 mg, 0.229 mmol, (68% NMR yield)].

^{1}H NMR (392 MHz, $CDCl_3$, δ): 1.33 (s, 12H), 1.37 (t, *J* = 7.5 Hz, 3H), 2.60 (s, 3H), 3.01 (q, *J* = 7.7 Hz, 2H). ^{13}C NMR (99 MHz, $CDCl_3$, δ): 14.2 (CH_3), 17.5 (CH_3), 24.8 (CH_3), 26.8 (CH_2), 83.9 (*C*), 162.9 (*C*), 177.3 (*C*). The carbon directly attached to the boron atom was not detected, likely due to quadropolar relaxation. HRMS-ESI (*m*/*z*): $[M+H]^+$ calcd for $C_{12}H_{21}O_2N^{10}BS$, 253.14169; found, 253.14184.

5-Phenyl-2-(piperidin-1-yl)pyrimidine (4t).

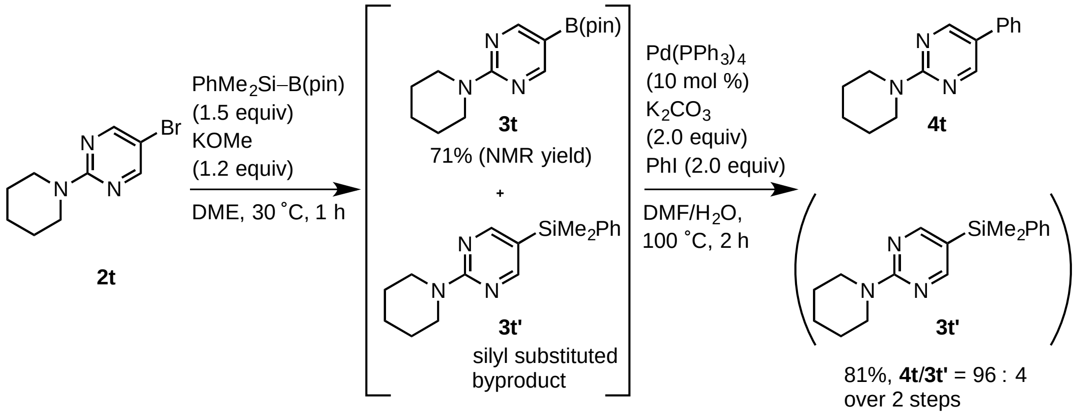

The reaction was performed according to the typical procedure **B** with **2t** (121.5 mg, 0.502 mmol). After the borylation reaction completed, the reaction mixture was cooled to −7 ºC followed by addition of TBAF (0.5 M THF solution, 1.6 mL) to remove unreacted (dimethylphenylsilyl)boronic acid pinacol ester and a byproduct, methoxydimethylphenylsilane. After that, H_2O was added to the mixture, then extracted three times with Et_2O. The resultant reaction mixture was transferred to a 20 mL-Schlenk flask with a magnetic stirrer bar. Then, DMF (4 mL), N_2-bubbled H_2O (0.4 mL), K_2CO_3 (138.2 mg, 1.00 mmol), iodobenzene (205.7 mg, 1.01 mmol) and $Pd(PPh_3)_4$ (57.8 mg, 0.0500 mmol) were successively added to the flask. The solution was heated to 100 ºC and stirred for 2 h. After that, H_2O was added to the mixture and extracted three times with Et_2O. The title compound was purified by silica-gel column chromatography (EtOAc/hexane = 0/100 to 8/92) to give inseparable mixture of **4t** and silyl substituted product **3t'** in 81% yield [98.4 mg, 0.407 mmol, **4t**/**3t'** = 96:4, the yield of **4t**: 78%, (NMR yield of **3t** in the crude mixture: 71%)]. The B/Si (**4t**/**3t'**) ratio was determined by ^{1}H

NMR spectrum.

^{1}H NMR (392 MHz, $CDCl_3$, δ): 1.58−1.75 (m, 6H), 3.81−3.87 (m, 4H), 7.29−7.35 (m, 1H), 7.40−7.49 (m, 4H), 8.54 (s, 2H). ^{13}C NMR (99 MHz, $CDCl_3$, δ): 24.8 (CH$_2$), 25.7 (CH$_2$), 44.9 (CH$_2$), 122.0 (C), 125.7 (CH), 127.0 (CH), 129.0 (CH), 135.8 (C), 155.8 (CH), 160.9 (C). HRMS-ESI (m/z): $[M+H]^+$ calcd for $C_{15}H_{18}N_3$, 240.14952; found, 240.14974.

3-(4-Methoxyphenyl)pyridine (4u).

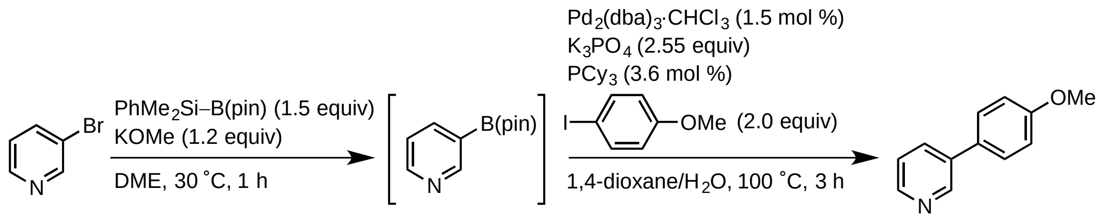

2u **3u** 68% (NMR yield) **4u** 64% (isolated) over 2 steps

This reaction was carried out in a 20 mL-Schlenk flask. The reaction was performed according to the typical procedure **B** with **2u** (79.2 mg, 0.501 mmol). After the borylation reaction completed, the reaction mixture was cooled to −7 ºC followed by addition of TBAF (0.5 M THF solution, 1.8 mL) to remove unreacted (dimethylphenylsilyl)boronic acid pinacol ester and a byproduct, methoxydimethylphenylsilane. After that, the solvent was removed in vacuum. Then, 1,4-dioxane (3 mL), N_2-bubbled H_2O (1 mL), K_3PO_4 (271.0 mg, 1.28 mmol), 4-iodoanisole (175.8 mg, 0.75 mmol), $Pd_2(dba)_3 \cdot CHCl_3$ (7.7 mg, 0.0074 mmol) and PCy_3 (5.1 mg, 0.018 mmol) were successively added to the flask. The solution was heated to 100 ºC and stirred for 3 h. After that, H_2O was added to the mixture and extracted three times with AcOEt. The title compound was purified by silica-gel column chromatography with 15% hexane/AcOEt eluent. A volatile byproduct, dimethyl(phenyl)silanol, was removed by keeping the sample under a reduced pressure (approx. 70 Pa) at 40 ºC for several hours to give **4u** in 64% isolated yield over two steps [59.5 mg, 0.321 mmol, (NMR yield of **3u** in the crude mixture: 68%)].

^{1}H and ^{13}C NMR spectra were in agreement with the literature.[29] ^{1}H NMR (396 MHz, $CDCl_3$, δ): 3.86 (s, 3H), 6.98−7.04 (m, 2H), 7.30−7.35 (m, 1H), 7.49−7.55 (m, 2H), 7.80−7.84 (m, 1H), 8.54 (dd, J = 1.6, 4.8 Hz, 1H), 8.82 (d, J = 2.3 Hz, 1H). ^{13}C NMR (100 MHz, $CDCl_3$, δ): 55.3 (CH$_3$), 114.5 (CH), 123.4 (CH), 128.2 (CH), 130.2 (C), 133.8 (CH), 136.2 (C), 147.8 (CH), 148.0 (CH), 159.7 (C). HRMS-EI (m/z): $[M]^+$ calcd for $C_{12}H_{11}ON$, 185.08406; found, 185.08387.

3-(4-Methoxyphenyl)quinoline (4v).

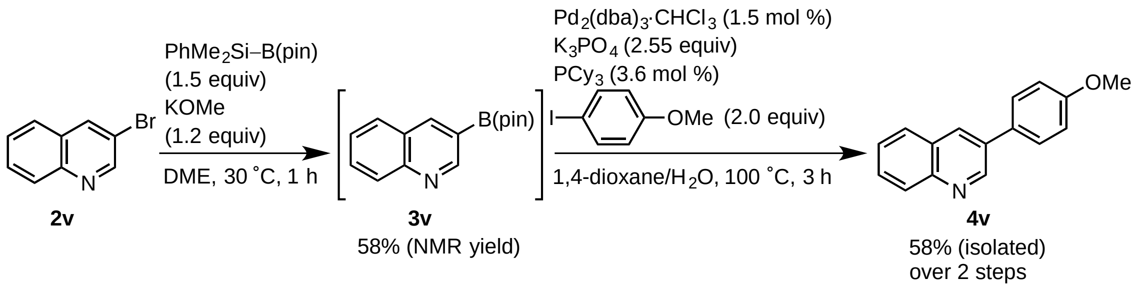

This reaction was carried out in a 20 mL-Schlenk flask. The reaction was performed according to the typical procedure **B** with **2v** (104.2 mg, 0.501 mmol). After the borylation reaction completed, the reaction mixture was cooled to −7 ºC followed by addition of TBAF (0.5 M THF solution, 1.8 mL) to remove unreacted (dimethylphenylsilyl)boronic acid pinacol ester and a byproduct, methoxydimethylphenylsilane. After that, the solvent was removed in vacuum, then 1,4-dioxane (3 mL), N_2-bubbled H_2O (1 mL), K_3PO_4 (271.2 mg, 1.28 mmol), 4-iodoanisole (175.6 mg, 0.75 mmol), $Pd_2(dba)_3\cdot CHCl_3$ (7.8 mg, 0.0075 mmol) and PCy_3 (5.1 mg, 0.018 mmol) were successively added to the flask. The solution was heated to 100 ºC and stirred for 2 h. After that, H_2O was added to the mixture and extracted three times with AcOEt. The title compound was purified by silica-gel column chromatography with 10% hexane/AcOEt eluent, then purified by GPC to give **4v** in 58% isolated yield over two steps [68.0 mg, 0.289 mmol, (NMR yield of **3v** in the crude mixture: 58%)].

^{1}H and ^{13}C NMR spectra were in agreement with the literature.[29] ^{1}H NMR (392 MHz, $CDCl_3$, δ): 3.87 (s, 3H), 7.05 (d, *J* = 7.5 Hz, 2H), 7.55−7.73 (m, 4H), 7.86 (d, *J* = 7.9 Hz, 1H), 8.17 (d, *J* = 8.2 Hz, 1H), 8.27 (s, 1H), 9.16 (s, 1H). ^{13}C NMR (99 MHz, $CDCl_3$, δ): 55.4 (CH_3), 114.6 (*C*H), 127.1 (*C*H), 127.8 (*C*H), 128.1 (*C*), 128.4 (*C*H), 128.6 (*C*H), 129.3 (*C*H), 129.9 (*C*), 132.9 (*C*H), 133.5 (*C*), 146.2 (*C*), 149.3 (*C*H), 159.8 (*C*). HRMS-EI (*m*/*z*): $[M]^+$ calcd for $C_{16}H_{13}ON$, 235.09971; found, 235.09956.

3-Methyl-4-(4-nitrophenyl)thiophene (4w).

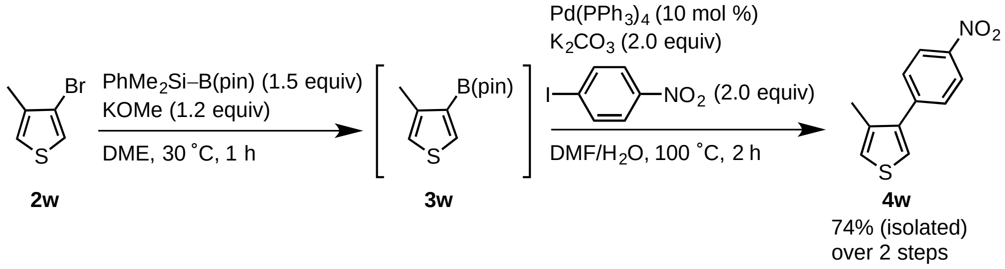

The reaction was performed according to the typical procedure **B** with **2w** (88.6 mg, 0.500 mmol). After the borylation reaction completed, the reaction mixture was cooled to −7 ºC followed by addition of TBAF (0.5 M THF solution, 1.6 mL) to remove unreacted (dimethylphenylsilyl)boronic acid pinacol ester and a byproduct, methoxydimethylphenylsilane. The resultant solution was stirred for 2 h at the same temperature. After that, H_2O was added to the mixture, then extracted three times with AcOEt. The resultant reaction mixture was transferred to a 20 mL-Schlenk flask with a magnetic stirrer bar. Then DMF (4 mL), N_2-bubbled H_2O (0.4 mL), K_2CO_3 (138.6 mg, 1.00 mmol), 1-iodo-4-nitrobenzene (249.5 mg, 1.00 mmol) and $Pd(PPh_3)_4$ (57.8 mg, 0.0500 mmol) were successively added to the flask. The solution was heated to 100 ºC and stirred for 2 h. After that, H_2O was added to the mixture and extracted three times with AcOEt. The title compound was purified by silica-gel column chromatography with hexane eluent to give **4w** in 74% isolated yield over two steps (81.6 mg, 0.372 mmol).

^{1}H NMR (392 MHz, $CDCl_3$, δ): 2.30 (s, 3H), 7.07–7.11 (m, 1H), 7.32 (d, *J* = 3.2 Hz, 1H), 7.53–7.58 (m, 2H), 8.24–8.29 (m, 2H). ^{13}C NMR (99 MHz, $CDCl_3$, δ): 15.5 (CH$_3$), 123.2 (CH), 123.7 (CH), 124.8 (CH), 129.1 (CH), 135.6 (C), 140.7 (C), 143.6 (C), 146.7 (C). HRMS-EI (*m*/*z*): $[M]^+$ calcd for $C_{11}H_9O_2NS$, 219.03540; found, 219.03504.

4'-Nitro-(1,1'-biphenyl)-4-carbonitrile (4x).

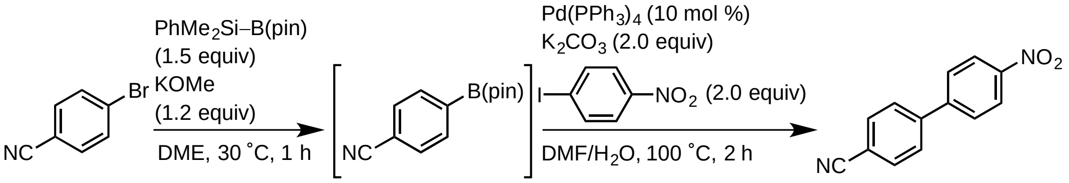

2x 3x 4x

36% (isolated) over 2 steps

The reaction was performed according to the typical procedure **B** with **2x** (91.2 mg, 0.501 mmol). After the borylation reaction completed, the reaction mixture was cooled to −7 ºC followed by addition of TBAF (0.5 M THF solution, 1.6 mL) to remove unreacted (dimethylphenylsilyl)boronic acid pinacol ester and a byproduct, methoxydimethylphenylsilane. The resultant solution was stirred for 2 h at the same temperature. After that, H_2O was added to the mixture, then extracted three times with Et_2O. The resultant reaction mixture was transferred to a 20 mL-Schlenk flask with a magnetic stirrer bar. Then, DMF (4 mL), N_2-bubbled H_2O (0.4 mL), K_2CO_3 (140.0 mg, 1.01 mmol), 1-iodo-4-nitrobenzene (250.8 mg, 1.01 mmol) and $Pd(PPh_3)_4$ (58.0 mg, 0.0502 mmol) were successively added to the flask. The solution was heated to 100 ºC and stirred for 2 h. After that, H_2O was added to the mixture and extracted three times with AcOEt. The title compound was

purified by silica-gel column chromatography (AcOEt/hexane = 0/200 to 40/60, then 10% eluent) to give **4x** in 36% isolated yield over two steps (40.8 mg, 0.182mmol).

^{1}H and ^{13}C NMR spectra were in agreement with the literature.[30] ^{1}H NMR (392 MHz, $CDCl_3$, δ): 7.71−7.83 (m, 6H), 8.32−8.39 (m, 2H). ^{13}C NMR (99 MHz, $CDCl_3$, δ): 112.6 (*C*), 118.4 (*C*), 124.4 (*C*H), 128.08 (*C*H), 128.14 (*C*H), 132.9 (*C*), 143.1 (*C*), 145.4 (*C*), 147.9 (*C*). HRMS-EI (*m*/*z*): $[M]^+$ calcd for $C_{13}H_8O_2N_2$, 224.05858 found, 224.05776.

***tert*-Butyl 4-(4-hydroxy-1*H*-pyrazol-1-yl)piperidine-1-carboxylate (12).**

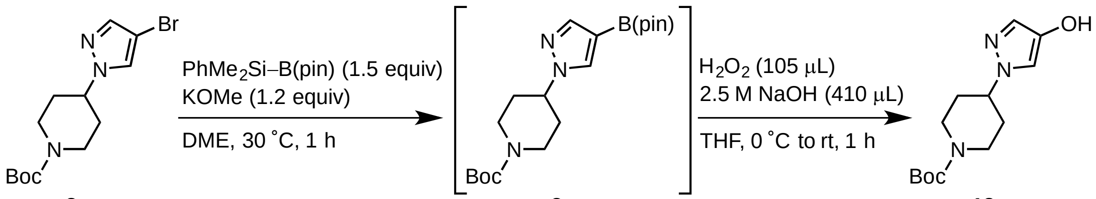

2y **3y** 51% (NMR yield) **12** 61% (isolated) over 2 steps

Potassium methoxide (0.60 mmol, 42.0 mg) and aryl halide **2y** (164.9 mg, 0.499 mmol) were added to a vial sealed with a screw cap containing a silicon-coated rubber septum in a glove box under argon atmosphere. After the reaction vial was removed from the glove box, DME (5 mL) and (dimethylphenylsilyl)boronic acid pinacol ester (0.751 mmol, 196.9 mg) were added to the vial, then stirred at 30 ºC. After 1 h, the reaction mixture was analyzed by GC to check the reaction. After the borylation reaction completed, the solvent was removed by evaporation. Then, THF (1.2 mL), HCl aqueous solution (2.5 M, 406 µL) and H_2O_2 (34.2 mg, 1.0 mmol) were successively added at 0 ºC, then stirred at room temperature for 45 min. After that, pH of the aqueous phase was adjusted to 2.0 by addition of 2.0 M aqueous HCl solution. The mixture was then extracted three times with CH_2Cl_2. The combined organic layer was dried over $MgSO_4$ followed by filtration and evaporation. The crude product was purified by silica-gel column chromatography with 50% hexane/AcOEt eluent to give **12** in 61% isolated yield over two steps [81.3 mg, 0.304 mmol, (NMR yield of **3y** in the crude mixture: 51%)].

^{1}H NMR (392 MHz, $CDCl_3$, δ): 1.47 (s, 9H), 1.78 (dq, J = 4.3, 12.3 Hz, 2H), 2.04 (d, J = 10.4 Hz, 2H), 2.70−2.95 (m, 2H) 4.06−4.30 (m, 3H), 7.08 (s, 1H), 7.15 (s, 1H), 8.04 (brs, 1H). ^{13}C NMR (99 MHz, $CDCl_3$, δ): 28.3 (CH_3), 32.2 (CH_2), 42.9 (CH_2), 59.4 (*C*H), 80.2 (*C*), 113.8 (*C*H), 127.6 (*C*H), 141.5 (*C*), 154.7 (*C*). HRMS-ESI (*m*/*z*): $[M\text{-}H]^+$ calcd for $C_{13}H_{20}O_3N_3$, 266.15101; found, 266.15122.

tert-Butyl 4-(5-hydroxypyrimidin-2-yl)piperazine-1-carboxylate (5z).

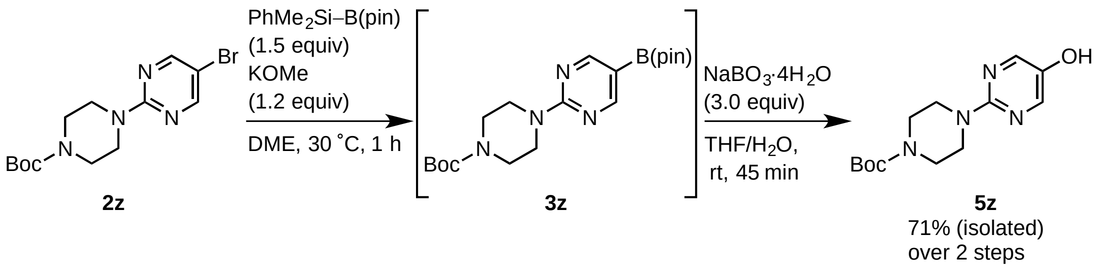

Potassium methoxide (0.60 mmol, 42.1 mg) and *tert*-butyl-4-(5-bromopyrimidin-2-yl)-piperazine-1-carboxylate **2z** (172.2 mg, 0.502 mmol) were added to a vial sealed with a screw cap containing a silicon-coated rubber septum in a glove box under argon atmosphere. After the reaction vial was removed from the glove box, DME (5 mL) and (dimethylphenylsilyl)boronic acid pinacol ester (0.753 mmol, 197.6 mg) were added to the vial, then stirred at 30 ºC. After 1 h, the reaction mixture was analyzed by GC to check the reaction. After the borylation reaction completed, the mixture was transferred to a 20 mL-round-bottomed flask with a magnetic stirrer bar and the solvent was removed by evaporation. Then, THF (4 mL), H_2O (4 mL) and $NaBO_3 \cdot 4H_2O$ (230.6 mg, 1.50 mmol) were added and stirred at room temperature. After 45 min, the reaction mixture was analyzed by GC to check the reaction. After that, the resultant solution was added to a saturated NH_4Cl aqueous solution and extracted three times with EtOAc. The combined organic layer was dried over $MgSO_4$ followed by filtration and evaporation. The crude product was purified by silica-gel column chromatography with 20% hexane/AcOEt eluent to give **5z** in 71% isolated yield over two steps (100.1 mg, 0.357 mmol).

^{1}H and ^{13}C NMR spectra were in agreement with the literature.[31] ^{1}H NMR (392 MHz, $CDCl_3$, δ): 1.49 (s, 9H), 1.65 (brs, 1H), 3.42–3.52 (m, 4H), 3.62–3.72 (m, 4H), 8.08 (s, 2H). ^{13}C NMR (100 MHz, $CDCl_3$, δ): 28.5 (CH_3), 43.6 (CH_2), 44.6 (CH_2), 80.5 (*C*), 143.5 (*C*), 146.0 (*C*H), 155.3 (*C*), 157.5 (*C*). HRMS-ESI (*m*/*z*): $[M+H]^+$ calcd for $C_{13}H_{19}O_3N_4$, 279.14626; found, 279.14660.

2-[Dimethyl(phenyl)silyl]quinoline (7).

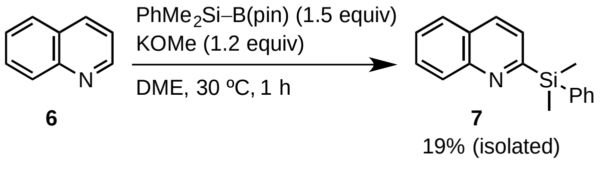

Potassium methoxide (0.60 mmol, 42.2 mg) was added to a vial sealed with a screw cap

containing a silicon-coated rubber septum in a glove box under argon atmosphere. After the reaction vial was removed from the glove box, DME (5 mL) and (dimethylphenylsilyl)boronic acid pinacol ester (0.748 mmol, 196.0 mg) were added to the vial, then stirred for 10 min at 30 ºC. Quinoline **6** (0.500 mmol, 64.6 mg) was added dropwise with a syringe. After 1 h, the reaction mixture was analyzed by GC to check the reaction. After (dimethylphenylsilyl)boronic acid pinacol ester was consumed, H_2O was added to the mixture, then extracted three times with Et_2O. The organic layer was washed with water. The combined organic layer was then dried over $MgSO_4$ followed by filtration and evaporation. The crude product was purified by silica-gel column chromatography (treated by 3% Et_3N/hexane solution) with 0−3% hexane/Et_2O eluent. A volatile byproduct, dimethyl(phenyl)silanol, was removed by keeping the sample under a reduced pressure (4.6×10^{-1} hPa) at 40 ºC for 2 h to give **7** in 19% isolated yield (24.4 mg, 0.09 mmol).

^{1}H NMR (392 MHz, $CDCl_3$, δ): 0.71 (s, 6H), 7.33−7.41 (m, 3H), 7.48−7.55 (m, 2H), 7.61−7.67 (m, 2H), 7.67−7.73 (m, 1H), 7.76 (d, *J* = 7.9 Hz, 1H), 8.00 (d, *J* = 8.2 Hz, 1H), 8.19 (d, *J* = 8.3 Hz, 1H). ^{13}C NMR (99 MHz, $CDCl_3$, δ): −3.0 (CH_3), 125.8 (*C*H), 126.5 (*C*H), 127.3 (*C*), 127.7 (*C*H), 127.9 (*C*H), 129.0 (*C*H), 129.3 (*C*H), 130.1 (*C*H), 133.2 (*C*H), 134.3 (*C*H), 137.5 (*C*) 148.9 (*C*), 168.7 (*C*). HRMS-ESI (*m*/*z*): $[M+H]^+$ calcd for $C_{17}H_{18}NSi$, 264.12030; found, 264.12012.

(*Z*)-2-(2-Cyclohexylvinyl)-4,4,5,5-tetramethyl-1,3,2-dioxaborolane [(*Z*)-9a].

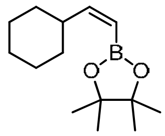

The reaction was performed according to the typical procedure **C** with (*Z*)-**8a** [*Z*/*E* = 98.5 : 1.5 (based on GC analysis), 119.7 mg, 0.507 mmol]. The title compound was purified by silica-gel column chromatography (hexane : Et_2O = 0/100 to 3/100) to give (*Z*)-**9a** in 71% isolated yield [84.7 mg, 0.359 mmol, (89% GC yield in the crude mixture)].

^{1}H and ^{13}C NMR spectra were in agreement with the literature.[32] ^{1}H NMR (396 MHz, $CDCl_3$, δ): 1.00−1.38 (m, 6H), 1.26 (s, 12H), 1.59−1.75 (m, 4H), 2.65−2.77 (m, 1H), 5.22 (dd, *J* = 0.7, 13.4 Hz, 1H), 6.26 (dd, *J* = 9.4, 13.4 Hz, 1H). ^{13}C NMR (100 MHz, $CDCl_3$, δ): 24.8 (CH_3), 25.7 (CH_2), 26.0 (CH_2), 33.3 (CH_2), 40.6 (*C*H), 82.7 (*C*), 114.0−119.0 (brs, B*C*H) 160.6 (*C*H). HRMS-EI (*m*/*z*): $[M]^+$ calcd for $C_{14}H_{25}O_2{}^{11}B$, 236.19476; found, 236.19495.

(*E*)-2-(2-Cyclohexylvinyl)-4,4,5,5-tetramethyl-1,3,2-dioxaborolane [(*E*)-9a].

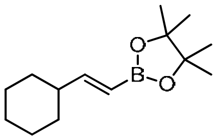

The reaction was performed according to the typical procedure **C** with (*E*)-**8a** [*Z*/*E* = 0 : 100 (based on GC analysis), 118.9 mg, 0.504 mmol]. The title compound was purified by silica-gel column chromatography (hexane : Et_2O = 0/100 to 2.5/100) to give(*E*)-**9a** in 68% isolated yield [81.0 mg, 0.343 mmol, (86% GC yield in the crude mixture)].

^{1}H and ^{13}C NMR spectra were in agreement with the literature.[33] ^{1}H NMR (396 MHz, $CDCl_3$, δ): 1.02−1.33 (m, 5H), 1.27 (s, 12H), 1.60−1.68 (m, 1H), 1.68−1,79 (m, 4H), 1.96−2.08 (m, 1H), 5.38 (dd, *J* = 1,4, 18.1 Hz, 1H), 6.58 (dd, *J* = 6.4, 18.1 Hz, 1H). ^{13}C NMR (100 MHz, $CDCl_3$, δ): 24.8 (CH_3), 25.9 (CH_2), 26.1 (CH_2), 31.9 (CH_2), 43.2 (*C*H), 83.0 (*C*), 159.9 (*C*H). The carbon directly attached to the boron atom was not detected, likely due to quadropolar relaxation. HRMS-EI (*m*/*z*): $[M]^+$ calcd for $C_{14}H_{25}O_2{}^{11}B$, 236.19476; found, 236.19483.

(Z)-2-(Hept-1-en-1-yl)-4,4,5,5-tetramethyl-1,3,2-dioxaborolane [(*Z*)-9b].

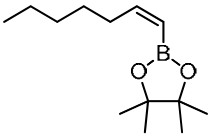

The reaction was performed according to the typical procedure **C** with (*E*)-**8b** [*Z*/*E* = 98.5 : 1.5 (based on GC analysis), 114.6 mg, 0.511 mmol]. The title compound was purified by silica-gel column chromatography using hexane/Et_2O eluents to give (*Z*)-**9b** in 72% isolated yield [82.5 mg, 0.368 mmol, (83% NMR yield)].

The coupling constant of the alkene protons (*J* = 13.6 Hz) in the product was consistent with *Z* configuration and smaller than that of (*E*)-**9b** reported in the literature (*J* = 17.9 Hz).[34] ^{1}H NMR (396 MHz, $CDCl_3$, δ): 0.89 (t, *J* = 7.0 Hz, 3H), 1.28 (s, 12H), 1.25−1.44 (m, 6H), 2.40 (dq, *J* = 1.1, 7.3 Hz, 2H), 5.33 (dd, *J* = 1.4, 13.6 Hz, 1H), 6.44 (dt, *J* = 7.0, 13.9 Hz, 1H). ^{13}C NMR (100 MHz, $CDCl_3$, δ): 14.0 (CH_3), 22.4 (CH_2), 24.8 (CH_3), 29.1 (CH_2), 31.2 (CH_2), 32.1 (CH_2), 82.7 (*C*), 155.3 (*C*H). The carbon directly attached to the boron atom was not detected, likely due to quadropolar relaxation. HRMS-EI (*m*/*z*): $[M]^+$ calcd for $C_{13}H_{25}O_2{}^{11}B$, 224.19476; found, 224.19441.

(Z)-4,4,5,5-Tetramethyl-2-(4-phenylbut-1-en-1-yl)-1,3,2-dioxaborolane [(*Z*)-9c].

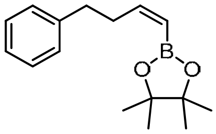

The reaction was performed according to the typical procedure **C** with (*E*)-**8c** [*Z*/*E* = 99.5 : 0.5 (based on GC analysis), 130.4 mg, 0.505 mmol]. The title compound was purified by silica-gel column chromatography (Et_2O/hexane = 0/100 to 2.5/100) to give (*Z*)-**9c** in 64% isolated yield [83.5 mg, 0.323 mmol, (80% GC yield in the crude mixture)].

The coupling constant of the alkene protons in the product was smaller than that of (*E*)-**9c** reported in the literature (*J* = 18.0 Hz).[35] ^{1}H NMR (396 MHz, $CDCl_3$, δ): 1.26 (s, 12H), 2.66−2.77 (m, 4H), 5.37 (d, *J* = 14.0 Hz, 1H), 6.40−6.54 (m, 1H), 7.14−7.30 (m, 5H). ^{13}C NMR (100 MHz, $CDCl_3$, δ): 24.8 (CH_3), 33.9 (CH_2), 36.0 (CH_2), 82.8 (*C*), 125.7 (*C*H), 128.2 (*C*H), 128.5 (*C*H), 141.9 (*C*), 153.8 (*C*H). The carbon directly attached to the boron atom was not detected, likely due to quadropolar relaxation. HRMS-EI (*m*/*z*): $[M]^+$ calcd for $C_{16}H_{23}O_2{}^{11}B$, 258.1794; found, 258.1799.

4,4,5,5-Tetramethyl-2-(5-phenylpent-1-en-2-yl)-1,3,2-dioxaborolane (9d).

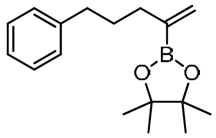

The reaction was performed according to the typical procedure **C** with **8d** (130.4 mg, 0.502 mmol). The title compound was purified by silica-gel column chromatography (Et_2O/hexane = 0/100 to 2/100) to give **9d** in 43% isolated yield (58.7 mg, 0.216 mmol).

^{1}H and ^{13}C NMR spectra were in agreement with the literature.[36] ^{1}H NMR (401 MHz, $CDCl_3$, δ): 1.27 (s, 12H), 1.71−1.81 (m, 2H), 2.21 (t, *J* = 7.6 Hz, 2H), 2.61 (t, *J* = 7.8 Hz, 2H), 5.58−5.65 (m, 1H), 5.79 (d, *J* = 3.6 Hz, 1H), 7.13−7.21 (m, 3H), 7.23−7.30 (m, 2H). ^{13}C NMR (100 MHz, $CDCl_3$, δ): 24.7 (CH_3), 30.9 (CH_2), 35.1 (CH_2), 35.5 (CH_2), 83.3 (*C*), 125.5 (*C*H), 128.2 (*C*H), 128.4 (*C*H), 129.3 (CH_2), 142.8 (*C*). The carbon directly attached to the boron atom was not detected, likely due to quadropolar relaxation. HRMS-EI (*m*/*z*): $[M]^+$ calcd for $C_{17}H_{25}O_2B$, 272.19507; found, 272.19422.

(*E*)-4,4,5,5-Tetramethyl-2-(5-phenylpent-2-en-2-yl)-1,3,2-dioxaborolane [(*E*)-9e].

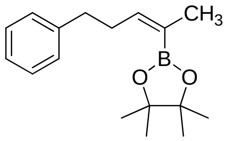

The reaction was performed according to the typical procedure **C** with **8e** [*Z*/*E* = 6 : 94 (based on GC analysis), 136.0 mg, 0.500 mmol). The title compound was purified by silica-gel column chromatography (Et_2O/hexane = 0/100 to 1/100) to give (*E*)-**9e** in 64% isolated yield (86.9 mg, 0.319 mmol). The geometry of the alkenyl group in the product was determined by NOE analysis as shown in the following figure.

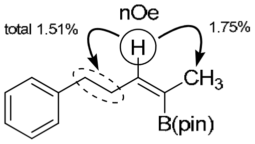

^{1}H NMR (401 MHz, $CDCl_3$, δ): 1.27 (s, 12H), 1.75 (s, 3H), 2.56−2.70 (m, 4H), 6.06−6.18 (m, 1H), 7.14−7.30 (m, 5H). ^{13}C NMR (100 MHz, $CDCl_3$, δ): 22.3 (CH_3), 24.8 (CH_3), 33.0 (CH_2), 36.6 (CH_2), 82.8 (*C*), 125.6 (*C*H), 128.2 (*C*H), 128.5 (*C*H), 142.4 (*C*), 146.2 (*C*H). The carbon directly attached to the boron atom was not detected, likely due to quadropolar relaxation. HRMS-EI (*m*/*z*): $[M]^+$ calcd for $C_{17}H_{25}O_2B$, 272.19507; found, 272.19399.

(*Z*)-6-Phenylhex-5-en-1-yl benzoate [(*Z*)-13g].

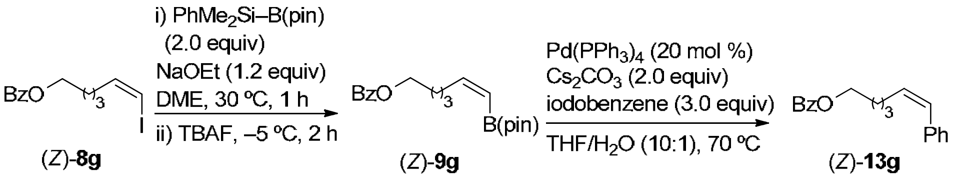

The borylation reaction was performed according to the typical procedure **C** with **8g** [*Z*/*E* = 100 : 0 (based on GC analysis), 165.1 mg, 0.500 mmol]. The borylated compound was roughly purified by silica-gel column chromatography (Et_2O/hexane = 0/100 to 2.5/100). The resultant, concentrated crude product was placed in a 20 mL-Schlenk flask. After that, the flask was connected to a vacuum-nitrogen manifold, and it was evacuated and refilled with nitrogen three times. Then, $Pd(PPh_3)_4$ (115.3 mg, 0.1 mmol), Cs_2CO_3 (329.2 mg, 1.0 mmol), THF (6.75 mL), N_2-bubbled H_2O (0.75 mL) and iodobenzene (309.0 mg, 1.5 mmol) were successively added to the flask. The resulting mixture was heated to 70 ºC and stirred for 24 h. After that, the reaction mixture was cooled to ambient temperature, and was diluted with ether and water, extracted three times with ether. The combined organic layer was dried over $MgSO_4$ followed by filtration and evaporation. The crude product was purified by silica-gel column chromatography (Et_2O/hexane = 0/100 to 5/100) to give the title compound (*Z*)-**13g** in 51% yield [71.5 mg, 0.255 mmol, (70% NMR yield in the crude reaction mixture)]. The geometry of the alkenyl group in the product was determined by NOE

analysis as shown in the following figure.

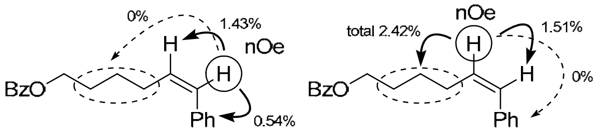

^{1}H NMR (392 MHz, $CDCl_3$, δ): 1.56–1.70 (m, 2H), 1.76–1.86 (m, 2H), 2.42 (dq, *J* = 1.8, 7.4 Hz, 2H), 4.31 (t, *J* = 6.5 Hz, 2H), 5.67 (dt, *J* = 7.3, 11.7 Hz, 1H), 6.45 (d, *J* = 11.8 Hz, 1H), 7.18–7.35 (m, 5H), 7.39–7.47 (m, 2H), 7.55 (tt, *J* = 1.5, 7.4 Hz, 1H), 8.00–8.07 (m, 2H). ^{13}C NMR (99 MHz, $CDCl_3$, δ): 26.4 (CH_2), 28.2 (CH_2), 28.4 (CH_2), 64.8 (CH_2), 126.5 (*C*H), 128.1 (*C*H), 128.3 (*C*H), 128.7 (*C*H), 129.3 (*C*H), 129.5 (*C*), 130.4 (*C*H), 132.3 (*C*H), 132.8 (*C*), 137.5 (*C*), 166.6 (*C*). HRMS-ESI (*m*/*z*): $[M+Na]^+$ calcd for $C_{19}H_{20}O_2Na$, 303.13555; found, 303.13589.

(*Z*)-4,4,5,5-Tetramethyl-2-{6-[(tetrahydro-2H-pyran-2-yl)oxy]hex-1-en-1-yl}-1,3,2-dioxaborolane [(*Z*)-9h].

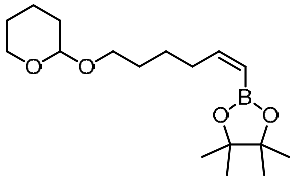

The reaction was performed according to the typical procedure **C** with **8h** [*Z*/*E* = 100 : 0 (based on GC analysis), 155.1 mg, 0.500 mmol]. The title compound was purified by silica-gel column chromatography (Et_2O/hexane = 0/100 to 10/100) followed by Kugelrohr distillation under a reduced pressure (50 °C, 0.45 to 0.38 hPa) to give (*Z*)-**9h** in 74% isolated yield (114.5 mg, 0.369 mmol). The geometry of the alkenyl group in the product was determined by NOE analysis as shown in the following figure.

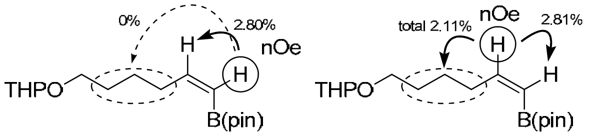

^{1}H NMR (392 MHz, $CDCl_3$, δ): 1.27 (s, 12H), 1.40–1.90 (m, 10H), 2.43 (q, *J* = 7.5 Hz, 2H), 3.40 (dt, *J* = 6.6, 9.6 Hz, 1H), 3.46–3.53 (m, 1H), 3.75 (dt, *J* = 6.7, 9.6 Hz, 1H), 3.83–3.92 (m, 1H), 4.56–4.60 (m, 1H), 5.34 (d, *J* = 13.6 Hz, 1H), 6.43 (dt, *J* = 7.2, 14.0 Hz, 1H). ^{13}C NMR (99 MHz, $CDCl_3$, δ): 19.6 (CH_2), 24.8 (CH_3), 25.5 (CH_2), 26.0 (CH_2), 29.1 (CH_2), 30.7 (CH_2), 31.9 (CH_2), 62.2 (CH_2), 67.4 (CH_2), 82.8 (*C*), 98.7 (*C*H), 154.8 (*C*H). HRMS-ESI (*m*/*z*): $[M+Na]^+$ calcd for $C_{17}H_{31}O_4Na$, 332.22439; found, 332.22452.

2-{2-[(Methoxymethoxy)methyl]cyclohex-1-en-1-yl}-4,4,5,5-tetramethyl-1,3,2-dioxaborolane (9i).

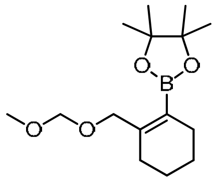

The reaction was performed according to the typical procedure **C** with **8i** (117.6 mg, 0.500 mmol). The title compound was purified by silica-gel column chromatography (Et_2O/hexane = 0/100 to 10/100) to give **9i** in 58% isolated yield (82.0 mg, 0.291 mmol).

^{1}H NMR (392 MHz, $CDCl_3$, δ): 1.27 (s, 12H), 1.51−1.67 (m, 4H), 2.10−2.18 (m, 4H), 3.39 (s, 3H), 4.19 (s, 2H), 4.64 (s, 2H). ^{13}C NMR (99 MHz, $CDCl_3$, δ): 22.28 (*C*H_2), 22.33 (*C*H_2), 24.8 (*C*H_3), 27.8 (*C*H_2), 28.4 (*C*H_2), 55.1 (*C*H_3), 70.5 (*C*H_2), 83.0 (*C*), 95.9 (*C*H_2), 146.7 (*C*). HRMS-ESI (*m*/*z*): $[M+Na]^+$ calcd for $C_{15}H_{27}O_4{}^{10}BNa$, 304.19309; found, 304.19315.

6-Cyclohexyl-4-(4-nitrophenyl)-3,6-dihydro-2*H*-pyran (13j).

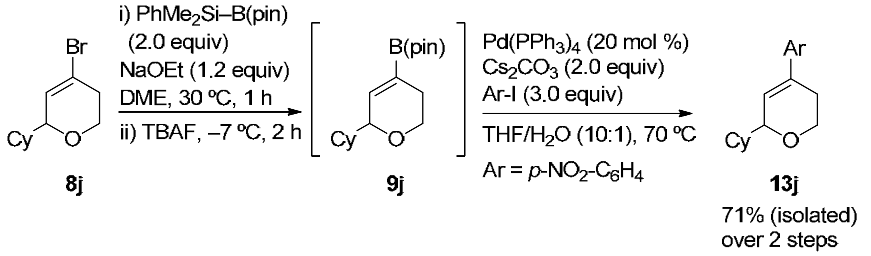

The reaction was performed according to the typical procedure **C** with **8j** (123.5 mg, 0.504 mmol). After the TBAF treatment, the resultant reaction mixture was added to water, then extracted three times with ether. The resulting crude solution was placed in a 20 mL-Schlenk flask and concentrated. After that, the flask was connected to a vacuum-nitrogen manifold, and it was evacuated and refilled with nitrogen three times. Then, $Pd(PPh_3)_4$ (116.6 mg, 0.1 mmol), Cs_2CO_3 (327.4 mg, 1.0 mmol), THF (6.75 mL), N_2-bubbled H_2O (0.75 mL) and 1-iodo-4-nitrobenzene (373.8 mg, 1.5 mmol) were successively added to the flask. The resulting mixture was heated to 70 ºC and stirred for 24 h. After that, the reaction mixture was cooled to ambient temperature, and was diluted with ether and water, extracted three times with ether. The combined organic layer was dried over $MgSO_4$ followed by filtration and evaporation. The crude product was purified by silica-gel column chromatography (Et_2O/hexane = 0/100 to 5/100) to give the title compound **13j** in 71% yield (103.5 mg, 0.360 mmol).

^{1}H NMR (401 MHz, $CDCl_3$, δ): 1.09−1.36 (m, 5H), 1.54−1.84 (m, 6H), 2.26−2.35 (m, 1H), 2.62−2.74 (m, 1H), 3.74 (dt, *J* = 3.3, 10.9 Hz, 1H), 4.04−4.10 (m, 1H), 4.18 (ddd, *J* = 2.2, 5.8, 11.6 Hz, 1H), 6.29 (s, 1H), 7.51−7.56 (m, 2H), 8.18−8.22 (m, 2H). ^{13}C NMR (99 MHz, $CDCl_3$, δ): 26.25 (CH_2), 26.28 (CH_2), 26.4 (CH_2), 27.2 (CH_2), 28.1 (CH_2), 28.9 (CH_2), 43.0 (*C*H), 63.6 (CH_2), 78.6 (*C*H), 123.8 (*C*H), 125.3 (*C*H), 129.6 (*C*H), 133.6 (*C*), 146.7 (*C*). HRMS-EI (*m*/*z*): $[M]^+$ calcd for $C_{17}H_{21}NO_3$, 287.15214; found, 287.15092.

Butyl 2,2,4-trimethyl-3-(4,4,5,5-tetramethyl-1,3,2-dioxaborolan-2-yl)cyclohex-3-enecarboxylate (9k).

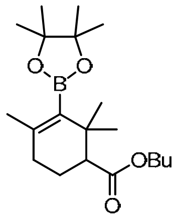

The reaction was performed according to the typical procedure **C** with **8k** (153.2 mg, 0.505 mmol). The title compound was purified by silica-gel column chromatography (Et_2O/hexane = 0/100 to 7/100) to give **9k** in 53% isolated yield (93.2 mg, 0.291 mmol).

^{1}H NMR (392 MHz, $CDCl_3$, δ): 0.92 (t, *J* = 7.5 Hz, 3H), 1.08 (s, 3H), 1.18 (s, 3H), 1.31 (s, 6H), 1.32 (s, 6H), 1.33−1.44 (m, 2H), 1.56−1.66 (m, 3H), 1.76 (s, 3H), 1.81−1.93 (m, 1H), 1.96−2.02 (m, 2H), 2.30 (dd, *J* = 3.1, 12.4 Hz, 1H), 4.03 (*J* = 6.6, 10.9 H, 1H), 4.10 (dt, *J* = 6.8, 10.9 Hz, 1H). ^{13}C NMR (99 MHz, $CDCl_3$, δ): 13.7 (CH_3), 19.2 (CH_2), 22.0 (CH_2), 23.5 (CH_3), 24.0 (CH_3), 24.8 (CH_3), 25.1 (CH_3), 29.1 (CH_3), 30.7 (CH_2), 31.4 (CH_2), 36.1 (*C*), 50.7 (*C*H), 63.8 (CH_2), 83.2 (*C*), 140.2 (*C*), 175.2 (*C*). HRMS-ESI (*m*/*z*): $[M]^+$ calcd for $C_{20}H_{36}O_4{}^{10}B$, 350.27375; found, 350.27430.

5. References

1) Suginome, M.; Matsuda, T.; Ito, Y. *Orgnometallics* **2000**, *19*, 5917.

2) Fleming, I.; Roberts, R. S.; Smith, S. C. *J. Chem. Soc., Perkin Trans. 1* **1998**, 1209.

3) Gigante, B.; Esteves, A. M.; Pires, N.; Davies, L. M.; Douglas, P.; Fonseca, M. S.; Burrows, D. H.; Castro, A. E. R.; Pina, J.; Melo, J. S. *New J. Chem.* **2009**, *33*, 877.

4) Hee-Choon, A.; Soo-Jin, Y.; Seung-Ae, K.; Kyung-Joo, L., Tae-Jin, L.; Hyuck-Joo, K.; Bong-Ok, K. Patent WO2014/61991 A1, **2014**.

5) Sakamoto, T.; Kondo, Y.; Suginome, T.; Ohba, S.; Yamanaka, H. *Synthesis* **1992**, *6*, 552.

6) Eli Lilly and Company Patent WO2006/102194 A1, **2006**.

7) Katz, D. J.; Jewell, P. J.; Guerin, J. D.; Lim, J.;Dinsmore, J. C.; Deshmukh, V. S.; Pan, B.; Marshall, C. G,; Lu, W.; Altman, D. M.; Dahlberg, K. W.; Davis, L.; Falcone, D.; Gabarda, E. A.; Hang, G.; Hatch, H.; Holmes, R.; Kunii, K.; Lumb, J. K.; Lutterbach, B.; Mathvink, R.; Nazef, N.; Patel, B. S.; Qu, X.; Reilly, F. J.; Rickert, W. K.; Rosenstein, C.; Soisson, M. S.; SpencerB. K.; Szewczak, A, A,; Walker, D.; Wang, W.; Young, J.; Zeng, Q. *J. Med. Chem.* **2011**, *51*, 4092.

8) Mitsubishi Tanabe Pharma Corporation Patent EP1956009 A1, **2008**.

9) Denmark, S. E.; Yang, S.-M. *J. Am. Chem. Soc.* **2002**, *124*, 2102.

10) Harrowven, D. C.; Pascoe, D. D.; Guy, I. L. *Angew. Chem., Int. Ed.* **2007**, *46*, 425.

11) Ishihara, Y.; Mendoza, A.; Baran, P. S. *Tetrahedron* **2013**, *69*, 5685.

12) Brozek, L. A.; Sieber, J. D.; Morken, J. P. *Org. Lett*. **2011**, *12*, 995.

13) Beshai, M.; Dhudshia, B.; Mills, R.; Thadani, A. N. *Tetrahedron Lett*. **2008**, *49*, 6794.

14) Coleman, B. E.; Cwynar, V.; Hart, D. J.; Havas, F.; Mohan, J. M.; Patterson, S.; Ridenour, S.; Schmidt, M.; Smith, E.; Wells, A. J. *Synlett* **2004**, *8*, 1339.

15) Menzel, K.; Fu, G. C. *J. Am. Chem. Soc.* **2003**, *125*, 3718.

16) Uenishi, J.; Iwamoto, T.; Ohmi, M. *Tetrahedron Lett*. **2007**, *48*, 1237.

17) Chen, J.; Wang, T.; Zhao, K. *Tetrahedron Lett*. **1994**, *35*, 2827.

18) Miranda, P. O.; Díaz, D. D.; Padrón, J. I.; Bermejo, J.; Martín, V. S. *Org. Lett.* **2003**, *5*, 1979.

19) James, A. N.; Lena, H.; Wayne, D. B.; Paul, M. W.; Steven, V. L.; Duncan, L. B. *Chem. Eur. J.* **2014**, *20*, 263.

20) Di, Q.; Liang. J.; Zhitong, Z.; He, M.; Fanyang, M.; Xi, W.; Yan, Z.; Jianbo, W. *J. Org. Chem.* **2013**, *78*, 1923.

21) Shen, R.; Chen, T.; Zhao, Y.; Qiu, R.; Zhou, Y.; Yin, S.; Wang, X.; Goto, M.; Han, L. *J. Am. Chem. Soc.* **2011**, *133*, 17037.

22) Kitamura, Y.; Sako, S.; Tsutsui, A.; Monguchi, Y.; Maegawa, T.; Kitada, Y.; Sajiki, H. *Adv. Synth.*

Catal. **2010**, *352*, 718.

23) Kim, S. H.; Cho, I.; Sim, M. K.; Park, S.; Park, S. Y. *J. Mater. Chem.* **2011**, *21*, 9139.

24) Jianguo, J.; Tao, L.; Christopher, L. L.; Murali, G. Patent US2008/45539 A1, **2010**.

25) Ghayoor, A. C.; Venkata, A. K.; Robert, E. M.; Milton, R. S. *Tetrahedron* **2008**, *64*, 6103.

26) Liu, H.; Hancock, A. A.; Cowart, M. D.; Hancock, K. J. Patent US/2007/78133 A1, **2007**.

27) Calle, M.; Cuadrado, P.; Gonzeález-Nogal, M. A.; Valero, R. *Synthesis* **2001**, *13*, 1949.

28) Wenjun, T.; Santosh, K.; Yongda, Z.; Xudong, W.; Jolaine, S.; Nitinchandra, D. P.; Nathan, K. Y.; Chris, H. S. *Org. Lett.* **2011**, *13*, 1366.

29) Rao, L. N. M.; Dhanovkar, J. R. *Eur. J. Org. Chem.* **2014**, 5214.

30) Kumar, V. K. R.; Krishnakumar, S.; Gopidas, K. R. *Eur. J. Org. Chem.* **2012**, 3447.

31) Larsen, A. M.; Hartwig, J. F. *J. Am. Chem. Soc.* **2014**, *136*, 4287.

32) Molander, G. A.; Ellis, N. M. *J. Org. Chem.* **2008**, *73*, 6841.

33) Haberberger, M.; Enthaler, S. *Chem. Asian J.* **2013**, *8*, 50.

34) Pietruszka, J.; Rieche, A. C. M.; Wilhelm, T.; Witt, A. *Adv. Synth. Catal.* **2003**, *345*, 1273.

35) Takai, K.; Kunisada, Y.; Tachibana, Y.; Yamaji, N.; Nakatani, E. *Bull. Chem. Soc. Jpn.* **2004**, *77*, 1581.

36) Moure, A. L.; Mauleón, P.; Arrayás, R. G.; Carretero, J. C. *Org. Lett.* **2013**, *15*, 2054.

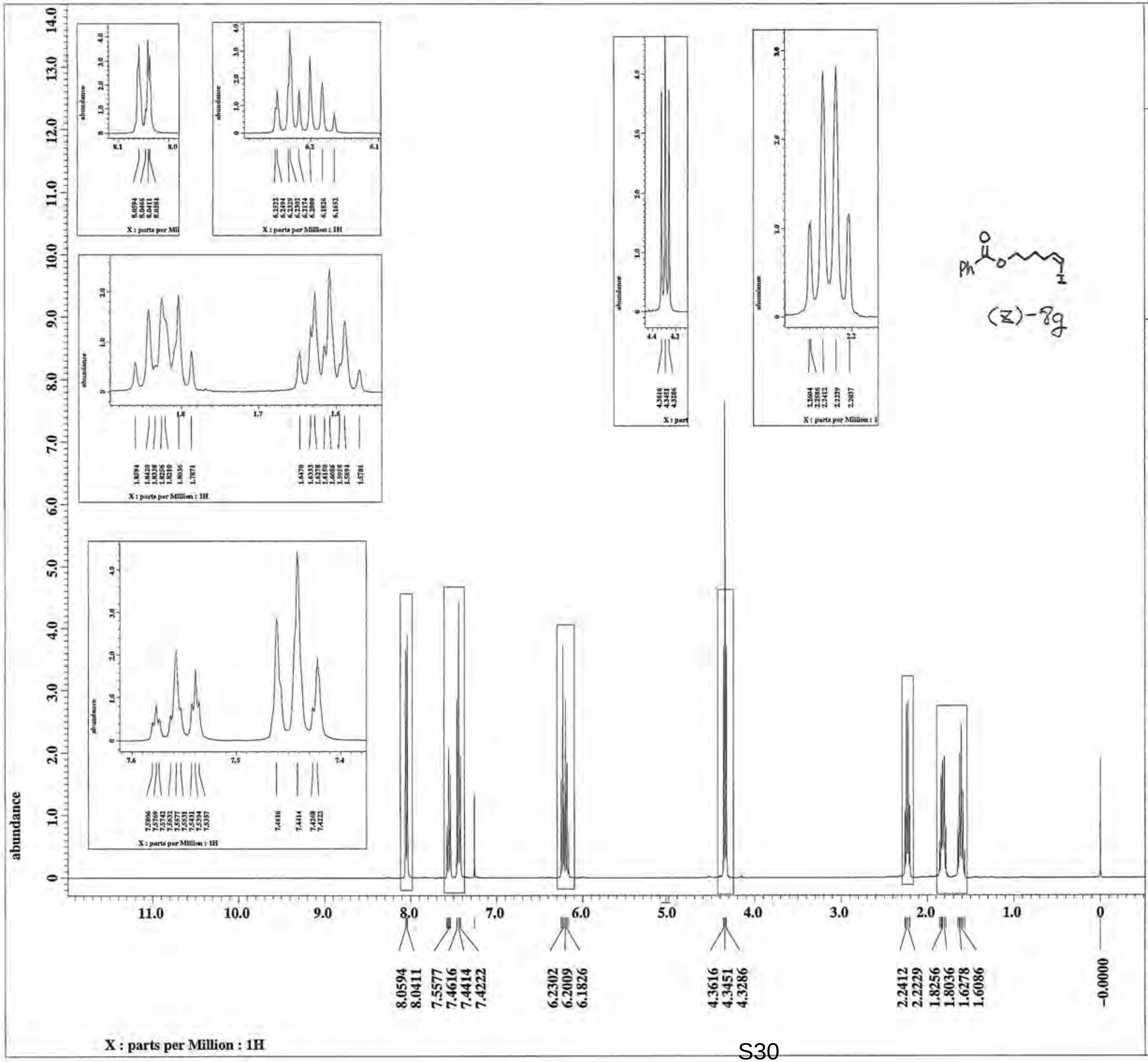

---- PROCESSING PARAMETERS ----
dc_balance : 0 : FALSE
sexp : 0.2[Hz] : 0.0[s]
trapezoid3 : 0[%] : 80[%] : 100[%]
zerofill : 1
fft : 1 : TRUE : TRUE
machinephase
ppm

Derived from: EIYA490H-1.jdf

Filename	= EIYA490H-5.jdf
Author	= element
Experiment	= single_pulse.ex2
Sample_id	= S#408176
Solvent	= CHLOROFORM-D
Creation_time	= 8-DEC-2014 10:59:25
Revision_time	= 8-DEC-2014 11:28:10
Current_time	= 8-DEC-2014 11:28:12
Comment	= single_pulse
Data_format	= 1D COMPLEX
Dim_size	= 16384
Dim_title	= 1H
Dim_units	= [ppm]
Dimensions	= X
Site	= ECS 400
Spectrometer	= JNM-ECS400
Field_strength	= 9.20197068[T] (390[MH
X_acq_duration	= 2.78790144[s]
X_domain	= 1H
X_freq	= 391.78655441[MHz]
X_offset	= 5[ppm]
X_points	= 16384
X_prescans	= 1
X_resolution	= 0.35869274[Hz]
X_sweep	= 5.87682181[kHz]
Irr_domain	= 1H
Irr_freq	= 391.78655441[MHz]
Irr_offset	= 5[ppm]
Tri_domain	= 1H
Tri_freq	= 391.78655441[MHz]
Tri_offset	= 5[ppm]
Clipped	= FALSE
Mod_return	= 1
Scans	= 8
Total_scans	= 8
X_90_width	= 10.7[us]
X_acq_time	= 2.78790144[s]
X_angle	= 45[deg]
X_atn	= 1.9[dB]
X_pulse	= 5.35[us]
Irr_mode	= Off
Tri_mode	= Off
Dante_presat	= FALSE
Initial_wait	= 1[s]
Recvr_gain	= 38
Relaxation_delay	= 5[s]
Repetition_time	= 7.78790144[s]
Temp_get	= 19.9[dC]

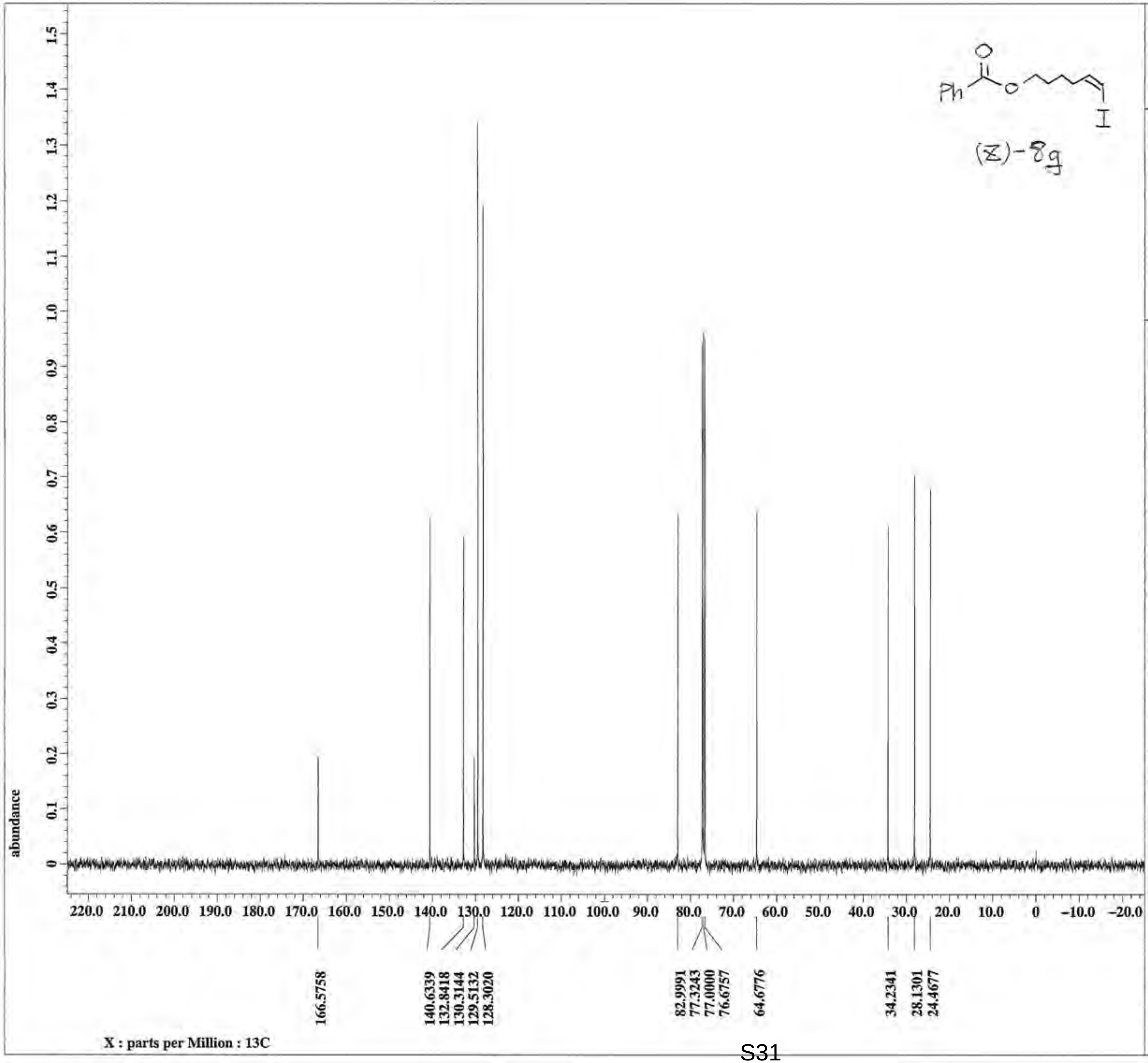

---- PROCESSING PARAMETERS ----
dc_balance : 0 : FALSE
sexp : 2.0[Hz] : 0.0[s]
trapezoid3 : 0[%] : 80[%] : 100[%]
zerofill : 1
fft : 1 : TRUE : TRUE
machinephase
ppm

Derived from: EIYA490C_copy-2.jdf

Filename	= EIYA490C_copy-4.jdf
Author	= element
Experiment	= single_pulse_dec
Sample_id	= S#409938
Solvent	= CHLOROFORM-D
Creation_time	= 8-DEC-2014 11:09:46
Revision_time	= 8-DEC-2014 11:33:53
Current_time	= 8-DEC-2014 11:34:05
Comment	= single pulse decouple
Data_format	= 1D COMPLEX
Dim_size	= 26214
Dim_title	= 13C
Dim_units	= [ppm]
Dimensions	= X
Site	= ECS 400
Spectrometer	= JNM-ECS400
Field_strength	= 9.20197068[T] (390[MH
X_acq_duration	= 1.06430464[s]
X_domain	= 13C
X_freq	= 98.51479726[MHz]
X_offset	= 100[ppm]
X_points	= 32768
X_prescans	= 4
X_resolution	= 0.93958061[Hz]
X_sweep	= 30.78817734[kHz]
Irr_domain	= 1H
Irr_freq	= 391.78655441[MHz]
Irr_offset	= 5[ppm]
Clipped	= FALSE
Incomplete_copy	= TRUE
Mod_return	= 1
Scans	= 173
Total_scans	= 173
X_90_width	= 8.8[us]
X_acq_time	= 1.06430464[s]
X_angle	= 30[deg]
X_atn	= 4.9[dB]
X_pulse	= 2.93333333[us]
Irr_atn_dec	= 22.52628[dB]
Irr_atn_noe	= 22.52628[dB]
Irr_noise	= WALTZ
Decoupling	= TRUE
Initial_wait	= 1[s]
Noe	= TRUE
Noe_time	= 2[s]
Recvr_gain	= 60
Relaxation_delay	= 2[s]
Repetition_time	= 3.06430464[s]
Temp_get	= 20.2[dC]

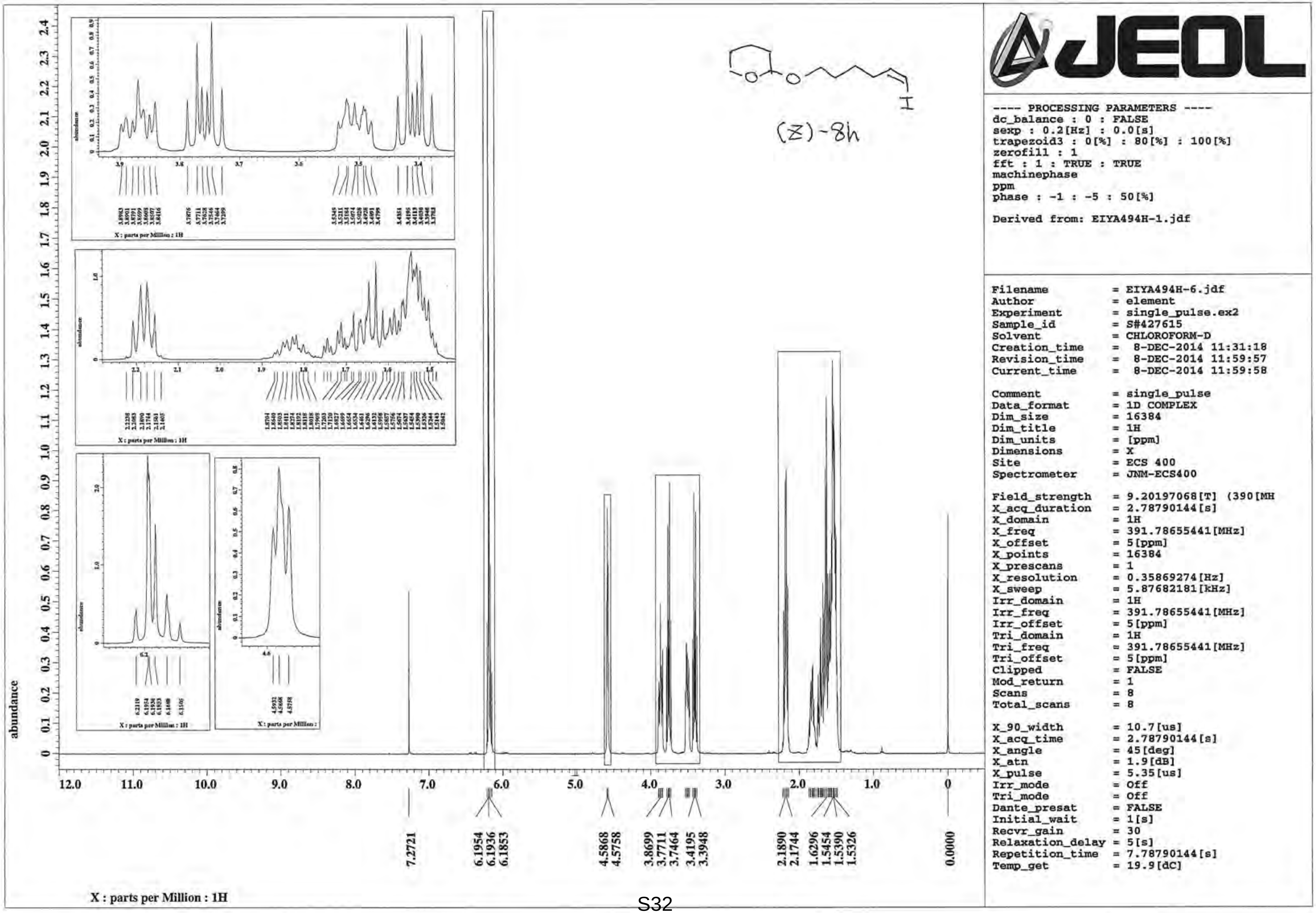

(Z)-8h
abundance
X : parts per Million : 1H
7.2721
6.1954
6.1936
6.1853
4.5868
4.5758
3.8699
3.7711
3.7464
3.4195
3.3948
2.1890
2.1744
1.6296
1.5454
1.5390
1.5326
0.0000
JEOL
---- PROCESSING PARAMETERS ----
dc_balance : 0 : FALSE
sexp : 0.2[Hz] : 0.0[s]
trapezoid3 : 0[%] : 80[%] : 100[%]
zerofill : 1
fft : 1 : TRUE : TRUE
machinephase
ppm
phase : -1 : -5 : 50[%]
Derived from: EIYA494H-1.jdf
Filename = EIYA494H-6.jdf
Author = element
Experiment = single_pulse.ex2
Sample_id = S#427615
Solvent = CHLOROFORM-D
Creation_time = 8-DEC-2014 11:31:18
Revision_time = 8-DEC-2014 11:59:57
Current_time = 8-DEC-2014 11:59:58
Comment = single_pulse
Data_format = 1D COMPLEX
Dim_size = 16384
Dim_title = 1H
Dim_units = [ppm]
Dimensions = X
Site = ECS 400
Spectrometer = JNM-ECS400
Field_strength = 9.20197068[T] (390[MH
X_acq_duration = 2.78790144[s]
X_domain = 1H
X_freq = 391.78655441[MHz]
X_offset = 5[ppm]
X_points = 16384
X_prescans = 1
X_resolution = 0.35869274[Hz]
X_sweep = 5.87682181[kHz]
Irr_domain = 1H
Irr_freq = 391.78655441[MHz]
Irr_offset = 5[ppm]
Tri_domain = 1H
Tri_freq = 391.78655441[MHz]
Tri_offset = 5[ppm]
Clipped = FALSE
Mod_return = 1
Scans = 8
Total_scans = 8
X_90_width = 10.7[us]
X_acq_time = 2.78790144[s]
X_angle = 45[deg]
X_atn = 1.9[dB]
X_pulse = 5.35[us]
Irr_mode = Off
Tri_mode = Off
Dante_presat = FALSE
Initial_wait = 1[s]
Recvr_gain = 30
Relaxation_delay = 5[s]
Repetition_time = 7.78790144[s]
Temp_get = 19.9[dC]

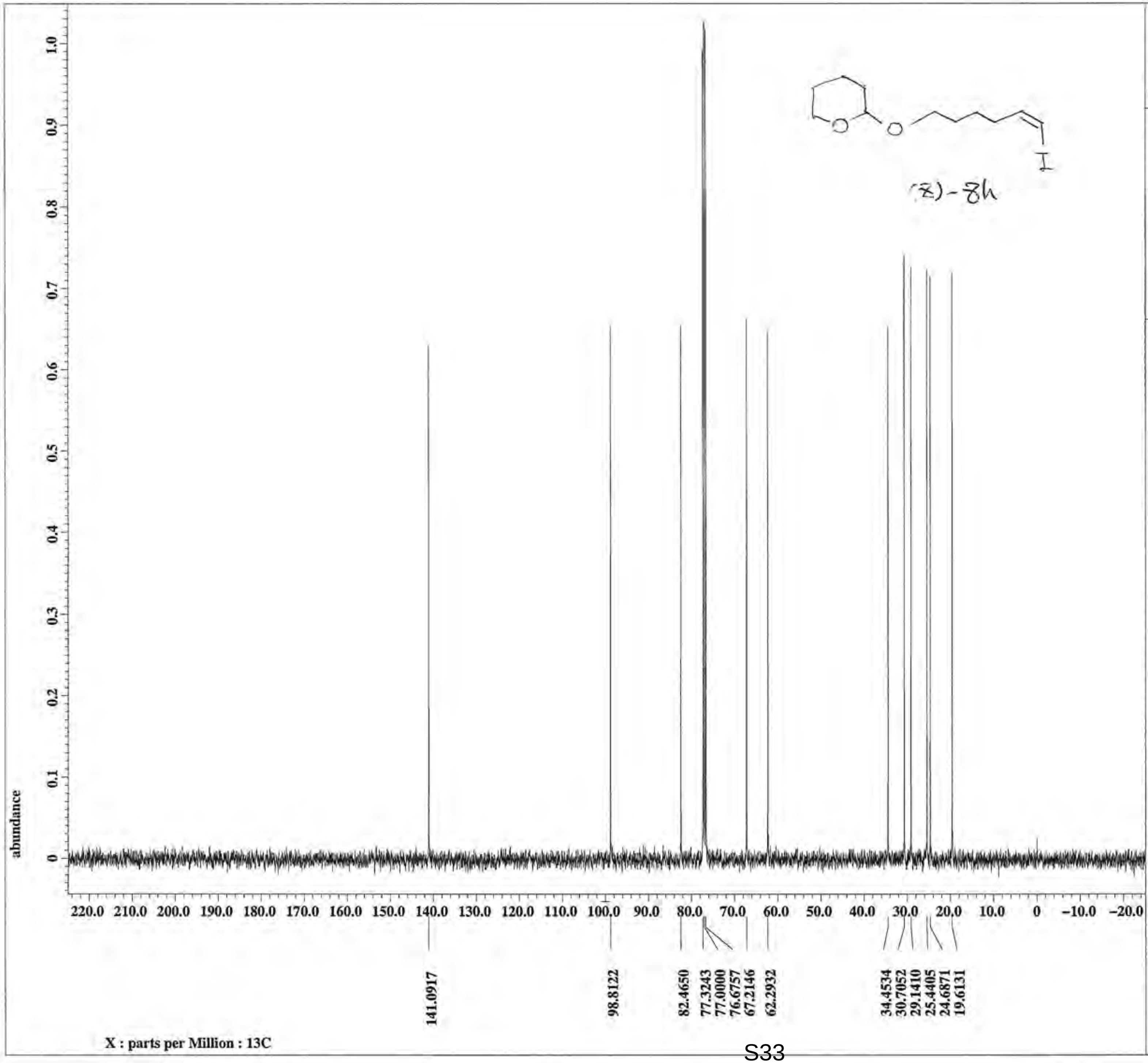

---- PROCESSING PARAMETERS ----
dc_balance : 0 : FALSE
sexp : 2.0[Hz] : 0.0[s]
trapezoid3 : 0[%] : 80[%] : 100[%]
zerofill : 1
fft : 1 : TRUE : TRUE
machinephase
ppm

Derived from: EIYA494C_copy-2.jdf

Filename	= EIYA494C_copy-4.jdf
Author	= element
Experiment	= single_pulse_dec
Sample_id	= S#428978
Solvent	= CHLOROFORM-D
Creation_time	= 8-DEC-2014 11:41:33
Revision_time	= 8-DEC-2014 12:05:32
Current_time	= 8-DEC-2014 12:05:51
Comment	= single pulse decouple
Data_format	= 1D COMPLEX
Dim_size	= 26214
Dim_title	= 13C
Dim_units	= [ppm]
Dimensions	= X
Site	= ECS 400
Spectrometer	= JNM-ECS400
Field_strength	= 9.20197068[T] (390[MH
X_acq_duration	= 1.06430464[s]
X_domain	= 13C
X_freq	= 98.51479726[MHz]
X_offset	= 100[ppm]
X_points	= 32768
X_prescans	= 4
X_resolution	= 0.93958061[Hz]
X_sweep	= 30.78817734[kHz]
Irr_domain	= 1H
Irr_freq	= 391.78655441[MHz]
Irr_offset	= 5[ppm]
Clipped	= FALSE
Incomplete_copy	= TRUE
Mod_return	= 1
Scans	= 168
Total_scans	= 168
X_90_width	= 8.8[us]
X_acq_time	= 1.06430464[s]
X_angle	= 30[deg]
X_atn	= 4.9[dB]
X_pulse	= 2.93333333[us]
Irr_atn_dec	= 22.52628[dB]
Irr_atn_noe	= 22.52628[dB]
Irr_noise	= WALTZ
Decoupling	= TRUE
Initial_wait	= 1[s]
Noe	= TRUE
Noe_time	= 2[s]
Recvr_gain	= 60
Relaxation_delay	= 2[s]
Repetition_time	= 3.06430464[s]
Temp_get	= 20.2[dC]

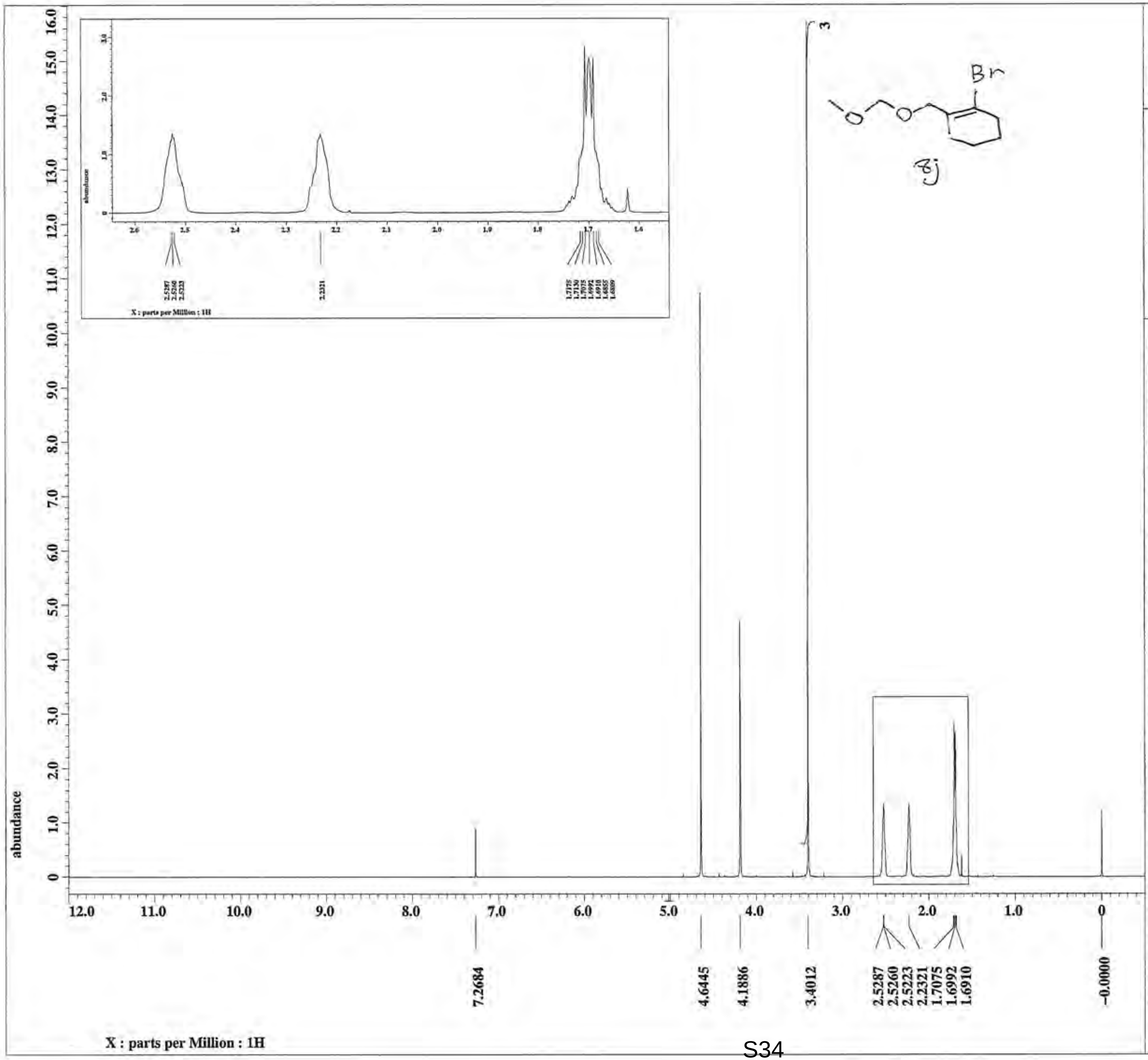

JEOL

---- PROCESSING PARAMETERS ----
dc_balance : 0 : FALSE
sexp : 0.2[Hz] : 0.0[s]
trapezoid3 : 0[%] : 80[%] : 100[%]
zerofill : 1
fft : 1 : TRUE : TRUE
machinephase
ppm

Derived from: EIYA232RP-H-1.jdf

Filename = EIYA232RP-H-4.jdf
Author = element
Experiment = single_pulse.ex2
Sample_id = S#584176
Solvent = CHLOROFORM-D
Creation_time = 8-DEC-2014 15:52:44
Revision_time = 8-DEC-2014 16:21:14
Current_time = 8-DEC-2014 16:21:17

Comment = single_pulse
Data_format = 1D COMPLEX
Dim_size = 16384
Dim_title = 1H
Dim_units = [ppm]
Dimensions = X
Site = ECS 400
Spectrometer = JNM-ECS400

Field_strength = 9.20197068[T] (390[MH
X_acq_duration = 2.78790144[s]
X_domain = 1H
X_freq = 391.78655441[MHz]
X_offset = 5[ppm]
X_points = 16384
X_prescans = 1
X_resolution = 0.35869274[Hz]
X_sweep = 5.87682181[kHz]
Irr_domain = 1H
Irr_freq = 391.78655441[MHz]
Irr_offset = 5[ppm]
Tri_domain = 1H
Tri_freq = 391.78655441[MHz]
Tri_offset = 5[ppm]
Clipped = FALSE
Mod_return = 1
Scans = 8
Total_scans = 8

X_90_width = 10.7[us]
X_acq_time = 2.78790144[s]
X_angle = 45[deg]
X_atn = 1.9[dB]
X_pulse = 5.35[us]
Irr_mode = Off
Tri_mode = Off
Dante_presat = FALSE
Initial_wait = 1[s]
Recvr_gain = 36
Relaxation_delay = 5[s]
Repetition_time = 7.78790144[s]
Temp_get = 20.1[dC]

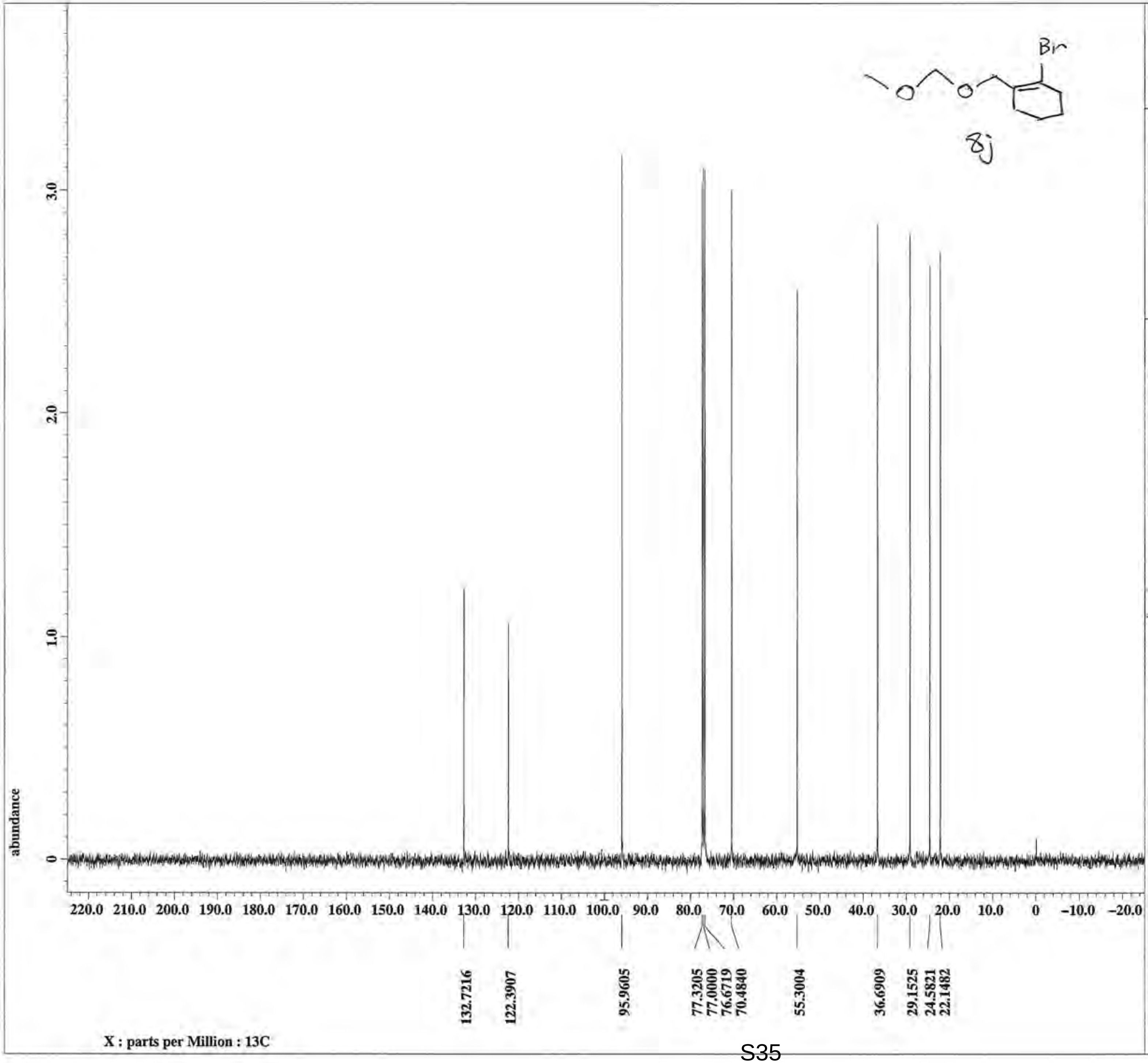

---- PROCESSING PARAMETERS ----
dc_balance : 0 : FALSE
sexp : 2.0[Hz] : 0.0[s]
trapezoid3 : 0[%] : 80[%] : 100[%]
zerofill : 1
fft : 1 : TRUE : TRUE
machinephase
ppm

Derived from: EIYA232RP-C_copy-3.jdf

Filename	= EIYA232RP-C_copy-6.jd
Author	= element
Experiment	= single_pulse_dec
Sample_id	= S#589639
Solvent	= CHLOROFORM-D
Creation_time	= 8-DEC-2014 16:09:01
Revision_time	= 8-DEC-2014 16:32:58
Current_time	= 8-DEC-2014 16:33:21
Comment	= single pulse decouple
Data_format	= 1D COMPLEX
Dim_size	= 32768
Dim_title	= 13C
Dim_units	= [ppm]
Dimensions	= X
Site	= ECS 400
Spectrometer	= JNM-ECS400
Field_strength	= 9.20197068[T] (390[MH
X_acq_duration	= 1.3303808[s]
X_domain	= 13C
X_freq	= 98.51479726[MHz]
X_offset	= 100[ppm]
X_points	= 32768
X_prescans	= 4
X_resolution	= 0.75166449[Hz]
X_sweep	= 24.63054187[kHz]
Irr_domain	= 1H
Irr_freq	= 391.78655441[MHz]
Irr_offset	= 5[ppm]
Clipped	= FALSE
Incomplete_copy	= TRUE
Mod_return	= 1
Scans	= 151
Total_scans	= 151
X_90_width	= 8.8[us]
X_acq_time	= 1.3303808[s]
X_angle	= 30[deg]
X_atn	= 4.9[dB]
X_pulse	= 2.93333333[us]
Irr_atn_dec	= 22.52628[dB]
Irr_atn_noe	= 22.52628[dB]
Irr_noise	= WALTZ
Decoupling	= TRUE
Initial_wait	= 1[s]
Noe	= TRUE
Noe_time	= 2[s]
Recvr_gain	= 70
Relaxation_delay	= 2[s]
Repetition_time	= 3.3303808[s]
Temp_get	= 20.4[dC]

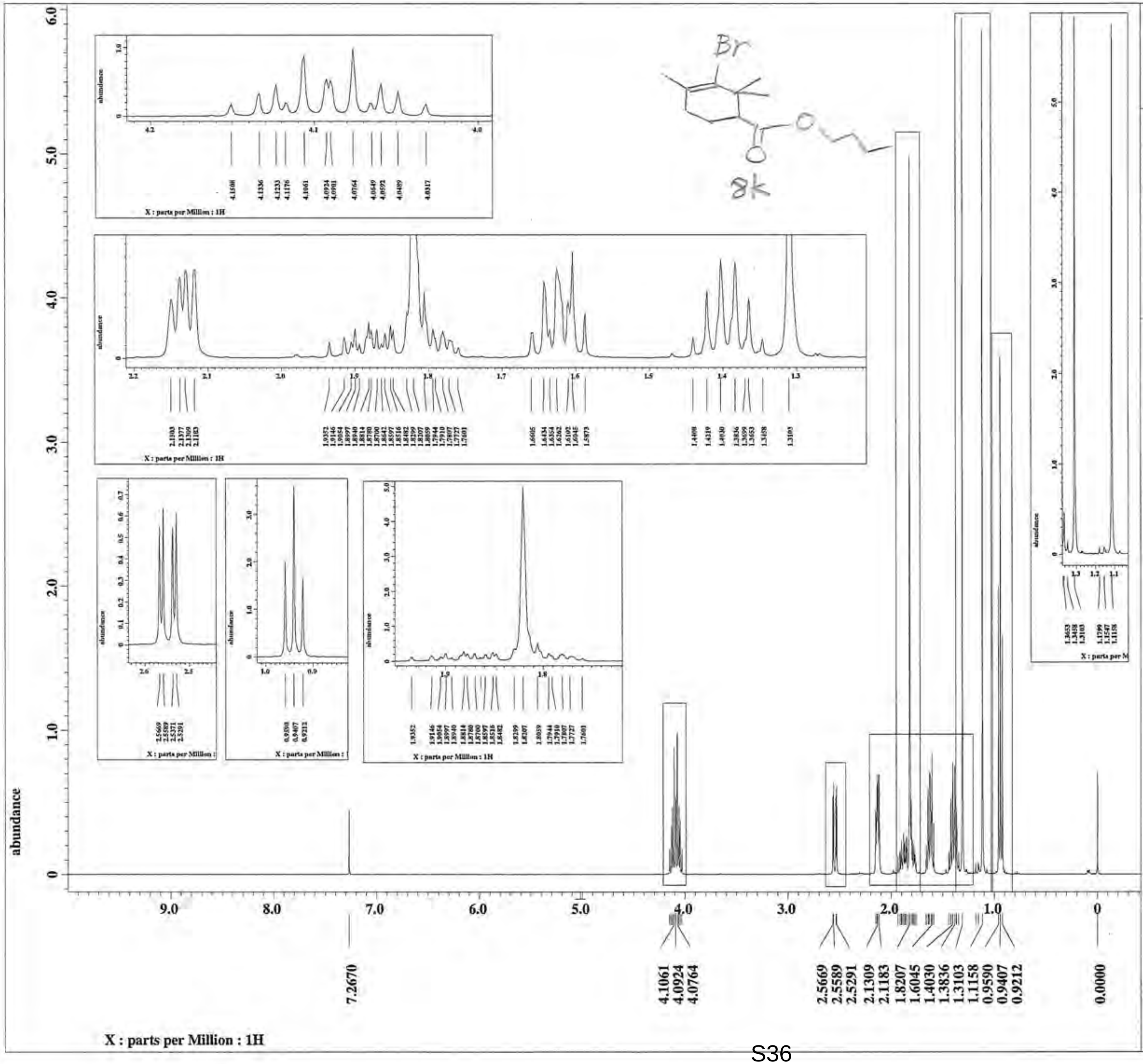

JEOL

---- PROCESSING PARAMETERS ----
dc_balance : 0 : FALSE
sexp : 0.2[Hz] : 0.0[s]
trapezoid3 : 0[%] : 80[%] : 100[%]
zerofill : 1
fft : 1 : TRUE : TRUE
machinephase
ppm

Derived from: EIYA449-2-1.jdf

Filename	= EIYA449-2-5.jdf
Author	= element
Experiment	= single_pulse.ex2
Sample_id	= S#744669
Solvent	= CHLOROFORM-D
Creation_time	= 5-DEC-2014 20:41:09
Revision_time	= 5-DEC-2014 20:47:43
Current_time	= 5-DEC-2014 20:47:48
Comment	= single_pulse
Data_format	= 1D COMPLEX
Dim_size	= 13107
Dim_title	= 1H
Dim_units	= [ppm]
Dimensions	= X
Site	= ECX 400P
Spectrometer	= DELTA2_NMR
Field_strength	= 9.2982153[T] (400[MHz
X_acq_duration	= 2.20725248[s]
X_domain	= 1H
X_freq	= 395.88430144[MHz]
X_offset	= 5[ppm]
X_points	= 16384
X_prescans	= 1
X_resolution	= 0.45305193[Hz]
X_sweep	= 7.42280285[kHz]
Irr_domain	= 1H
Irr_freq	= 395.88430144[MHz]
Irr_offset	= 5[ppm]
Tri_domain	= 1H
Tri_freq	= 395.88430144[MHz]
Tri_offset	= 5[ppm]
Clipped	= FALSE
Mod_return	= 1
Scans	= 8
Total_scans	= 8
X_90_width	= 12.6[us]
X_acq_time	= 2.20725248[s]
X_angle	= 45[deg]
X_atn	= 1[dB]
X_pulse	= 6.3[us]
Irr_mode	= Off
Tri_mode	= Off
Dante_presat	= FALSE
Initial_wait	= 1[s]
Recvr_gain	= 30
Relaxation_delay	= 5[s]
Repetition_time	= 7.20725248[s]
Temp_get	= 22.6[dC]

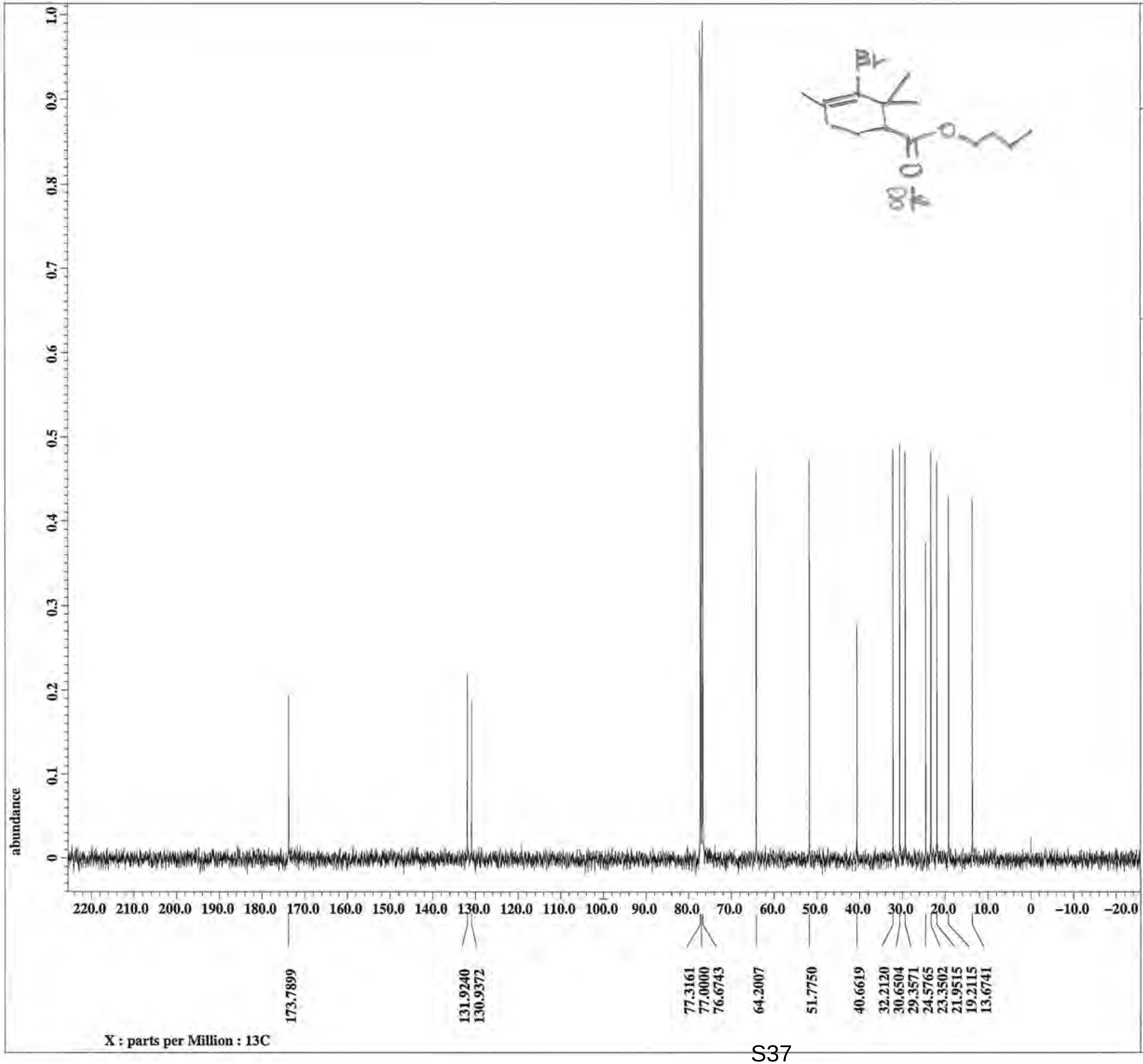


```
---- PROCESSING PARAMETERS ----
dc_balance : 0 : FALSE
sexp : 2.0[Hz] : 0.0[s]
trapezoid3 : 0[%] : 80[%] : 100[%]
zerofill : 1
fft : 1 : TRUE : TRUE
machinephase
ppm

Derived from: EIYA499C-1.jdf
```

```
Filename            = EIYA499C-3.jdf
Author              = element
Experiment          = single_pulse_dec
Sample_id           = S#733419
Solvent             = CHLOROFORM-D
Creation_time       =  5-DEC-2014 20:31:44
Revision_time       =  5-DEC-2014 20:34:56
Current_time        =  5-DEC-2014 20:35:11

Comment             = single pulse decouple
Data_format         = 1D COMPLEX
Dim_size            = 26214
Dim_title           = 13C
Dim_units           = [ppm]
Dimensions          = X
Site                = ECX 400P
Spectrometer        = DELTA2_NMR

Field_strength      = 9.2982153[T] (400[MHz
X_acq_duration      = 1.048576[s]
X_domain            = 13C
X_freq              = 99.54517646[MHz]
X_offset            = 100[ppm]
X_points            = 32768
X_prescans          = 4
X_resolution        = 0.95367432[Hz]
X_sweep             = 31.25[kHz]
Irr_domain          = 1H
Irr_freq            = 395.88430144[MHz]
Irr_offset          = 5[ppm]
Clipped             = TRUE
Mod_return          = 1
Scans               = 202
Total_scans         = 202

X_90_width          = 9.3[us]
X_acq_time          = 1.048576[s]
X_angle             = 30[deg]
X_atn               = 3.4[dB]
X_pulse             = 3.1[us]
Irr_atn_dec         = 20.20655[dB]
Irr_atn_noe         = 20.20655[dB]
Irr_noise           = WALTZ
Decoupling          = TRUE
Initial_wait        = 1[s]
Noe                 = TRUE
Noe_time            = 2[s]
Recvr_gain          = 58
Relaxation_delay    = 2[s]
Repetition_time     = 3.048576[s]
Temp_get            = 22.8[dC]
```

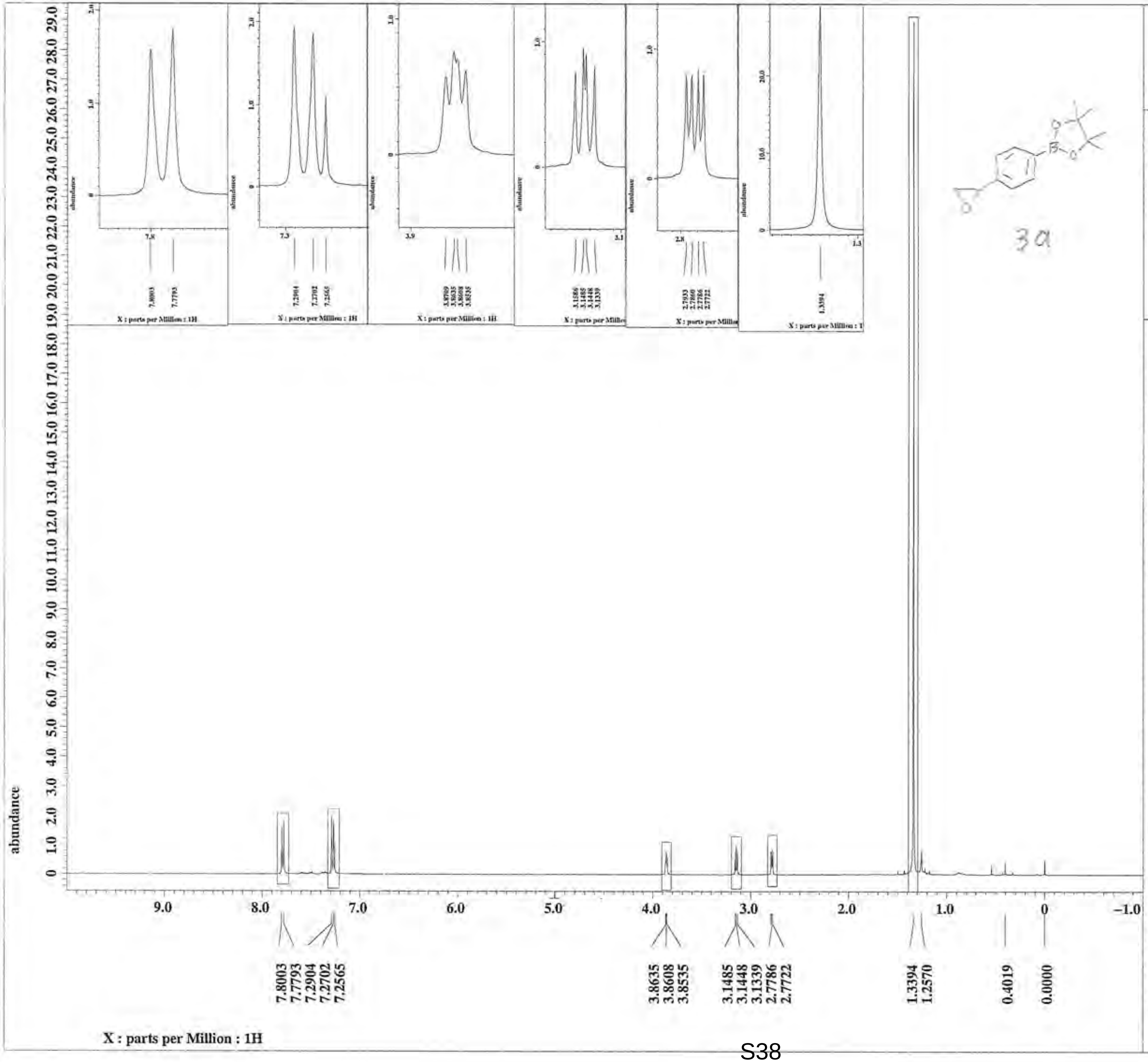

---- PROCESSING PARAMETERS ----
dc_balance : 0 : FALSE
sexp : 0.2[Hz] : 0.0[s]
trapezoid3 : 0[%] : 80[%] : 100[%]
zerofill : 1
fft : 1 : TRUE : TRUE
machinephase
ppm

Derived from: UKI-107-A proton-1.jdf

Filename	= UKI-107-A proton-4.jd
Author	= element
Experiment	= single_pulse.ex2
Sample_id	= 107
Solvent	= CHLOROFORM-D
Creation_time	= 31-JUL-2014 09:21:09
Revision_time	= 31-JUL-2014 09:34:55
Current_time	= 31-JUL-2014 09:34:57
Comment	= single_pulse
Data_format	= 1D COMPLEX
Dim_size	= 16384
Dim_title	= 1H
Dim_units	= [ppm]
Dimensions	= X
Site	= ECS 400
Spectrometer	= JNM-ECS400
Field_strength	= 9.20197068[T] (390[MH
X_acq_duration	= 2.78790144[s]
X_domain	= 1H
X_freq	= 391.78655441[MHz]
X_offset	= 5[ppm]
X_points	= 16384
X_prescans	= 1
X_resolution	= 0.35869274[Hz]
X_sweep	= 5.87682181[kHz]
Irr_domain	= 1H
Irr_freq	= 391.78655441[MHz]
Irr_offset	= 5[ppm]
Tri_domain	= 1H
Tri_freq	= 391.78655441[MHz]
Tri_offset	= 5[ppm]
Clipped	= FALSE
Mod_return	= 1
Scans	= 15
Total_scans	= 15
X_90_width	= 10.7[us]
X_acq_time	= 2.78790144[s]
X_angle	= 45[deg]
X_atn	= 1.9[dB]
X_pulse	= 5.35[us]
Irr_mode	= Off
Tri_mode	= Off
Dante_presat	= FALSE
Initial_wait	= 1[s]
Recvr_gain	= 30
Relaxation_delay	= 4[s]
Repetition_time	= 6.78790144[s]
Temp_get	= 21.1[dC]

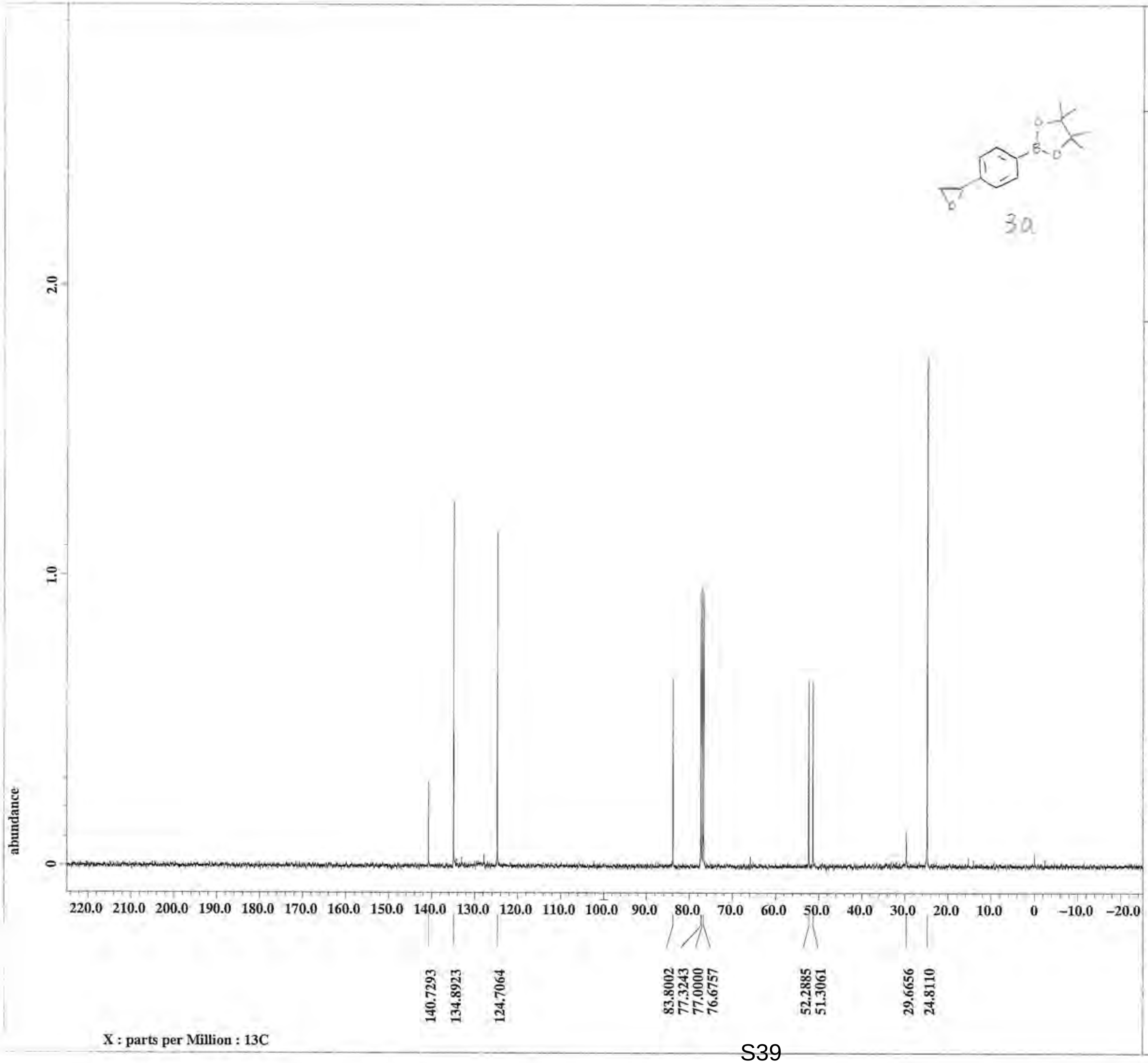


```
---- PROCESSING PARAMETERS ----
dc_balance : 0 : FALSE
sexp : 2.0[Hz] : 0.0[s]
trapezoid3 : 0[%] : 80[%] : 100[%]
zerofill : 1
fft : 1 : TRUE : TRUE
machinephase
ppm

Derived from: UKI-107-B carbon2-1.jdf

Filename          = UKI-107-B carbon2-3.j
Author            = element
Experiment        = single_pulse_dec
Sample_id         = 107
Solvent           = CHLOROFORM-D
Creation_time     =  4-OCT-2014 13:43:03
Revision_time     =  4-OCT-2014 14:03:04
Current_time      =  4-OCT-2014 14:03:11

Comment           = single pulse decouple
Data_format       = 1D COMPLEX
Dim_size          = 26214
Dim_title         = 13C
Dim_units         = [ppm]
Dimensions        = X
Site              = ECS 400
Spectrometer      = JNM-ECS400

Field_strength    = 9.20197068[T] (390[MH
X_acq_duration    = 1.06430464[s]
X_domain          = 13C
X_freq            = 98.51479726[MHz]
X_offset          = 100[ppm]
X_points          = 32768
X_prescans        = 4
X_resolution      = 0.93958061[Hz]
X_sweep           = 30.78817734[kHz]
Irr_domain        = 1H
Irr_freq          = 391.78655441[MHz]
Irr_offset        = 5[ppm]
Clipped           = FALSE
Mod_return        = 1
Scans             = 200
Total_scans       = 200

X_90_width        = 8.8[us]
X_acq_time        = 1.06430464[s]
X_angle           = 30[deg]
X_atn             = 4.9[dB]
X_pulse           = 2.93333333[us]
Irr_atn_dec       = 22.52628[dB]
Irr_atn_noe       = 22.52628[dB]
Irr_noise         = WALTZ
Decoupling        = TRUE
Initial_wait      = 1[s]
Noe               = TRUE
Noe_time          = 2[s]
Recvr_gain        = 60
Relaxation_delay  = 2[s]
Repetition_time   = 3.06430464[s]
Temp_get          = 23.6[dC]
```

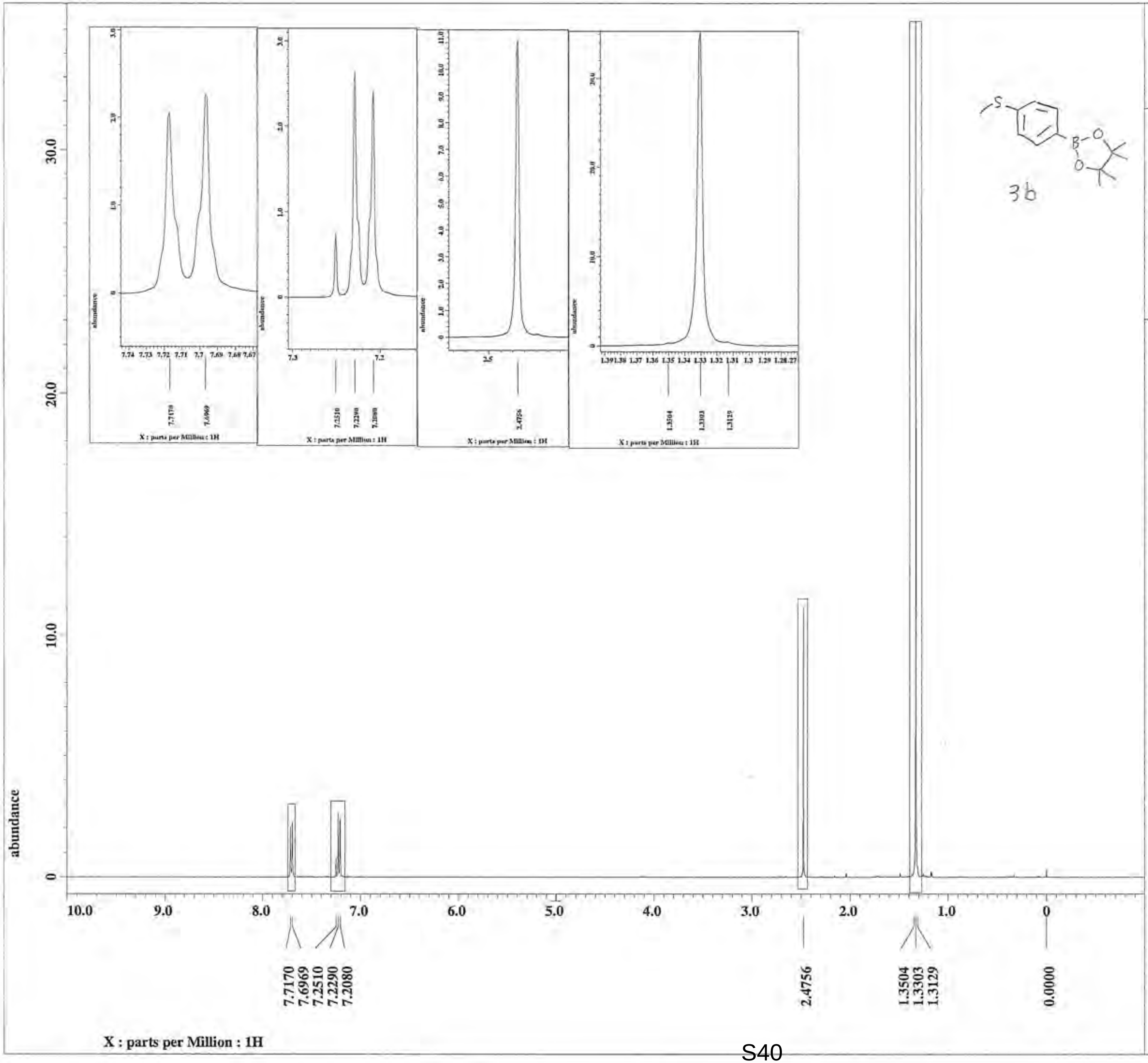


```
---- PROCESSING PARAMETERS ----
dc_balance : 0 : FALSE
sexp : 0.2[Hz] : 0.0[s]
trapezoid3 : 0[%] : 80[%] : 100[%]
zerofill : 1
fft : 1 : TRUE : TRUE
machinephase
ppm

Derived from: UKI-156-A proton-1.jdf

Filename           = UKI-156-A proton-5.jd
Author             = element
Experiment         = single_pulse.ex2
Sample_id          = UKI-156
Solvent            = CHLOROFORM-D
Creation_time      = 26-FEB-2014 18:44:17
Revision_time      = 26-FEB-2014 20:08:04
Current_time       = 26-FEB-2014 20:08:06

Comment            = single_pulse
Data_format        = 1D COMPLEX
Dim_size           = 16384
Dim_title          = 1H
Dim_units          = [ppm]
Dimensions         = X
Site               = ECS 400
Spectrometer       = JNM-ECS400

Field_strength     = 9.20197068[T] (390[MH
X_acq_duration     = 2.78790144[s]
X_domain           = 1H
X_freq             = 391.78655441[MHz]
X_offset           = 5[ppm]
X_points           = 16384
X_prescans         = 1
X_resolution       = 0.35869274[Hz]
X_sweep            = 5.87682181[kHz]
Irr_domain         = 1H
Irr_freq           = 391.78655441[MHz]
Irr_offset         = 5[ppm]
Tri_domain         = 1H
Tri_freq           = 391.78655441[MHz]
Tri_offset         = 5[ppm]
Clipped            = FALSE
Mod_return         = 1
Scans              = 16
Total_scans        = 16

X_90_width         = 10.8[us]
X_acq_time         = 2.78790144[s]
X_angle            = 45[deg]
X_atn              = 1.9[dB]
X_pulse            = 5.4[us]
Irr_mode           = Off
Tri_mode           = Off
Dante_presat       = FALSE
Initial_wait       = 1[s]
Recvr_gain         = 26
Relaxation_delay   = 4[s]
Repetition_time    = 6.78790144[s]
Temp_get           = 20[dC]
```

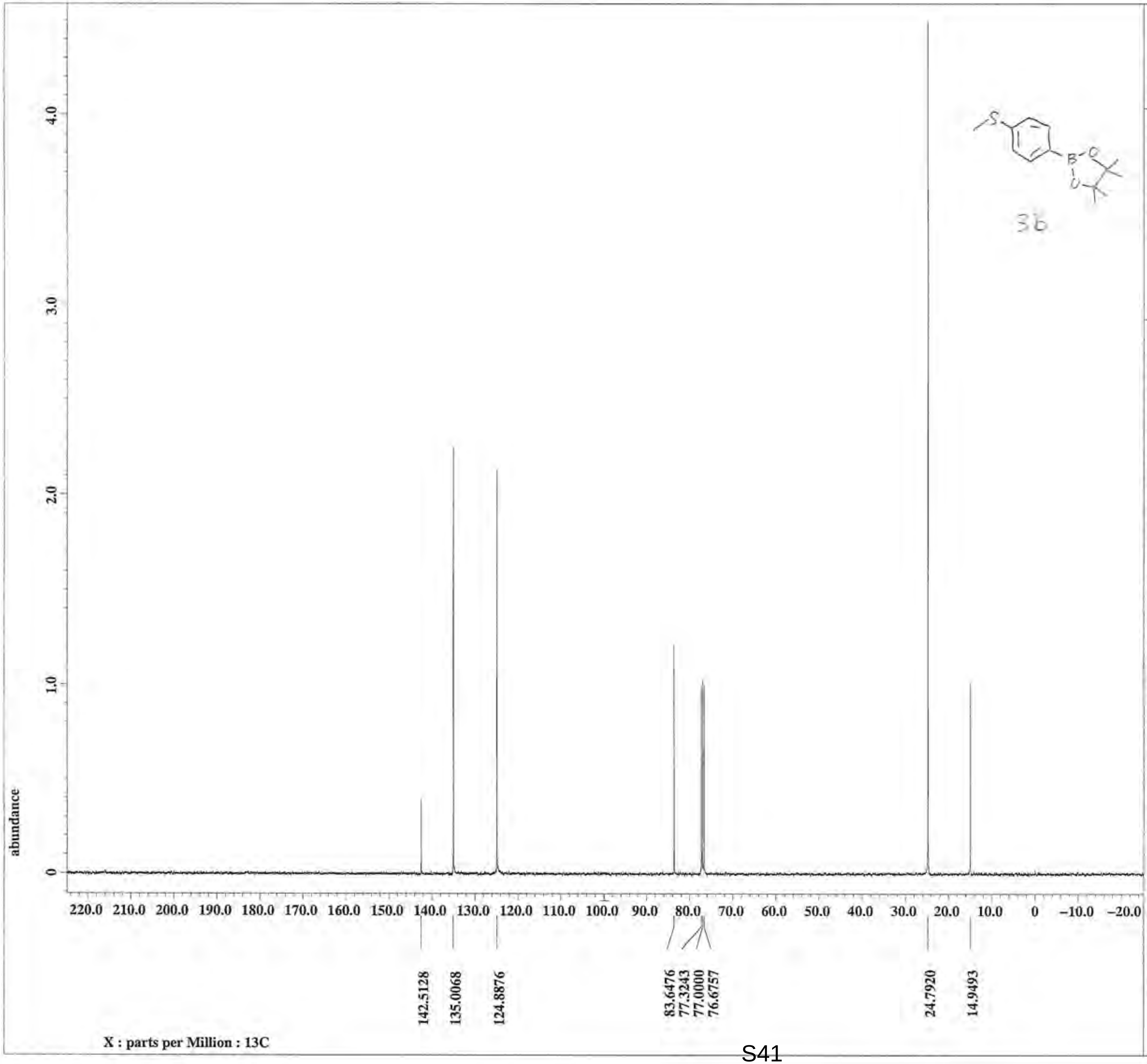

JEOL

---- PROCESSING PARAMETERS ----
dc_balance : 0 : FALSE
sexp : 2.0[Hz] : 0.0[s]
trapezoid3 : 0[%] : 80[%] : 100[%]
zerofill : 1
fft : 1 : TRUE : TRUE
machinephase
ppm

Derived from: UKI-156-B carbon-1.jdf

Filename	= UKI-156-B carbon-3.jd
Author	= element
Experiment	= single_pulse_dec
Sample_id	= UKI-156
Solvent	= CHLOROFORM-D
Creation_time	= 26-FEB-2014 18:55:44
Revision_time	= 26-FEB-2014 20:09:35
Current_time	= 26-FEB-2014 20:10:00
Comment	= single pulse decouple
Data_format	= 1D COMPLEX
Dim_size	= 26214
Dim_title	= 13C
Dim_units	= [ppm]
Dimensions	= X
Site	= ECS 400
Spectrometer	= JNM-ECS400
Field_strength	= 9.20197068[T] (390[MH
X_acq_duration	= 1.06430464[s]
X_domain	= 13C
X_freq	= 98.51479726[MHz]
X_offset	= 100[ppm]
X_points	= 32768
X_prescans	= 4
X_resolution	= 0.93958061[Hz]
X_sweep	= 30.78817734[kHz]
Irr_domain	= 1H
Irr_freq	= 391.78655441[MHz]
Irr_offset	= 5[ppm]
Clipped	= FALSE
Mod_return	= 1
Scans	= 200
Total_scans	= 200
X_90_width	= 8.15[us]
X_acq_time	= 1.06430464[s]
X_angle	= 30[deg]
X_atn	= 4.9[dB]
X_pulse	= 2.71666667[us]
Irr_atn_dec	= 22.445[dB]
Irr_atn_noe	= 22.445[dB]
Irr_noise	= WALTZ
Decoupling	= TRUE
Initial_wait	= 1[s]
Noe	= TRUE
Noe_time	= 2[s]
Recvr_gain	= 60
Relaxation_delay	= 2[s]
Repetition_time	= 3.06430464[s]
Temp_get	= 20.2[dC]

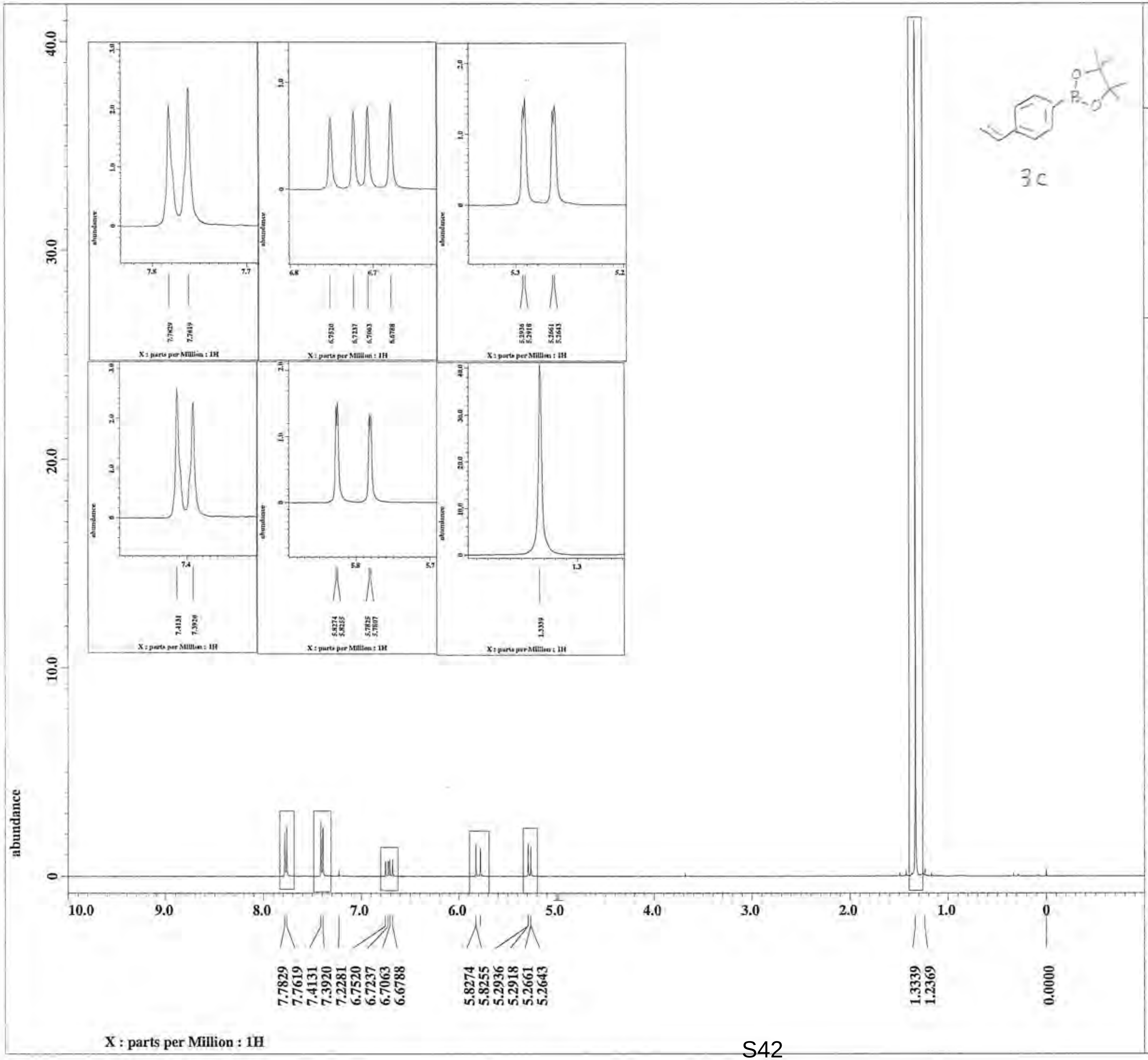

---- PROCESSING PARAMETERS ----
dc_balance : 0 : FALSE
sexp : 0.2[Hz] : 0.0[s]
trapezoid3 : 0[%] : 80[%] : 100[%]
zerofill : 1
fft : 1 : TRUE : TRUE
machinephase
ppm

Derived from: UKI-125-A proton-1.jdf

Filename = UKI-125-A proton-4.jd
Author = element
Experiment = single_pulse.ex2
Sample_id = 125
Solvent = CHLOROFORM-D
Creation_time = 27-NOV-2013 19:04:30
Revision_time = 27-NOV-2013 20:26:57
Current_time = 27-NOV-2013 20:29:09

Comment = single_pulse
Data_format = 1D COMPLEX
Dim_size = 16384
Dim_title = 1H
Dim_units = [ppm]
Dimensions = X
Site = ECS 400
Spectrometer = JNM-ECS400

Field_strength = 9.20197068[T] (390[MH
X_acq_duration = 2.78790144[s]
X_domain = 1H
X_freq = 391.78655441[MHz]
X_offset = 5[ppm]
X_points = 16384
X_prescans = 1
X_resolution = 0.35869274[Hz]
X_sweep = 5.87682181[kHz]
Irr_domain = 1H
Irr_freq = 391.78655441[MHz]
Irr_offset = 5[ppm]
Tri_domain = 1H
Tri_freq = 391.78655441[MHz]
Tri_offset = 5[ppm]
Clipped = FALSE
Mod_return = 1
Scans = 8
Total_scans = 8

X_90_width = 10.8[us]
X_acq_time = 2.78790144[s]
X_angle = 45[deg]
X_atn = 1.9[dB]
X_pulse = 5.4[us]
Irr_mode = Off
Tri_mode = Off
Dante_presat = FALSE
Initial_wait = 1[s]
Recvr_gain = 24
Relaxation_delay = 5[s]
Repetition_time = 7.78790144[s]
Temp_get = 19.6[dC]

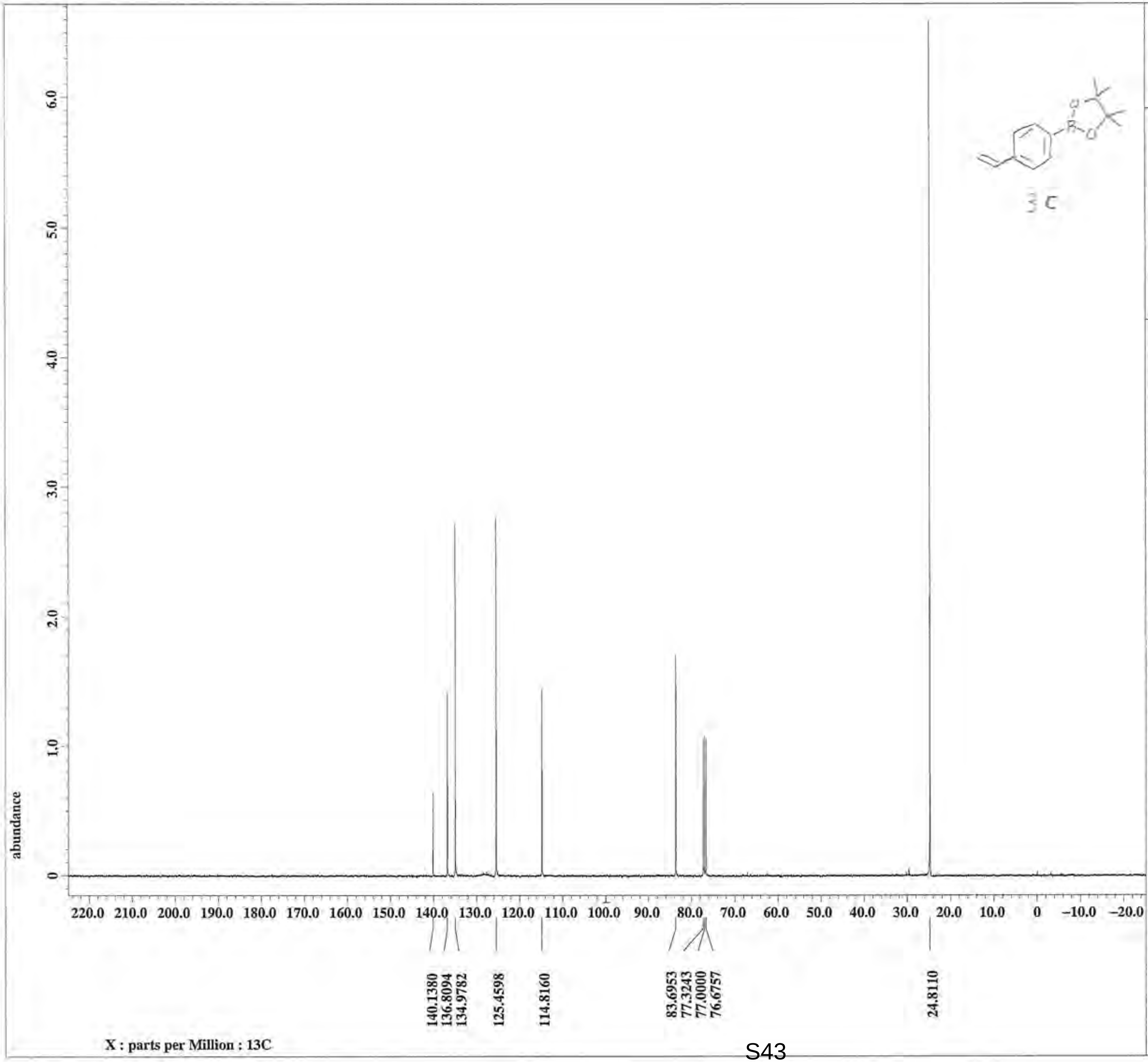

JEOL

---- PROCESSING PARAMETERS ----
dc_balance : 0 : FALSE
sexp : 2.0[Hz] : 0.0[s]
trapezoid3 : 0[%] : 80[%] : 100[%]
zerofill : 1
fft : 1 : TRUE : TRUE
machinephase
ppm

Derived from: UKI-125-B carbon-1.jdf

Filename	= UKI-125-B carbon-3.jd
Author	= element
Experiment	= single_pulse_dec
Sample_id	= 125
Solvent	= CHLOROFORM-D
Creation_time	= 27-NOV-2013 19:15:56
Revision_time	= 27-NOV-2013 20:30:03
Current_time	= 27-NOV-2013 20:30:04
Comment	= single pulse decouple
Data_format	= 1D COMPLEX
Dim_size	= 26214
Dim_title	= 13C
Dim_units	= [ppm]
Dimensions	= X
Site	= ECS 400
Spectrometer	= JNM-ECS400
Field_strength	= 9.20197068[T] (390[MH
X_acq_duration	= 1.06430464[s]
X_domain	= 13C
X_freq	= 98.51479726[MHz]
X_offset	= 100[ppm]
X_points	= 32768
X_prescans	= 4
X_resolution	= 0.93958061[Hz]
X_sweep	= 30.78817734[kHz]
Irr_domain	= 1H
Irr_freq	= 391.78655441[MHz]
Irr_offset	= 5[ppm]
Clipped	= FALSE
Mod_return	= 1
Scans	= 200
Total_scans	= 200
X_90_width	= 8.15[us]
X_acq_time	= 1.06430464[s]
X_angle	= 30[deg]
X_atn	= 4.9[dB]
X_pulse	= 2.71666667[us]
Irr_atn_dec	= 22.445[dB]
Irr_atn_noe	= 22.445[dB]
Irr_noise	= WALTZ
Decoupling	= TRUE
Initial_wait	= 1[s]
Noe	= TRUE
Noe_time	= 2[s]
Recvr_gain	= 60
Relaxation_delay	= 2[s]
Repetition_time	= 3.06430464[s]
Temp_get	= 20.4[dC]

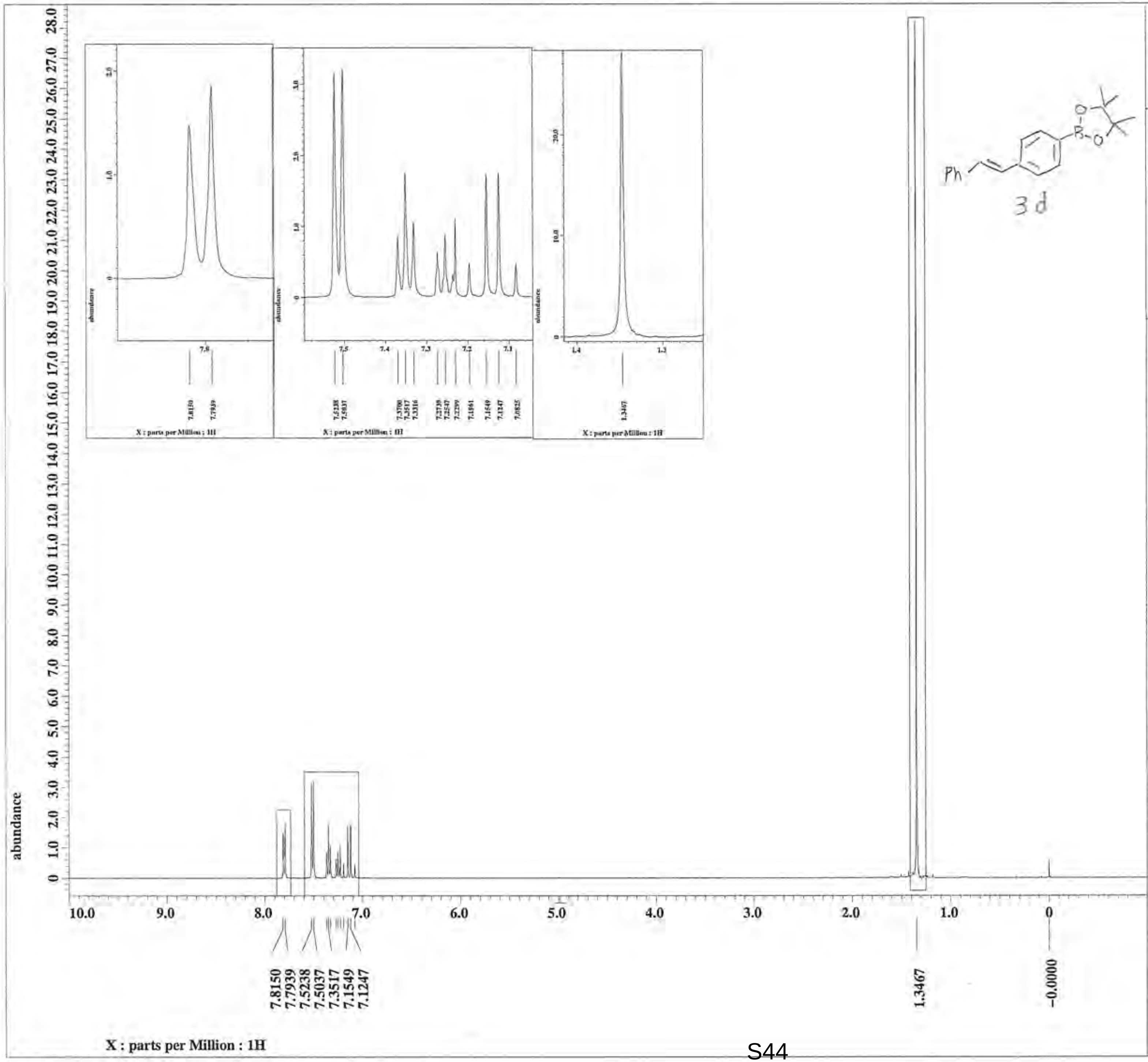

---- PROCESSING PARAMETERS ----
dc_balance : 0 : FALSE
sexp : 0.2[Hz] : 0.0[s]
trapezoid3 : 0[%] : 80[%] : 100[%]
zerofill : 1
fft : 1 : TRUE : TRUE
machinephase
ppm

Derived from: UKI-127-A proton-1.jdf

Filename = UKI-127-A proton-4.jd
Author = element
Experiment = single_pulse.ex2
Sample_id = 127
Solvent = CHLOROFORM-D
Creation_time = 2-DEC-2013 17:46:18
Revision_time = 2-DEC-2013 19:03:26
Current_time = 2-DEC-2013 19:03:28

Comment = single_pulse
Data_format = 1D COMPLEX
Dim_size = 16384
Dim_title = 1H
Dim_units = [ppm]
Dimensions = X
Site = ECS 400
Spectrometer = JNM-ECS400

Field_strength = 9.20197068[T] (390[MH
X_acq_duration = 2.78790144[s]
X_domain = 1H
X_freq = 391.78655441[MHz]
X_offset = 5[ppm]
X_points = 16384
X_prescans = 1
X_resolution = 0.35869274[Hz]
X_sweep = 5.87682181[kHz]
Irr_domain = 1H
Irr_freq = 391.78655441[MHz]
Irr_offset = 5[ppm]
Tri_domain = 1H
Tri_freq = 391.78655441[MHz]
Tri_offset = 5[ppm]
Clipped = FALSE
Mod_return = 1
Scans = 8
Total_scans = 8

X_90_width = 10.8[us]
X_acq_time = 2.78790144[s]
X_angle = 45[deg]
X_atn = 1.9[dB]
X_pulse = 5.4[us]
Irr_mode = Off
Tri_mode = Off
Dante_presat = FALSE
Initial_wait = 1[s]
Recvr_gain = 30
Relaxation_delay = 5[s]
Repetition_time = 7.78790144[s]
Temp_get = 20.4[dC]

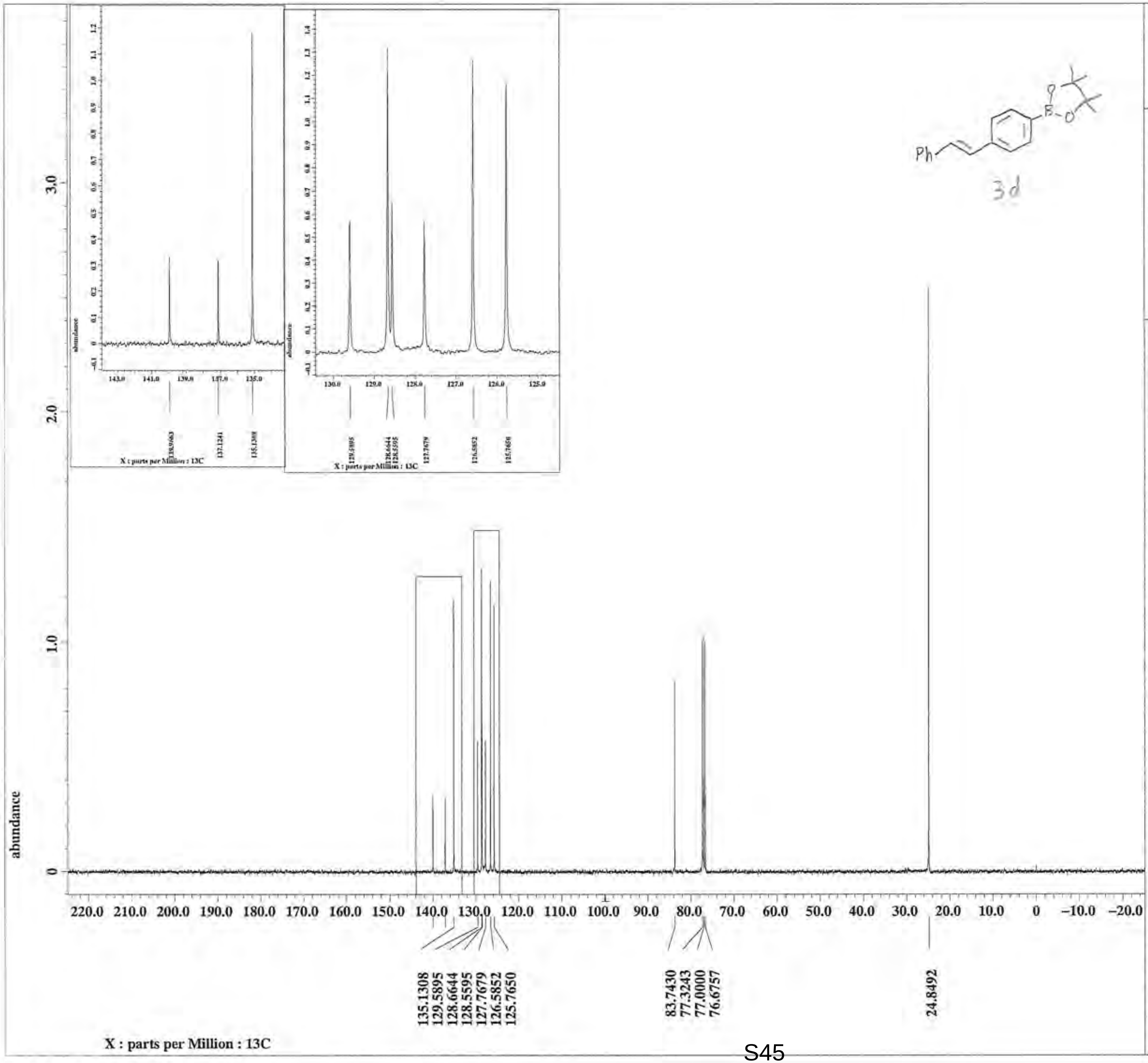


```
---- PROCESSING PARAMETERS ----
dc_balance : 0 : FALSE
sexp : 2.0[Hz] : 0.0[s]
trapezoid3 : 0[%] : 80[%] : 100[%]
zerofill : 1
fft : 1 : TRUE : TRUE
machinephase
ppm

Derived from: UKI-127-B carbon-1.jdf

Filename          = UKI-127-B carbon-3.jd
Author            = element
Experiment        = single_pulse_dec
Sample_id         = 127
Solvent           = CHLOROFORM-D
Creation_time     =  2-DEC-2013 17:57:44
Revision_time     =  2-DEC-2013 19:06:09
Current_time      =  2-DEC-2013 19:27:18

Comment           = single pulse decouple
Data_format       = 1D COMPLEX
Dim_size          = 26214
Dim_title         = 13C
Dim_units         = [ppm]
Dimensions        = X
Site              = ECS 400
Spectrometer      = JNM-ECS400

Field_strength    = 9.20197068[T] (390[MH
X_acq_duration    = 1.06430464[s]
X_domain          = 13C
X_freq            = 98.51479726[MHz]
X_offset          = 100[ppm]
X_points          = 32768
X_prescans        = 4
X_resolution      = 0.93958061[Hz]
X_sweep           = 30.78817734[kHz]
Irr_domain        = 1H
Irr_freq          = 391.78655441[MHz]
Irr_offset        = 5[ppm]
Clipped           = FALSE
Mod_return        = 1
Scans             = 200
Total_scans       = 200

X_90_width        = 8.15[us]
X_acq_time        = 1.06430464[s]
X_angle           = 30[deg]
X_atn             = 4.9[dB]
X_pulse           = 2.71666667[us]
Irr_atn_dec       = 22.445[dB]
Irr_atn_noe       = 22.445[dB]
Irr_noise         = WALTZ
Decoupling        = TRUE
Initial_wait      = 1[s]
Noe               = TRUE
Noe_time          = 2[s]
Recvr_gain        = 60
Relaxation_delay  = 2[s]
Repetition_time   = 3.06430464[s]
Temp_get          = 20.3[dC]
```

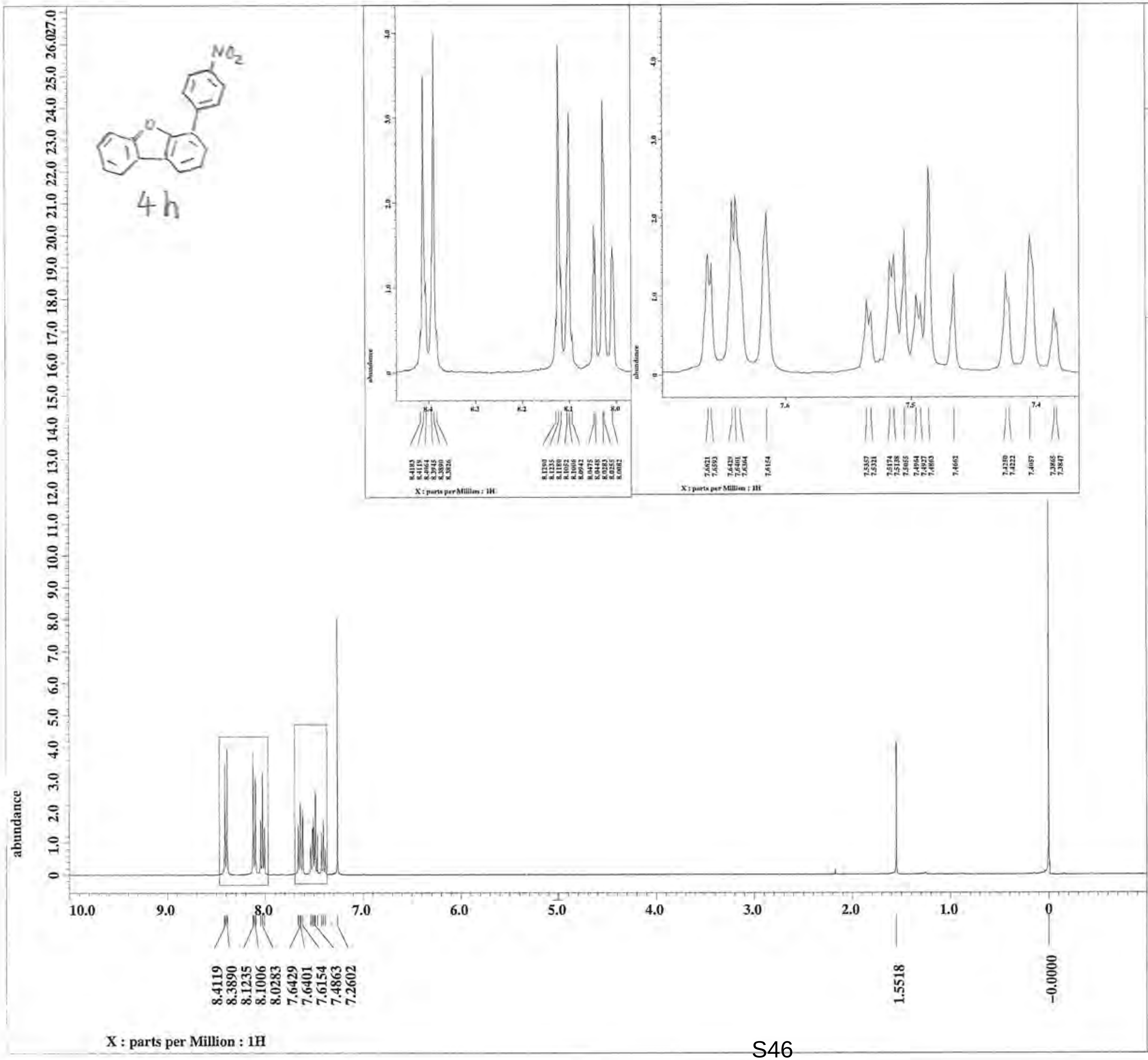

---- PROCESSING PARAMETERS ----
dc_balance : 0 : FALSE
sexp : 0.2[Hz] : 0.0[s]
trapezoid3 : 0[%] : 80[%] : 100[%]
zerofill : 1
fft : 1 : TRUE : TRUE
machinephase
ppm

Derived from: UKI-186-A proton-1.jdf

Filename	= UKI-186-A proton-4.jd
Author	= element
Experiment	= single_pulse.ex2
Sample_id	= 186
Solvent	= CHLOROFORM-D
Creation_time	= 27-JUN-2014 13:34:02
Revision_time	= 11-SEP-2014 16:10:35
Current_time	= 11-SEP-2014 16:10:39
Comment	= single_pulse
Data_format	= 1D COMPLEX
Dim_size	= 16384
Dim_title	= 1H
Dim_units	= [ppm]
Dimensions	= X
Site	= ECS 400
Spectrometer	= JNM-ECS400
Field_strength	= 9.20197068[T] (390[MH
X_acq_duration	= 2.78790144[s]
X_domain	= 1H
X_freq	= 391.78655441[MHz]
X_offset	= 5[ppm]
X_points	= 16384
X_prescans	= 1
X_resolution	= 0.35869274[Hz]
X_sweep	= 5.87682181[kHz]
Irr_domain	= 1H
Irr_freq	= 391.78655441[MHz]
Irr_offset	= 5[ppm]
Tri_domain	= 1H
Tri_freq	= 391.78655441[MHz]
Tri_offset	= 5[ppm]
Clipped	= FALSE
Mod_return	= 1
Scans	= 15
Total_scans	= 15
X_90_width	= 10.7[us]
X_acq_time	= 2.78790144[s]
X_angle	= 45[deg]
X_atn	= 1.9[dB]
X_pulse	= 5.35[us]
Irr_mode	= Off
Tri_mode	= Off
Dante_presat	= FALSE
Initial_wait	= 1[s]
Recvr_gain	= 56
Relaxation_delay	= 4[s]
Repetition_time	= 6.78790144[s]
Temp_get	= 20.2[dC]

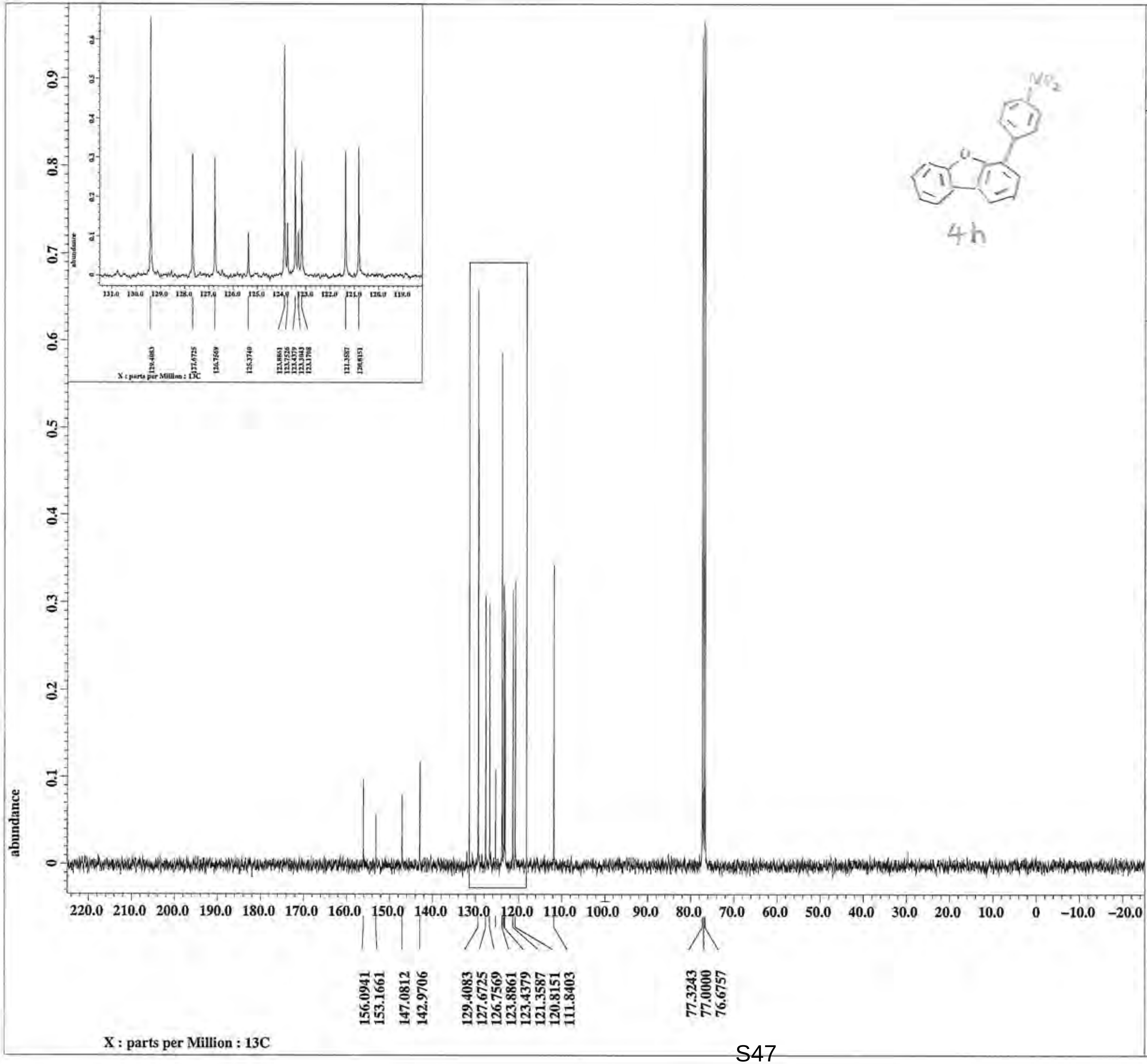

4h

---- PROCESSING PARAMETERS ----
dc_balance : 0 : FALSE
sexp : 2.0[Hz] : 0.0[s]
trapezoid3 : 0[%] : 80[%] : 100[%]
zerofill : 1
fft : 1 : TRUE : TRUE
machinephase
ppm

Derived from: UKI-186-B2 carbon-1.jdf

Filename	= UKI-186-B2 carbon-3.j
Author	= element
Experiment	= single_pulse_dec
Sample_id	= S#627309
Solvent	= CHLOROFORM-D
Creation_time	= 24-OCT-2014 17:24:38
Revision_time	= 24-OCT-2014 17:47:55
Current_time	= 24-OCT-2014 17:51:35
Comment	= single pulse decouple
Data_format	= 1D COMPLEX
Dim_size	= 26214
Dim_title	= 13C
Dim_units	= [ppm]
Dimensions	= X
Site	= ECS 400
Spectrometer	= JNM-ECS400
Field_strength	= 9.20197068[T] (390[MH
X_acq_duration	= 1.06430464[s]
X_domain	= 13C
X_freq	= 98.51479726[MHz]
X_offset	= 100[ppm]
X_points	= 32768
X_prescans	= 4
X_resolution	= 0.93958061[Hz]
X_sweep	= 30.78817734[kHz]
Irr_domain	= 1H
Irr_freq	= 391.78655441[MHz]
Irr_offset	= 5[ppm]
Clipped	= FALSE
Mod_return	= 1
Scans	= 300
Total_scans	= 300
X_90_width	= 8.8[us]
X_acq_time	= 1.06430464[s]
X_angle	= 30[deg]
X_atn	= 4.9[dB]
X_pulse	= 2.93333333[us]
Irr_atn_dec	= 22.52628[dB]
Irr_atn_noe	= 22.52628[dB]
Irr_noise	= WALTZ
Decoupling	= TRUE
Initial_wait	= 1[s]
Noe	= TRUE
Noe_time	= 2[s]
Recvr_gain	= 60
Relaxation_delay	= 2[s]
Repetition_time	= 3.06430464[s]
Temp_get	= 22[dC]

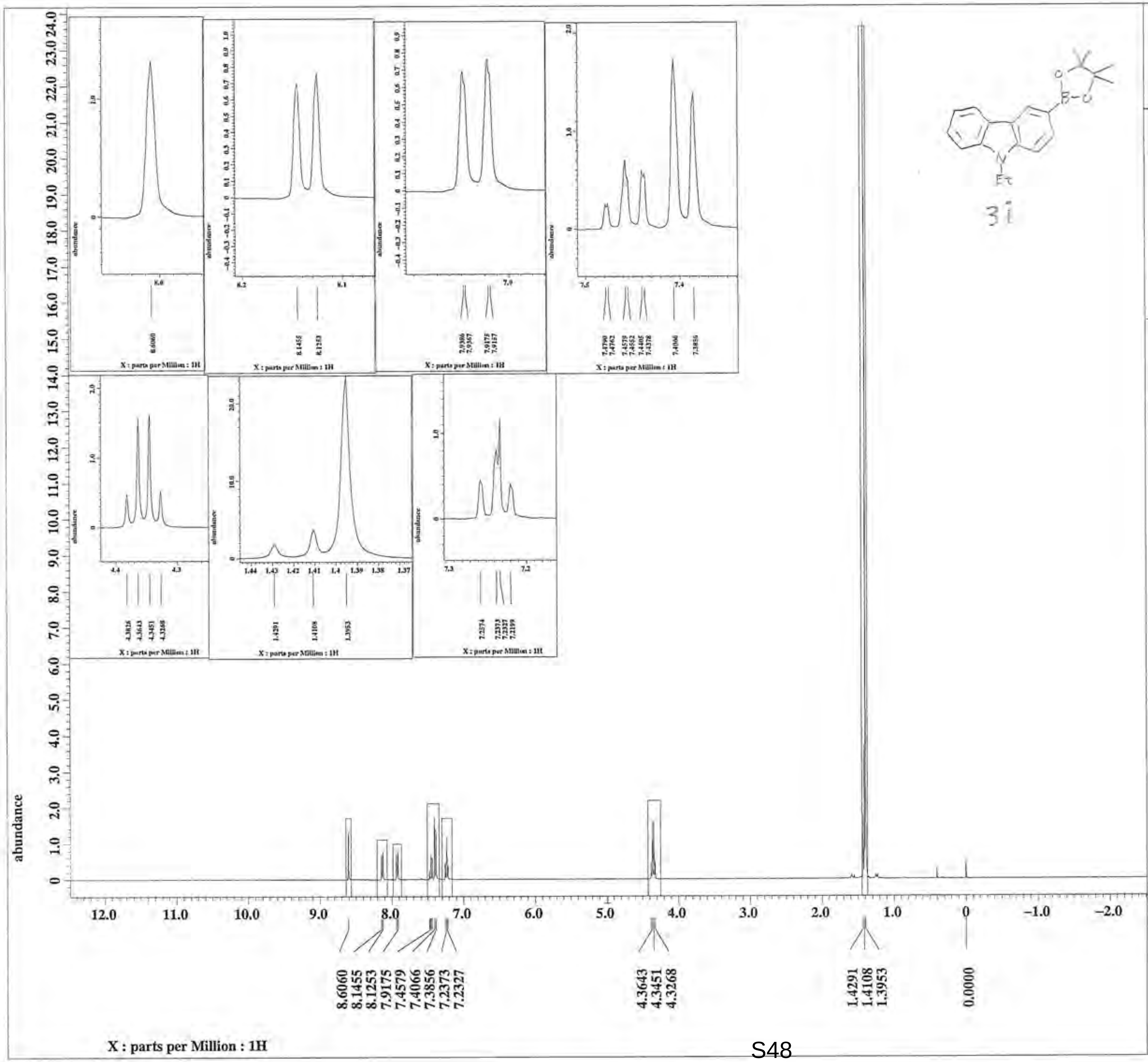

JEOL

---- PROCESSING PARAMETERS ----
dc_balance : 0 : FALSE
sexp : 0.2[Hz] : 0.0[s]
trapezoid3 : 0[%] : 80[%] : 100[%]
zerofill : 1
fft : 1 : TRUE : TRUE
machinephase
ppm

Derived from: UKI-123-A proton-1.jdf

Filename	= UKI-123-A proton-5.jd
Author	= element
Experiment	= single_pulse.ex2
Sample_id	= 123
Solvent	= CHLOROFORM-D
Creation_time	= 21-NOV-2013 20:52:25
Revision_time	= 1-FEB-2014 09:49:32
Current_time	= 1-FEB-2014 09:49:37
Comment	= single_pulse
Data_format	= 1D COMPLEX
Dim_size	= 16384
Dim_title	= 1H
Dim_units	= [ppm]
Dimensions	= X
Site	= ECS 400
Spectrometer	= JNM-ECS400
Field_strength	= 9.20197068[T] (390[MH
X_acq_duration	= 2.78790144[s]
X_domain	= 1H
X_freq	= 391.78655441[MHz]
X_offset	= 5[ppm]
X_points	= 16384
X_prescans	= 1
X_resolution	= 0.35869274[Hz]
X_sweep	= 5.87682181[kHz]
Irr_domain	= 1H
Irr_freq	= 391.78655441[MHz]
Irr_offset	= 5[ppm]
Tri_domain	= 1H
Tri_freq	= 391.78655441[MHz]
Tri_offset	= 5[ppm]
Clipped	= FALSE
Mod_return	= 1
Scans	= 8
Total_scans	= 8
X_90_width	= 10.8[us]
X_acq_time	= 2.78790144[s]
X_angle	= 45[deg]
X_atn	= 1.9[dB]
X_pulse	= 5.4[us]
Irr_mode	= Off
Tri_mode	= Off
Dante_presat	= FALSE
Initial_wait	= 1[s]
Recvr_gain	= 32
Relaxation_delay	= 5[s]
Repetition_time	= 7.78790144[s]
Temp_get	= 20.5[dC]

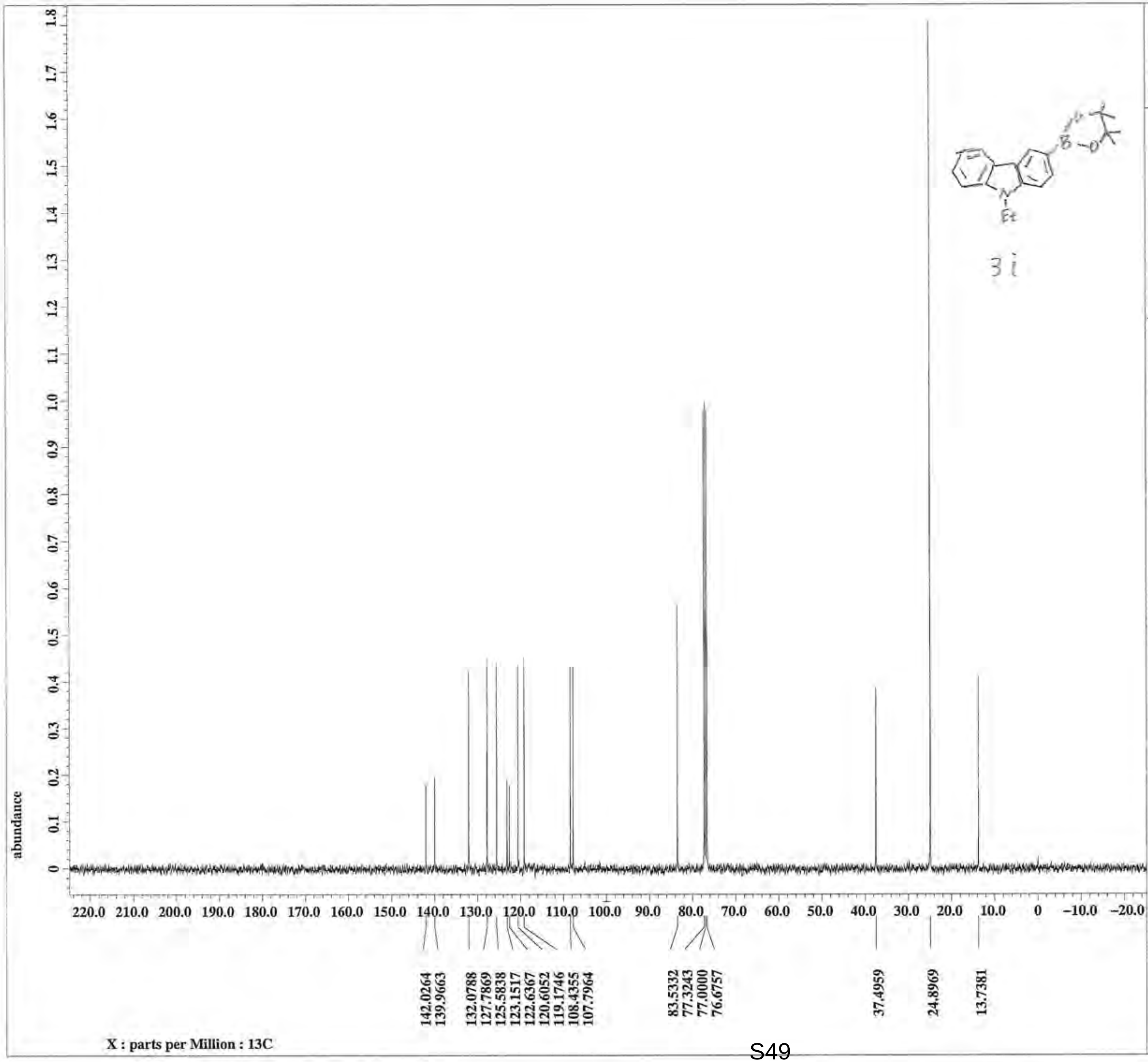


```
---- PROCESSING PARAMETERS ----
dc_balance : 0 : FALSE
sexp : 2.0[Hz] : 0.0[s]
trapezoid3 : 0[%] : 80[%] : 100[%]
zerofill : 1
fft : 1 : TRUE : TRUE
machinephase
ppm

Derived from: UKI-123-B carbon-1.jdf
```

```
Filename           = UKI-123-B carbon-3.jd
Author             = element
Experiment         = single_pulse_dec
Sample_id          = 123
Solvent            = CHLOROFORM-D
Creation_time      = 21-NOV-2013 21:03:52
Revision_time      = 21-NOV-2013 22:21:19
Current_time       = 21-NOV-2013 22:21:35

Comment            = single pulse decouple
Data_format        = 1D COMPLEX
Dim_size           = 26214
Dim_title          = 13C
Dim_units          = [ppm]
Dimensions         = X
Site               = ECS 400
Spectrometer       = JNM-ECS400

Field_strength     = 9.20197068[T] (390[MH
X_acq_duration     = 1.06430464[s]
X_domain           = 13C
X_freq             = 98.51479726[MHz]
X_offset           = 100[ppm]
X_points           = 32768
X_prescans         = 4
X_resolution       = 0.93958061[Hz]
X_sweep            = 30.78817734[kHz]
Irr_domain         = 1H
Irr_freq           = 391.78655441[MHz]
Irr_offset         = 5[ppm]
Clipped            = FALSE
Mod_return         = 1
Scans              = 200
Total_scans        = 200

X_90_width         = 8.15[us]
X_acq_time         = 1.06430464[s]
X_angle            = 30[deg]
X_atn              = 4.9[dB]
X_pulse            = 2.71666667[us]
Irr_atn_dec        = 22.445[dB]
Irr_atn_noe        = 22.445[dB]
Irr_noise          = WALTZ
Decoupling         = TRUE
Initial_wait       = 1[s]
Noe                = TRUE
Noe_time           = 2[s]
Recvr_gain         = 60
Relaxation_delay   = 2[s]
Repetition_time    = 3.06430464[s]
Temp_get           = 20.2[dC]
```

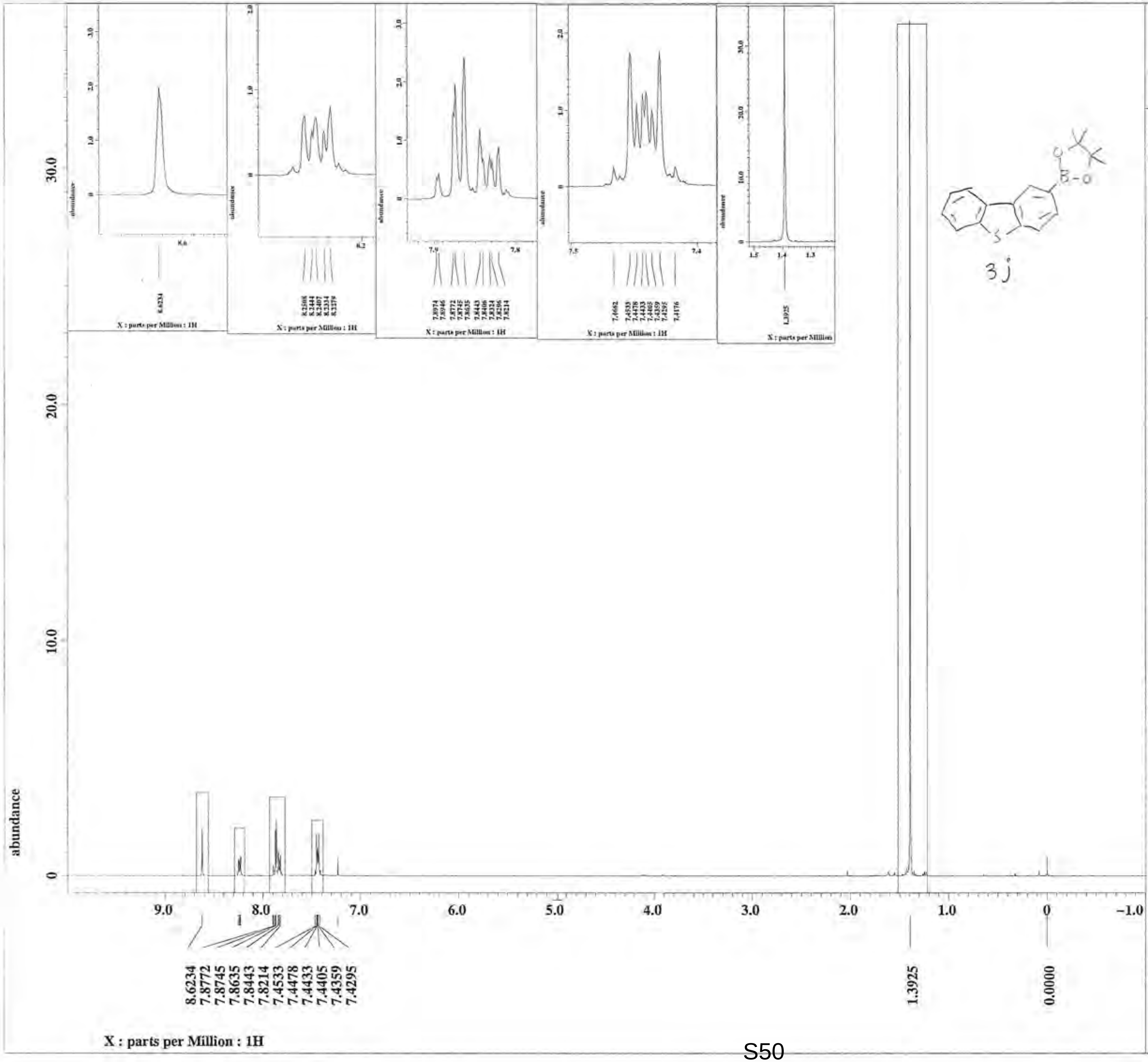

---- PROCESSING PARAMETERS ----
dc_balance : 0 : FALSE
sexp : 0.2[Hz] : 0.0[s]
trapezoid3 : 0[%] : 80[%] : 100[%]
zerofill : 1
fft : 1 : TRUE : TRUE
machinephase
ppm

Derived from: UKI-195-A proton-1.jdf

Filename	= UKI-195-A proton-5.jd
Author	= element
Experiment	= single_pulse.ex2
Sample_id	= 195
Solvent	= CHLOROFORM-D
Creation_time	= 22-JUL-2014 15:29:33
Revision_time	= 22-JUL-2014 15:43:51
Current_time	= 22-JUL-2014 15:43:54
Comment	= single_pulse
Data_format	= 1D COMPLEX
Dim_size	= 16384
Dim_title	= 1H
Dim_units	= [ppm]
Dimensions	= X
Site	= ECS 400
Spectrometer	= JNM-ECS400
Field_strength	= 9.20197068[T] (390[MH
X_acq_duration	= 2.78790144[s]
X_domain	= 1H
X_freq	= 391.78655441[MHz]
X_offset	= 5[ppm]
X_points	= 16384
X_prescans	= 1
X_resolution	= 0.35869274[Hz]
X_sweep	= 5.87682181[kHz]
Irr_domain	= 1H
Irr_freq	= 391.78655441[MHz]
Irr_offset	= 5[ppm]
Tri_domain	= 1H
Tri_freq	= 391.78655441[MHz]
Tri_offset	= 5[ppm]
Clipped	= FALSE
Mod_return	= 1
Scans	= 15
Total_scans	= 15
X_90_width	= 10.7[us]
X_acq_time	= 2.78790144[s]
X_angle	= 45[deg]
X_atn	= 1.9[dB]
X_pulse	= 5.35[us]
Irr_mode	= Off
Tri_mode	= Off
Dante_presat	= FALSE
Initial_wait	= 1[s]
Recvr_gain	= 32
Relaxation_delay	= 4[s]
Repetition_time	= 6.78790144[s]
Temp_get	= 20.7[dC]

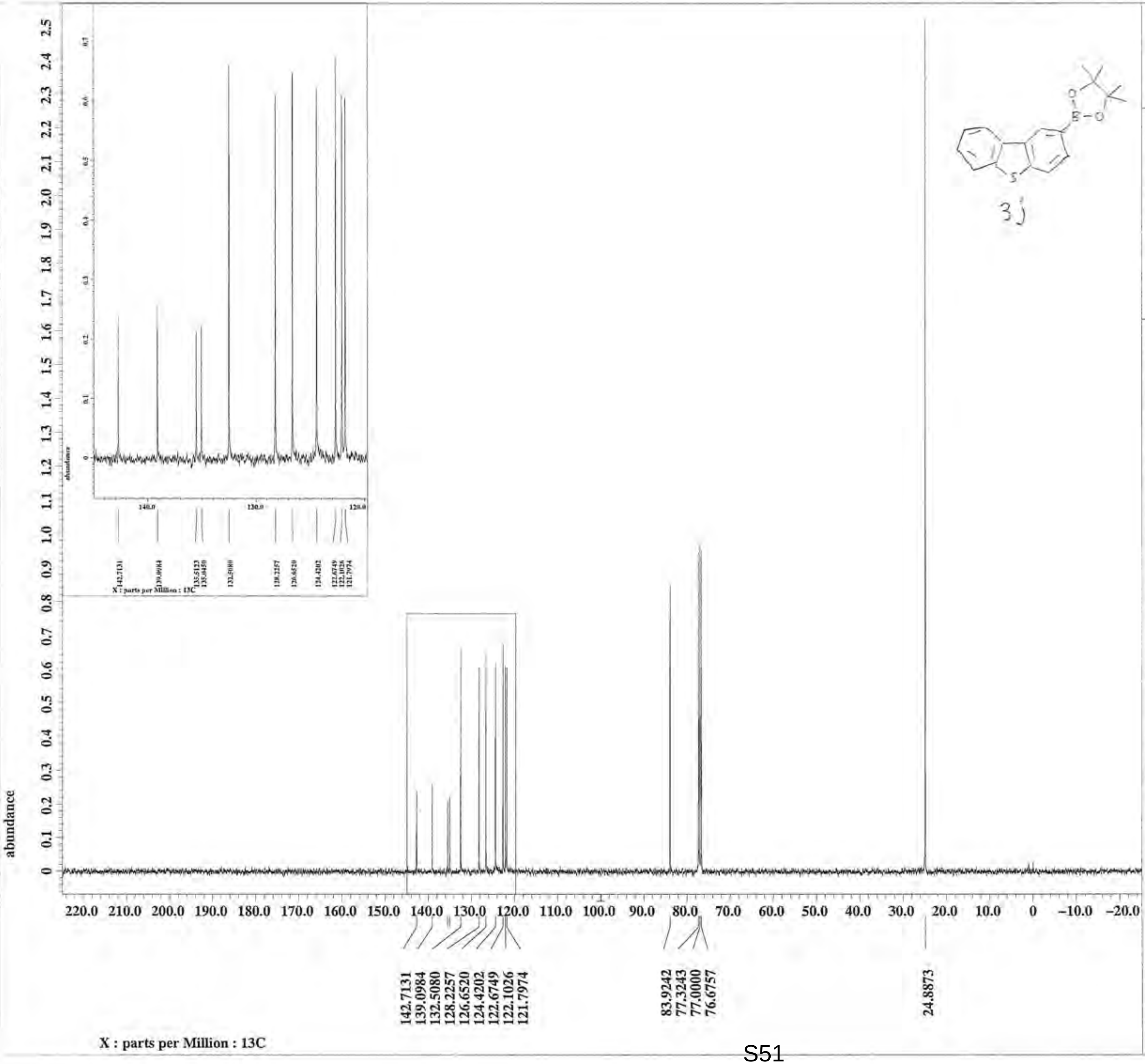

JEOL

---- PROCESSING PARAMETERS ----
dc_balance : 0 : FALSE
sexp : 2.0[Hz] : 0.0[s]
trapezoid3 : 0[%] : 80[%] : 100[%]
zerofill : 1
fft : 1 : TRUE : TRUE
machinephase
ppm

Derived from: UKI-195-B carbon-1.jdf

Filename	= UKI-195-B carbon-3.jd
Author	= element
Experiment	= single_pulse_dec
Sample_id	= 195
Solvent	= CHLOROFORM-D
Creation_time	= 22-JUL-2014 15:41:00
Revision_time	= 22-JUL-2014 15:50:48
Current_time	= 22-JUL-2014 15:51:18
Comment	= single pulse decouple
Data_format	= 1D COMPLEX
Dim_size	= 26214
Dim_title	= 13C
Dim_units	= [ppm]
Dimensions	= X
Site	= ECS 400
Spectrometer	= JNM-ECS400
Field_strength	= 9.20197068[T] (390[MH
X_acq_duration	= 1.06430464[s]
X_domain	= 13C
X_freq	= 98.51479726[MHz]
X_offset	= 100[ppm]
X_points	= 32768
X_prescans	= 4
X_resolution	= 0.93958061[Hz]
X_sweep	= 30.78817734[kHz]
Irr_domain	= 1H
Irr_freq	= 391.78655441[MHz]
Irr_offset	= 5[ppm]
Clipped	= FALSE
Mod_return	= 1
Scans	= 200
Total_scans	= 200
X_90_width	= 8.8[us]
X_acq_time	= 1.06430464[s]
X_angle	= 30[deg]
X_atn	= 4.9[dB]
X_pulse	= 2.93333333[us]
Irr_atn_dec	= 22.52628[dB]
Irr_atn_noe	= 22.52628[dB]
Irr_noise	= WALTZ
Decoupling	= TRUE
Initial_wait	= 1[s]
Noe	= TRUE
Noe_time	= 2[s]
Recvr_gain	= 60
Relaxation_delay	= 2[s]
Repetition_time	= 3.06430464[s]
Temp_get	= 21[dC]

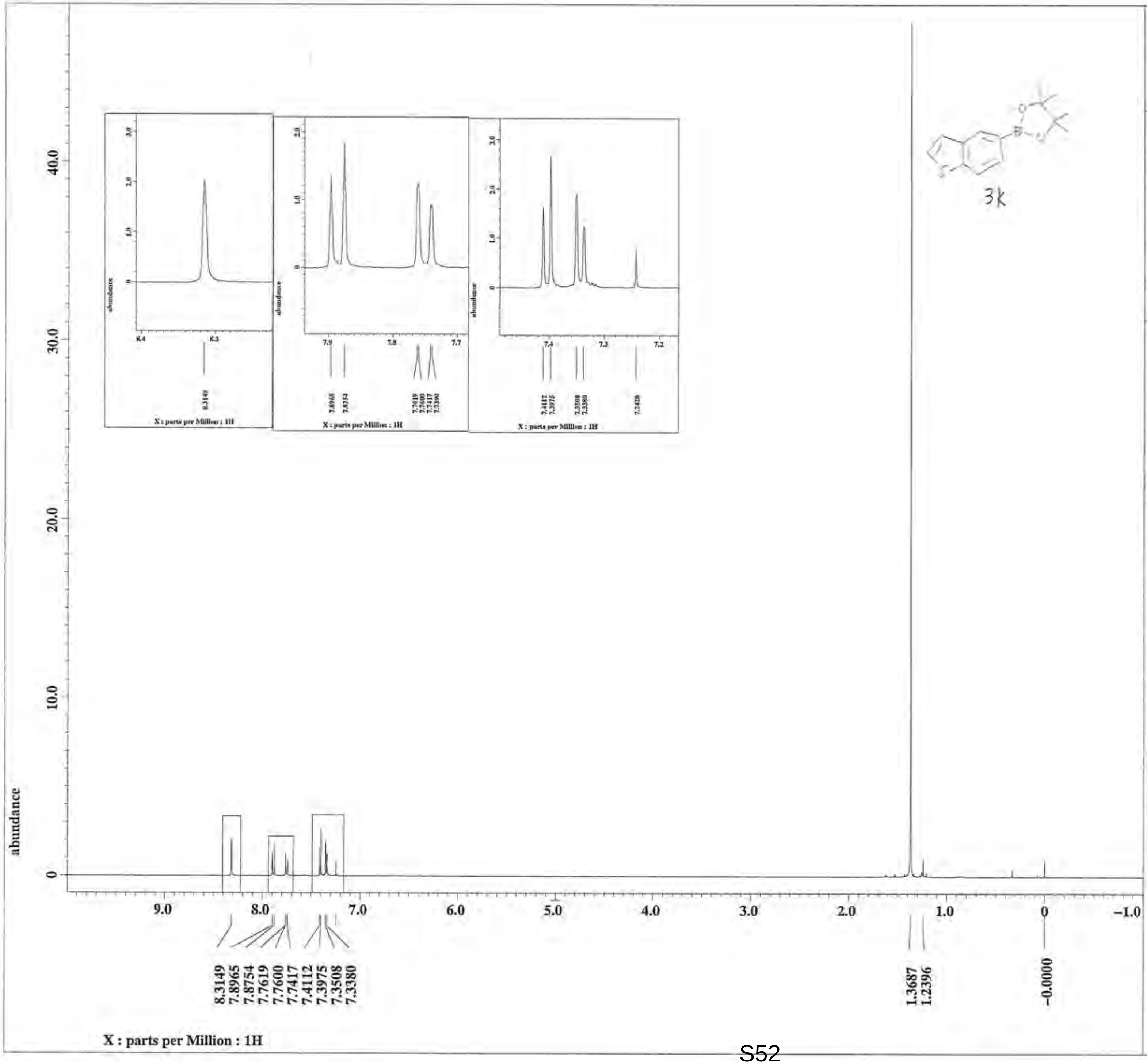


```
---- PROCESSING PARAMETERS ----
dc_balance : 0 : FALSE
sexp : 0.2[Hz] : 0.0[s]
trapezoid3 : 0[%] : 80[%] : 100[%]
zerofill : 1
fft : 1 : TRUE : TRUE
machinephase
ppm

Derived from: UKI-096-A proton-1.jdf

Filename          = UKI-096-A proton-6.jd
Author            = element
Experiment        = single_pulse.ex2
Sample_id         = 096
Solvent           = CHLOROFORM-D
Creation_time     =  2-NOV-2013 12:50:46
Revision_time     =  2-NOV-2013 14:09:19
Current_time      =  2-NOV-2013 14:09:23

Comment           = single_pulse
Data_format       = 1D COMPLEX
Dim_size          = 16384
Dim_title         = 1H
Dim_units         = [ppm]
Dimensions        = X
Site              = ECS 400
Spectrometer      = JNM-ECS400

Field_strength    = 9.20197068[T] (390[MH
X_acq_duration    = 2.78790144[s]
X_domain          = 1H
X_freq            = 391.78655441[MHz]
X_offset          = 5[ppm]
X_points          = 16384
X_prescans        = 1
X_resolution      = 0.35869274[Hz]
X_sweep           = 5.87682181[kHz]
Irr_domain        = 1H
Irr_freq          = 391.78655441[MHz]
Irr_offset        = 5[ppm]
Tri_domain        = 1H
Tri_freq          = 391.78655441[MHz]
Tri_offset        = 5[ppm]
Clipped           = FALSE
Mod_return        = 1
Scans             = 8
Total_scans       = 8

X_90_width        = 10.8[us]
X_acq_time        = 2.78790144[s]
X_angle           = 45[deg]
X_atn             = 1.9[dB]
X_pulse           = 5.4[us]
Irr_mode          = Off
Tri_mode          = Off
Dante_presat      = FALSE
Initial_wait      = 1[s]
Recvr_gain        = 32
Relaxation_delay  = 5[s]
Repetition_time   = 7.78790144[s]
Temp_get          = 20.4[dC]
```

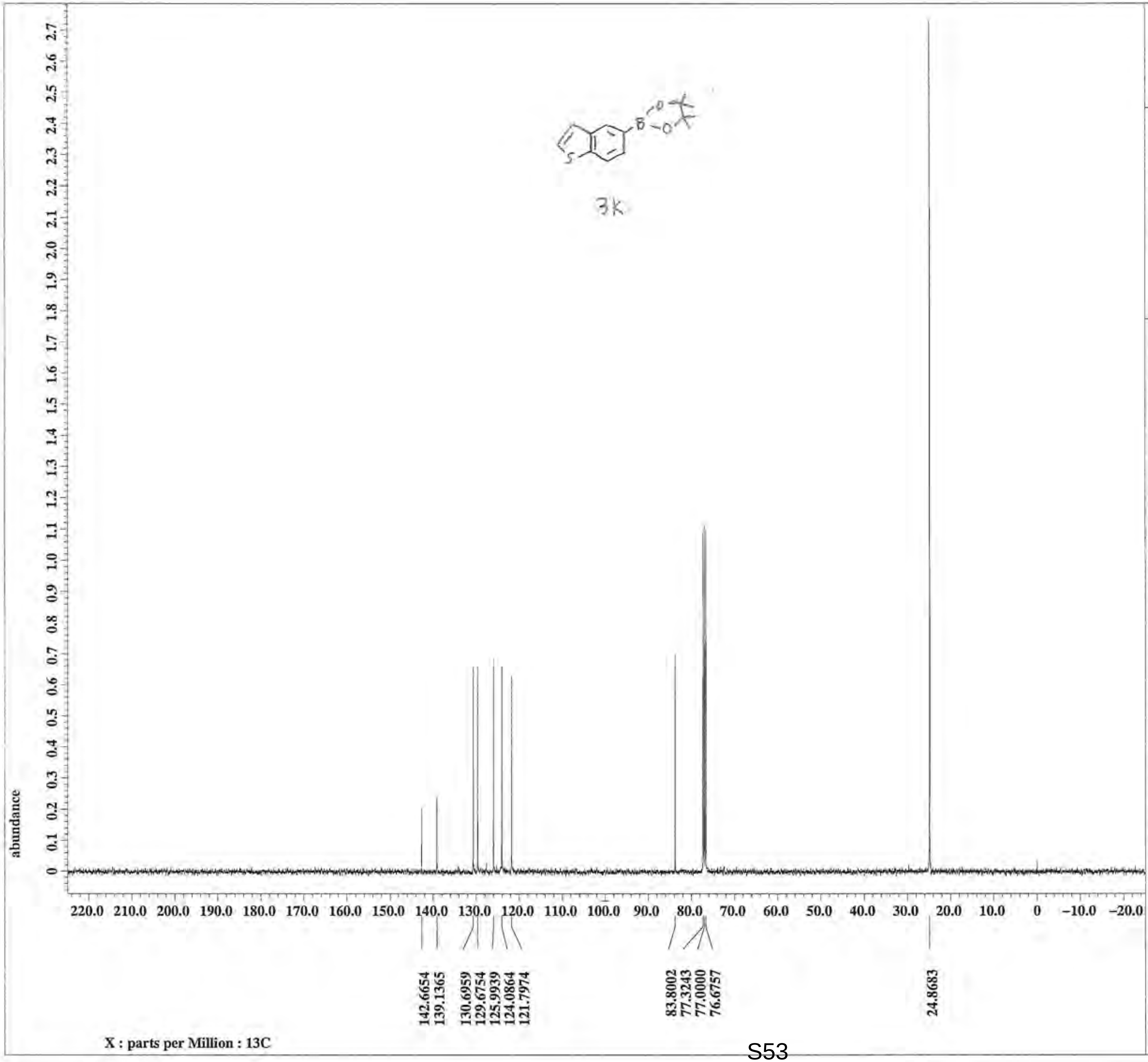


```
---- PROCESSING PARAMETERS ----
dc_balance : 0 : FALSE
sexp : 2.0[Hz] : 0.0[s]
trapezoid3 : 0[%] : 80[%] : 100[%]
zerofill : 1
fft : 1 : TRUE : TRUE
machinephase
ppm

Derived from: UKI-096-B carbon-1.jdf

Filename          = UKI-096-B carbon-3.jd
Author            = element
Experiment        = single_pulse_dec
Sample_id         = 096
Solvent           = CHLOROFORM-D
Creation_time     =  2-NOV-2013 13:01:31
Revision_time     =  2-NOV-2013 14:12:32
Current_time      =  2-NOV-2013 14:13:34

Comment           = single pulse decouple
Data_format       = 1D COMPLEX
Dim_size          = 26214
Dim_title         = 13C
Dim_units         = [ppm]
Dimensions        = X
Site              = ECS 400
Spectrometer      = JNM-ECS400

Field_strength    = 9.20197068[T] (390[MH
X_acq_duration    = 1.06430464[s]
X_domain          = 13C
X_freq            = 98.51479726[MHz]
X_offset          = 100[ppm]
X_points          = 32768
X_prescans        = 4
X_resolution      = 0.93958061[Hz]
X_sweep           = 30.78817734[kHz]
Irr_domain        = 1H
Irr_freq          = 391.78655441[MHz]
Irr_offset        = 5[ppm]
Clipped           = FALSE
Mod_return        = 1
Scans             = 200
Total_scans       = 200

X_90_width        = 8.15[us]
X_acq_time        = 1.06430464[s]
X_angle           = 30[deg]
X_atn             = 4.9[dB]
X_pulse           = 2.71666667[us]
Irr_atn_dec       = 22.445[dB]
Irr_atn_noe       = 22.445[dB]
Irr_noise         = WALTZ
Decoupling        = TRUE
Initial_wait      = 1[s]
Noe               = TRUE
Noe_time          = 2[s]
Recvr_gain        = 60
Relaxation_delay  = 2[s]
Repetition_time   = 3.06430464[s]
Temp_get          = 20.3[dC]
```

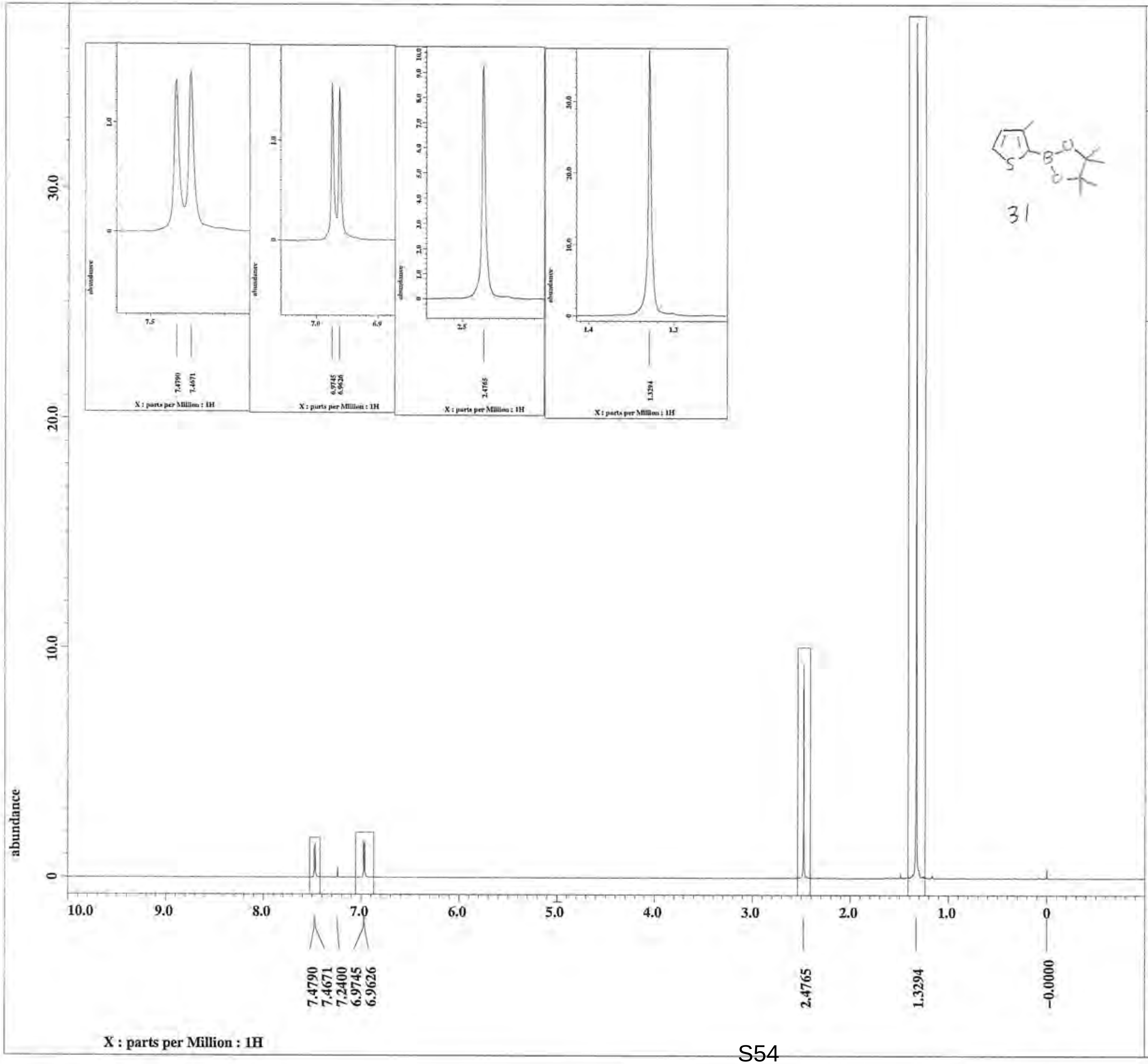

31

```
---- PROCESSING PARAMETERS ----
dc_balance : 0 : FALSE
sexp : 0.2[Hz] : 0.0[s]
trapezoid3 : 0[%] : 80[%] : 100[%]
zerofill : 1
fft : 1 : TRUE : TRUE
machinephase
ppm

Derived from: UKI-132-A proton-1.jdf
```

```
Filename          = UKI-132-A proton-12.j
Author            = element
Experiment        = single_pulse.ex2
Sample_id         = 132
Solvent           = CHLOROFORM-D
Creation_time     = 13-DEC-2013 17:17:21
Revision_time     = 13-DEC-2013 18:37:34
Current_time      = 13-DEC-2013 18:37:35

Comment           = single_pulse
Data_format       = 1D COMPLEX
Dim_size          = 16384
Dim_title         = 1H
Dim_units         = [ppm]
Dimensions        = X
Site              = ECS 400
Spectrometer      = JNM-ECS400

Field_strength    = 9.20197068[T] (390[MH
X_acq_duration    = 2.78790144[s]
X_domain          = 1H
X_freq            = 391.78655441[MHz]
X_offset          = 5[ppm]
X_points          = 16384
X_prescans        = 1
X_resolution      = 0.35869274[Hz]
X_sweep           = 5.87682181[kHz]
Irr_domain        = 1H
Irr_freq          = 391.78655441[MHz]
Irr_offset        = 5[ppm]
Tri_domain        = 1H
Tri_freq          = 391.78655441[MHz]
Tri_offset        = 5[ppm]
Clipped           = FALSE
Mod_return        = 1
Scans             = 8
Total_scans       = 8

X_90_width        = 10.8[us]
X_acq_time        = 2.78790144[s]
X_angle           = 45[deg]
X_atn             = 1.9[dB]
X_pulse           = 5.4[us]
Irr_mode          = Off
Tri_mode          = Off
Dante_presat      = FALSE
Initial_wait      = 1[s]
Recvr_gain        = 28
Relaxation_delay  = 5[s]
Repetition_time   = 7.78790144[s]
Temp_get          = 19.9[dC]
```

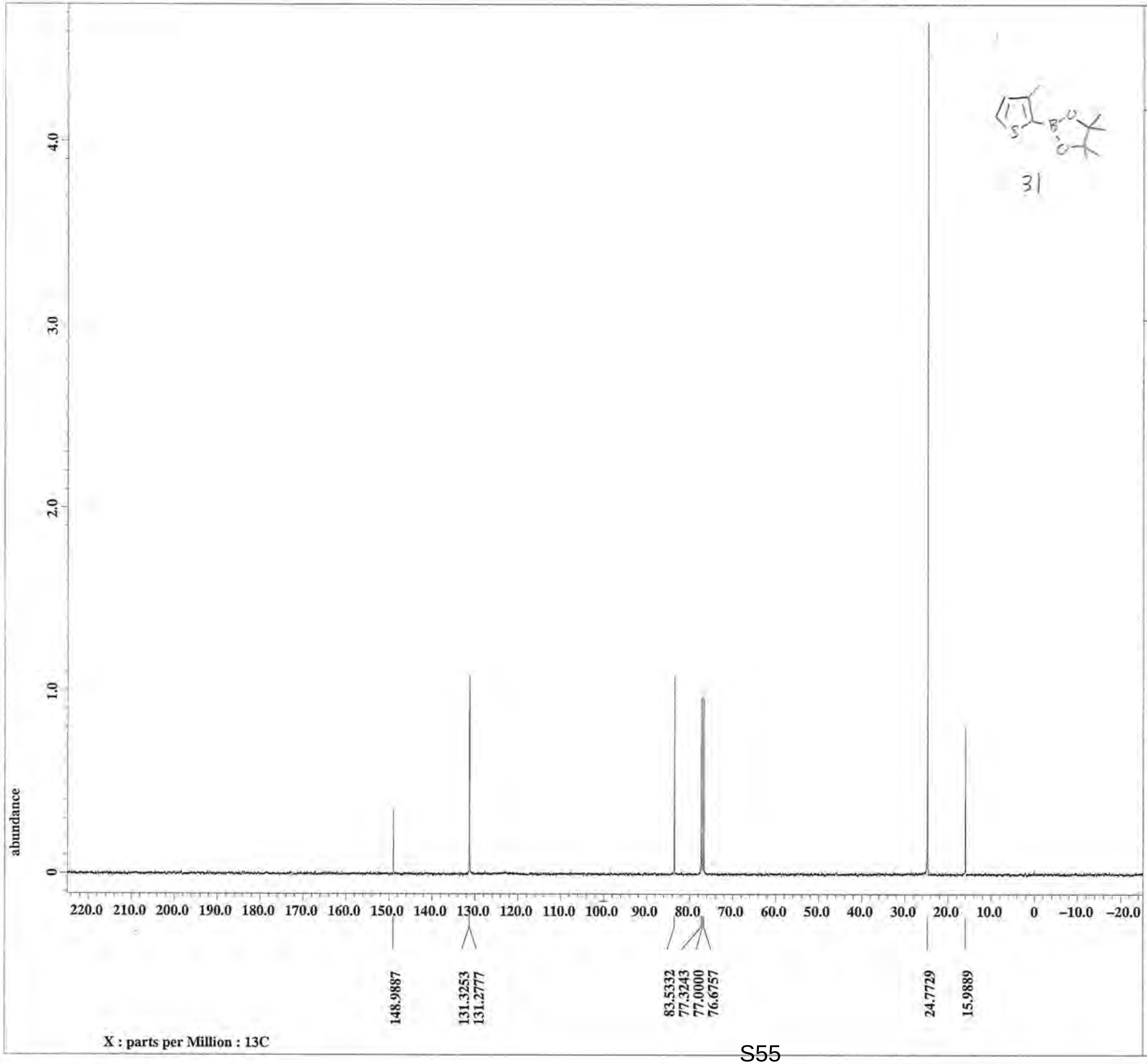

JEOL

```
---- PROCESSING PARAMETERS ----
dc_balance : 0 : FALSE
sexp : 2.0[Hz] : 0.0[s]
trapezoid3 : 0[%] : 80[%] : 100[%]
zerofill : 1
fft : 1 : TRUE : TRUE
machinephase
ppm

Derived from: UKI-132-B carbon-1.jdf

Filename          = UKI-132-B carbon-3.jd
Author            = element
Experiment        = single_pulse_dec
Sample_id         = 132
Solvent           = CHLOROFORM-D
Creation_time     = 13-DEC-2013 17:28:45
Revision_time     = 13-DEC-2013 18:39:00
Current_time      = 13-DEC-2013 18:39:23

Comment           = single pulse decouple
Data_format       = 1D COMPLEX
Dim_size          = 26214
Dim_title         = 13C
Dim_units         = [ppm]
Dimensions        = X
Site              = ECS 400
Spectrometer      = JNM-ECS400

Field_strength    = 9.20197068[T] (390[MH
X_acq_duration    = 1.06430464[s]
X_domain          = 13C
X_freq            = 98.51479726[MHz]
X_offset          = 100[ppm]
X_points          = 32768
X_prescans        = 4
X_resolution      = 0.93958061[Hz]
X_sweep           = 30.78817734[kHz]
Irr_domain        = 1H
Irr_freq          = 391.78655441[MHz]
Irr_offset        = 5[ppm]
Clipped           = FALSE
Mod_return        = 1
Scans             = 200
Total_scans       = 200

X_90_width        = 8.15[us]
X_acq_time        = 1.06430464[s]
X_angle           = 30[deg]
X_atn             = 4.9[dB]
X_pulse           = 2.71666667[us]
Irr_atn_dec       = 22.445[dB]
Irr_atn_noe       = 22.445[dB]
Irr_noise         = WALTZ
Decoupling        = TRUE
Initial_wait      = 1[s]
Noe               = TRUE
Noe_time          = 2[s]
Recvr_gain        = 60
Relaxation_delay  = 2[s]
Repetition_time   = 3.06430464[s]
Temp_get          = 20[dC]
```

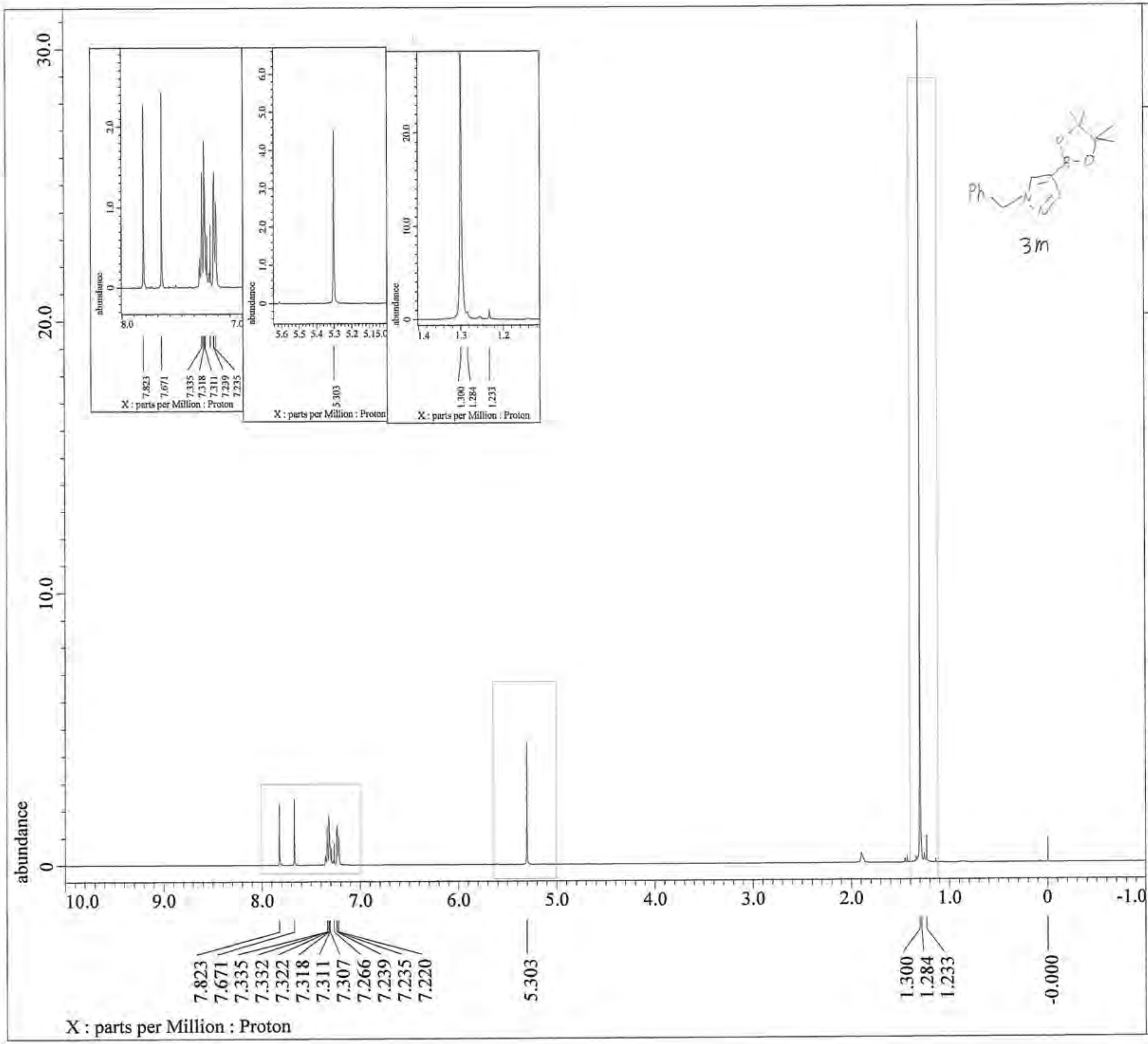

JEOL RESONANCE

```
---- PROCESSING PARAMETERS ----
dc_balance( 0, FALSE )
sexp( 0.2[Hz], 0.0[s] )
trapezoid( 0[%], 0[%], 80[%], 100[%] )
zerofill( 1 )
fft( 1, TRUE, TRUE )
machinephase
ppm

以下に由来:: UKI-111-2 H_Proton-1-1.jdf

Filename            = UKI-111-2 H_Proton-1-7.jdf
Author              = element
Experiment          = proton.jxp
Sample_Id           = UKI-111-2 H
Solvent             = CHLOROFORM-D
Creation_Time       =  7-NOV-2013 20:28:40
Revision_Time       =  7-NOV-2013 20:33:38
Current_Time        =  7-NOV-2013 20:33:44

Comment             = single_pulse
Data_Format         = 1D COMPLEX
Dim_Size            = 13107
Dim_Title           = Proton
Dim_Units           = [ppm]
Dimensions          = X
Site                = JNM-ECS400
Spectrometer        = DELTA2_NMR

Field_Strength      = 9.4073814[T] (400[MHz])
X_Acq_Duration      = 2.18103808[s]
X_Domain            = 1H
X_Freq              = 400.53219825[MHz]
X_Offset            = 5[ppm]
X_Points            = 16384
X_Prescans          = 1
X_Resolution        = 0.45849727[Hz]
X_Sweep             = 7.51201923[kHz]
X_Sweep_Clipped     = 6.00961538[kHz]
Irr_Domain          = Proton
Irr_Freq            = 400.53219825[MHz]
Irr_Offset          = 5[ppm]
Tri_Domain          = Proton
Tri_Freq            = 400.53219825[MHz]
Tri_Offset          = 5[ppm]
Clipped             = FALSE
Scans               = 8
Total_Scans         = 8

Relaxation_Delay    = 5[s]
Recvr_Gain          = 26
Temp_Get            = 20.1[dC]
X_90_Width          = 6.5[us]
X_Acq_Time          = 2.18103808[s]
X_Angle             = 45[deg]
X_Atn               = 0.8[dB]
X_Pulse             = 3.25[us]
Irr_Mode            = Off
Tri_Mode            = Off
```

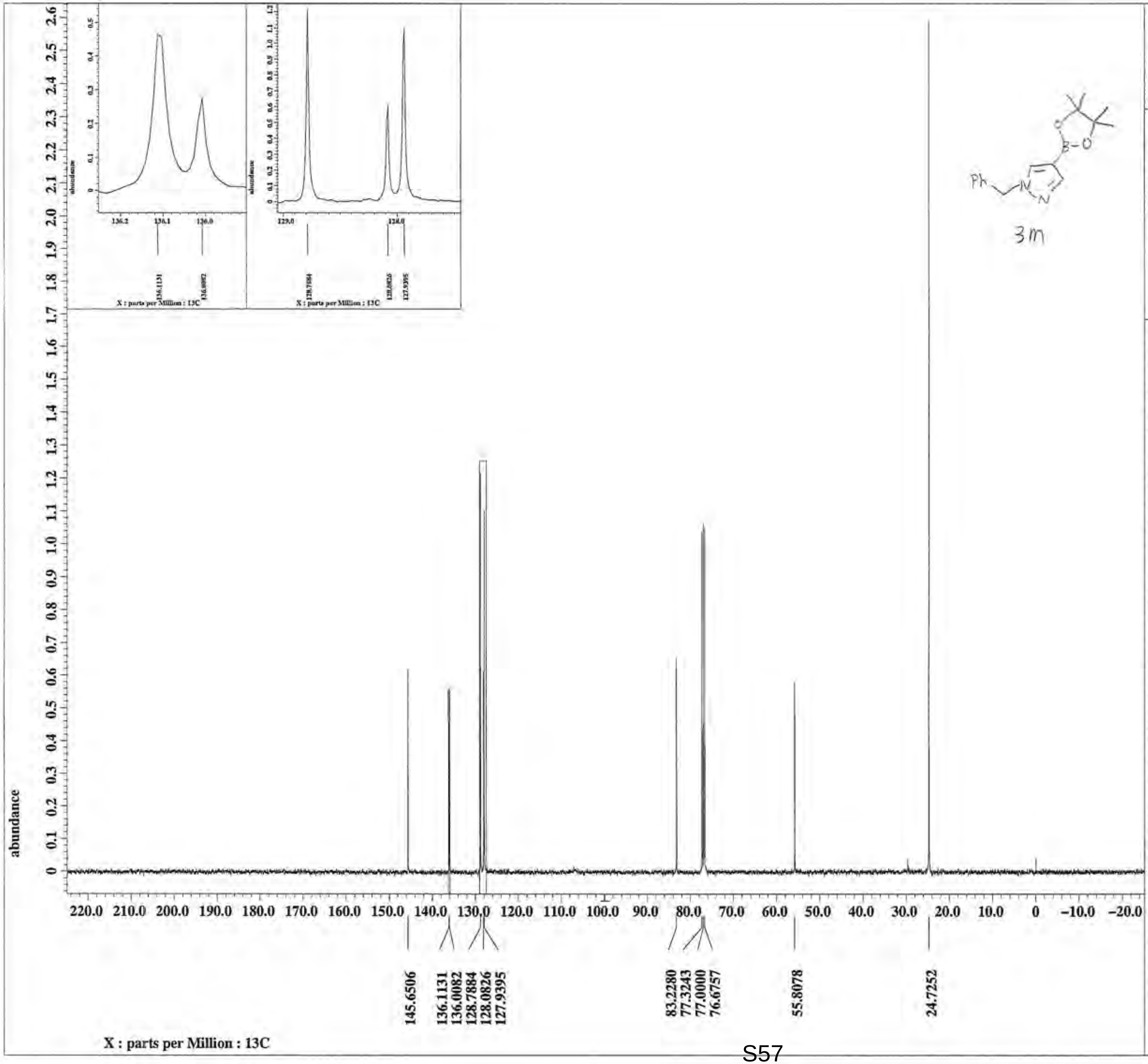


```
---- PROCESSING PARAMETERS ----
dc_balance : 0 : FALSE
sexp : 2.0[Hz] : 0.0[s]
trapezoid3 : 0[%] : 80[%] : 100[%]
zerofill : 1
fft : 1 : TRUE : TRUE
machinephase
ppm

Derived from: UKI-111-B-2 carbon-1.jdf

Filename          = UKI-111-B-2 carbon-4.
Author            = element
Experiment        = single_pulse_dec
Sample_id         = S#671564
Solvent           = CHLOROFORM-D
Creation_time     =  6-JAN-2015 18:28:11
Revision_time     =  6-JAN-2015 18:56:31
Current_time      =  6-JAN-2015 18:59:10

Comment           = single pulse decouple
Data_format       = 1D COMPLEX
Dim_size          = 26214
Dim_title         = 13C
Dim_units         = [ppm]
Dimensions        = X
Site              = ECS 400
Spectrometer      = JNM-ECS400

Field_strength    = 9.20197068[T] (390[MH
X_acq_duration    = 1.06430464[s]
X_domain          = 13C
X_freq            = 98.51479726[MHz]
X_offset          = 100[ppm]
X_points          = 32768
X_prescans        = 4
X_resolution      = 0.93958061[Hz]
X_sweep           = 30.78817734[kHz]
Irr_domain        = 1H
Irr_freq          = 391.78655441[MHz]
Irr_offset        = 5[ppm]
Clipped           = FALSE
Mod_return        = 1
Scans             = 300
Total_scans       = 300

X_90_width        = 8.8[us]
X_acq_time        = 1.06430464[s]
X_angle           = 30[deg]
X_atn             = 4.9[dB]
X_pulse           = 2.93333333[us]
Irr_atn_dec       = 22.52628[dB]
Irr_atn_noe       = 22.52628[dB]
Irr_noise         = WALTZ
Decoupling        = TRUE
Initial_wait      = 1[s]
Noe               = TRUE
Noe_time          = 2[s]
Recvr_gain        = 60
Relaxation_delay  = 2[s]
Repetition_time   = 3.06430464[s]
Temp_get          = 19.8[dC]
```

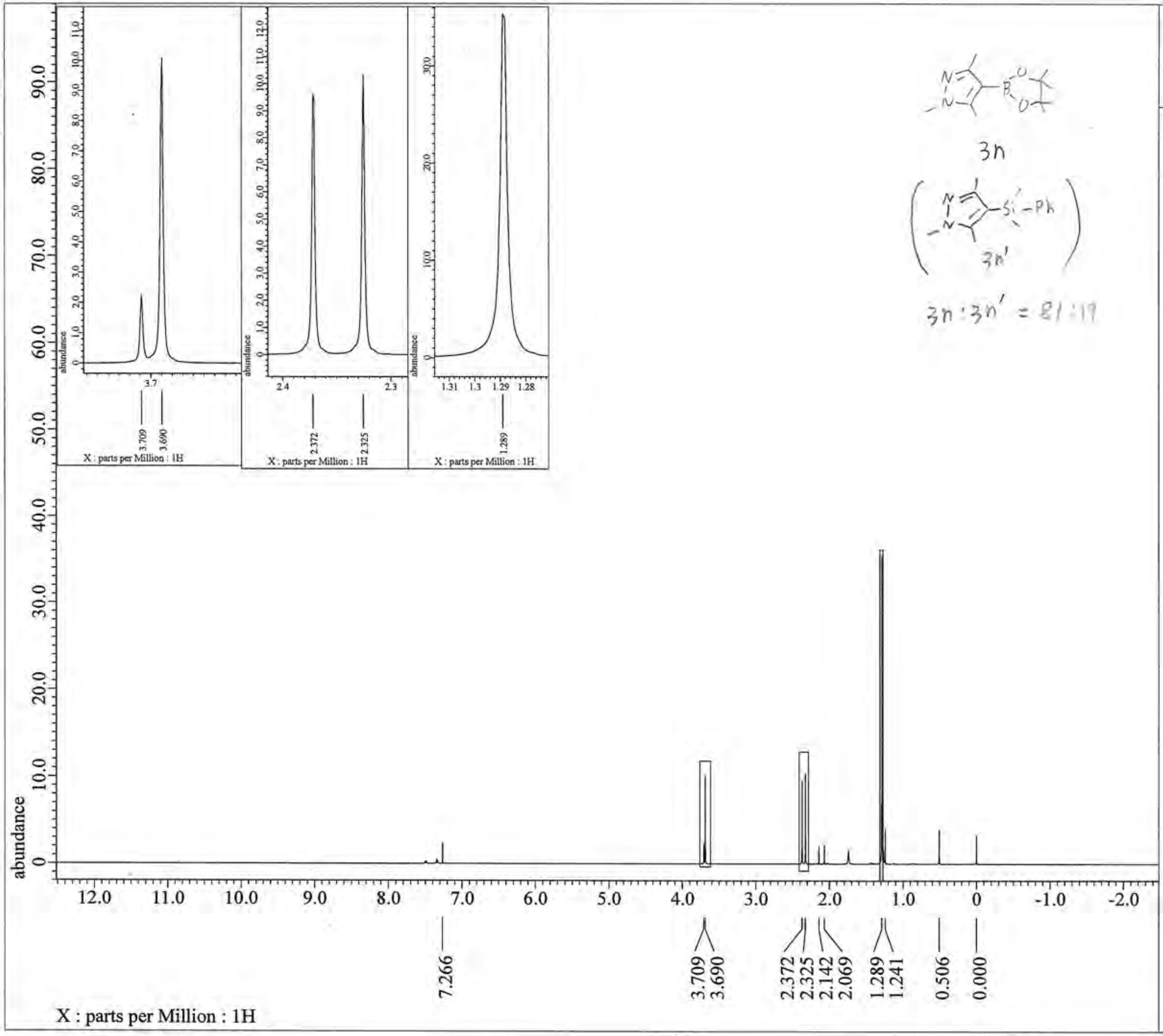

JEOL RESONANCE

```
Filename          = UKI-109-A proton-5.jdf
Author            = element
Experiment        = single_pulse.ex2
Sample_Id         = 109
Solvent           = CHLOROFORM-D
Creation_Time     =  5-AUG-2014 18:59:15
Revision_Time     =  5-AUG-2014 20:08:48
Current_Time      =  5-AUG-2014 20:10:02

Comment           = single_pulse
Data_Format       = 1D COMPLEX
Dim_Size          = 16384
Dim_Title         = 1H
Dim_Units         = [ppm]
Dimensions        = X
Site              = ECS 400
Spectrometer      = JNM-ECS400

Field_Strength    = 9.20197068[T] (390[MHz])
X_Acq_Duration    = 2.78790144[s]
X_Domain          = 1H
X_Freq            = 391.78655441[MHz]
X_Offset          = 5[ppm]
X_Points          = 16384
X_Prescans        = 1
X_Resolution      = 0.35869274[Hz]
X_Sweep           = 5.87682181[kHz]
Irr_Domain        = 1H
Irr_Freq          = 391.78655441[MHz]
Irr_Offset        = 5[ppm]
Tri_Domain        = 1H
Tri_Freq          = 391.78655441[MHz]
Tri_Offset        = 5[ppm]
Clipped           = FALSE
Scans             = 20
Total_Scans       = 20

Relaxation_Delay  = 4[s]
Recvr_Gain        = 44
Temp_Get          = 21.5[dC]
X_90_Width        = 10.7[us]
X_Acq_Time        = 2.78790144[s]
X_Angle           = 45[deg]
X_Atn             = 1.9[dB]
X_Pulse           = 5.35[us]
Irr_Mode          = Off
Tri_Mode          = Off
Dante_Presat      = FALSE
Initial_Wait      = 1[s]
Repetition_Time   = 6.78790144[s]
```

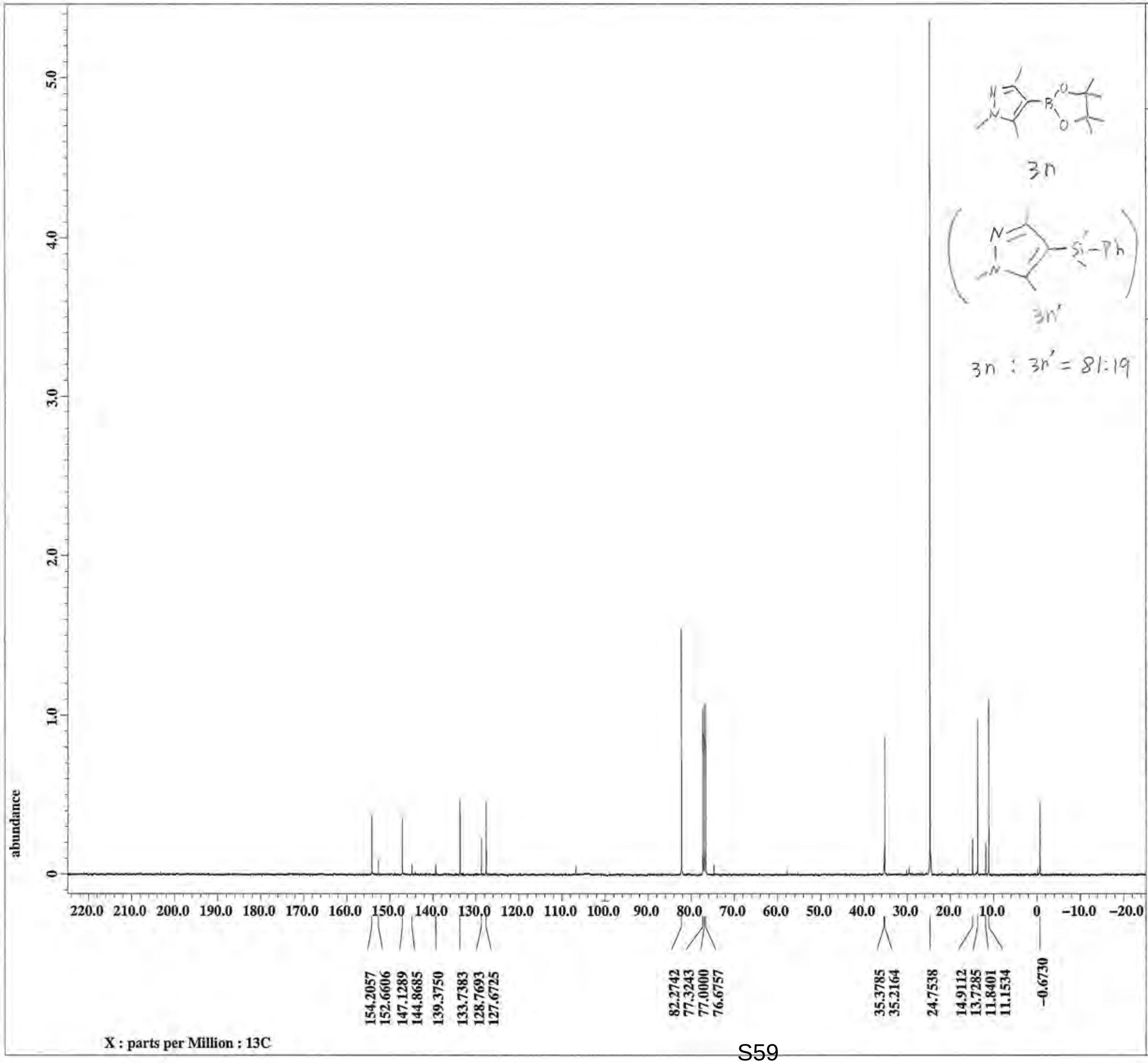

---- PROCESSING PARAMETERS ----
dc_balance : 0 : FALSE
sexp : 2.0[Hz] : 0.0[s]
trapezoid3 : 0[%] : 80[%] : 100[%]
zerofill : 1
fft : 1 : TRUE : TRUE
machinephase
ppm

Derived from: UKI-109-B-2 carbon-1.jdf

Filename	= UKI-109-B-2 carbon-3.
Author	= element
Experiment	= single_pulse_dec
Sample_id	= S#536422
Solvent	= CHLOROFORM-D
Creation_time	= 6-JAN-2015 14:42:59
Revision_time	= 6-JAN-2015 15:11:54
Current_time	= 6-JAN-2015 15:12:24
Comment	= single pulse decouple
Data_format	= 1D COMPLEX
Dim_size	= 26214
Dim_title	= 13C
Dim_units	= [ppm]
Dimensions	= X
Site	= ECS 400
Spectrometer	= JNM-ECS400
Field_strength	= 9.20197068[T] (390[MH
X_acq_duration	= 1.06430464[s]
X_domain	= 13C
X_freq	= 98.51479726[MHz]
X_offset	= 100[ppm]
X_points	= 32768
X_prescans	= 4
X_resolution	= 0.93958061[Hz]
X_sweep	= 30.78817734[kHz]
Irr_domain	= 1H
Irr_freq	= 391.78655441[MHz]
Irr_offset	= 5[ppm]
Clipped	= FALSE
Mod_return	= 1
Scans	= 300
Total_scans	= 300
X_90_width	= 8.8[us]
X_acq_time	= 1.06430464[s]
X_angle	= 30[deg]
X_atn	= 4.9[dB]
X_pulse	= 2.93333333[us]
Irr_atn_dec	= 22.52628[dB]
Irr_atn_noe	= 22.52628[dB]
Irr_noise	= WALTZ
Decoupling	= TRUE
Initial_wait	= 1[s]
Noe	= TRUE
Noe_time	= 2[s]
Recvr_gain	= 60
Relaxation_delay	= 2[s]
Repetition_time	= 3.06430464[s]
Temp_get	= 19.8[dC]

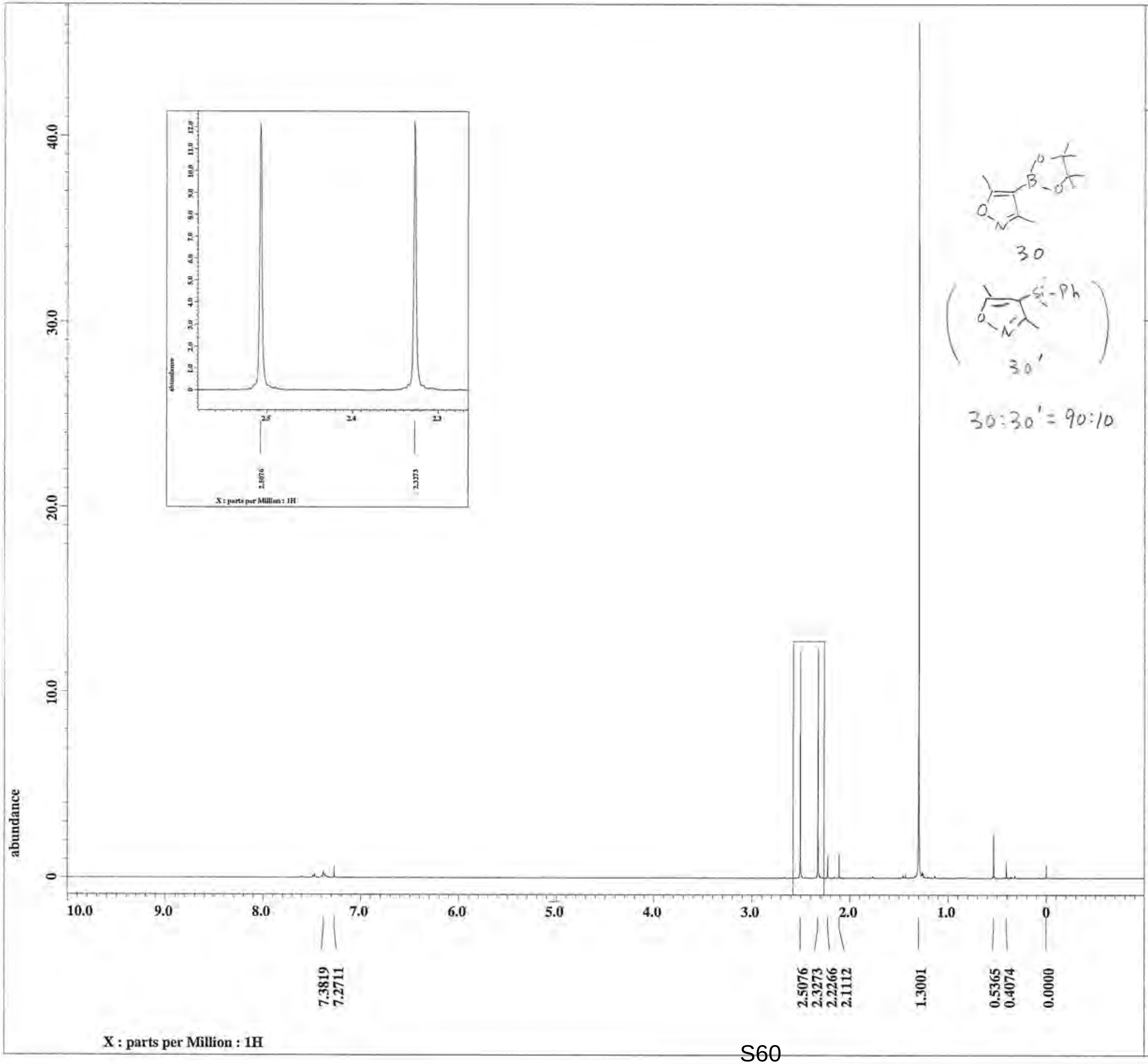


```
---- PROCESSING PARAMETERS ----
dc_balance : 0 : FALSE
sexp : 0.2[Hz] : 0.0[s]
trapezoid3 : 0[%] : 80[%] : 100[%]
zerofill : 1
fft : 1 : TRUE : TRUE
machinephase
ppm

Derived from: UKI-092-A proton-1.jdf
```

```
Filename           = UKI-092-A proton-4.jd
Author             = element
Experiment         = single_pulse.ex2
Sample_id          = 092
Solvent            = CHLOROFORM-D
Creation_time      =  2-NOV-2013 14:19:27
Revision_time      =  2-NOV-2013 15:35:43
Current_time       =  2-NOV-2013 15:35:48

Comment            = single_pulse
Data_format        = 1D COMPLEX
Dim_size           = 16384
Dim_title          = 1H
Dim_units          = [ppm]
Dimensions         = X
Site               = ECS 400
Spectrometer       = JNM-ECS400

Field_strength     = 9.20197068[T] (390[MH
X_acq_duration     = 2.78790144[s]
X_domain           = 1H
X_freq             = 391.78655441[MHz]
X_offset           = 5[ppm]
X_points           = 16384
X_prescans         = 1
X_resolution       = 0.35869274[Hz]
X_sweep            = 5.87682181[kHz]
Irr_domain         = 1H
Irr_freq           = 391.78655441[MHz]
Irr_offset         = 5[ppm]
Tri_domain         = 1H
Tri_freq           = 391.78655441[MHz]
Tri_offset         = 5[ppm]
Clipped            = FALSE
Mod_return         = 1
Scans              = 8
Total_scans        = 8

X_90_width         = 10.8[us]
X_acq_time         = 2.78790144[s]
X_angle            = 45[deg]
X_atn              = 1.9[dB]
X_pulse            = 5.4[us]
Irr_mode           = Off
Tri_mode           = Off
Dante_presat       = FALSE
Initial_wait       = 1[s]
Recvr_gain         = 32
Relaxation_delay   = 5[s]
Repetition_time    = 7.78790144[s]
Temp_get           = 20.1[dC]
```

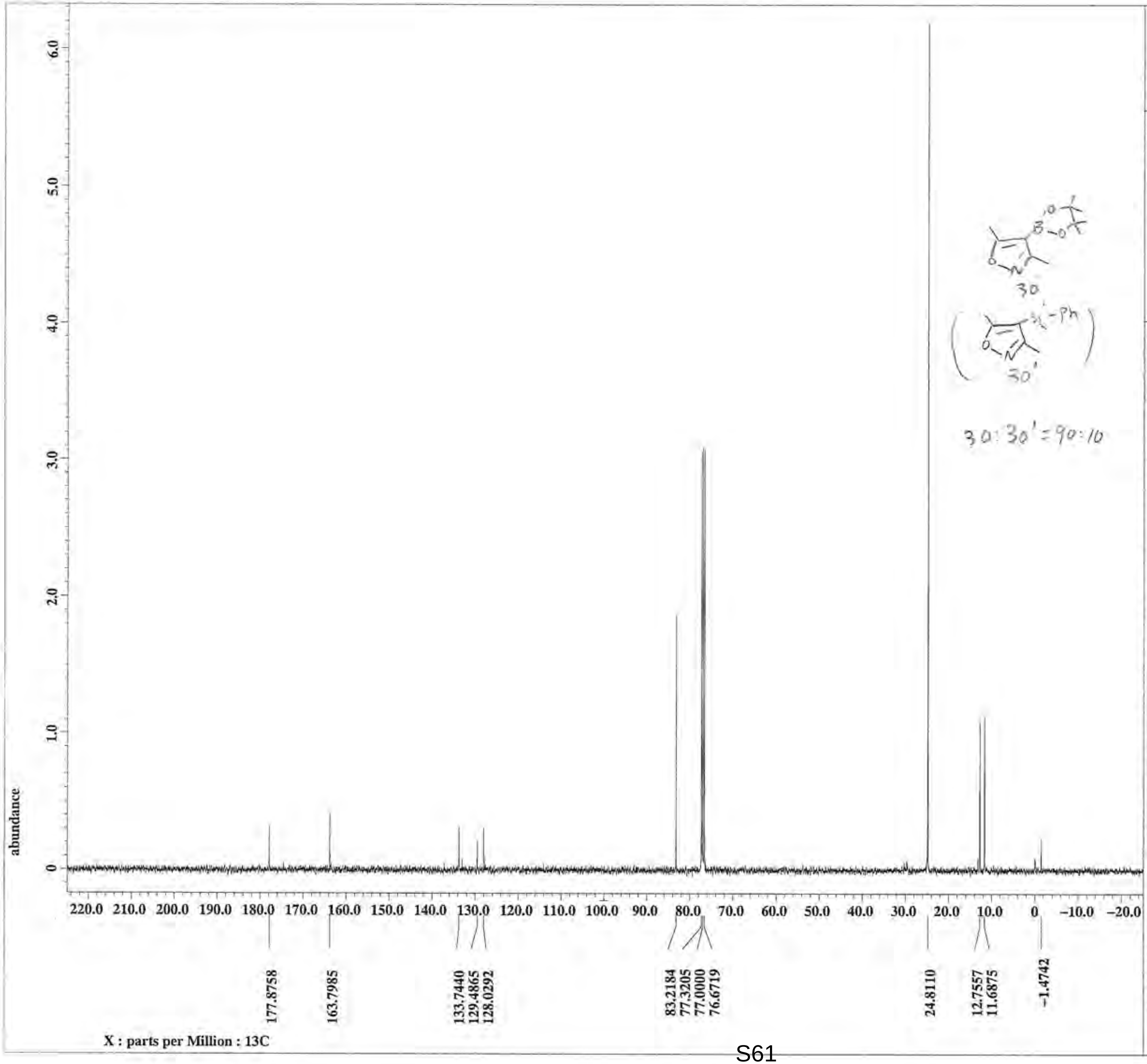

JEOL

---- PROCESSING PARAMETERS ----
dc_balance : 0 : FALSE
sexp : 2.0[Hz] : 0.0[s]
trapezoid3 : 0[%] : 80[%] : 100[%]
zerofill : 1
fft : 1 : TRUE : TRUE
machinephase
ppm

Derived from: UKI-092-B carbon-1.jdf

Filename	= UKI-092-B carbon-3.jd
Author	= element
Experiment	= single_pulse_dec
Sample_id	= 092
Solvent	= CHLOROFORM-D
Creation_time	= 2-NOV-2013 14:31:14
Revision_time	= 2-NOV-2013 15:39:50
Current_time	= 2-NOV-2013 15:41:11
Comment	= single pulse decouple
Data_format	= 1D COMPLEX
Dim_size	= 32768
Dim_title	= 13C
Dim_units	= [ppm]
Dimensions	= X
Site	= ECS 400
Spectrometer	= JNM-ECS400
Field_strength	= 9.20197068[T] (390[MH
X_acq_duration	= 1.3303808[s]
X_domain	= 13C
X_freq	= 98.51479726[MHz]
X_offset	= 100[ppm]
X_points	= 32768
X_prescans	= 4
X_resolution	= 0.75166449[Hz]
X_sweep	= 24.63054187[kHz]
Irr_domain	= 1H
Irr_freq	= 391.78655441[MHz]
Irr_offset	= 5[ppm]
Clipped	= FALSE
Mod_return	= 1
Scans	= 200
Total_scans	= 200
X_90_width	= 8.15[us]
X_acq_time	= 1.3303808[s]
X_angle	= 30[deg]
X_atn	= 4.9[dB]
X_pulse	= 2.71666667[us]
Irr_atn_dec	= 22.445[dB]
Irr_atn_noe	= 22.445[dB]
Irr_noise	= WALTZ
Decoupling	= TRUE
Initial_wait	= 1[s]
Noe	= TRUE
Noe_time	= 2[s]
Recvr_gain	= 70
Relaxation_delay	= 2[s]
Repetition_time	= 3.3303808[s]
Temp_get	= 20.5[dC]

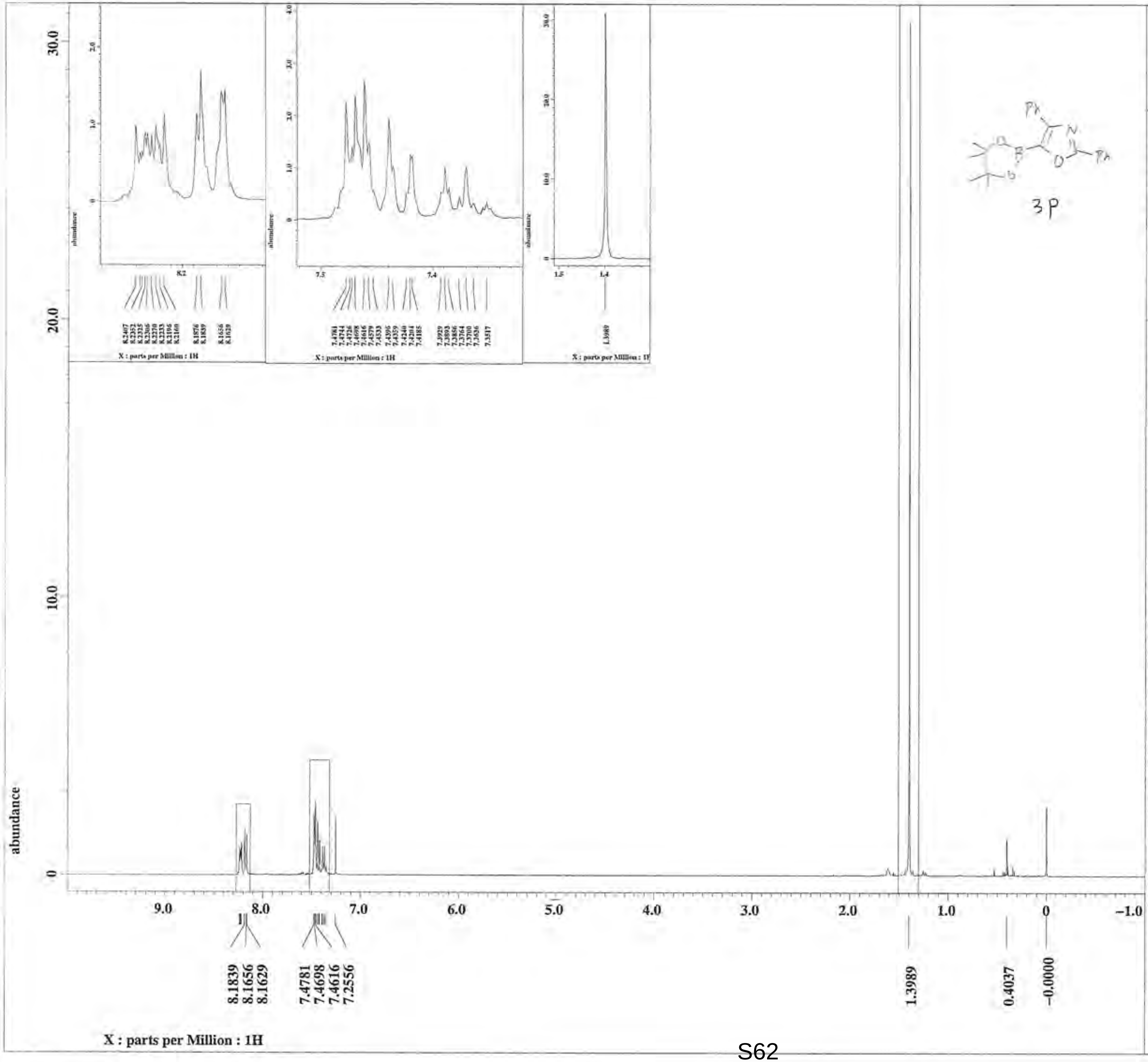

JEOL

```
---- PROCESSING PARAMETERS ----
dc_balance : 0 : FALSE
sexp : 0.2[Hz] : 0.0[s]
trapezoid3 : 0[%] : 80[%] : 100[%]
zerofill : 1
fft : 1 : TRUE : TRUE
machinephase
ppm

Derived from: UKI-161-A proton-1.jdf

Filename           = UKI-161-A proton-4.jd
Author             = element
Experiment         = single_pulse.ex2
Sample_id          = 161
Solvent            = CHLOROFORM-D
Creation_time      = 22-JUL-2014 19:54:30
Revision_time      = 22-JUL-2014 20:24:15
Current_time       = 22-JUL-2014 20:24:16

Comment            = single_pulse
Data_format        = 1D COMPLEX
Dim_size           = 16384
Dim_title          = 1H
Dim_units          = [ppm]
Dimensions         = X
Site               = ECS 400
Spectrometer       = JNM-ECS400

Field_strength     = 9.20197068[T] (390[MH
X_acq_duration     = 2.78790144[s]
X_domain           = 1H
X_freq             = 391.78655441[MHz]
X_offset           = 5[ppm]
X_points           = 16384
X_prescans         = 1
X_resolution       = 0.35869274[Hz]
X_sweep            = 5.87682181[kHz]
Irr_domain         = 1H
Irr_freq           = 391.78655441[MHz]
Irr_offset         = 5[ppm]
Tri_domain         = 1H
Tri_freq           = 391.78655441[MHz]
Tri_offset         = 5[ppm]
Clipped            = FALSE
Mod_return         = 1
Scans              = 15
Total_scans        = 15

X_90_width         = 10.7[us]
X_acq_time         = 2.78790144[s]
X_angle            = 45[deg]
X_atn              = 1.9[dB]
X_pulse            = 5.35[us]
Irr_mode           = Off
Tri_mode           = Off
Dante_presat       = FALSE
Initial_wait       = 1[s]
Recvr_gain         = 42
Relaxation_delay   = 4[s]
Repetition_time    = 6.78790144[s]
Temp_get           = 20.3[dC]
```

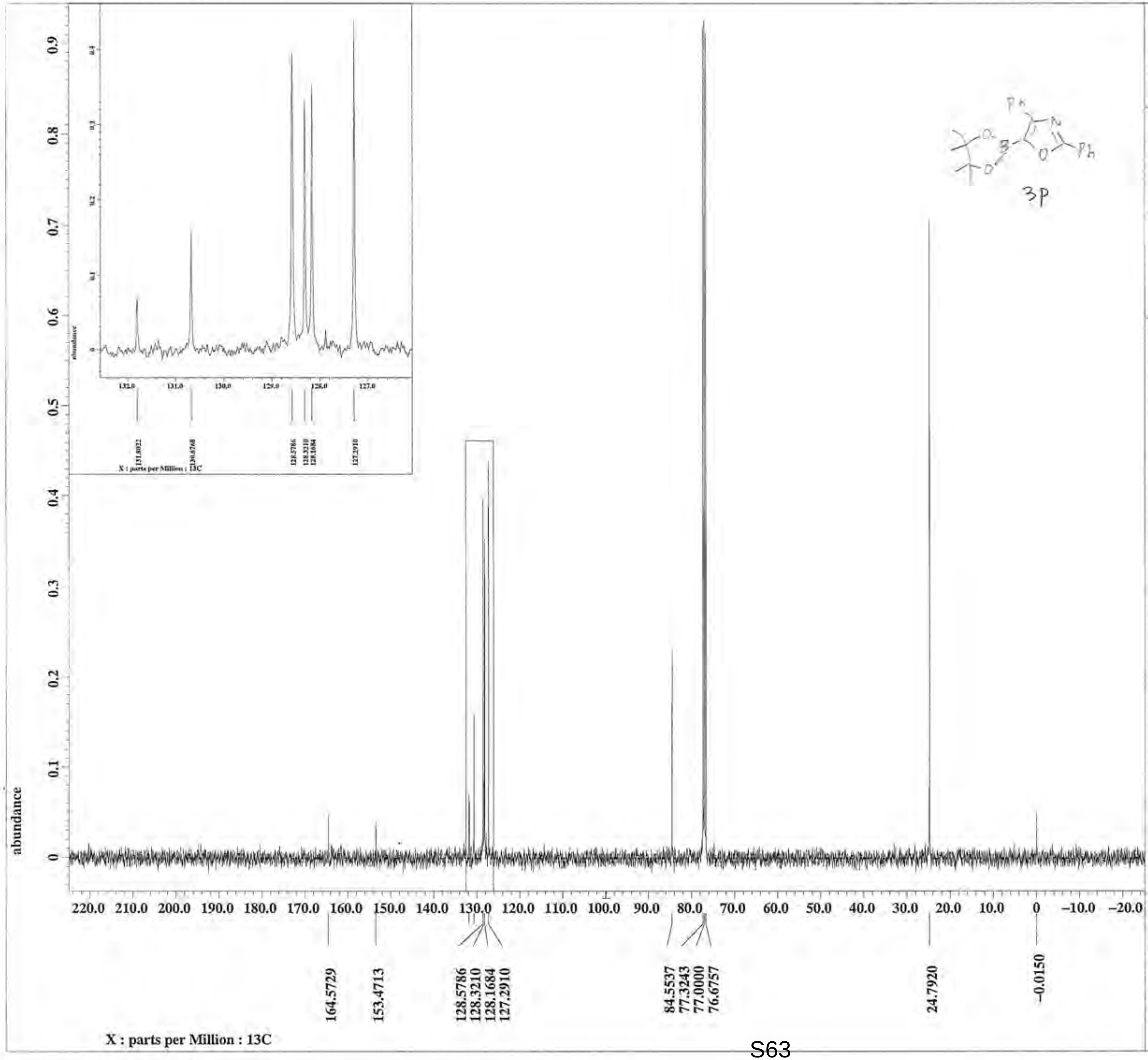

---- PROCESSING PARAMETERS ----
dc_balance : 0 : FALSE
sexp : 2.0[Hz] : 0.0[s]
trapezoid3 : 0[%] : 80[%] : 100[%]
zerofill : 1
fft : 1 : TRUE : TRUE
machinephase
ppm

Derived from: UKI-161-B carbon-1.jdf

Filename	= UKI-161-B carbon-5.jd
Author	= element
Experiment	= single_pulse_dec
Sample_id	= 161
Solvent	= CHLOROFORM-D
Creation_time	= 22-JUL-2014 20:05:55
Revision_time	= 22-JUL-2014 20:25:59
Current_time	= 22-JUL-2014 20:26:58
Comment	= single pulse decouple
Data_format	= 1D COMPLEX
Dim_size	= 26214
Dim_title	= 13C
Dim_units	= [ppm]
Dimensions	= X
Site	= ECS 400
Spectrometer	= JNM-ECS400
Field_strength	= 9.20197068[T] (390[MH
X_acq_duration	= 1.06430464[s]
X_domain	= 13C
X_freq	= 98.51479726[MHz]
X_offset	= 100[ppm]
X_points	= 32768
X_prescans	= 4
X_resolution	= 0.93958061[Hz]
X_sweep	= 30.78817734[kHz]
Irr_domain	= 1H
Irr_freq	= 391.78655441[MHz]
Irr_offset	= 5[ppm]
Clipped	= FALSE
Mod_return	= 1
Scans	= 200
Total_scans	= 200
X_90_width	= 8.8[us]
X_acq_time	= 1.06430464[s]
X_angle	= 30[deg]
X_atn	= 4.9[dB]
X_pulse	= 2.93333333[us]
Irr_atn_dec	= 22.52628[dB]
Irr_atn_noe	= 22.52628[dB]
Irr_noise	= WALTZ
Decoupling	= TRUE
Initial_wait	= 1[s]
Noe	= TRUE
Noe_time	= 2[s]
Recvr_gain	= 60
Relaxation_delay	= 2[s]
Repetition_time	= 3.06430464[s]
Temp_get	= 20.5[dC]

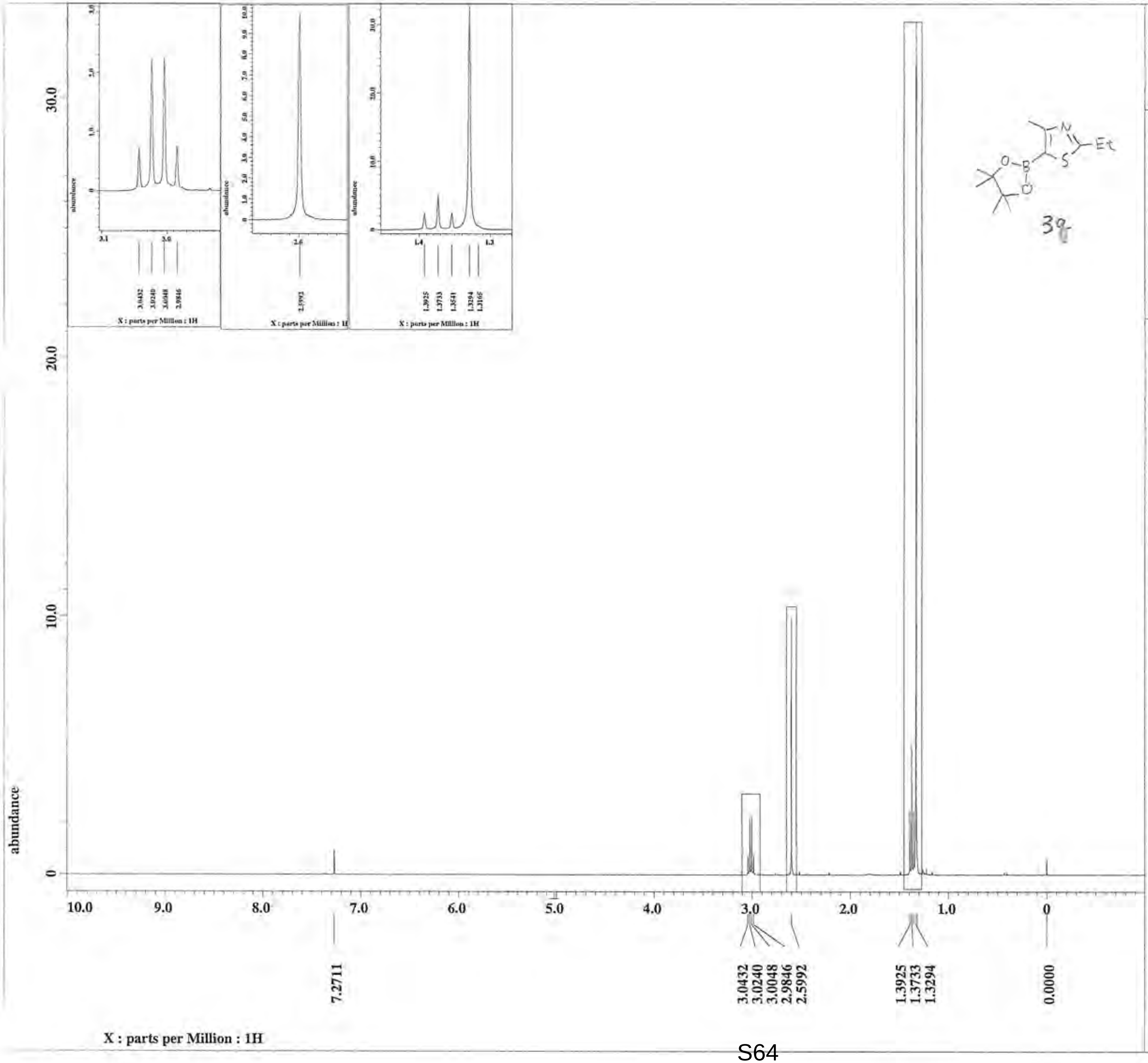

---- PROCESSING PARAMETERS ----
dc_balance : 0 : FALSE
sexp : 0.2[Hz] : 0.0[s]
trapezoid3 : 0[%] : 80[%] : 100[%]
zerofill : 1
fft : 1 : TRUE : TRUE
machinephase
ppm

Derived from: UKI-176-A H-1.jdf

Filename	= UKI-176-A H-7.jdf
Author	= element
Experiment	= single_pulse.ex2
Sample_id	= UKI176
Solvent	= CHLOROFORM-D
Creation_time	= 9-JUN-2014 14:13:56
Revision_time	= 9-JUN-2014 14:19:31
Current_time	= 9-JUN-2014 14:19:34
Comment	= single_pulse
Data_format	= 1D COMPLEX
Dim_size	= 16384
Dim_title	= 1H
Dim_units	= [ppm]
Dimensions	= X
Site	= ECS 400
Spectrometer	= JNM-ECS400
Field_strength	= 9.20197068[T] (390[MH
X_acq_duration	= 2.78790144[s]
X_domain	= 1H
X_freq	= 391.78655441[MHz]
X_offset	= 5[ppm]
X_points	= 16384
X_prescans	= 1
X_resolution	= 0.35869274[Hz]
X_sweep	= 5.87682181[kHz]
Irr_domain	= 1H
Irr_freq	= 391.78655441[MHz]
Irr_offset	= 5[ppm]
Tri_domain	= 1H
Tri_freq	= 391.78655441[MHz]
Tri_offset	= 5[ppm]
Clipped	= FALSE
Mod_return	= 1
Scans	= 8
Total_scans	= 8
X_90_width	= 10.7[us]
X_acq_time	= 2.78790144[s]
X_angle	= 45[deg]
X_atn	= 1.9[dB]
X_pulse	= 5.35[us]
Irr_mode	= Off
Tri_mode	= Off
Dante_presat	= FALSE
Initial_wait	= 1[s]
Recvr_gain	= 36
Relaxation_delay	= 4[s]
Repetition_time	= 6.78790144[s]
Temp_get	= 21.8[dC]

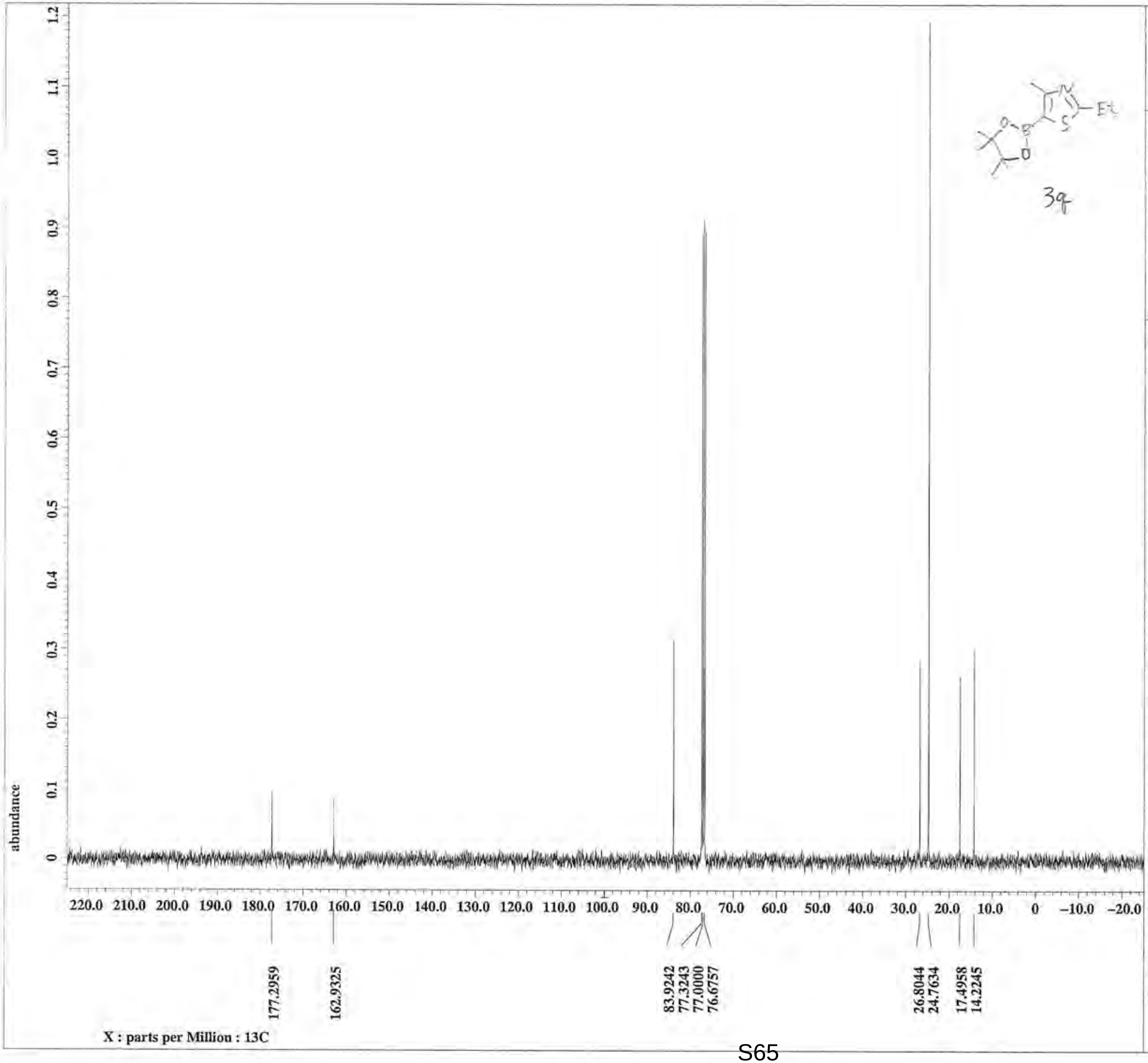

---- PROCESSING PARAMETERS ----
dc_balance : 0 : FALSE
sexp : 2.0[Hz] : 0.0[s]
trapezoid3 : 0[%] : 80[%] : 100[%]
zerofill : 1
fft : 1 : TRUE : TRUE
machinephase
ppm

Derived from: UKI-1176-B-1.jdf

Filename	= UKI-1176-B-3.jdf
Author	= element
Experiment	= single_pulse_dec
Sample_id	= UKI176
Solvent	= CHLOROFORM-D
Creation_time	= 9-JUN-2014 14:22:25
Revision_time	= 9-JUN-2014 14:23:22
Current_time	= 9-JUN-2014 14:23:31
Comment	= single pulse decouple
Data_format	= 1D COMPLEX
Dim_size	= 26214
Dim_title	= 13C
Dim_units	= [ppm]
Dimensions	= X
Site	= ECS 400
Spectrometer	= JNM-ECS400
Field_strength	= 9.20197068[T] (390[MH
X_acq_duration	= 1.06430464[s]
X_domain	= 13C
X_freq	= 98.51479726[MHz]
X_offset	= 100[ppm]
X_points	= 32768
X_prescans	= 4
X_resolution	= 0.93958061[Hz]
X_sweep	= 30.78817734[kHz]
Irr_domain	= 1H
Irr_freq	= 391.78655441[MHz]
Irr_offset	= 5[ppm]
Clipped	= FALSE
Mod_return	= 1
Scans	= 150
Total_scans	= 150
X_90_width	= 8.8[us]
X_acq_time	= 1.06430464[s]
X_angle	= 30[deg]
X_atn	= 4.9[dB]
X_pulse	= 2.93333333[us]
Irr_atn_dec	= 22.52628[dB]
Irr_atn_noe	= 22.52628[dB]
Irr_noise	= WALTZ
Decoupling	= TRUE
Initial_wait	= 1[s]
Noe	= TRUE
Noe_time	= 2[s]
Recvr_gain	= 60
Relaxation_delay	= 2[s]
Repetition_time	= 3.06430464[s]
Temp_get	= 22[dC]

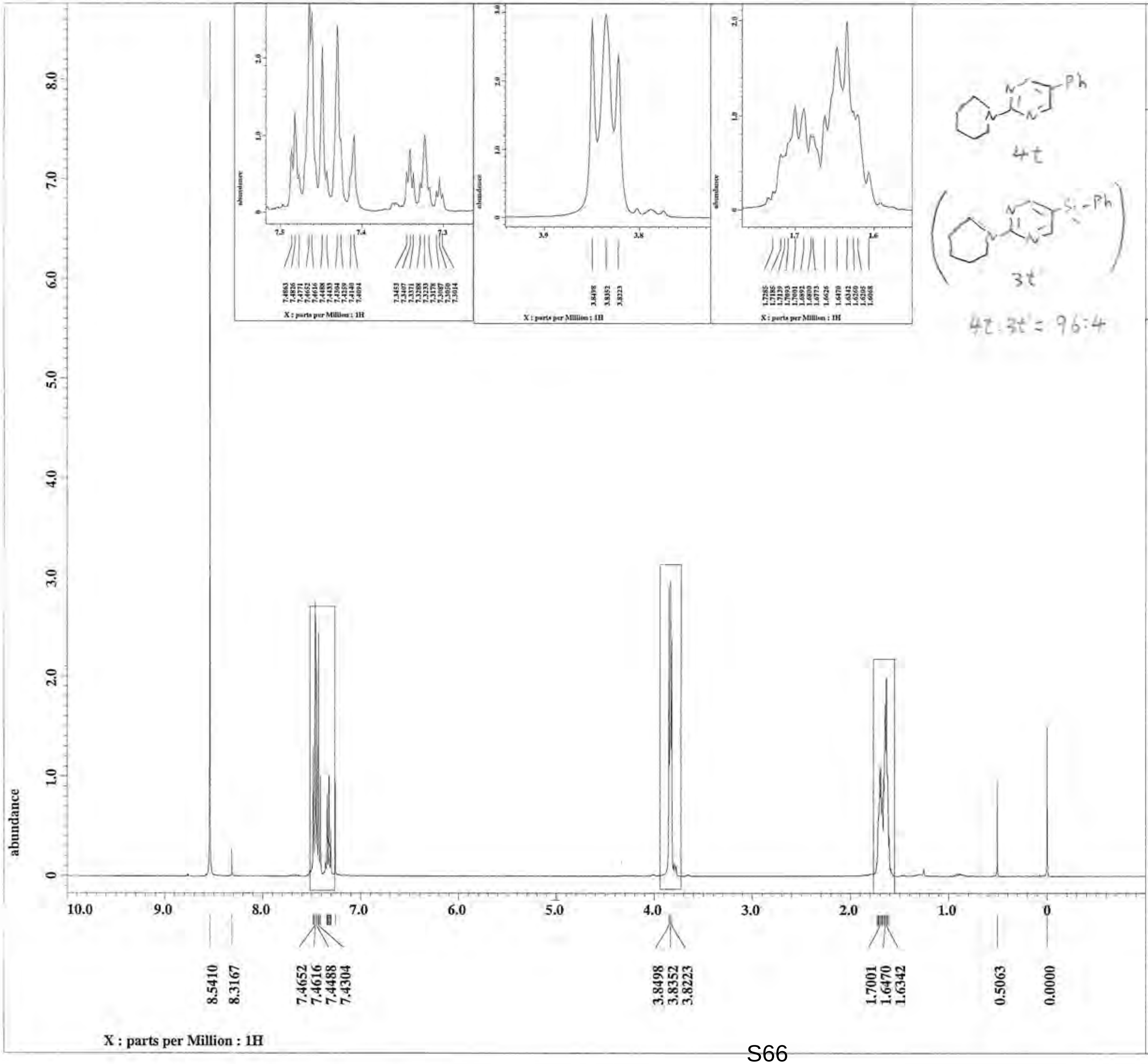

---- PROCESSING PARAMETERS ----
dc_balance : 0 : FALSE
sexp : 0.2[Hz] : 0.0[s]
trapezoid3 : 0[%] : 80[%] : 100[%]
zerofill : 1
fft : 1 : TRUE : TRUE
machinephase
ppm

Derived from: UKI-170-A-2 proton-1.jdf

Filename	= UKI-170-A-2 proton-4.
Author	= element
Experiment	= single_pulse.ex2
Sample_id	= 170
Solvent	= CHLOROFORM-D
Creation_time	= 11-SEP-2014 20:10:52
Revision_time	= 11-SEP-2014 20:29:33
Current_time	= 11-SEP-2014 20:29:56
Comment	= single_pulse
Data_format	= 1D COMPLEX
Dim_size	= 16384
Dim_title	= 1H
Dim_units	= [ppm]
Dimensions	= X
Site	= ECS 400
Spectrometer	= JNM-ECS400
Field_strength	= 9.20197068[T] (390[MH
X_acq_duration	= 2.78790144[s]
X_domain	= 1H
X_freq	= 391.78655441[MHz]
X_offset	= 5[ppm]
X_points	= 16384
X_prescans	= 1
X_resolution	= 0.35869274[Hz]
X_sweep	= 5.87682181[kHz]
Irr_domain	= 1H
Irr_freq	= 391.78655441[MHz]
Irr_offset	= 5[ppm]
Tri_domain	= 1H
Tri_freq	= 391.78655441[MHz]
Tri_offset	= 5[ppm]
Clipped	= FALSE
Mod_return	= 1
Scans	= 8
Total_scans	= 8
X_90_width	= 10.7[us]
X_acq_time	= 2.78790144[s]
X_angle	= 45[deg]
X_atn	= 1.9[dB]
X_pulse	= 5.35[us]
Irr_mode	= Off
Tri_mode	= Off
Dante_presat	= FALSE
Initial_wait	= 1[s]
Recvr_gain	= 38
Relaxation_delay	= 4[s]
Repetition_time	= 6.78790144[s]
Temp_get	= 20.7[dC]

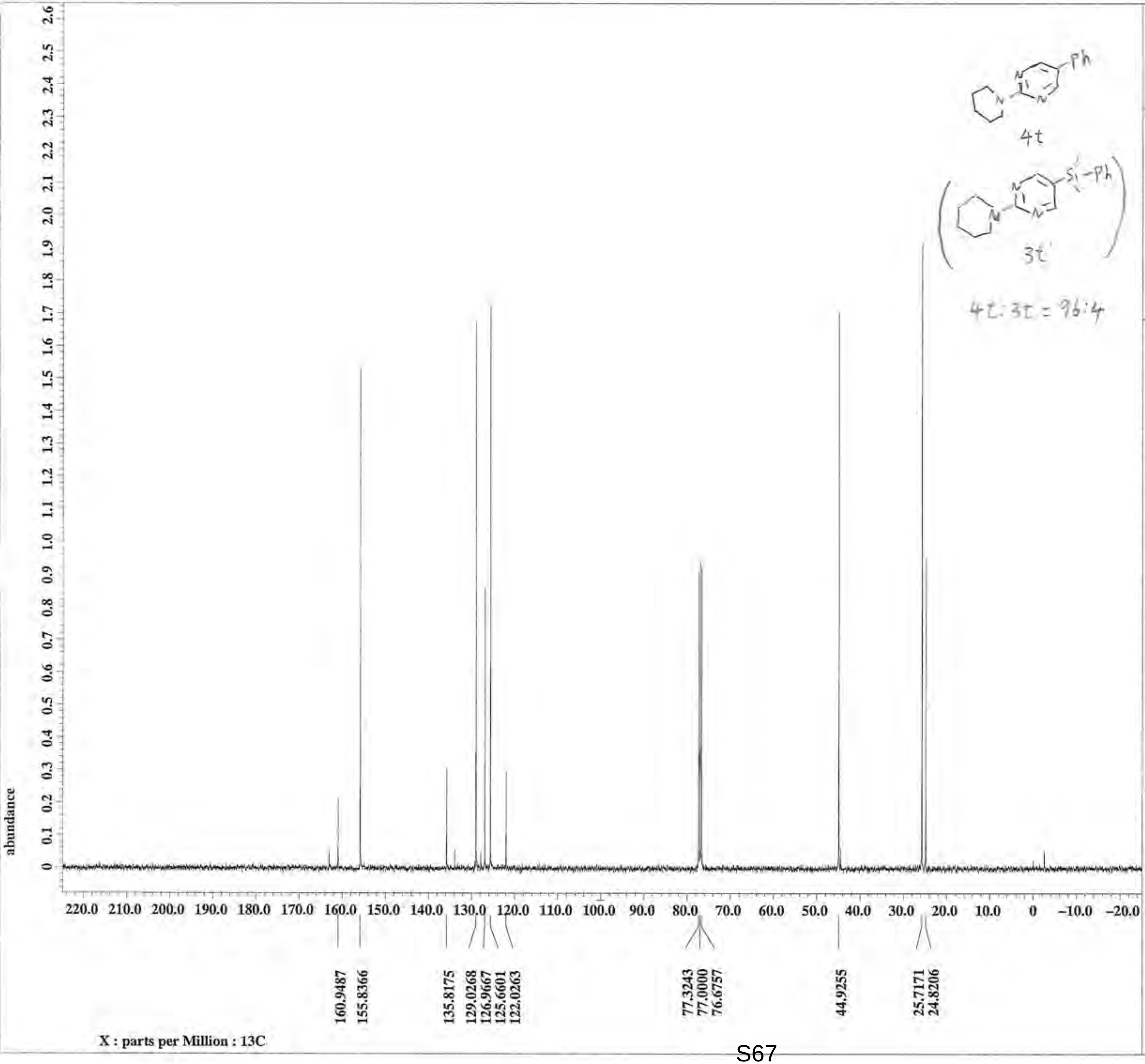

---- PROCESSING PARAMETERS ----
dc_balance : 0 : FALSE
sexp : 2.0[Hz] : 0.0[s]
trapezoid3 : 0[%] : 80[%] : 100[%]
zerofill : 1
fft : 1 : TRUE : TRUE
machinephase
ppm

Derived from: UKI-170-B carbon-1.jdf

Filename	= UKI-170-B carbon-3.jd
Author	= element
Experiment	= single_pulse_dec
Sample_id	= 170
Solvent	= CHLOROFORM-D
Creation_time	= 11-SEP-2014 15:46:11
Revision_time	= 11-SEP-2014 16:13:51
Current_time	= 11-SEP-2014 16:14:08
Comment	= single pulse decouple
Data_format	= 1D COMPLEX
Dim_size	= 26214
Dim_title	= 13C
Dim_units	= [ppm]
Dimensions	= X
Site	= ECS 400
Spectrometer	= JNM-ECS400
Field_strength	= 9.20197068[T] (390[MH
X_acq_duration	= 1.06430464[s]
X_domain	= 13C
X_freq	= 98.51479726[MHz]
X_offset	= 100[ppm]
X_points	= 32768
X_prescans	= 4
X_resolution	= 0.93958061[Hz]
X_sweep	= 30.78817734[kHz]
Irr_domain	= 1H
Irr_freq	= 391.78655441[MHz]
Irr_offset	= 5[ppm]
Clipped	= FALSE
Mod_return	= 1
Scans	= 200
Total_scans	= 200
X_90_width	= 8.8[us]
X_acq_time	= 1.06430464[s]
X_angle	= 30[deg]
X_atn	= 4.9[dB]
X_pulse	= 2.93333333[us]
Irr_atn_dec	= 22.52628[dB]
Irr_atn_noe	= 22.52628[dB]
Irr_noise	= WALTZ
Decoupling	= TRUE
Initial_wait	= 1[s]
Noe	= TRUE
Noe_time	= 2[s]
Recvr_gain	= 60
Relaxation_delay	= 2[s]
Repetition_time	= 3.06430464[s]
Temp_get	= 21.2[dC]

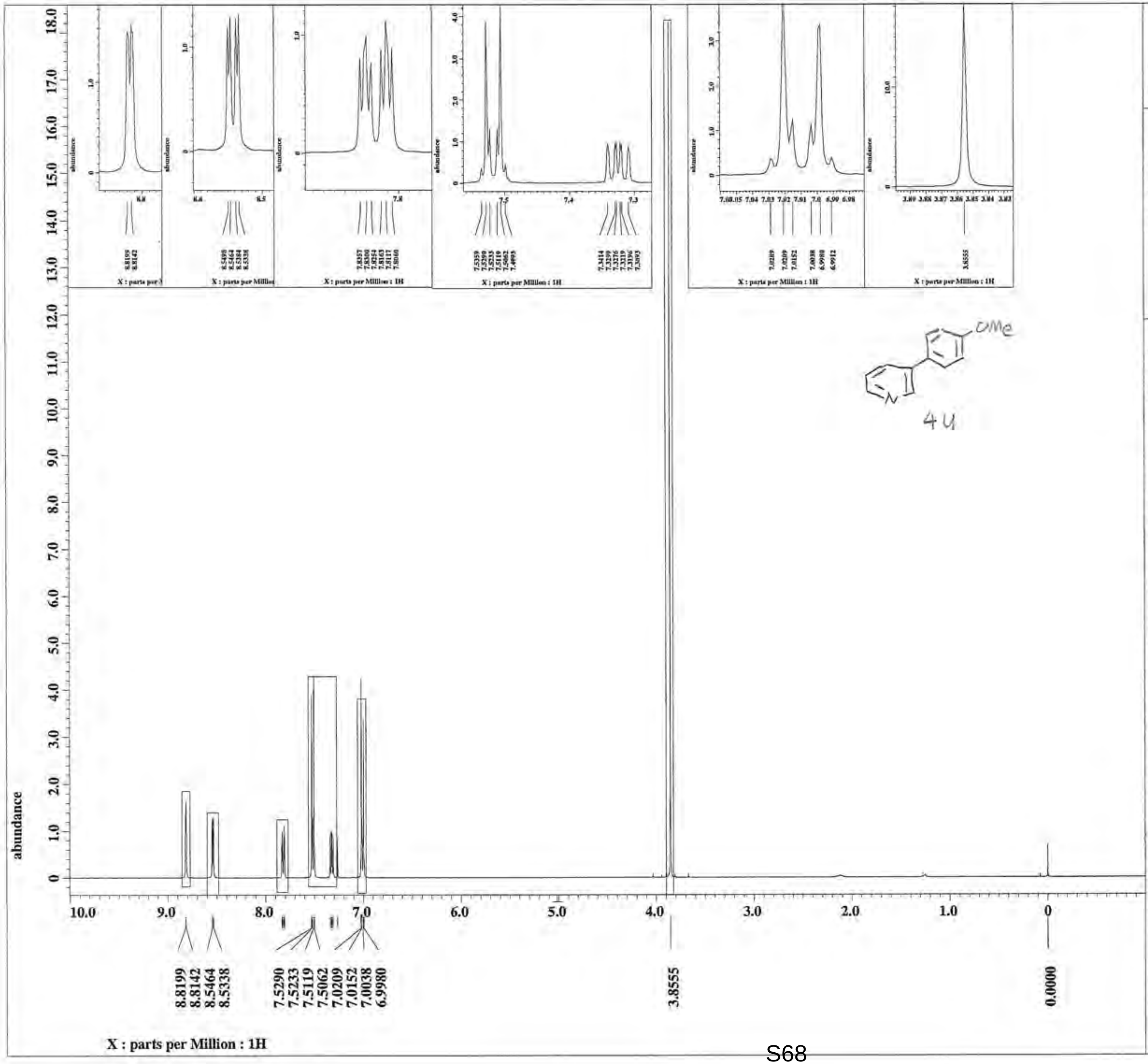


```
---- PROCESSING PARAMETERS ----
dc_balance : 0 : FALSE
sexp : 0.2[Hz] : 0.0[s]
trapezoid3 : 0[%] : 80[%] : 100[%]
zerofill : 1
fft : 1 : TRUE : TRUE
machinephase
ppm

Derived from: UKI-250-A proton-1.jdf
```

```
Filename          = UKI-250-A proton-4.jd
Author            = element
Experiment        = single_pulse.ex2
Sample_id         = 250
Solvent           = CHLOROFORM-D
Creation_time     = 22-OCT-2014 20:37:06
Revision_time     = 22-OCT-2014 20:48:22
Current_time      = 22-OCT-2014 20:48:25

Comment           = single_pulse
Data_format       = 1D COMPLEX
Dim_size          = 13107
Dim_title         = 1H
Dim_units         = [ppm]
Dimensions        = X
Site              = ECX 400P
Spectrometer      = DELTA2_NMR

Field_strength    = 9.2982153[T] (400[MHz
X_acq_duration    = 2.20725248[s]
X_domain          = 1H
X_freq            = 395.88430144[MHz]
X_offset          = 5[ppm]
X_points          = 16384
X_prescans        = 1
X_resolution      = 0.45305193[Hz]
X_sweep           = 7.42280285[kHz]
Irr_domain        = 1H
Irr_freq          = 395.88430144[MHz]
Irr_offset        = 5[ppm]
Tri_domain        = 1H
Tri_freq          = 395.88430144[MHz]
Tri_offset        = 5[ppm]
Clipped           = FALSE
Mod_return        = 1
Scans             = 8
Total_scans       = 8

X_90_width        = 12.6[us]
X_acq_time        = 2.20725248[s]
X_angle           = 45[deg]
X_atn             = 1[dB]
X_pulse           = 6.3[us]
Irr_mode          = Off
Tri_mode          = Off
Dante_presat      = FALSE
Initial_wait      = 1[s]
Recvr_gain        = 34
Relaxation_delay  = 5[s]
Repetition_time   = 7.20725248[s]
Temp_get          = 405.2[dC]
```

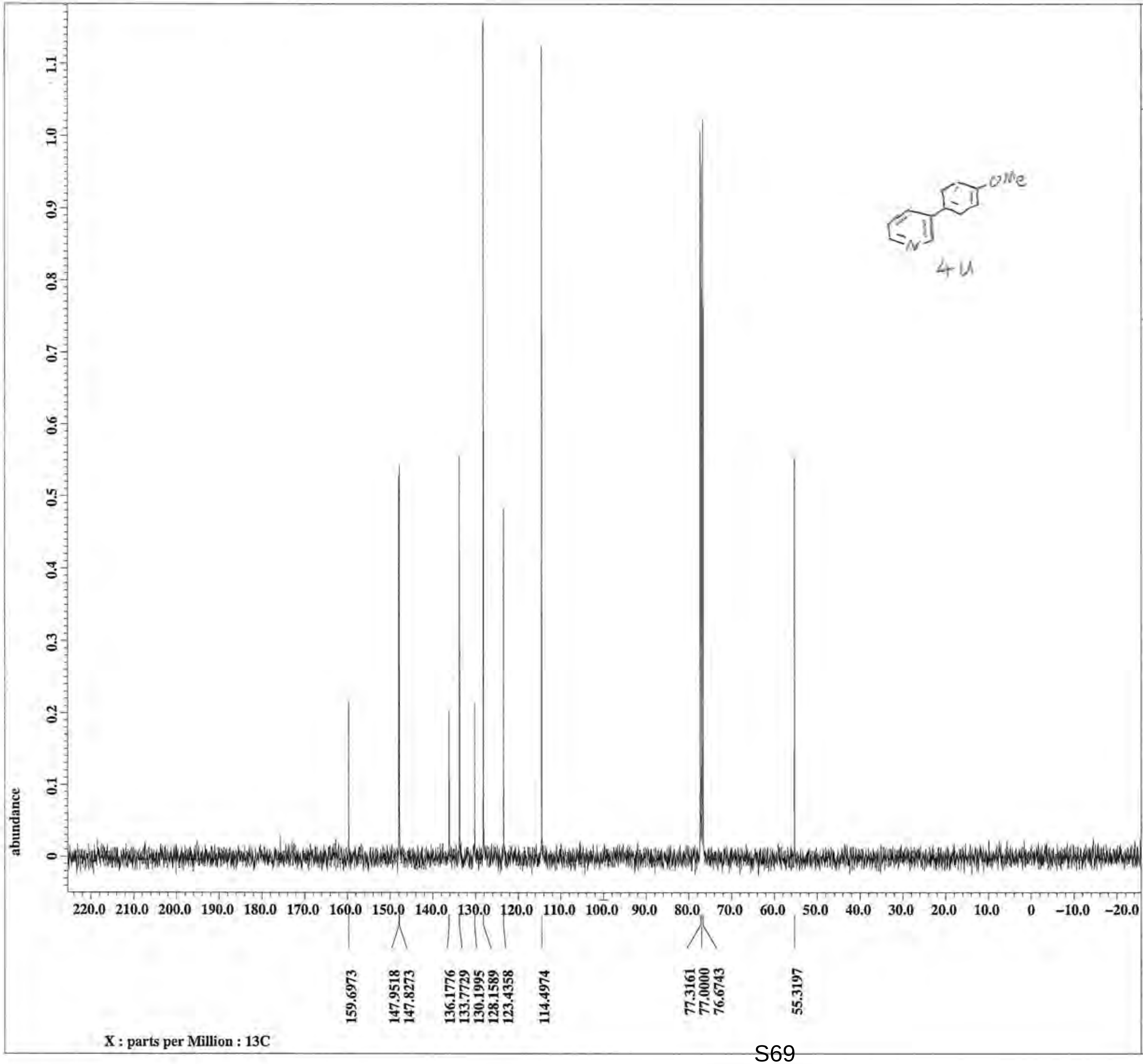


```
---- PROCESSING PARAMETERS ----
dc_balance : 0 : FALSE
sexp : 2.0[Hz] : 0.0[s]
trapezoid3 : 0[%] : 80[%] : 100[%]
zerofill : 1
fft : 1 : TRUE : TRUE
machinephase
ppm

Derived from: UKI-250-B carbon-1.jdf

Filename          = UKI-250-B carbon-3.jd
Author            = element
Experiment        = single_pulse_dec
Sample_id         = 250
Solvent           = CHLOROFORM-D
Creation_time     = 22-OCT-2014 20:43:28
Revision_time     = 22-OCT-2014 20:47:27
Current_time      = 22-OCT-2014 20:47:34

Comment           = single pulse decouple
Data_format       = 1D COMPLEX
Dim_size          = 26214
Dim_title         = 13C
Dim_units         = [ppm]
Dimensions        = X
Site              = ECX 400P
Spectrometer      = DELTA2_NMR

Field_strength    = 9.2982153[T] (400[MHz
X_acq_duration    = 1.048576[s]
X_domain          = 13C
X_freq            = 99.54517646[MHz]
X_offset          = 100[ppm]
X_points          = 32768
X_prescans        = 4
X_resolution      = 0.95367432[Hz]
X_sweep           = 31.25[kHz]
Irr_domain        = 1H
Irr_freq          = 395.88430144[MHz]
Irr_offset        = 5[ppm]
Clipped           = TRUE
Mod_return        = 1
Scans             = 100
Total_scans       = 100

X_90_width        = 9.3[us]
X_acq_time        = 1.048576[s]
X_angle           = 30[deg]
X_atn             = 3.4[dB]
X_pulse           = 3.1[us]
Irr_atn_dec       = 20.20655[dB]
Irr_atn_noe       = 20.20655[dB]
Irr_noise         = WALTZ
Decoupling        = TRUE
Initial_wait      = 1[s]
Noe               = TRUE
Noe_time          = 2[s]
Recvr_gain        = 58
Relaxation_delay  = 2[s]
Repetition_time   = 3.048576[s]
Temp_get          = 405.3[dC]
```

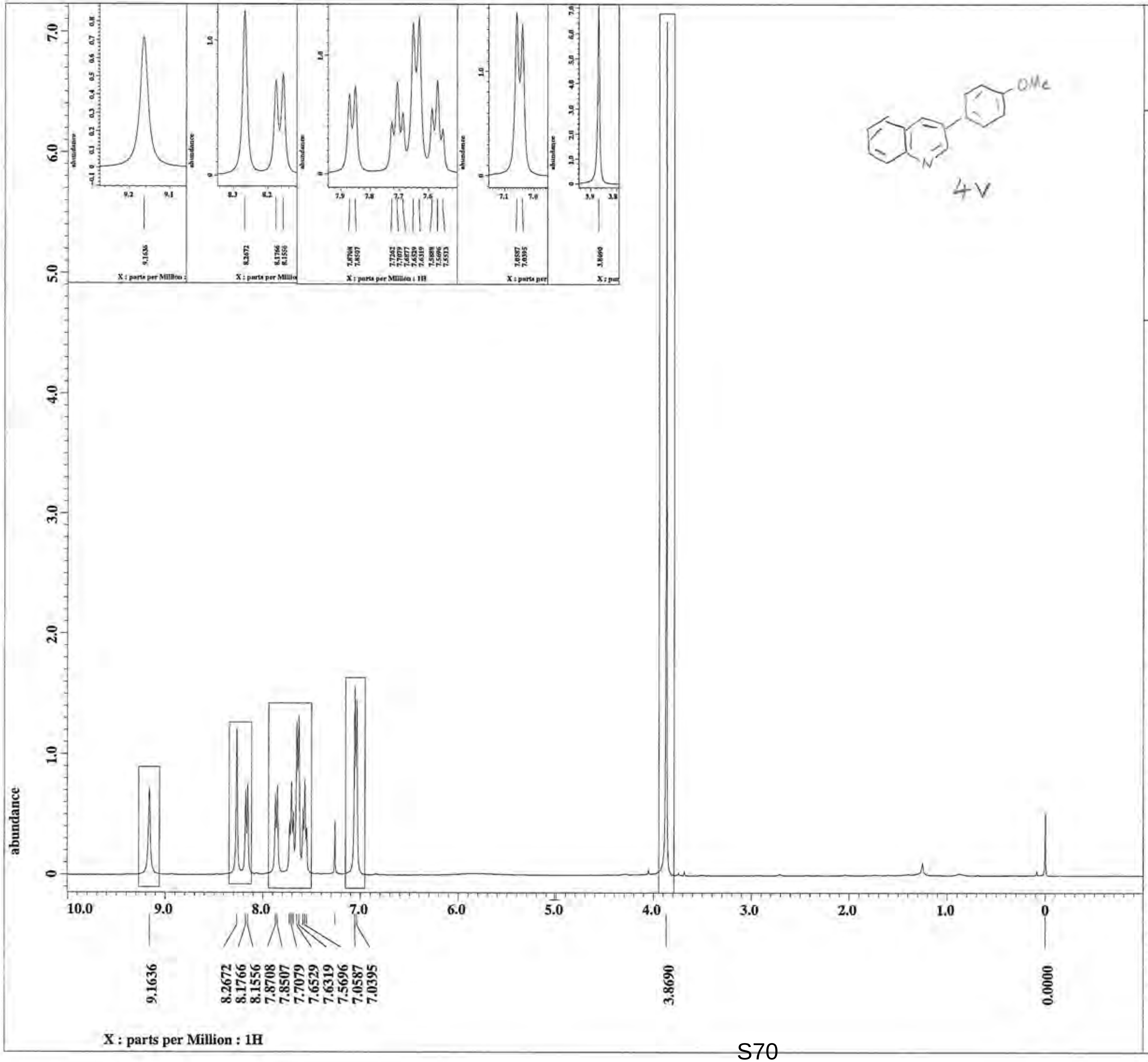

4V

---- PROCESSING PARAMETERS ----
dc_balance : 0 : FALSE
sexp : 0.2[Hz] : 0.0[s]
trapezoid3 : 0[%] : 80[%] : 100[%]
zerofill : 1
fft : 1 : TRUE : TRUE
machinephase
ppm

Derived from: UKI-262-A-2 proton-1.jdf

Filename	= UKI-262-A-2 proton-4.
Author	= element
Experiment	= single_pulse.ex2
Sample_id	= 262
Solvent	= CHLOROFORM-D
Creation_time	= 12-NOV-2014 22:16:15
Revision_time	= 12-NOV-2014 22:43:45
Current_time	= 12-NOV-2014 22:45:08
Comment	= single_pulse
Data_format	= 1D COMPLEX
Dim_size	= 16384
Dim_title	= 1H
Dim_units	= [ppm]
Dimensions	= X
Site	= ECS 400
Spectrometer	= JNM-ECS400
Field_strength	= 9.20197068[T] (390[MH
X_acq_duration	= 2.78790144[s]
X_domain	= 1H
X_freq	= 391.78655441[MHz]
X_offset	= 5[ppm]
X_points	= 16384
X_prescans	= 1
X_resolution	= 0.35869274[Hz]
X_sweep	= 5.87682181[kHz]
Irr_domain	= 1H
Irr_freq	= 391.78655441[MHz]
Irr_offset	= 5[ppm]
Tri_domain	= 1H
Tri_freq	= 391.78655441[MHz]
Tri_offset	= 5[ppm]
Clipped	= FALSE
Mod_return	= 1
Scans	= 10
Total_scans	= 10
X_90_width	= 10.7[us]
X_acq_time	= 2.78790144[s]
X_angle	= 45[deg]
X_atn	= 1.9[dB]
X_pulse	= 5.35[us]
Irr_mode	= Off
Tri_mode	= Off
Dante_presat	= FALSE
Initial_wait	= 1[s]
Recvr_gain	= 36
Relaxation_delay	= 4[s]
Repetition_time	= 6.78790144[s]
Temp_get	= 22.4[dC]

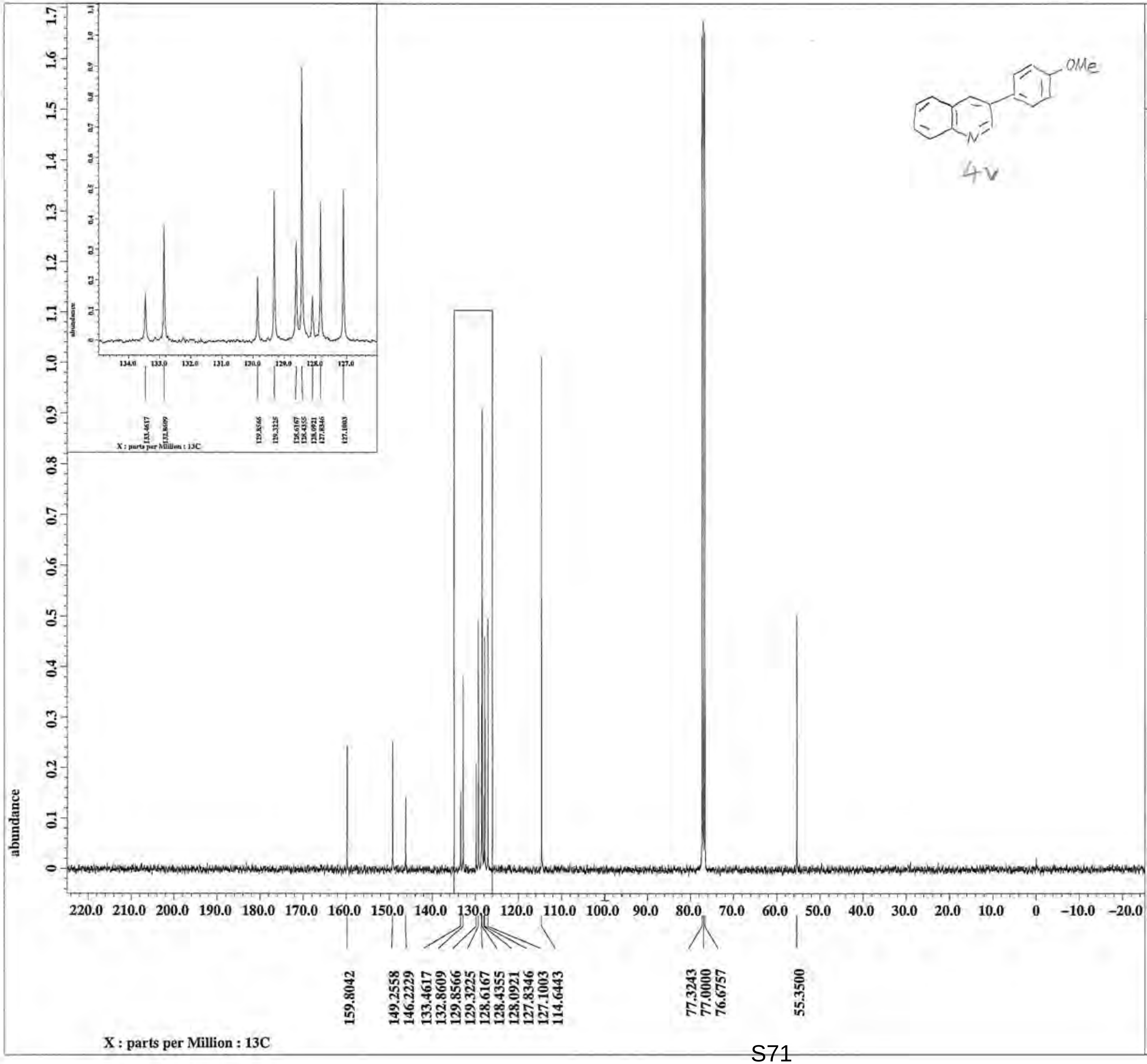

---- PROCESSING PARAMETERS ----
dc_balance : 0 : FALSE
sexp : 2.0[Hz] : 0.0[s]
trapezoid3 : 0[%] : 80[%] : 100[%]
zerofill : 1
fft : 1 : TRUE : TRUE
machinephase
ppm

Derived from: UKI-262-B-2 carbon-1.jdf

Filename = UKI-262-B-2 carbon-3.
Author = element
Experiment = single_pulse_dec
Sample_id = 262
Solvent = CHLOROFORM-D
Creation_time = 12-NOV-2014 22:33:06
Revision_time = 12-NOV-2014 22:54:23
Current_time = 12-NOV-2014 22:56:34

Comment = single pulse decouple
Data_format = 1D COMPLEX
Dim_size = 26214
Dim_title = 13C
Dim_units = [ppm]
Dimensions = X
Site = ECS 400
Spectrometer = JNM-ECS400

Field_strength = 9.20197068[T] (390[MH
X_acq_duration = 1.06430464[s]
X_domain = 13C
X_freq = 98.51479726[MHz]
X_offset = 100[ppm]
X_points = 32768
X_prescans = 4
X_resolution = 0.93958061[Hz]
X_sweep = 30.78817734[kHz]
Irr_domain = 1H
Irr_freq = 391.78655441[MHz]
Irr_offset = 5[ppm]
Clipped = FALSE
Mod_return = 1
Scans = 300
Total_scans = 300

X_90_width = 8.8[us]
X_acq_time = 1.06430464[s]
X_angle = 30[deg]
X_atn = 4.9[dB]
X_pulse = 2.93333333[us]
Irr_atn_dec = 22.52628[dB]
Irr_atn_noe = 22.52628[dB]
Irr_noise = WALTZ
Decoupling = TRUE
Initial_wait = 1[s]
Noe = TRUE
Noe_time = 2[s]
Recvr_gain = 60
Relaxation_delay = 2[s]
Repetition_time = 3.06430464[s]
Temp_get = 22.8[dC]

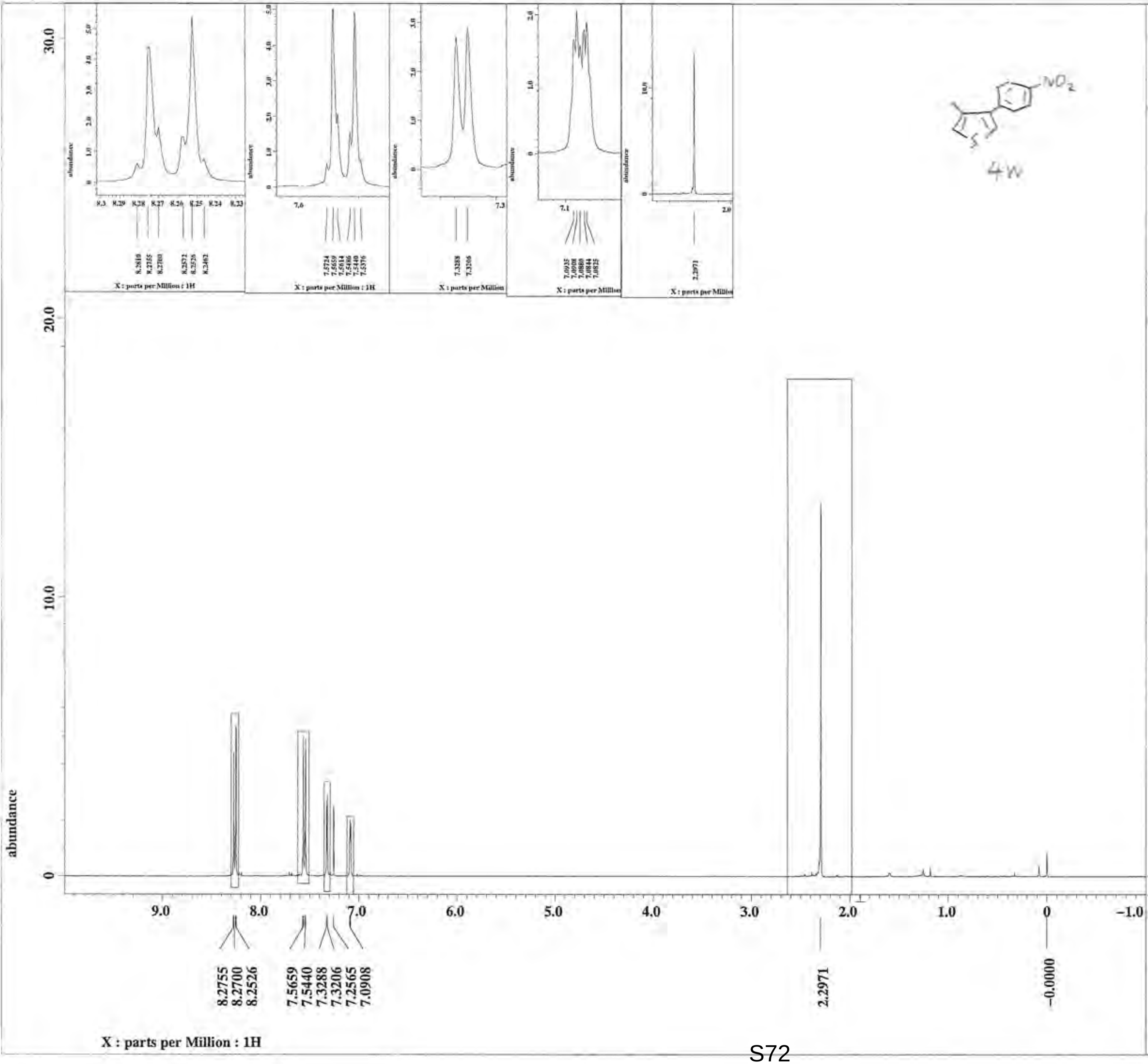


```
---- PROCESSING PARAMETERS ----
dc_balance : 0 : FALSE
sexp : 0.2[Hz] : 0.0[s]
trapezoid3 : 0[%] : 80[%] : 100[%]
zerofill : 1
fft : 1 : TRUE : TRUE
machinephase
ppm

Derived from: UKI-225-A proton-1.jdf

Filename           = UKI-225-A proton-10.j
Author             = element
Experiment         = single_pulse.ex2
Sample_id          = S#821325
Solvent            = CHLOROFORM-D
Creation_time      = 27-AUG-2014 22:39:54
Revision_time      = 11-SEP-2014 16:30:17
Current_time       = 11-SEP-2014 16:30:19

Comment            = single_pulse
Data_format        = 1D COMPLEX
Dim_size           = 16384
Dim_title          = 1H
Dim_units          = [ppm]
Dimensions         = X
Site               = ECS 400
Spectrometer       = JNM-ECS400

Field_strength     = 9.20197068[T] (390[MH
X_acq_duration     = 2.78790144[s]
X_domain           = 1H
X_freq             = 391.78655441[MHz]
X_offset           = 5[ppm]
X_points           = 16384
X_prescans         = 1
X_resolution       = 0.35869274[Hz]
X_sweep            = 5.87682181[kHz]
Irr_domain         = 1H
Irr_freq           = 391.78655441[MHz]
Irr_offset         = 5[ppm]
Tri_domain         = 1H
Tri_freq           = 391.78655441[MHz]
Tri_offset         = 5[ppm]
Clipped            = FALSE
Mod_return         = 1
Scans              = 10
Total_scans        = 10

X_90_width         = 10.7[us]
X_acq_time         = 2.78790144[s]
X_angle            = 45[deg]
X_atn              = 1.9[dB]
X_pulse            = 5.35[us]
Irr_mode           = Off
Tri_mode           = Off
Dante_presat       = FALSE
Initial_wait       = 1[s]
Recvr_gain         = 36
Relaxation_delay   = 4[s]
Repetition_time    = 6.78790144[s]
Temp_get           = 21.5[dC]
```

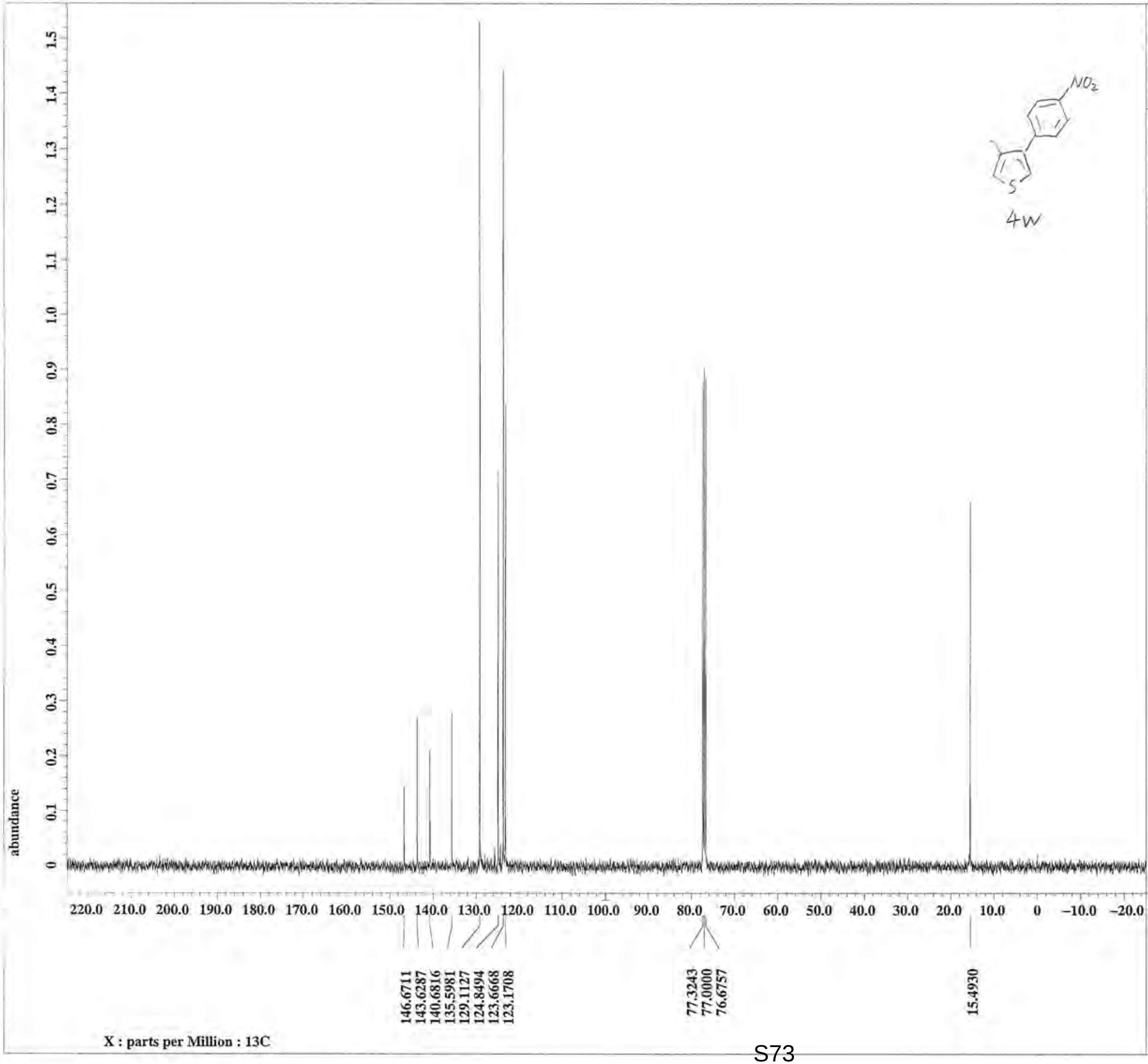

JEOL

```
---- PROCESSING PARAMETERS ----
dc_balance : 0 : FALSE
sexp : 2.0[Hz] : 0.0[s]
trapezoid3 : 0[%] : 80[%] : 100[%]
zerofill : 1
fft : 1 : TRUE : TRUE
machinephase
ppm

Derived from: UKI-225-B carbon-1.jdf

Filename          = UKI-225-B carbon-4.jd
Author            = element
Experiment        = single_pulse_dec
Sample_id         = 225
Solvent           = CHLOROFORM-D
Creation_time     = 27-AUG-2014 22:22:14
Revision_time     = 27-AUG-2014 22:42:43
Current_time      = 27-AUG-2014 22:50:39

Comment           = single pulse decouple
Data_format       = 1D COMPLEX
Dim_size          = 26214
Dim_title         = 13C
Dim_units         = [ppm]
Dimensions        = X
Site              = ECS 400
Spectrometer      = JNM-ECS400

Field_strength    = 9.20197068[T] (390[MH
X_acq_duration    = 1.06430464[s]
X_domain          = 13C
X_freq            = 98.51479726[MHz]
X_offset          = 100[ppm]
X_points          = 32768
X_prescans        = 4
X_resolution      = 0.93958061[Hz]
X_sweep           = 30.78817734[kHz]
Irr_domain        = 1H
Irr_freq          = 391.78655441[MHz]
Irr_offset        = 5[ppm]
Clipped           = FALSE
Mod_return        = 1
Scans             = 150
Total_scans       = 150

X_90_width        = 8.8[us]
X_acq_time        = 1.06430464[s]
X_angle           = 30[deg]
X_atn             = 4.9[dB]
X_pulse           = 2.93333333[us]
Irr_atn_dec       = 22.52628[dB]
Irr_atn_noe       = 22.52628[dB]
Irr_noise         = WALTZ
Decoupling        = TRUE
Initial_wait      = 1[s]
Noe               = TRUE
Noe_time          = 2[s]
Recvr_gain        = 60
Relaxation_delay  = 2[s]
Repetition_time   = 3.06430464[s]
Temp_get          = 21[dC]
```

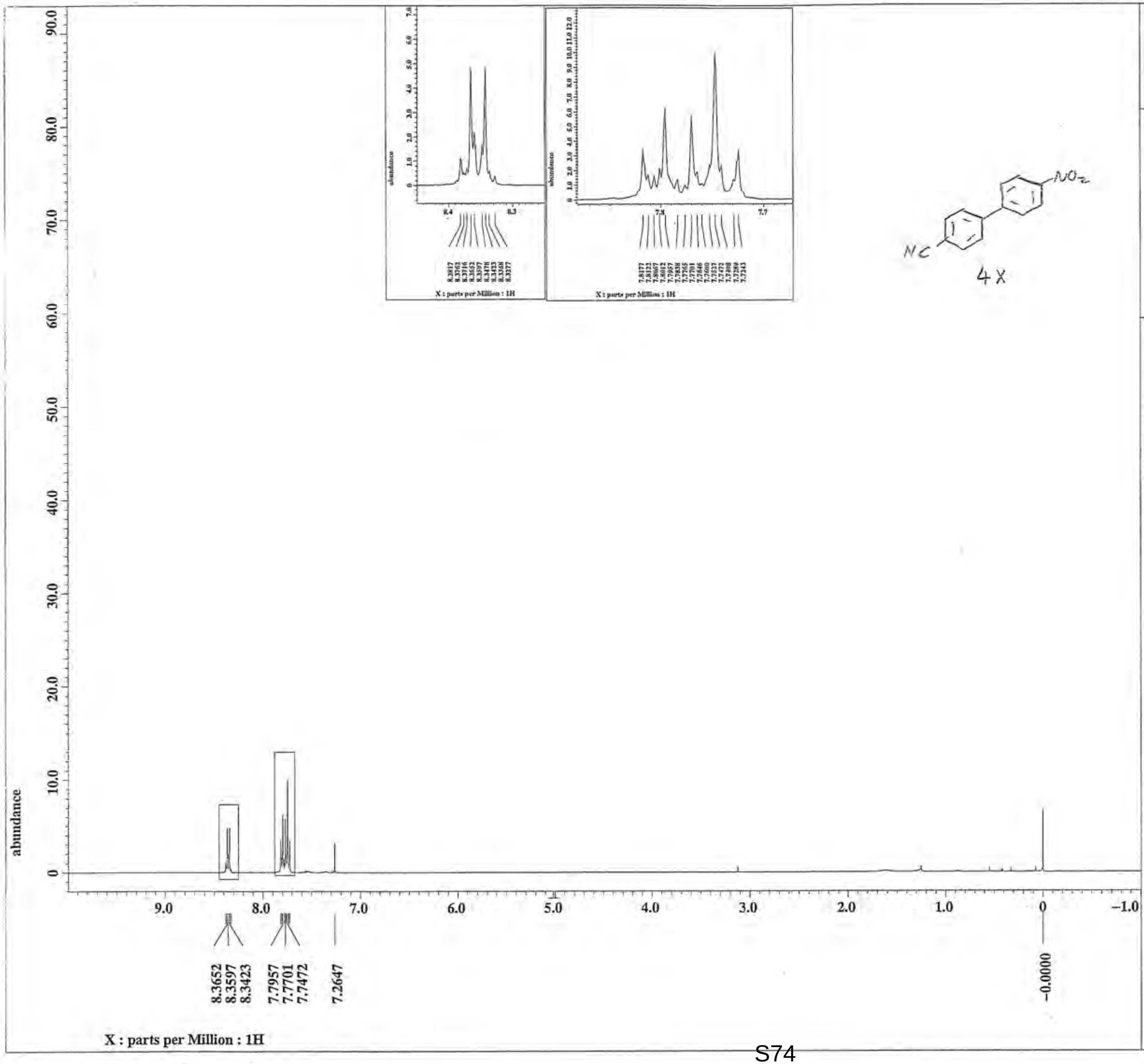

JEOL

---- PROCESSING PARAMETERS ----
dc_balance : 0 : FALSE
sexp : 0.2[Hz] : 0.0[s]
trapezoid3 : 0[%] : 80[%] : 100[%]
zerofill : 1
fft : 1 : TRUE : TRUE
machinephase
ppm

Derived from: UKI-192-A proton-1.jdf

Filename	= UKI-192-A proton-6.jd
Author	= element
Experiment	= single_pulse.ex2
Sample_id	= 192
Solvent	= CHLOROFORM-D
Creation_time	= 4-SEP-2014 18:08:35
Revision_time	= 4-SEP-2014 18:28:34
Current_time	= 4-SEP-2014 18:28:36
Comment	= single_pulse
Data_format	= 1D COMPLEX
Dim_size	= 16384
Dim_title	= 1H
Dim_units	= [ppm]
Dimensions	= X
Site	= ECS 400
Spectrometer	= JNM-ECS400
Field_strength	= 9.20197068[T] (390[MH
X_acq_duration	= 2.78790144[s]
X_domain	= 1H
X_freq	= 391.78655441[MHz]
X_offset	= 5[ppm]
X_points	= 16384
X_prescans	= 1
X_resolution	= 0.35869274[Hz]
X_sweep	= 5.87682181[kHz]
Irr_domain	= 1H
Irr_freq	= 391.78655441[MHz]
Irr_offset	= 5[ppm]
Tri_domain	= 1H
Tri_freq	= 391.78655441[MHz]
Tri_offset	= 5[ppm]
Clipped	= FALSE
Mod_return	= 1
Scans	= 20
Total_scans	= 20
X_90_width	= 10.7[us]
X_acq_time	= 2.78790144[s]
X_angle	= 45[deg]
X_atn	= 1.9[dB]
X_pulse	= 5.35[us]
Irr_mode	= Off
Tri_mode	= Off
Dante_presat	= FALSE
Initial_wait	= 1[s]
Recvr_gain	= 50
Relaxation_delay	= 4[s]
Repetition_time	= 6.78790144[s]
Temp_get	= 20.5[dC]

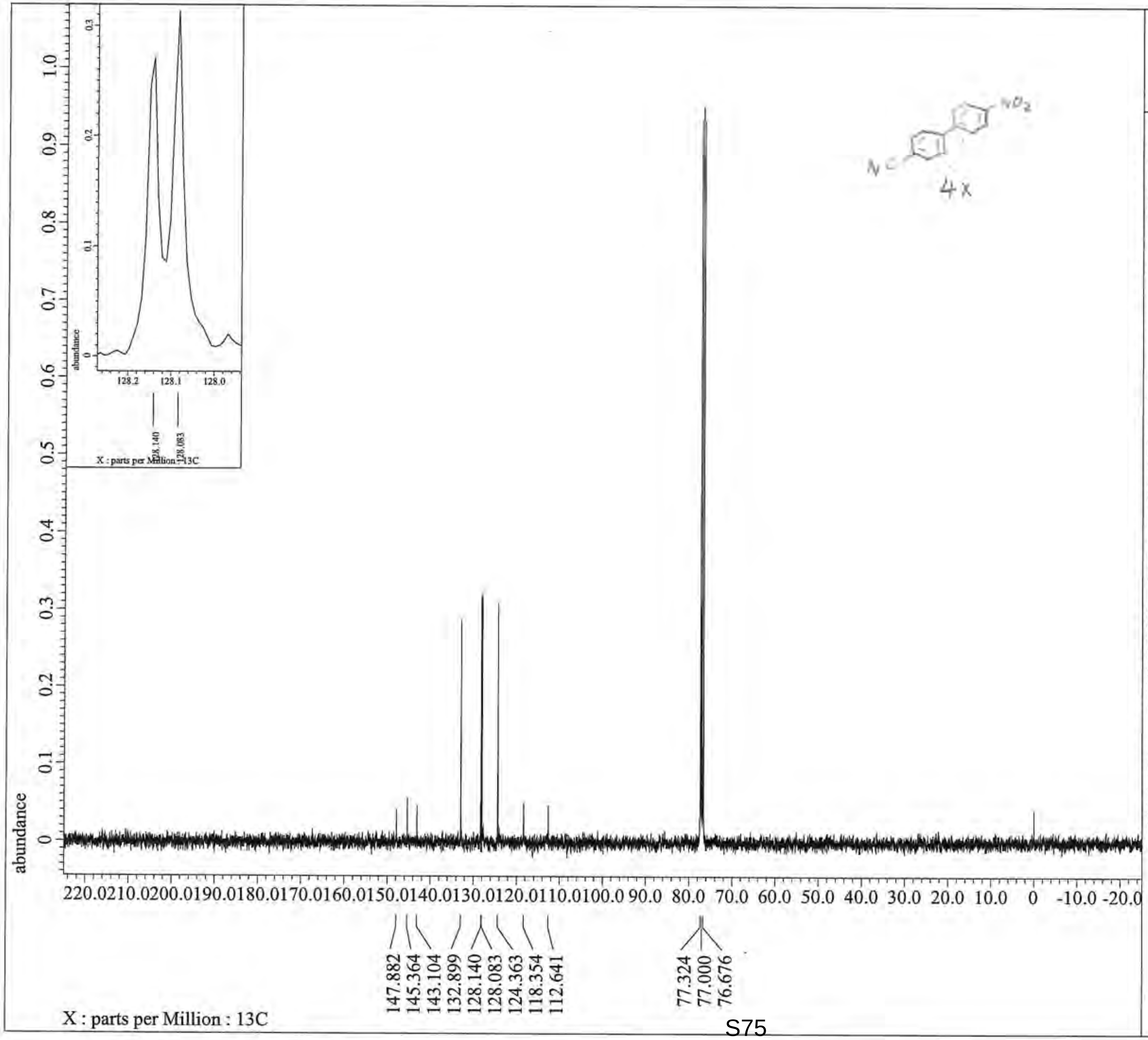

JEOL RESONANCE

Filename	= UKI-192-B carbon-4.jdf
Author	= element
Experiment	= single_pulse_dec
Sample_Id	= 192
Solvent	= CHLOROFORM-D
Creation_Time	= 4-SEP-2014 18:29:38
Revision_Time	= 5-DEC-2014 14:41:15
Current_Time	= 5-DEC-2014 14:41:24
Comment	= single pulse decoupled gat
Data_Format	= 1D COMPLEX
Dim_Size	= 26214
Dim_Title	= 13C
Dim_Units	= [ppm]
Dimensions	= X
Site	= ECS 400
Spectrometer	= JNM-ECS400
Field_Strength	= 9.20197068[T] (390[MHz])
X_Acq_Duration	= 1.06430464[s]
X_Domain	= 13C
X_Freq	= 98.51479726[MHz]
X_Offset	= 100[ppm]
X_Points	= 32768
X_Prescans	= 4
X_Resolution	= 0.93958061[Hz]
X_Sweep	= 30.78817734[kHz]
Irr_Domain	= 1H
Irr_Freq	= 391.78655441[MHz]
Irr_Offset	= 5[ppm]
Clipped	= FALSE
Scans	= 200
Total_Scans	= 200
Relaxation_Delay	= 2[s]
Recvr_Gain	= 60
Temp_Get	= 20.8[dC]
X_90_Width	= 8.8[us]
X_Acq_Time	= 1.06430464[s]
X_Angle	= 30[deg]
X_Atn	= 4.9[dB]
X_Pulse	= 2.93333333[us]
Irr_Atn_Dec	= 22.52628[dB]
Irr_Atn_Noe	= 22.52628[dB]
Irr_Noise	= WALTZ
Decoupling	= TRUE
Initial_Wait	= 1[s]
Noe	= TRUE
Noe_Time	= 2[s]
Repetition_Time	= 3.06430464[s]

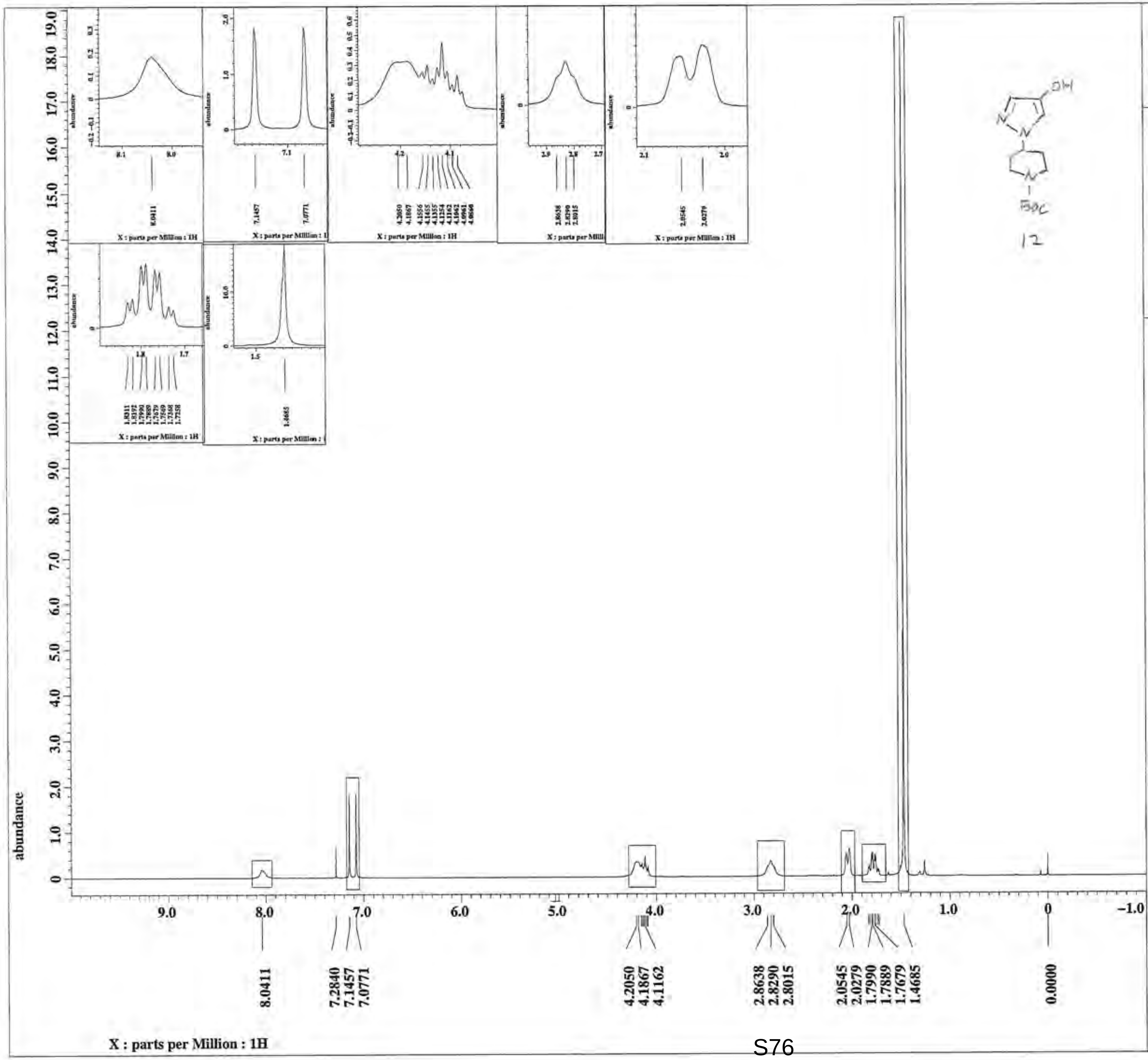

12

---- PROCESSING PARAMETERS ----
dc_balance : 0 : FALSE
sexp : 0.2[Hz] : 0.0[s]
trapezoid3 : 0[%] : 80[%] : 100[%]
zerofill : 1
fft : 1 : TRUE : TRUE
machinephase
ppm

Derived from: UKI-221-A proton-1.jdf

Filename	= UKI-221-A proton-4.jd
Author	= element
Experiment	= single_pulse.ex2
Sample_id	= 221
Solvent	= CHLOROFORM-D
Creation_time	= 2-SEP-2014 13:21:09
Revision_time	= 24-OCT-2014 17:44:24
Current_time	= 24-OCT-2014 17:49:20
Comment	= single_pulse
Data_format	= 1D COMPLEX
Dim_size	= 16384
Dim_title	= 1H
Dim_units	= [ppm]
Dimensions	= X
Site	= ECS 400
Spectrometer	= JNM-ECS400
Field_strength	= 9.20197068[T] (390[MH
X_acq_duration	= 2.78790144[s]
X_domain	= 1H
X_freq	= 391.78655441[MHz]
X_offset	= 5[ppm]
X_points	= 16384
X_prescans	= 1
X_resolution	= 0.35869274[Hz]
X_sweep	= 5.87682181[kHz]
Irr_domain	= 1H
Irr_freq	= 391.78655441[MHz]
Irr_offset	= 5[ppm]
Tri_domain	= 1H
Tri_freq	= 391.78655441[MHz]
Tri_offset	= 5[ppm]
Clipped	= FALSE
Mod_return	= 1
Scans	= 10
Total_scans	= 10
X_90_width	= 10.7[us]
X_acq_time	= 2.78790144[s]
X_angle	= 45[deg]
X_atn	= 1.9[dB]
X_pulse	= 5.35[us]
Irr_mode	= Off
Tri_mode	= Off
Dante_presat	= FALSE
Initial_wait	= 1[s]
Recvr_gain	= 30
Relaxation_delay	= 4[s]
Repetition_time	= 6.78790144[s]
Temp_get	= 21.1[dC]

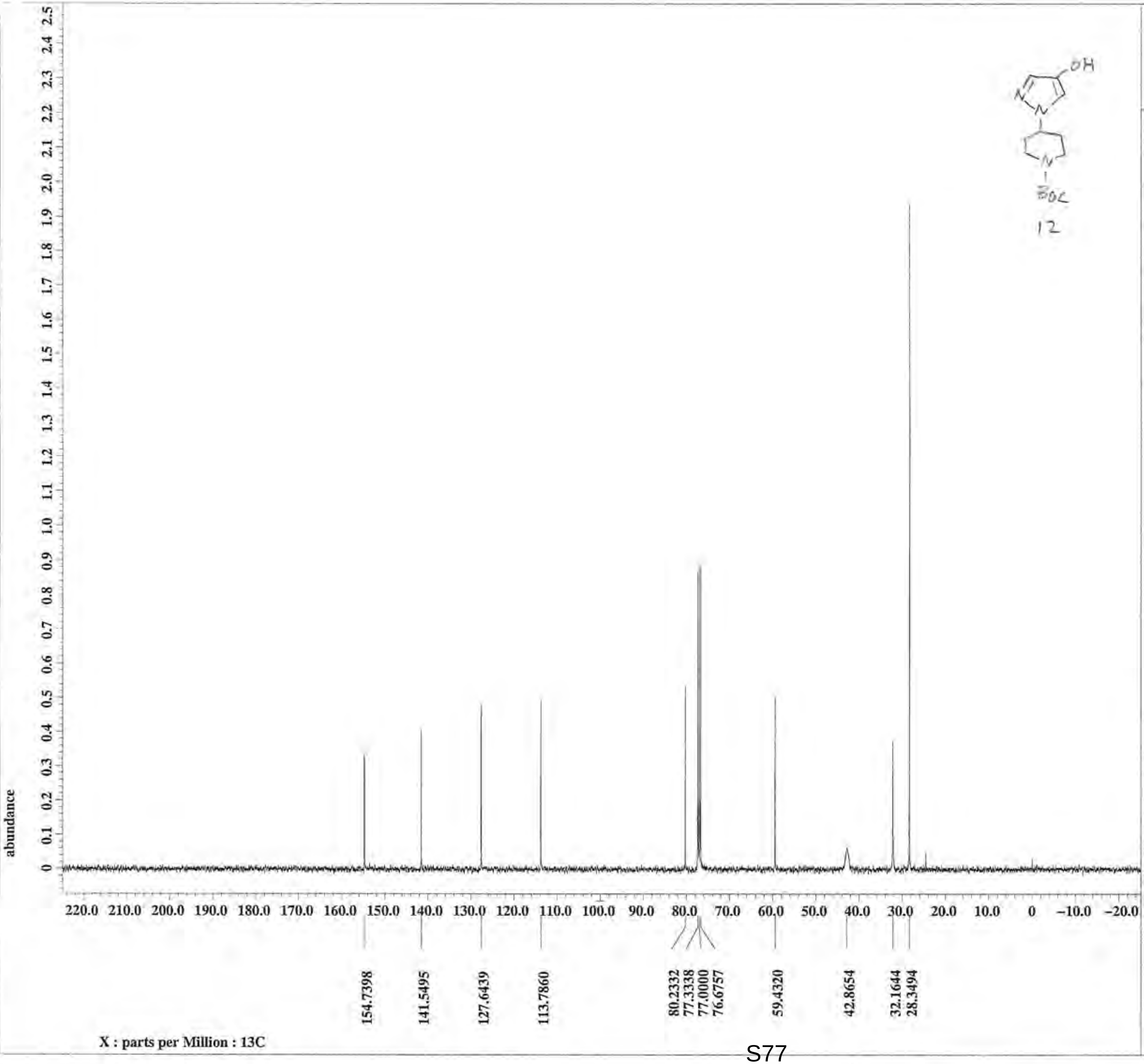

---- PROCESSING PARAMETERS ----
dc_balance : 0 : FALSE
sexp : 2.0[Hz] : 0.0[s]
trapezoid3 : 0[%] : 80[%] : 100[%]
zerofill : 1
fft : 1 : TRUE : TRUE
machinephase
ppm

Derived from: UKI-221-B carbon-1.jdf

Filename = UKI-221-B carbon-3.jd
Author = element
Experiment = single_pulse_dec
Sample_id = 221
Solvent = CHLOROFORM-D
Creation_time = 2-SEP-2014 13:32:36
Revision_time = 2-SEP-2014 13:47:33
Current_time = 2-SEP-2014 13:48:04

Comment = single pulse decouple
Data_format = 1D COMPLEX
Dim_size = 26214
Dim_title = 13C
Dim_units = [ppm]
Dimensions = X
Site = ECS 400
Spectrometer = JNM-ECS400

Field_strength = 9.20197068[T] (390[MH
X_acq_duration = 1.06430464[s]
X_domain = 13C
X_freq = 98.51479726[MHz]
X_offset = 100[ppm]
X_points = 32768
X_prescans = 4
X_resolution = 0.93958061[Hz]
X_sweep = 30.78817734[kHz]
Irr_domain = 1H
Irr_freq = 391.78655441[MHz]
Irr_offset = 5[ppm]
Clipped = FALSE
Mod_return = 1
Scans = 200
Total_scans = 200

X_90_width = 8.8[us]
X_acq_time = 1.06430464[s]
X_angle = 30[deg]
X_atn = 4.9[dB]
X_pulse = 2.93333333[us]
Irr_atn_dec = 22.52628[dB]
Irr_atn_noe = 22.52628[dB]
Irr_noise = WALTZ
Decoupling = TRUE
Initial_wait = 1[s]
Noe = TRUE
Noe_time = 2[s]
Recvr_gain = 60
Relaxation_delay = 2[s]
Repetition_time = 3.06430464[s]
Temp_get = 21.3[dC]

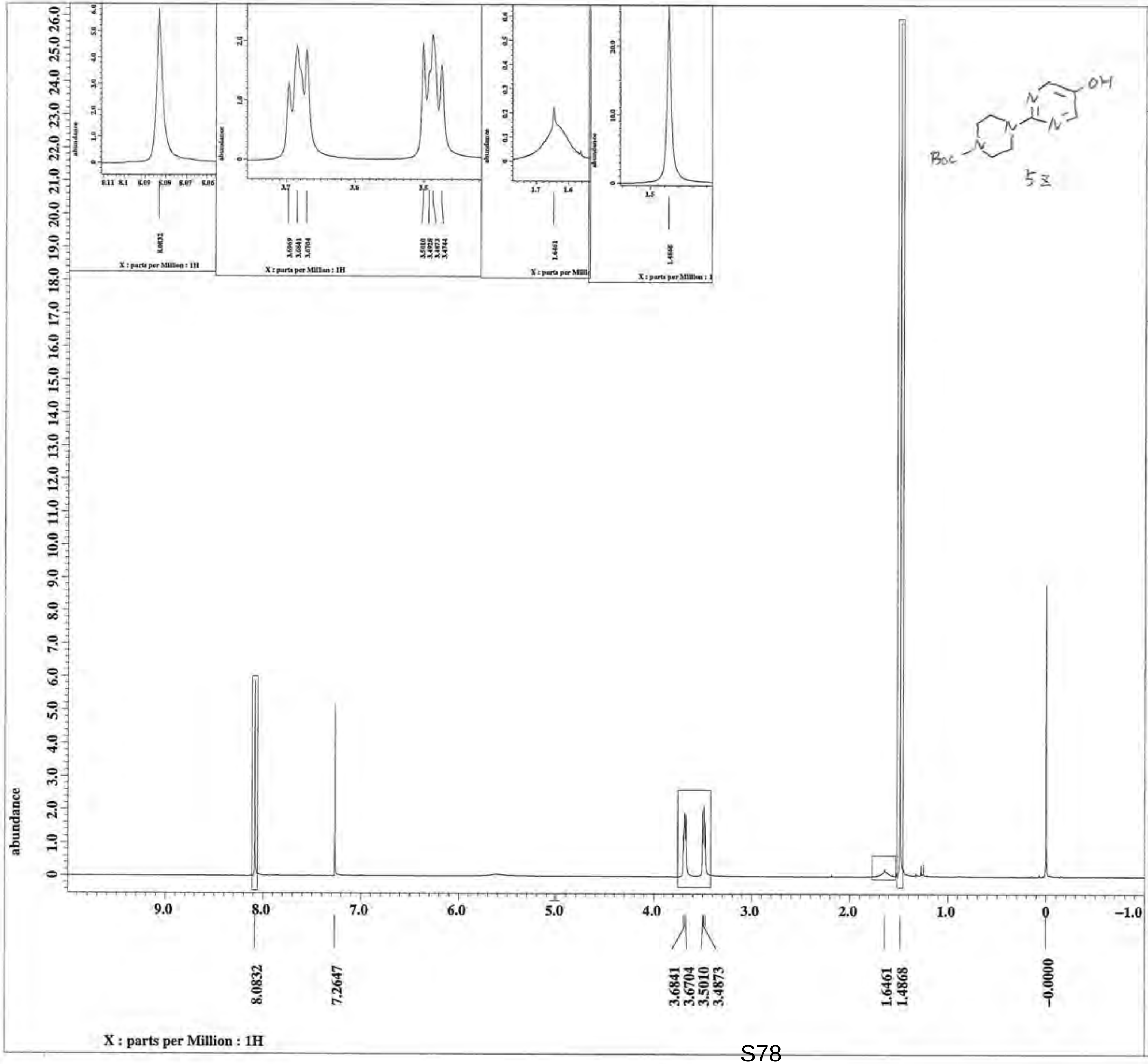

---- PROCESSING PARAMETERS ----
dc_balance : 0 : FALSE
sexp : 0.2[Hz] : 0.0[s]
trapezoid3 : 0[%] : 80[%] : 100[%]
zerofill : 1
fft : 1 : TRUE : TRUE
machinephase
ppm

Derived from: UKI-206-A proton-1.jdf

Filename	= UKI-206-A proton-6.jd
Author	= element
Experiment	= single_pulse.ex2
Sample_id	= 206
Solvent	= CHLOROFORM-D
Creation_time	= 2-JUL-2014 20:36:00
Revision_time	= 29-NOV-2014 16:27:23
Current_time	= 29-NOV-2014 16:27:24
Comment	= single_pulse
Data_format	= 1D COMPLEX
Dim_size	= 16384
Dim_title	= 1H
Dim_units	= [ppm]
Dimensions	= X
Site	= ECS 400
Spectrometer	= JNM-ECS400
Field_strength	= 9.20197068[T] (390[MH
X_acq_duration	= 2.78790144[s]
X_domain	= 1H
X_freq	= 391.78655441[MHz]
X_offset	= 5[ppm]
X_points	= 16384
X_prescans	= 1
X_resolution	= 0.35869274[Hz]
X_sweep	= 5.87682181[kHz]
Irr_domain	= 1H
Irr_freq	= 391.78655441[MHz]
Irr_offset	= 5[ppm]
Tri_domain	= 1H
Tri_freq	= 391.78655441[MHz]
Tri_offset	= 5[ppm]
Clipped	= FALSE
Mod_return	= 1
Scans	= 15
Total_scans	= 15
X_90_width	= 10.7[us]
X_acq_time	= 2.78790144[s]
X_angle	= 45[deg]
X_atn	= 1.9[dB]
X_pulse	= 5.35[us]
Irr_mode	= Off
Tri_mode	= Off
Dante_presat	= FALSE
Initial_wait	= 1[s]
Recvr_gain	= 52
Relaxation_delay	= 4[s]
Repetition_time	= 6.78790144[s]
Temp_get	= 19.9[dC]

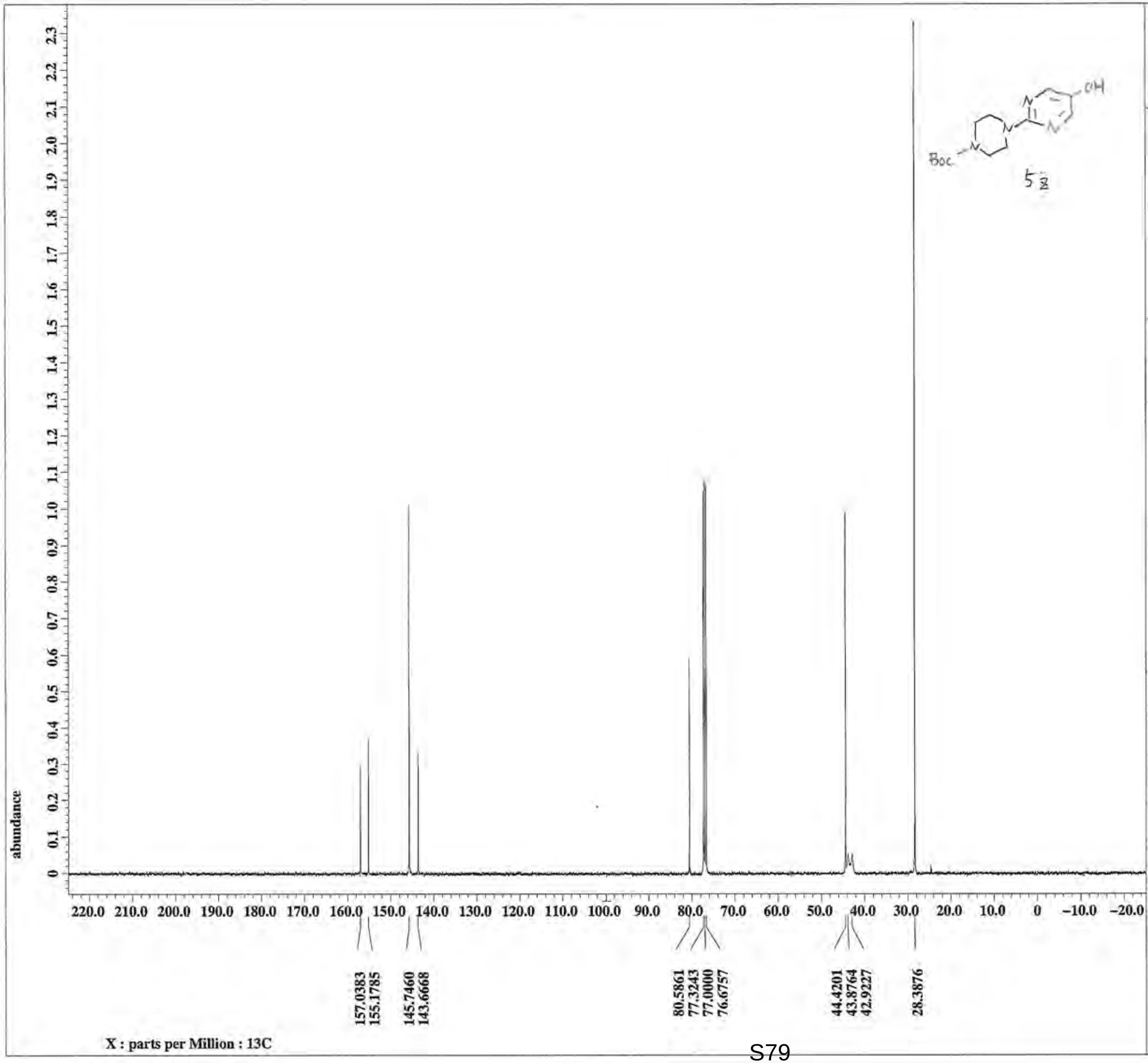


```
---- PROCESSING PARAMETERS ----
dc_balance : 0 : FALSE
sexp : 2.0[Hz] : 0.0[s]
trapezoid3 : 0[%] : 80[%] : 100[%]
zerofill : 1
fft : 1 : TRUE : TRUE
machinephase
ppm

Derived from: UKI-206-B carbon2-1.jdf

Filename          = UKI-206-B carbon2-5.j
Author            = element
Experiment        = single_pulse_dec
Sample_id         = S#445404
Solvent           = CHLOROFORM-D
Creation_time     = 15-DEC-2014 12:39:54
Revision_time     = 15-DEC-2014 13:08:12
Current_time      = 15-DEC-2014 13:08:21

Comment           = single pulse decouple
Data_format       = 1D COMPLEX
Dim_size          = 26214
Dim_title         = 13C
Dim_units         = [ppm]
Dimensions        = X
Site              = ECS 400
Spectrometer      = JNM-ECS400

Field_strength    = 9.20197068[T] (390[MH
X_acq_duration    = 1.06430464[s]
X_domain          = 13C
X_freq            = 98.51479726[MHz]
X_offset          = 100[ppm]
X_points          = 32768
X_prescans        = 4
X_resolution      = 0.93958061[Hz]
X_sweep           = 30.78817734[kHz]
Irr_domain        = 1H
Irr_freq          = 391.78655441[MHz]
Irr_offset        = 5[ppm]
Clipped           = FALSE
Mod_return        = 1
Scans             = 800
Total_scans       = 800

X_90_width        = 8.8[us]
X_acq_time        = 1.06430464[s]
X_angle           = 30[deg]
X_atn             = 4.9[dB]
X_pulse           = 2.93333333[us]
Irr_atn_dec       = 22.52628[dB]
Irr_atn_noe       = 22.52628[dB]
Irr_noise         = WALTZ
Decoupling        = TRUE
Initial_wait      = 1[s]
Noe               = TRUE
Noe_time          = 2[s]
Recvr_gain        = 60
Relaxation_delay  = 2[s]
Repetition_time   = 3.06430464[s]
Temp_get          = 19.2[dC]
```

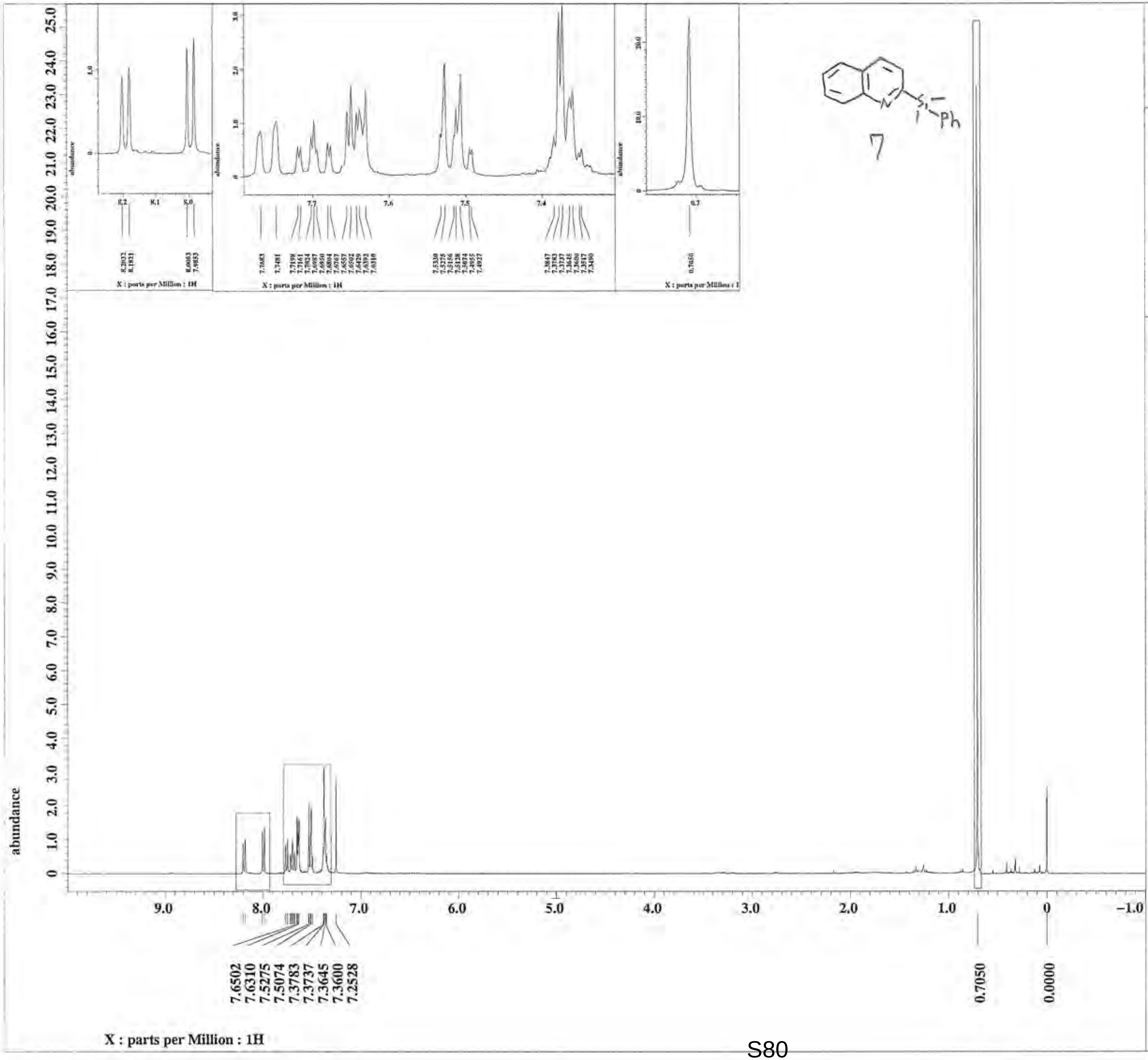

---- PROCESSING PARAMETERS ----
dc_balance : 0 : FALSE
sexp : 0.2[Hz] : 0.0[s]
trapezoid3 : 0[%] : 80[%] : 100[%]
zerofill : 1
fft : 1 : TRUE : TRUE
machinephase
ppm

Derived from: UKI-173-3 H-1.jdf

Filename	= UKI-173-3 H-5.jdf
Author	= element
Experiment	= single_pulse.ex2
Sample_id	= S#519505
Solvent	= CHLOROFORM-D
Creation_time	= 24-MAR-2014 13:13:12
Revision_time	= 11-SEP-2014 16:02:24
Current_time	= 11-SEP-2014 16:02:25
Comment	= single_pulse
Data_format	= 1D COMPLEX
Dim_size	= 16384
Dim_title	= 1H
Dim_units	= [ppm]
Dimensions	= X
Site	= ECS 400
Spectrometer	= JNM-ECS400
Field_strength	= 9.20197068[T] (390[MH
X_acq_duration	= 2.78790144[s]
X_domain	= 1H
X_freq	= 391.78655441[MHz]
X_offset	= 5[ppm]
X_points	= 16384
X_prescans	= 1
X_resolution	= 0.35869274[Hz]
X_sweep	= 5.87682181[kHz]
Irr_domain	= 1H
Irr_freq	= 391.78655441[MHz]
Irr_offset	= 5[ppm]
Tri_domain	= 1H
Tri_freq	= 391.78655441[MHz]
Tri_offset	= 5[ppm]
Clipped	= FALSE
Mod_return	= 1
Scans	= 16
Total_scans	= 16
X_90_width	= 10.8[us]
X_acq_time	= 2.78790144[s]
X_angle	= 45[deg]
X_atn	= 1.9[dB]
X_pulse	= 5.4[us]
Irr_mode	= Off
Tri_mode	= Off
Dante_presat	= FALSE
Initial_wait	= 1[s]
Recvr_gain	= 46
Relaxation_delay	= 4[s]
Repetition_time	= 6.78790144[s]
Temp_get	= 20.2[dC]

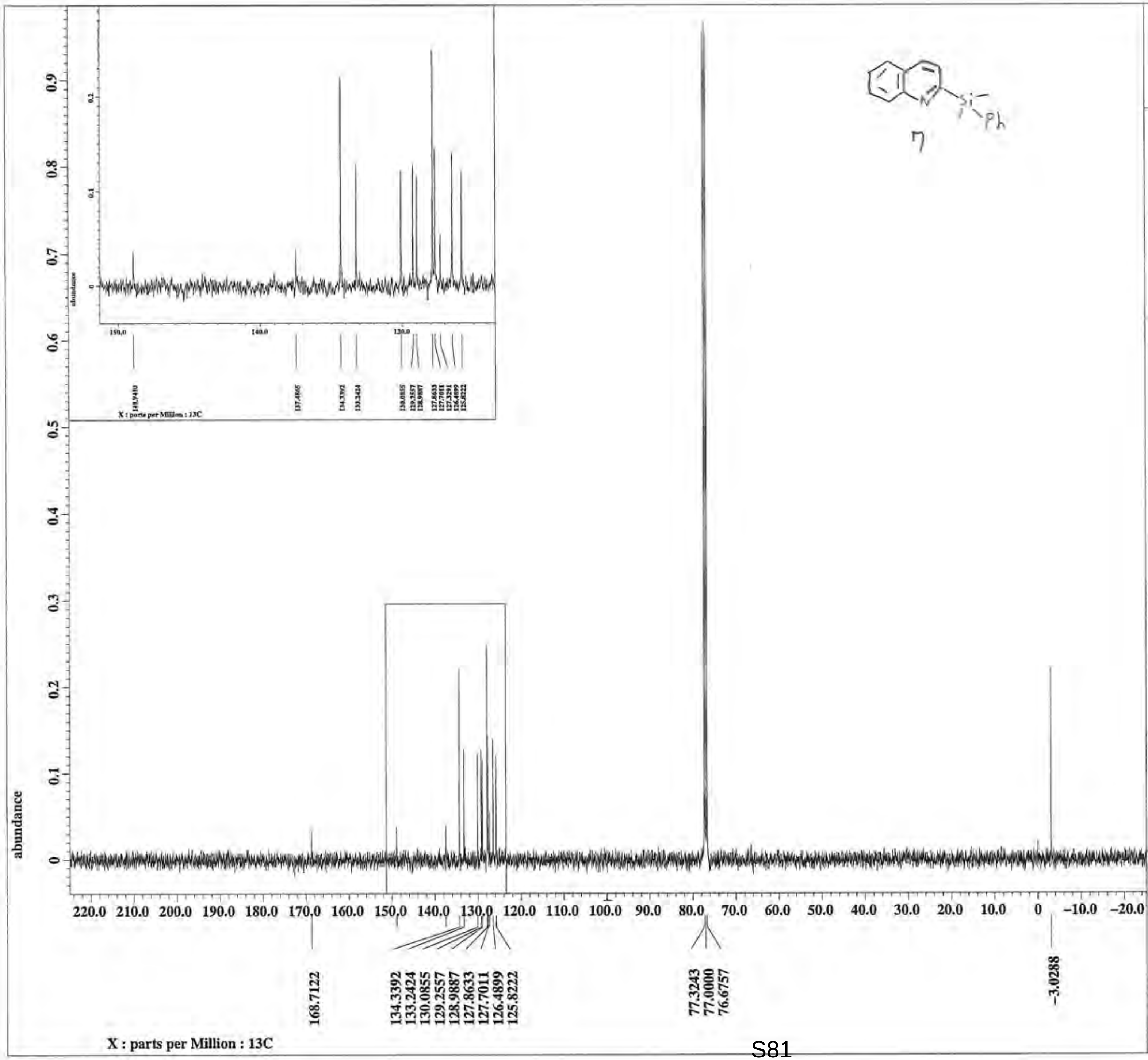

---- PROCESSING PARAMETERS ----
dc_balance : 0 : FALSE
sexp : 2.0[Hz] : 0.0[s]
trapezoid3 : 0[%] : 80[%] : 100[%]
zerofill : 1
fft : 1 : TRUE : TRUE
machinephase
ppm

Derived from: UKI-173-5 C-1.jdf

Filename	= UKI-173-5 C-8.jdf
Author	= element
Experiment	= single_pulse_dec
Sample_id	= S#521510
Solvent	= CHLOROFORM-D
Creation_time	= 24-MAR-2014 13:24:30
Revision_time	= 24-OCT-2014 17:36:45
Current_time	= 24-OCT-2014 17:36:55
Comment	= single pulse decouple
Data_format	= 1D COMPLEX
Dim_size	= 26214
Dim_title	= 13C
Dim_units	= [ppm]
Dimensions	= X
Site	= ECS 400
Spectrometer	= JNM-ECS400
Field_strength	= 9.20197068[T] (390[MH
X_acq_duration	= 1.06430464[s]
X_domain	= 13C
X_freq	= 98.51479726[MHz]
X_offset	= 100[ppm]
X_points	= 32768
X_prescans	= 4
X_resolution	= 0.93958061[Hz]
X_sweep	= 30.78817734[kHz]
Irr_domain	= 1H
Irr_freq	= 391.78655441[MHz]
Irr_offset	= 5[ppm]
Clipped	= FALSE
Mod_return	= 1
Scans	= 200
Total_scans	= 200
X_90_width	= 8.15[us]
X_acq_time	= 1.06430464[s]
X_angle	= 30[deg]
X_atn	= 4.9[dB]
X_pulse	= 2.71666667[us]
Irr_atn_dec	= 22.445[dB]
Irr_atn_noe	= 22.445[dB]
Irr_noise	= WALTZ
Decoupling	= TRUE
Initial_wait	= 1[s]
Noe	= TRUE
Noe_time	= 2[s]
Recvr_gain	= 60
Relaxation_delay	= 2[s]
Repetition_time	= 3.06430464[s]
Temp_get	= 19.9[dC]

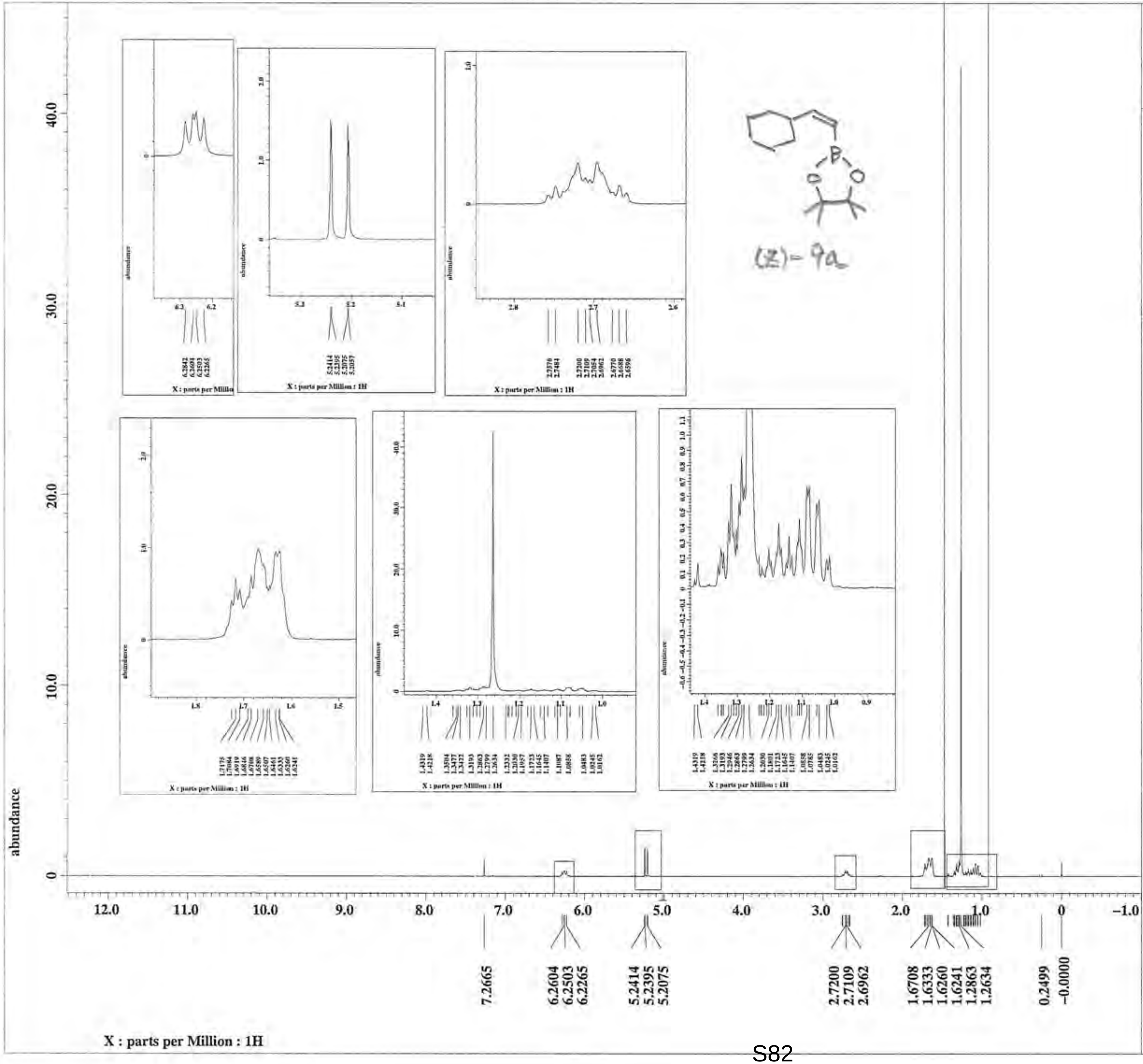


```
---- PROCESSING PARAMETERS ----
dc_balance : 0 : FALSE
sexp : 0.2[Hz] : 0.0[s]
trapezoid3 : 0[%] : 80[%] : 100[%]
zerofill : 1
fft : 1 : TRUE : TRUE
machinephase
ppm

Derived from: EIYA402P-1.jdf

Filename          = EIYA402P-4.jdf
Author            = element
Experiment        = single_pulse.ex2
Sample_id         = S#848036
Solvent           = CHLOROFORM-D
Creation_time     = 11-NOV-2013 22:55:33
Revision_time     = 11-NOV-2013 23:47:07
Current_time      = 11-NOV-2013 23:48:23

Comment           = single_pulse
Data_format       = 1D COMPLEX
Dim_size          = 16384
Dim_title         = 1H
Dim_units         = [ppm]
Dimensions        = X
Site              = ECX 400P
Spectrometer      = DELTA2_NMR

Field_strength    = 9.2982153[T] (400[MHz
X_acq_duration    = 2.7590656[s]
X_domain          = 1H
X_freq            = 395.88430144[MHz]
X_offset          = 5[ppm]
X_points          = 16384
X_prescans        = 1
X_resolution      = 0.36244155[Hz]
X_sweep           = 5.93824228[kHz]
Irr_domain        = 1H
Irr_freq          = 395.88430144[MHz]
Irr_offset        = 5[ppm]
Tri_domain        = 1H
Tri_freq          = 395.88430144[MHz]
Tri_offset        = 5[ppm]
Clipped           = FALSE
Mod_return        = 1
Scans             = 8
Total_scans       = 8

X_90_width        = 11.8[us]
X_acq_time        = 2.7590656[s]
X_angle           = 45[deg]
X_atn             = 1[dB]
X_pulse           = 5.9[us]
Irr_mode          = Off
Tri_mode          = Off
Dante_presat      = FALSE
Initial_wait      = 1[s]
Recvr_gain        = 34
Relaxation_delay  = 3[s]
Repetition_time   = 5.7590656[s]
Temp_get          = 402.4[dC]
```

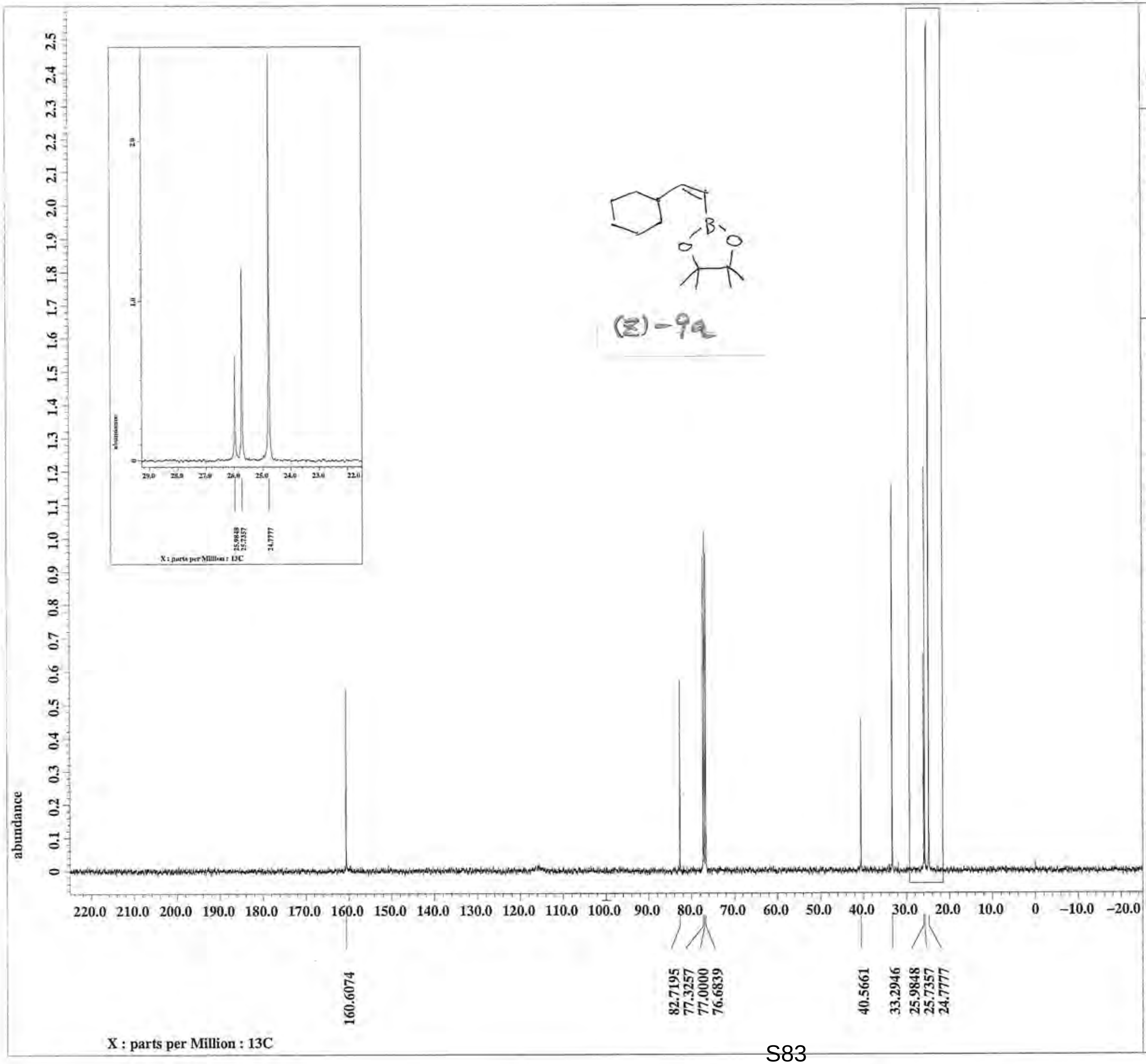

JEOL

```
---- PROCESSING PARAMETERS ----
dc_balance : 0 : FALSE
sexp : 2.0[Hz] : 0.0[s]
trapezoid3 : 0[%] : 80[%] : 100[%]
zerofill : 1
fft : 1 : TRUE : TRUE
machinephase
ppm

Derived from: EIYA402P C_copy-1.jdf
```

```
Filename          = EIYA402P C_copy-3.jdf
Author            = element
Experiment        = single_pulse_dec
Sample_id         = S#849345
Solvent           = CHLOROFORM-D
Creation_time     = 11-NOV-2013 23:08:44
Revision_time     = 11-NOV-2013 23:49:17
Current_time      = 11-NOV-2013 23:50:09

Comment           = single pulse decouple
Data_format       = 1D COMPLEX
Dim_size          = 26214
Dim_title         = 13C
Dim_units         = [ppm]
Dimensions        = X
Site              = ECX 400P
Spectrometer      = DELTA2_NMR

Field_strength    = 9.2982153[T] (400[MHz
X_acq_duration    = 1.048576[s]
X_domain          = 13C
X_freq            = 99.54517646[MHz]
X_offset          = 100[ppm]
X_points          = 32768
X_prescans        = 4
X_resolution      = 0.95367432[Hz]
X_sweep           = 31.25[kHz]
Irr_domain        = 1H
Irr_freq          = 395.88430144[MHz]
Irr_offset        = 5[ppm]
Clipped           = TRUE
Incomplete_copy   = TRUE
Mod_return        = 1
Scans             = 234
Total_scans       = 234

X_90_width        = 9.3[us]
X_acq_time        = 1.048576[s]
X_angle           = 30[deg]
X_atn             = 3.4[dB]
X_pulse           = 3.1[us]
Irr_atn_dec       = 19.515[dB]
Irr_atn_noe       = 19.515[dB]
Irr_noise         = WALTZ
Decoupling        = TRUE
Initial_wait      = 1[s]
Noe               = TRUE
Noe_time          = 2[s]
Recvr_gain        = 58
Relaxation_delay  = 2[s]
Repetition_time   = 3.048576[s]
Temp_get          = 402.4[dC]
```

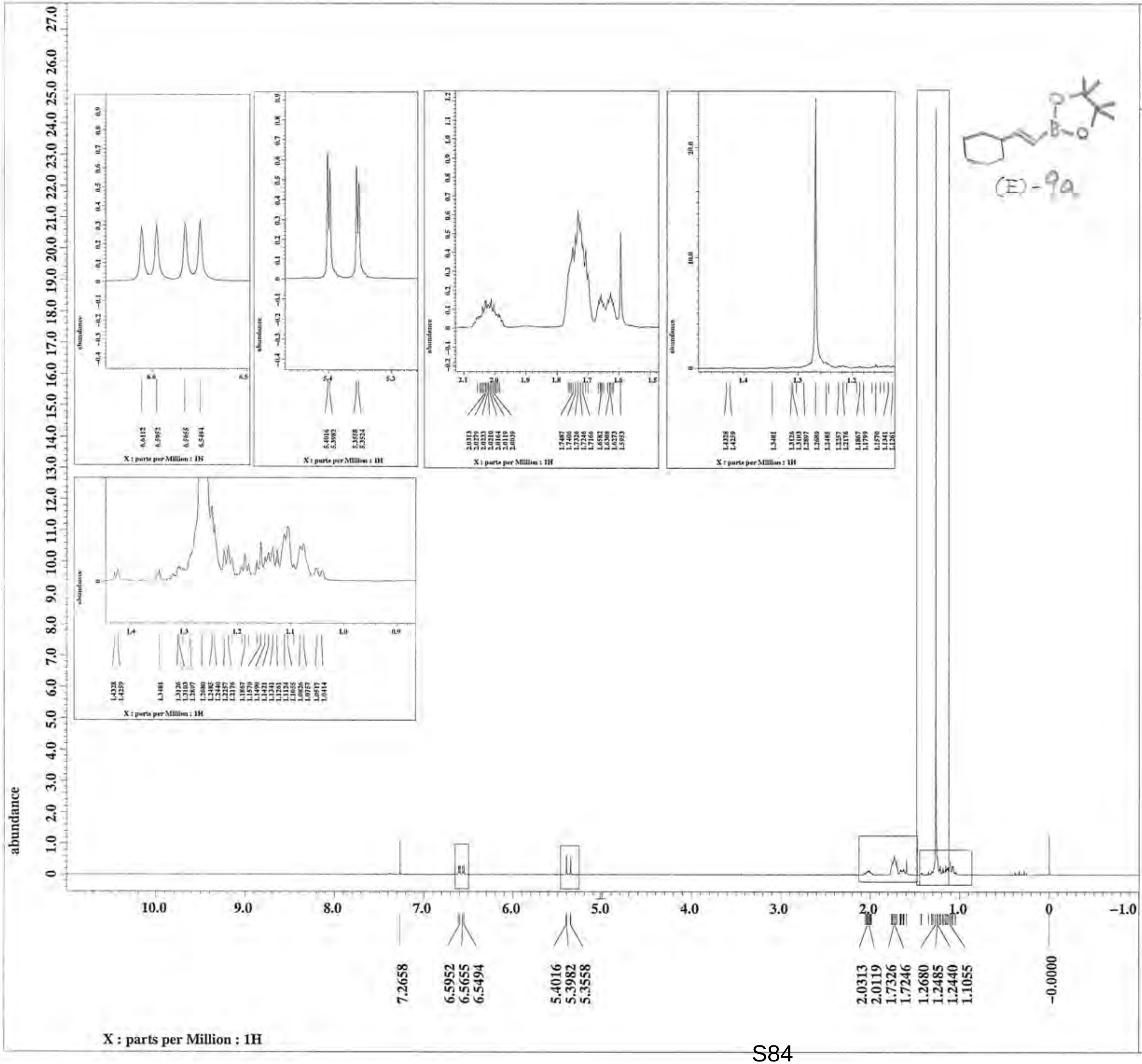

---- PROCESSING PARAMETERS ----
dc_balance : 0 : FALSE
sexp : 0.2[Hz] : 0.0[s]
trapezoid3 : 0[%] : 80[%] : 100[%]
zerofill : 1
fft : 1 : TRUE : TRUE
machinephase
ppm

Derived from: EIYA403-1.jdf

Filename	= EIYA403-4.jdf
Author	= element
Experiment	= single_pulse.ex2
Sample_id	= S#679644
Solvent	= CHLOROFORM-D
Creation_time	= 12-NOV-2013 18:14:26
Revision_time	= 12-NOV-2013 19:02:13
Current_time	= 12-NOV-2013 19:02:16
Comment	= single_pulse
Data_format	= 1D COMPLEX
Dim_size	= 13107
Dim_title	= 1H
Dim_units	= [ppm]
Dimensions	= X
Site	= ECX 400P
Spectrometer	= DELTA2_NMR
Field_strength	= 9.2982153[T] (400[MHz
X_acq_duration	= 2.20725248[s]
X_domain	= 1H
X_freq	= 395.88430144[MHz]
X_offset	= 5[ppm]
X_points	= 16384
X_prescans	= 1
X_resolution	= 0.45305193[Hz]
X_sweep	= 7.42280285[kHz]
Irr_domain	= 1H
Irr_freq	= 395.88430144[MHz]
Irr_offset	= 5[ppm]
Tri_domain	= 1H
Tri_freq	= 395.88430144[MHz]
Tri_offset	= 5[ppm]
Clipped	= FALSE
Mod_return	= 1
Scans	= 8
Total_scans	= 8
X_90_width	= 11.8[us]
X_acq_time	= 2.20725248[s]
X_angle	= 45[deg]
X_atn	= 1[dB]
X_pulse	= 5.9[us]
Irr_mode	= Off
Tri_mode	= Off
Dante_presat	= FALSE
Initial_wait	= 1[s]
Recvr_gain	= 34
Relaxation_delay	= 3[s]
Repetition_time	= 5.20725248[s]
Temp_get	= 402.4[dC]

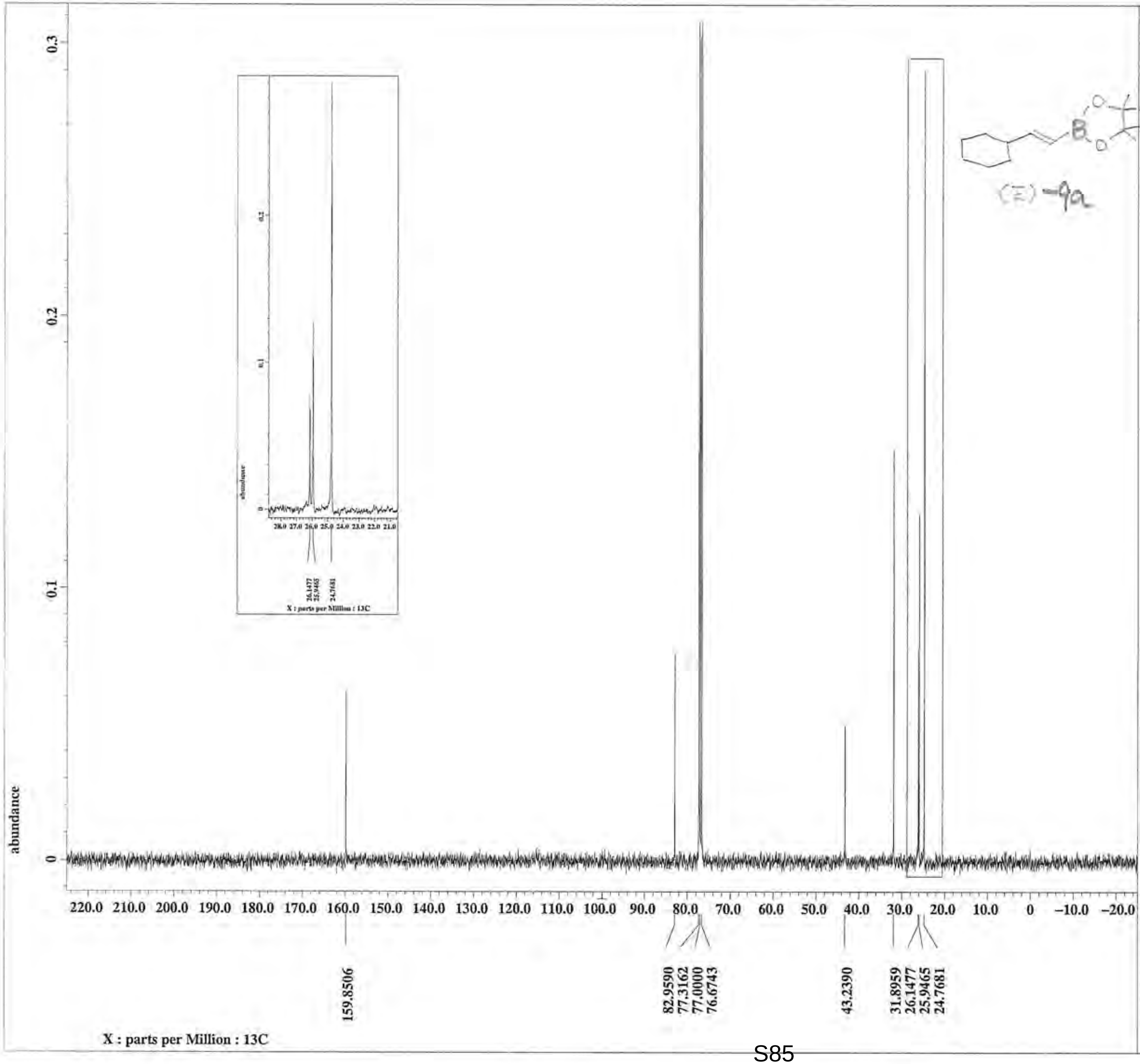


```
---- PROCESSING PARAMETERS ----
dc_balance : 0 : FALSE
sexp : 2.0[Hz] : 0.0[s]
trapezoid3 : 0[%] : 80[%] : 100[%]
zerofill : 1
fft : 1 : TRUE : TRUE
machinephase
ppm

Derived from: EIYA403 C_copy-4.jdf

Filename           = EIYA403 C_copy-7.jdf
Author             = element
Experiment         = single_pulse_dec
Sample_id          = S#680686
Solvent            = CHLOROFORM-D
Creation_time      = 12-NOV-2013 18:31:21
Revision_time      = 12-NOV-2013 19:11:42
Current_time       = 12-NOV-2013 19:12:38

Comment            = single pulse decouple
Data_format        = 1D COMPLEX
Dim_size           = 26214
Dim_title          = 13C
Dim_units          = [ppm]
Dimensions         = X
Site               = ECX 400P
Spectrometer       = DELTA2_NMR

Field_strength     = 9.2982153[T] (400[MHz
X_acq_duration     = 1.048576[s]
X_domain           = 13C
X_freq             = 99.54517646[MHz]
X_offset           = 100[ppm]
X_points           = 32768
X_prescans         = 4
X_resolution       = 0.95367432[Hz]
X_sweep            = 31.25[kHz]
Irr_domain         = 1H
Irr_freq           = 395.88430144[MHz]
Irr_offset         = 5[ppm]
Clipped            = FALSE
Incomplete_copy    = TRUE
Mod_return         = 1
Scans              = 302
Total_scans        = 302

X_90_width         = 9.3[us]
X_acq_time         = 1.048576[s]
X_angle            = 30[deg]
X_atn              = 3.4[dB]
X_pulse            = 3.1[us]
Irr_atn_dec        = 19.515[dB]
Irr_atn_noe        = 19.515[dB]
Irr_noise          = WALTZ
Decoupling         = TRUE
Initial_wait       = 1[s]
Noe                = TRUE
Noe_time           = 2[s]
Recvr_gain         = 48
Relaxation_delay   = 2[s]
Repetition_time    = 3.048576[s]
Temp_get           = 402.3[dC]
```

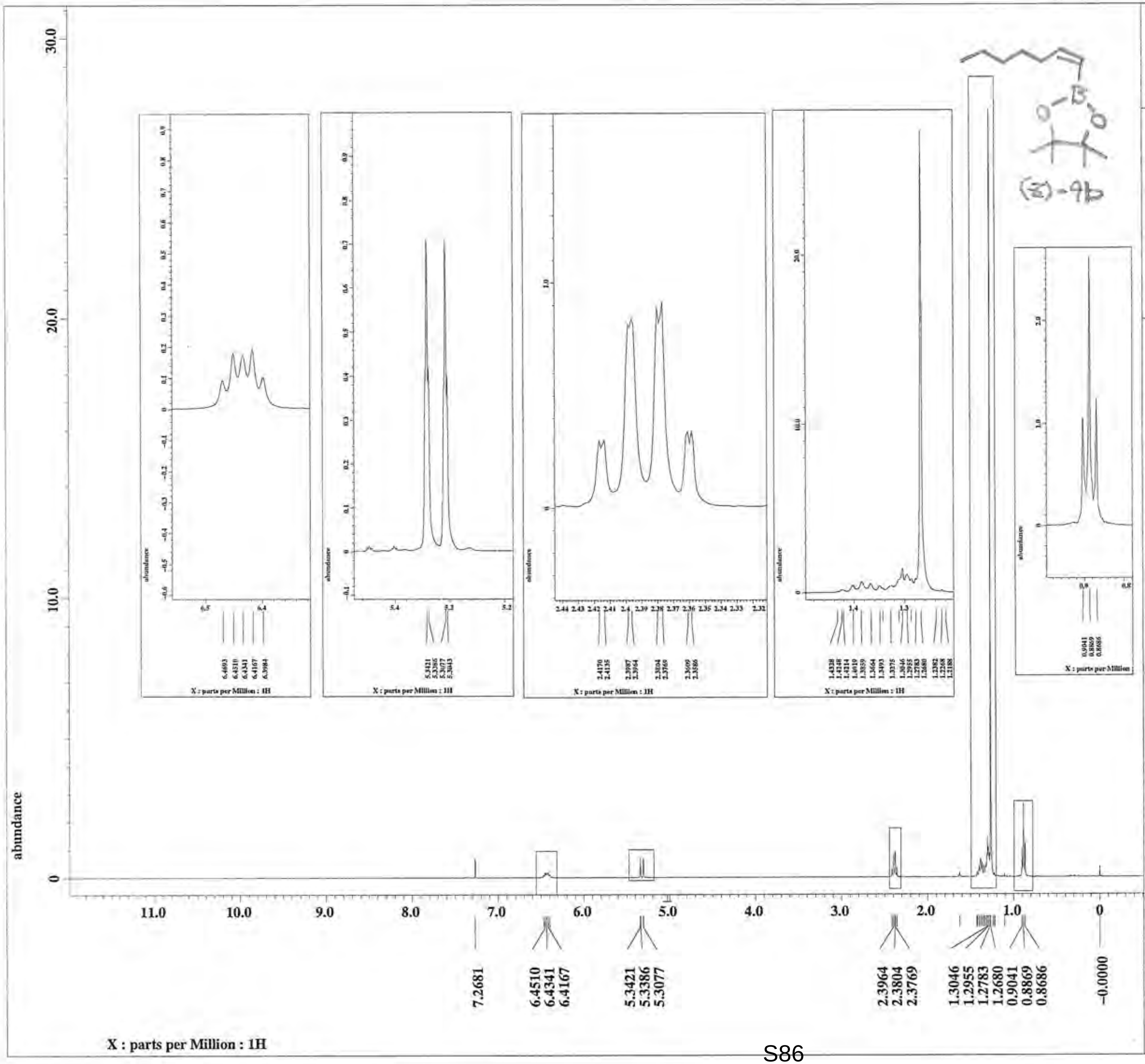

---- PROCESSING PARAMETERS ----
dc_balance : 0 : FALSE
sexp : 0.2[Hz] : 0.0[s]
trapezoid3 : 0[%] : 80[%] : 100[%]
zerofill : 1
fft : 1 : TRUE : TRUE
machinephase
ppm

Derived from: EIYA410-1.jdf

Filename	= EIYA410-5.jdf
Author	= element
Experiment	= single_pulse.ex2
Sample_id	= S#755561
Solvent	= CHLOROFORM-D
Creation_time	= 10-DEC-2013 20:21:34
Revision_time	= 10-DEC-2013 21:09:16
Current_time	= 10-DEC-2013 21:09:23
Comment	= single_pulse
Data_format	= 1D COMPLEX
Dim_size	= 13107
Dim_title	= 1H
Dim_units	= [ppm]
Dimensions	= X
Site	= ECX 400P
Spectrometer	= DELTA2_NMR
Field_strength	= 9.2982153[T] (400[MHz
X_acq_duration	= 2.20725248[s]
X_domain	= 1H
X_freq	= 395.88430144[MHz]
X_offset	= 5[ppm]
X_points	= 16384
X_prescans	= 1
X_resolution	= 0.45305193[Hz]
X_sweep	= 7.42280285[kHz]
Irr_domain	= 1H
Irr_freq	= 395.88430144[MHz]
Irr_offset	= 5[ppm]
Tri_domain	= 1H
Tri_freq	= 395.88430144[MHz]
Tri_offset	= 5[ppm]
Clipped	= FALSE
Mod_return	= 1
Scans	= 8
Total_scans	= 8
X_90_width	= 13.2[us]
X_acq_time	= 2.20725248[s]
X_angle	= 45[deg]
X_atn	= 1[dB]
X_pulse	= 6.6[us]
Irr_mode	= Off
Tri_mode	= Off
Dante_presat	= FALSE
Initial_wait	= 1[s]
Recvr_gain	= 30
Relaxation_delay	= 5[s]
Repetition_time	= 7.20725248[s]
Temp_get	= 22.5[dC]

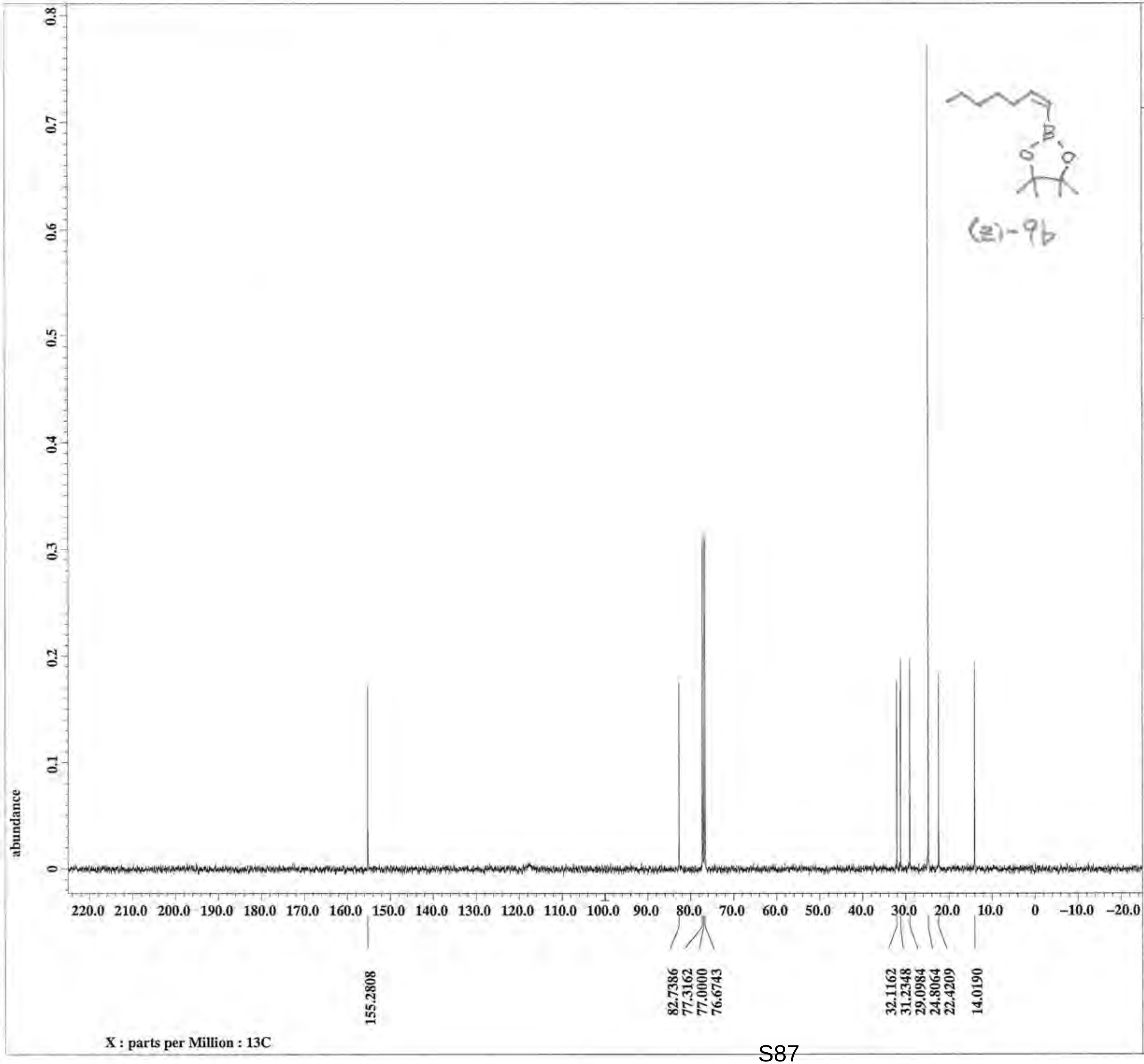

JEOL

---- PROCESSING PARAMETERS ----
dc_balance : 0 : FALSE
sexp : 2.0[Hz] : 0.0[s]
trapezoid3 : 0[%] : 80[%] : 100[%]
zerofill : 1
fft : 1 : TRUE : TRUE
machinephase
ppm

Derived from: EIYA410-C-1.jdf

Filename	= EIYA410-C-3.jdf
Author	= element
Experiment	= single_pulse_dec
Sample_id	= S#756772
Solvent	= CHLOROFORM-D
Creation_time	= 10-DEC-2013 20:31:30
Revision_time	= 10-DEC-2013 21:11:41
Current_time	= 10-DEC-2013 21:13:33
Comment	= single pulse decouple
Data_format	= 1D COMPLEX
Dim_size	= 26214
Dim_title	= 13C
Dim_units	= [ppm]
Dimensions	= X
Site	= ECX 400P
Spectrometer	= DELTA2_NMR
Field_strength	= 9.2982153[T] (400[MHz
X_acq_duration	= 1.048576[s]
X_domain	= 13C
X_freq	= 99.54517646[MHz]
X_offset	= 100[ppm]
X_points	= 32768
X_prescans	= 4
X_resolution	= 0.95367432[Hz]
X_sweep	= 31.25[kHz]
Irr_domain	= 1H
Irr_freq	= 395.88430144[MHz]
Irr_offset	= 5[ppm]
Clipped	= FALSE
Mod_return	= 1
Scans	= 164
Total_scans	= 164
X_90_width	= 9.3[us]
X_acq_time	= 1.048576[s]
X_angle	= 30[deg]
X_atn	= 3.4[dB]
X_pulse	= 3.1[us]
Irr_atn_dec	= 19.554[dB]
Irr_atn_noe	= 19.554[dB]
Irr_noise	= WALTZ
Decoupling	= TRUE
Initial_wait	= 1[s]
Noe	= TRUE
Noe_time	= 2[s]
Recvr_gain	= 48
Relaxation_delay	= 2[s]
Repetition_time	= 3.048576[s]
Temp_get	= 23.1[dC]

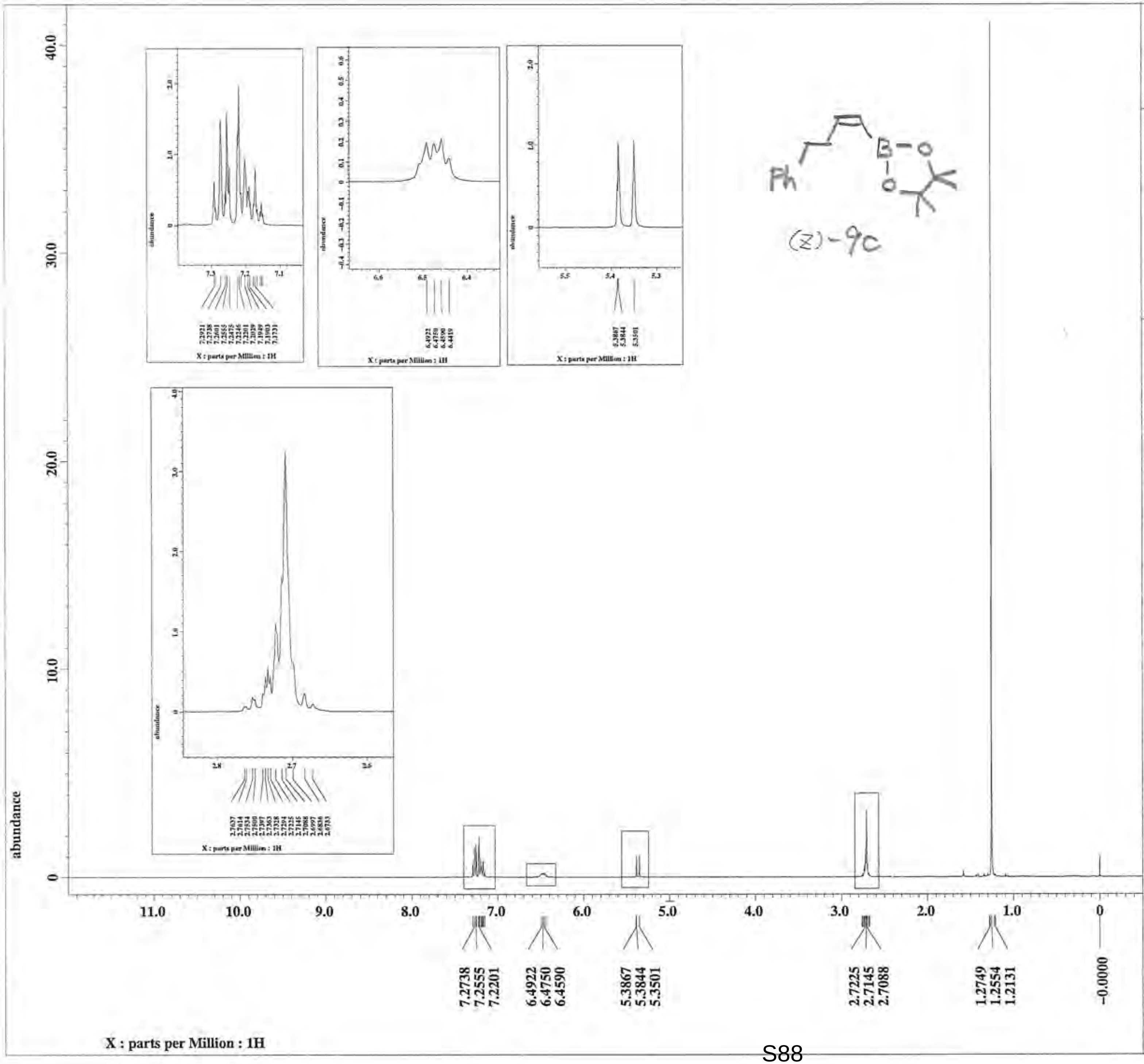

---- PROCESSING PARAMETERS ----
dc_balance : 0 : FALSE
sexp : 0.2[Hz] : 0.0[s]
trapezoid3 : 0[%] : 80[%] : 100[%]
zerofill : 1
fft : 1 : TRUE : TRUE
machinephase
ppm

Derived from: EIYA411-1.jdf

Filename	= EIYA411-6.jdf
Author	= element
Experiment	= single_pulse.ex2
Sample_id	= S#776397
Solvent	= CHLOROFORM-D
Creation_time	= 10-DEC-2013 20:56:34
Revision_time	= 10-DEC-2013 21:42:18
Current_time	= 10-DEC-2013 21:42:34
Comment	= single_pulse
Data_format	= 1D COMPLEX
Dim_size	= 13107
Dim_title	= 1H
Dim_units	= [ppm]
Dimensions	= X
Site	= ECX 400P
Spectrometer	= DELTA2_NMR
Field_strength	= 9.2982153[T] (400[MHz
X_acq_duration	= 2.20725248[s]
X_domain	= 1H
X_freq	= 395.88430144[MHz]
X_offset	= 5[ppm]
X_points	= 16384
X_prescans	= 1
X_resolution	= 0.45305193[Hz]
X_sweep	= 7.42280285[kHz]
Irr_domain	= 1H
Irr_freq	= 395.88430144[MHz]
Irr_offset	= 5[ppm]
Tri_domain	= 1H
Tri_freq	= 395.88430144[MHz]
Tri_offset	= 5[ppm]
Clipped	= FALSE
Mod_return	= 1
Scans	= 8
Total_scans	= 8
X_90_width	= 13.2[us]
X_acq_time	= 2.20725248[s]
X_angle	= 45[deg]
X_atn	= 1[dB]
X_pulse	= 6.6[us]
Irr_mode	= Off
Tri_mode	= Off
Dante_presat	= FALSE
Initial_wait	= 1[s]
Recvr_gain	= 32
Relaxation_delay	= 5[s]
Repetition_time	= 7.20725248[s]
Temp_get	= 22.4[dC]

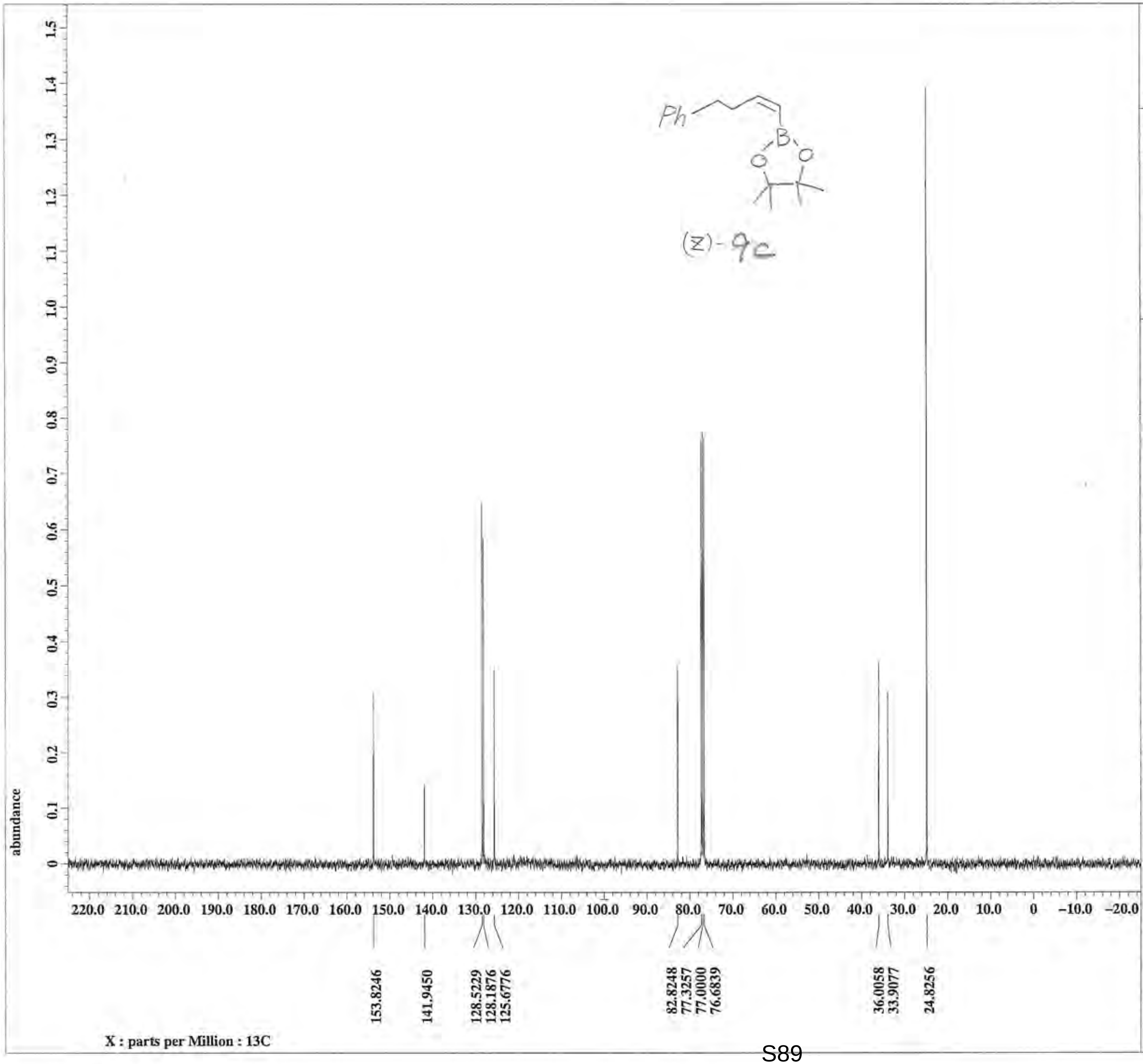


```
---- PROCESSING PARAMETERS ----
dc_balance : 0 : FALSE
sexp : 2.0[Hz] : 0.0[s]
trapezoid3 : 0[%] : 80[%] : 100[%]
zerofill : 1
fft : 1 : TRUE : TRUE
machinephase
ppm

Derived from: EIYA411-C_copy-2.jdf

Filename           = EIYA411-C_copy-4.jdf
Author             = element
Experiment         = single_pulse_dec
Sample_id          = S#777789
Solvent            = CHLOROFORM-D
Creation_time      = 10-DEC-2013 21:05:57
Revision_time      = 10-DEC-2013 21:45:53
Current_time       = 10-DEC-2013 21:46:26

Comment            = single pulse decouple
Data_format        = 1D COMPLEX
Dim_size           = 26214
Dim_title          = 13C
Dim_units          = [ppm]
Dimensions         = X
Site               = ECX 400P
Spectrometer       = DELTA2_NMR

Field_strength     = 9.2982153[T] (400[MHz
X_acq_duration     = 1.048576[s]
X_domain           = 13C
X_freq             = 99.54517646[MHz]
X_offset           = 100[ppm]
X_points           = 32768
X_prescans         = 4
X_resolution       = 0.95367432[Hz]
X_sweep            = 31.25[kHz]
Irr_domain         = 1H
Irr_freq           = 395.88430144[MHz]
Irr_offset         = 5[ppm]
Clipped            = FALSE
Incomplete_copy    = TRUE
Mod_return         = 1
Scans              = 157
Total_scans        = 157

X_90_width         = 9.3[us]
X_acq_time         = 1.048576[s]
X_angle            = 30[deg]
X_atn              = 3.4[dB]
X_pulse            = 3.1[us]
Irr_atn_dec        = 19.554[dB]
Irr_atn_noe        = 19.554[dB]
Irr_noise          = WALTZ
Decoupling         = TRUE
Initial_wait       = 1[s]
Noe                = TRUE
Noe_time           = 2[s]
Recvr_gain         = 56
Relaxation_delay   = 2[s]
Repetition_time    = 3.048576[s]
Temp_get           = 22.5[dC]
```

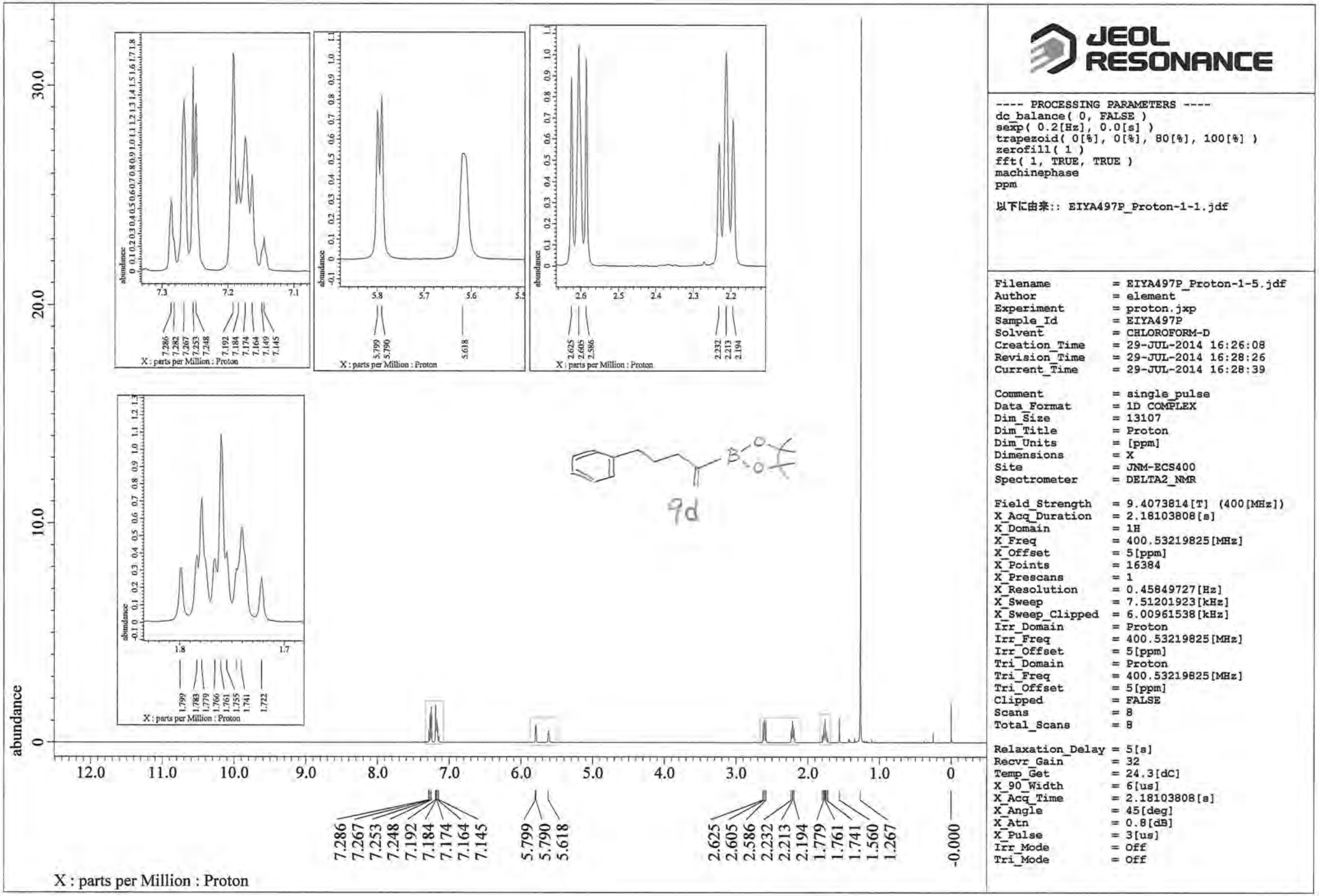
JEOL RESONANCE
---- PROCESSING PARAMETERS ----
dc_balance(0, FALSE)
sexp(0.2[Hz], 0.0[s])
trapezoid(0[%], 0[%], 80[%], 100[%])
zerofill(1)
fft(1, TRUE, TRUE)
machinephase
ppm
以下に由来:: EIYA497P_Proton-1-1.jdf
Filename = EIYA497P_Proton-1-5.jdf
Author = element
Experiment = proton.jxp
Sample_Id = EIYA497P
Solvent = CHLOROFORM-D
Creation_Time = 29-JUL-2014 16:26:08
Revision_Time = 29-JUL-2014 16:28:26
Current_Time = 29-JUL-2014 16:28:39
Comment = single_pulse
Data_Format = 1D COMPLEX
Dim_Size = 13107
Dim_Title = Proton
Dim_Units = [ppm]
Dimensions = X
Site = JNM-ECS400
Spectrometer = DELTA2_NMR
Field_Strength = 9.4073814[T] (400[MHz])
X_Acq_Duration = 2.18103808[s]
X_Domain = 1H
X_Freq = 400.53219825[MHz]
X_Offset = 5[ppm]
X_Points = 16384
X_Prescans = 1
X_Resolution = 0.45849727[Hz]
X_Sweep = 7.51201923[kHz]
X_Sweep_Clipped = 6.00961538[kHz]
Irr_Domain = Proton
Irr_Freq = 400.53219825[MHz]
Irr_Offset = 5[ppm]
Tri_Domain = Proton
Tri_Freq = 400.53219825[MHz]
Tri_Offset = 5[ppm]
Clipped = FALSE
Scans = 8
Total_Scans = 8
Relaxation_Delay = 5[s]
Recvr_Gain = 32
Temp_Get = 24.3[dC]
X_90_Width = 6[us]
X_Acq_Time = 2.18103808[s]
X_Angle = 45[deg]
X_Atn = 0.8[dB]
X_Pulse = 3[us]
Irr_Mode = Off
Tri_Mode = Off
9d
abundance
X : parts per Million : Proton
7.286 7.282 7.267 7.253 7.248 7.192 7.184 7.174 7.164 7.149 7.145
5.799 5.790 5.618
2.625 2.605 2.586 2.232 2.213 2.194
1.799 1.783 1.779 1.766 1.761 1.755 1.741 1.722
7.286 7.267 7.253 7.248 7.192 7.184 7.174 7.164 7.145 5.799 5.790 5.618 2.625 2.605 2.586 2.232 2.213 2.194 1.779 1.761 1.741 1.560 1.267 -0.000

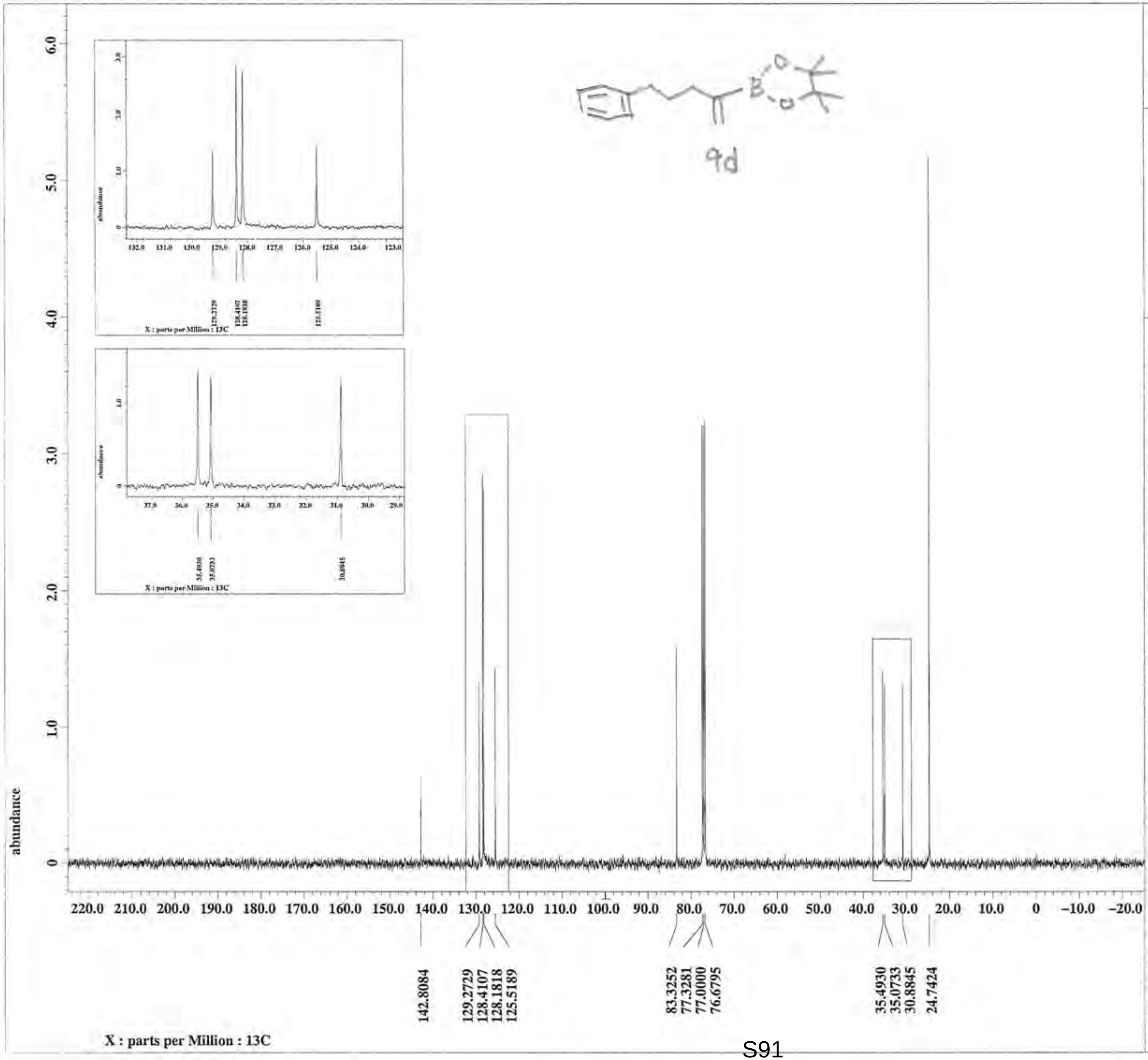

---- PROCESSING PARAMETERS ----
dc_balance : 0 : FALSE
sexp : 2.0[Hz] : 0.0[s]
trapezoid3 : 0[%] : 80[%] : 100[%]
zerofill : 1
fft : 1 : TRUE : TRUE
machinephase
ppm

Derived from: EIYA497P-C_copy-4.jdf

Filename	= EIYA497P-C_copy-6.jdf
Author	= element
Experiment	= single_pulse_dec
Sample_id	= S#618056
Solvent	= CHLOROFORM-D
Creation_time	= 29-JUL-2014 17:12:29
Revision_time	= 29-JUL-2014 17:20:17
Current_time	= 29-JUL-2014 17:21:06
Comment	= single pulse decouple
Data_format	= 1D COMPLEX
Dim_size	= 32768
Dim_title	= 13C
Dim_units	= [ppm]
Dimensions	= X
Site	= ECS 400
Spectrometer	= JNM-ECS400
Field_strength	= 9.20197068[T] (390[MH
X_acq_duration	= 1.3303808[s]
X_domain	= 13C
X_freq	= 98.51479726[MHz]
X_offset	= 100[ppm]
X_points	= 32768
X_prescans	= 4
X_resolution	= 0.75166449[Hz]
X_sweep	= 24.63054187[kHz]
Irr_domain	= 1H
Irr_freq	= 391.78655441[MHz]
Irr_offset	= 5[ppm]
Clipped	= FALSE
Incomplete_copy	= TRUE
Mod_return	= 1
Scans	= 151
Total_scans	= 151
X_90_width	= 8.8[us]
X_acq_time	= 1.3303808[s]
X_angle	= 30[deg]
X_atn	= 4.9[dB]
X_pulse	= 2.93333333[us]
Irr_atn_dec	= 22.52628[dB]
Irr_atn_noe	= 22.52628[dB]
Irr_noise	= WALTZ
Decoupling	= TRUE
Initial_wait	= 1[s]
Noe	= TRUE
Noe_time	= 2[s]
Recvr_gain	= 72
Relaxation_delay	= 2[s]
Repetition_time	= 3.3303808[s]
Temp_get	= 21.9[dC]

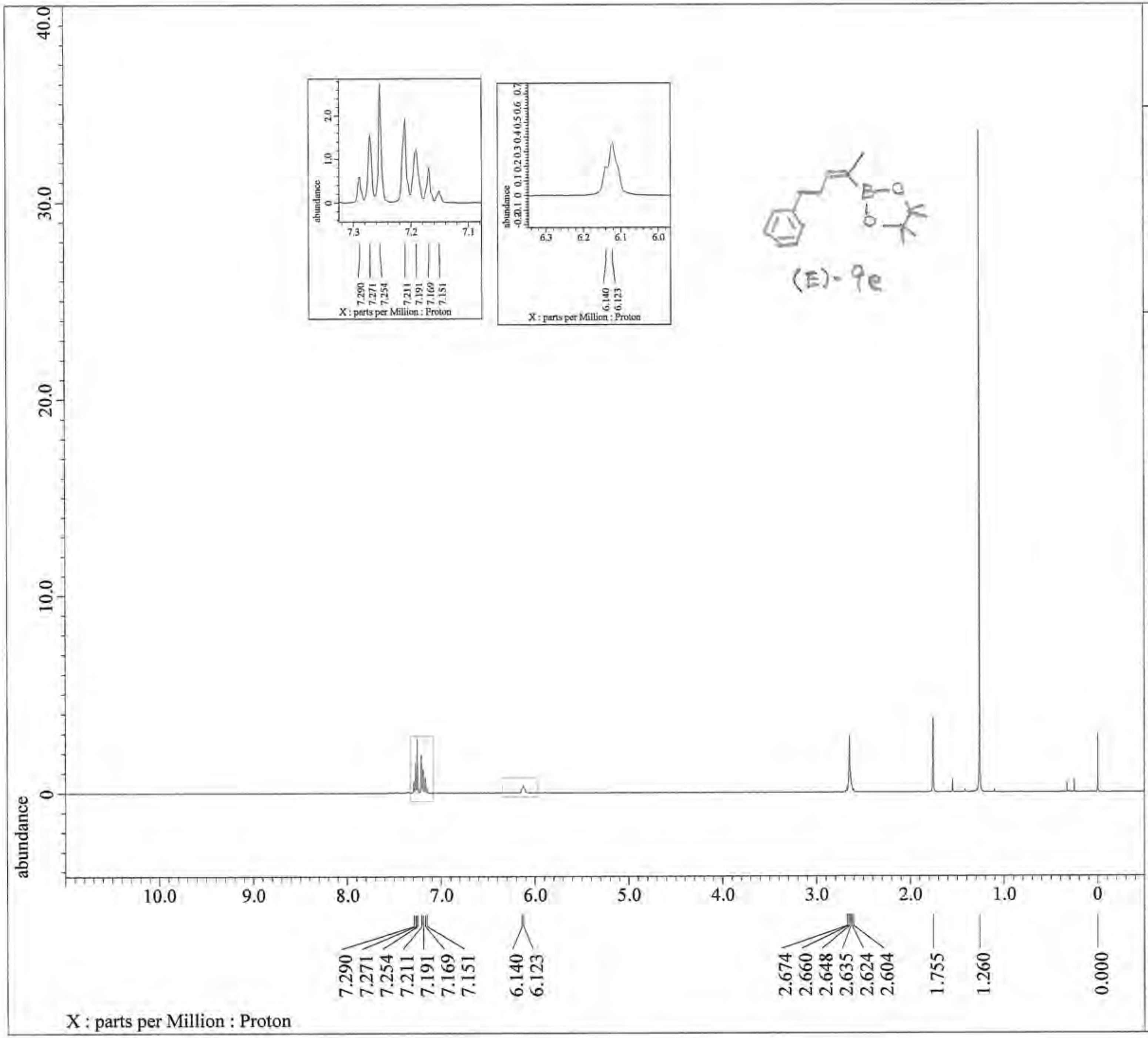

JEOL RESONANCE

```
---- PROCESSING PARAMETERS ----
dc_balance( 0, FALSE )
sexp( 0.2[Hz], 0.0[s] )
trapezoid( 0[%], 0[%], 80[%], 100[%] )
zerofill( 1 )
fft( 1, TRUE, TRUE )
machinephase
ppm

以下に由来:: EIYA437_Proton-1-1.jdf

Filename          = EIYA437_Proton-1-4.jdf
Author            = element
Experiment        = proton.jxp
Sample_Id         = EIYA437
Solvent           = CHLOROFORM-D
Creation_Time     = 19-JUN-2014 12:36:56
Revision_Time     = 19-JUN-2014 12:53:37
Current_Time      = 19-JUN-2014 12:53:43

Comment           = single_pulse
Data_Format       = 1D COMPLEX
Dim_Size          = 13107
Dim_Title         = Proton
Dim_Units         = [ppm]
Dimensions        = X
Site              = JNM-ECS400
Spectrometer      = DELTA2_NMR

Field_Strength    = 9.4073814[T] (400[MHz])
X_Acq_Duration    = 2.18103808[s]
X_Domain          = 1H
X_Freq            = 400.53219825[MHz]
X_Offset          = 5[ppm]
X_Points          = 16384
X_Prescans        = 1
X_Resolution      = 0.45849727[Hz]
X_Sweep           = 7.51201923[kHz]
X_Sweep_Clipped   = 6.00961538[kHz]
Irr_Domain        = Proton
Irr_Freq          = 400.53219825[MHz]
Irr_Offset        = 5[ppm]
Tri_Domain        = Proton
Tri_Freq          = 400.53219825[MHz]
Tri_Offset        = 5[ppm]
Clipped           = FALSE
Scans             = 8
Total_Scans       = 8

Relaxation_Delay  = 5[s]
Recvr_Gain        = 36
Temp_Get          = 22.3[dC]
X_90_Width        = 6[us]
X_Acq_Time        = 2.18103808[s]
X_Angle           = 45[deg]
X_Atn             = 0.8[dB]
X_Pulse           = 3[us]
Irr_Mode          = Off
Tri_Mode          = Off
```

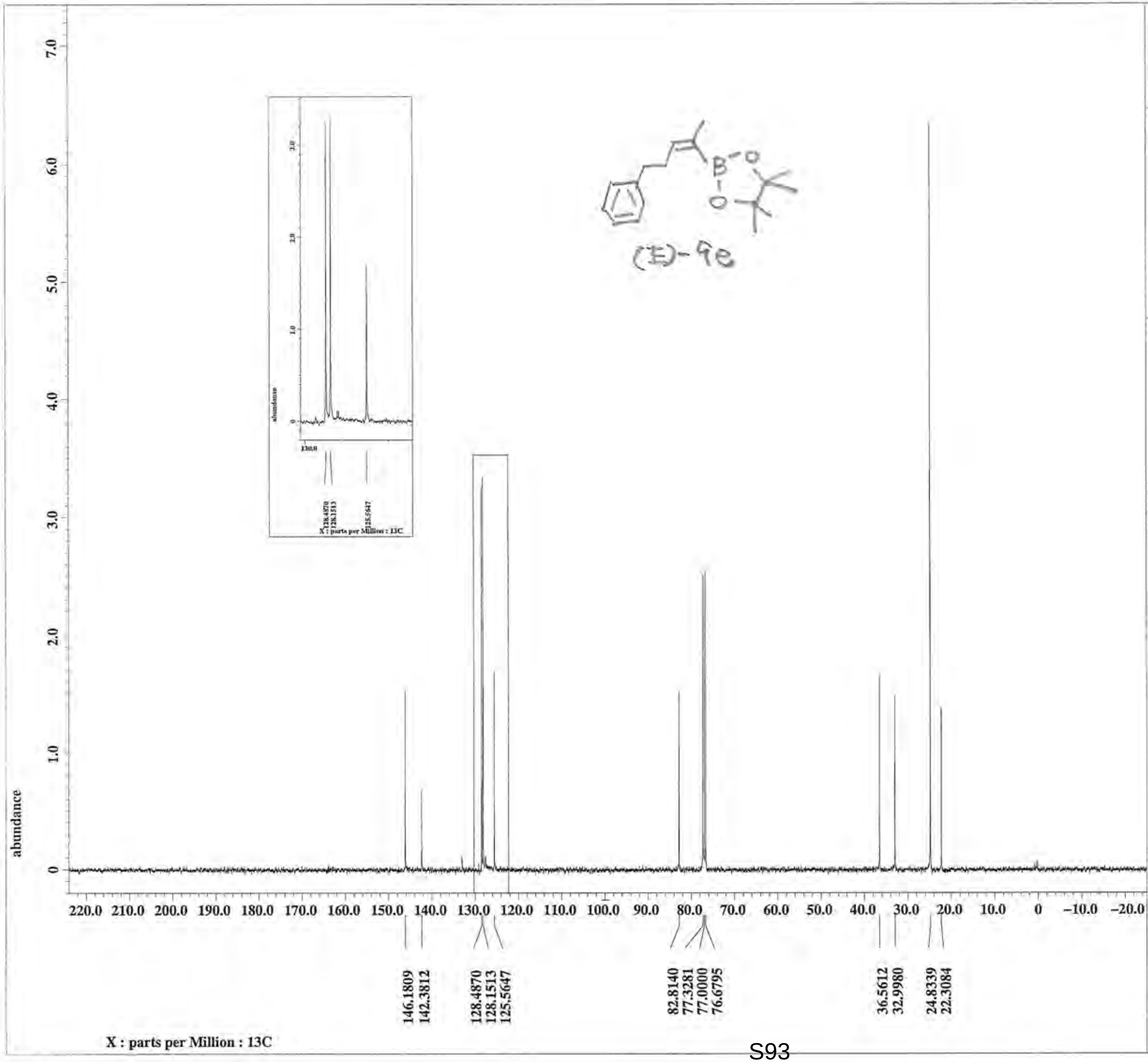

---- PROCESSING PARAMETERS ----
dc_balance : 0 : FALSE
sexp : 2.0[Hz] : 0.0[s]
trapezoid3 : 0[%] : 80[%] : 100[%]
zerofill : 1
fft : 1 : TRUE : TRUE
machinephase
ppm

Derived from: EIYA437C-2-1.jdf

Filename	= EIYA437C-2-3.jdf
Author	= element
Experiment	= single_pulse_dec
Sample_id	= S#533314
Solvent	= CHLOROFORM-D
Creation_time	= 19-JUN-2014 15:02:26
Revision_time	= 19-JUN-2014 15:04:43
Current_time	= 19-JUN-2014 15:05:21
Comment	= single pulse decouple
Data_format	= 1D COMPLEX
Dim_size	= 32768
Dim_title	= 13C
Dim_units	= [ppm]
Dimensions	= X
Site	= ECS 400
Spectrometer	= JNM-ECS400
Field_strength	= 9.20197068[T] (390[MH
X_acq_duration	= 1.3303808[s]
X_domain	= 13C
X_freq	= 98.51479726[MHz]
X_offset	= 100[ppm]
X_points	= 32768
X_prescans	= 4
X_resolution	= 0.75166449[Hz]
X_sweep	= 24.63054187[kHz]
Irr_domain	= 1H
Irr_freq	= 391.78655441[MHz]
Irr_offset	= 5[ppm]
Clipped	= FALSE
Mod_return	= 1
Scans	= 252
Total_scans	= 252
X_90_width	= 8.8[us]
X_acq_time	= 1.3303808[s]
X_angle	= 30[deg]
X_atn	= 4.9[dB]
X_pulse	= 2.93333333[us]
Irr_atn_dec	= 22.52628[dB]
Irr_atn_noe	= 22.52628[dB]
Irr_noise	= WALTZ
Decoupling	= TRUE
Initial_wait	= 1[s]
Noe	= TRUE
Noe_time	= 2[s]
Recvr_gain	= 70
Relaxation_delay	= 2[s]
Repetition_time	= 3.3303808[s]
Temp_get	= 21.6[dC]

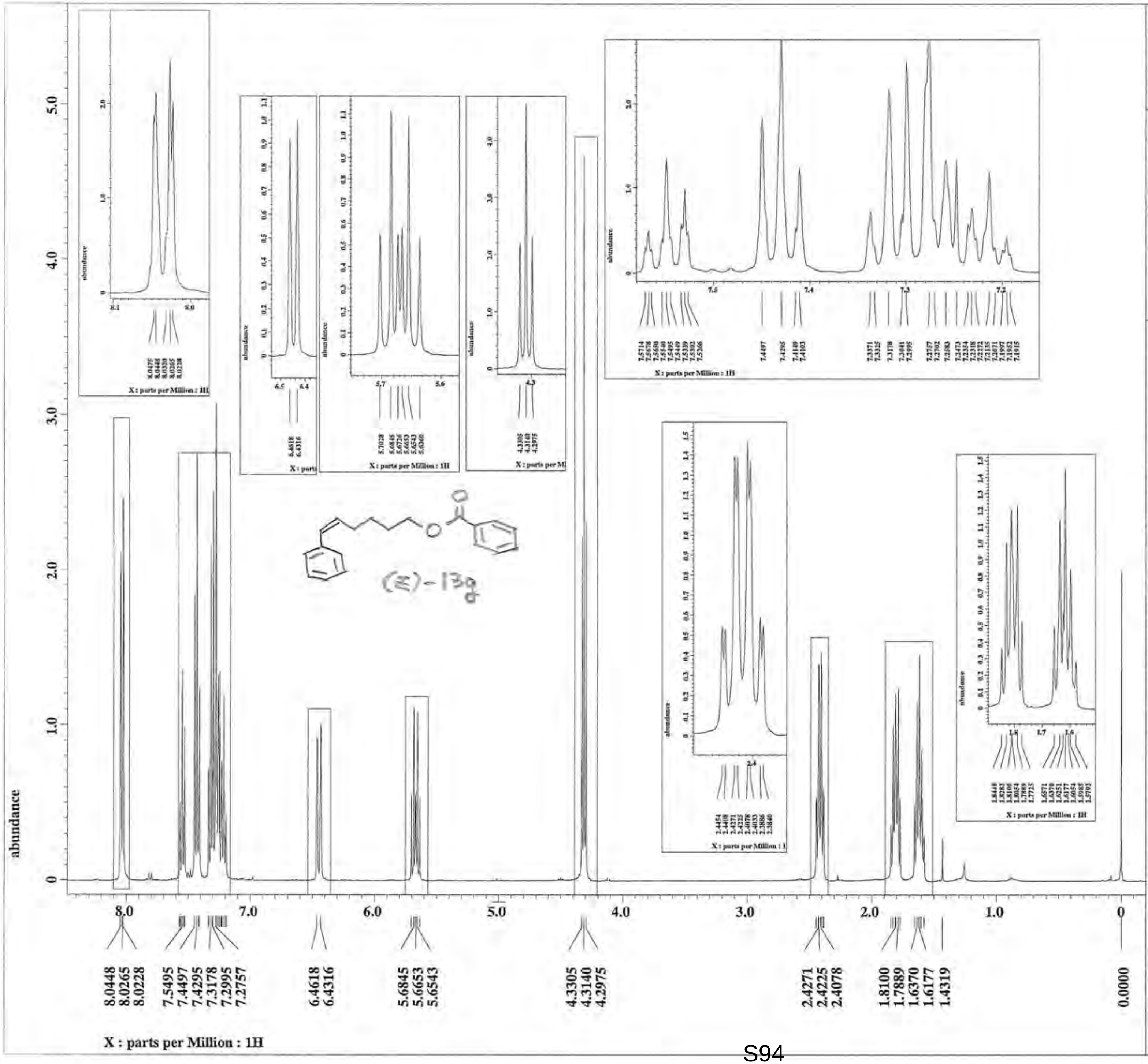

JEOL

---- PROCESSING PARAMETERS ----
dc_balance : 0 : FALSE
sexp : 0.2[Hz] : 0.0[s]
trapezoid3 : 0[%] : 80[%] : 100[%]
zerofill : 1
fft : 1 : TRUE : TRUE
machinephase
ppm

Derived from: EIYA475P-H-1.jdf

Filename	= EIYA475P-H-4.jdf
Author	= element
Experiment	= single_pulse.ex2
Sample_id	= S#557648
Solvent	= CHLOROFORM-D
Creation_time	= 4-MAY-2014 14:10:42
Revision_time	= 4-MAY-2014 15:39:59
Current_time	= 4-MAY-2014 15:40:00
Comment	= single_pulse
Data_format	= 1D COMPLEX
Dim_size	= 16384
Dim_title	= 1H
Dim_units	= [ppm]
Dimensions	= X
Site	= ECS 400
Spectrometer	= JNM-ECS400
Field_strength	= 9.20197068[T] (390[MH
X_acq_duration	= 2.78790144[s]
X_domain	= 1H
X_freq	= 391.78655441[MHz]
X_offset	= 5[ppm]
X_points	= 16384
X_prescans	= 1
X_resolution	= 0.35869274[Hz]
X_sweep	= 5.87682181[kHz]
Irr_domain	= 1H
Irr_freq	= 391.78655441[MHz]
Irr_offset	= 5[ppm]
Tri_domain	= 1H
Tri_freq	= 391.78655441[MHz]
Tri_offset	= 5[ppm]
Clipped	= FALSE
Mod_return	= 1
Scans	= 8
Total_scans	= 8
X_90_width	= 10.8[us]
X_acq_time	= 2.78790144[s]
X_angle	= 45[deg]
X_atn	= 1.9[dB]
X_pulse	= 5.4[us]
Irr_mode	= Off
Tri_mode	= Off
Dante_presat	= FALSE
Initial_wait	= 1[s]
Recvr_gain	= 38
Relaxation_delay	= 3[s]
Repetition_time	= 5.78790144[s]
Temp_get	= 20.1[dC]

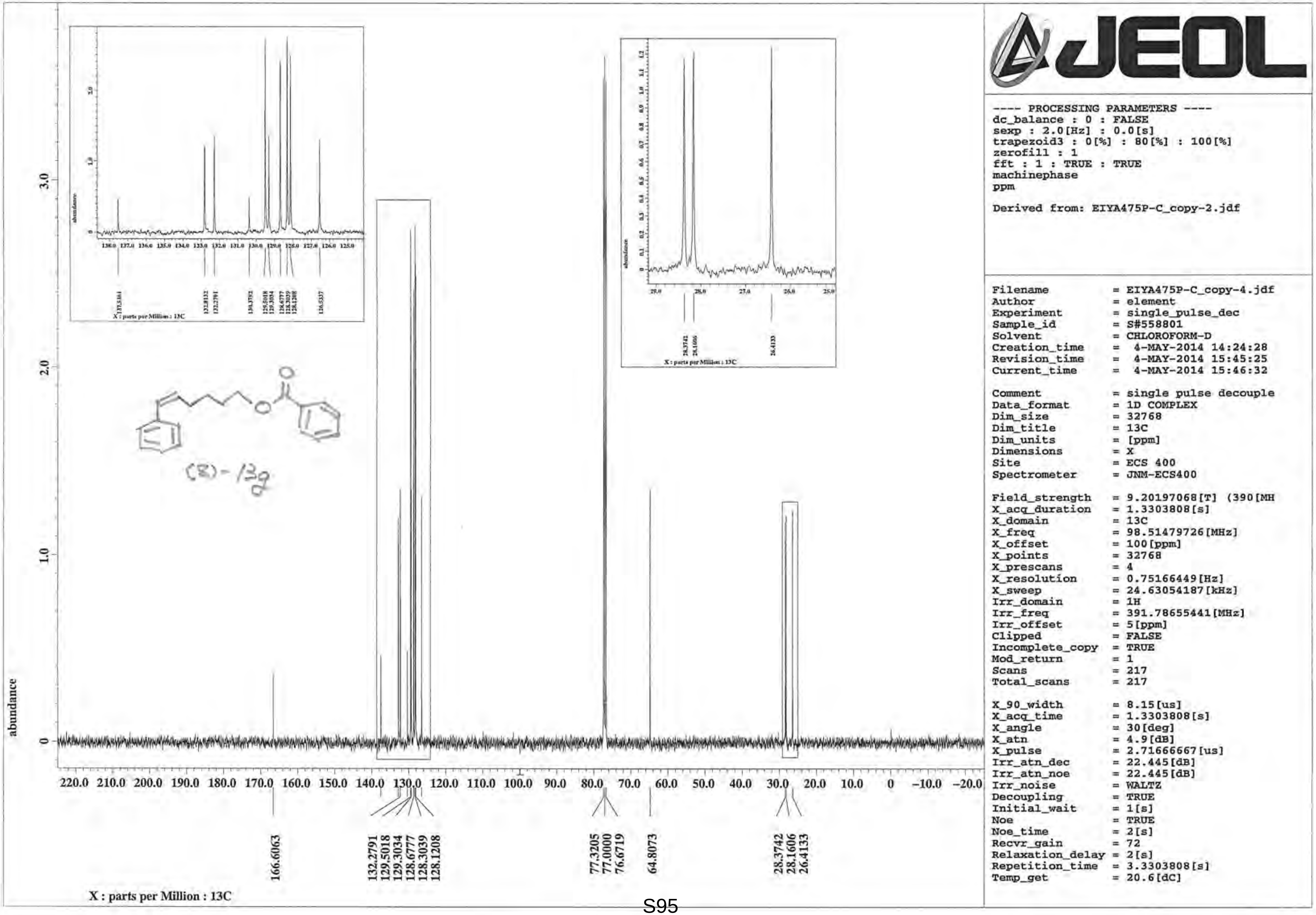
JEOL
---- PROCESSING PARAMETERS ----
dc_balance : 0 : FALSE
sexp : 2.0[Hz] : 0.0[s]
trapezoid3 : 0[%] : 80[%] : 100[%]
zerofill : 1
fft : 1 : TRUE : TRUE
machinephase
ppm
Derived from: EIYA475P-C_copy-2.jdf
Filename = EIYA475P-C_copy-4.jdf
Author = element
Experiment = single_pulse_dec
Sample_id = S#558801
Solvent = CHLOROFORM-D
Creation_time = 4-MAY-2014 14:24:28
Revision_time = 4-MAY-2014 15:45:25
Current_time = 4-MAY-2014 15:46:32
Comment = single pulse decouple
Data_format = 1D COMPLEX
Dim_size = 32768
Dim_title = 13C
Dim_units = [ppm]
Dimensions = X
Site = ECS 400
Spectrometer = JNM-ECS400
Field_strength = 9.20197068[T] (390[MH
X_acq_duration = 1.3303808[s]
X_domain = 13C
X_freq = 98.51479726[MHz]
X_offset = 100[ppm]
X_points = 32768
X_prescans = 4
X_resolution = 0.75166449[Hz]
X_sweep = 24.63054187[kHz]
Irr_domain = 1H
Irr_freq = 391.78655441[MHz]
Irr_offset = 5[ppm]
Clipped = FALSE
Incomplete_copy = TRUE
Mod_return = 1
Scans = 217
Total_scans = 217
X_90_width = 8.15[us]
X_acq_time = 1.3303808[s]
X_angle = 30[deg]
X_atn = 4.9[dB]
X_pulse = 2.71666667[us]
Irr_atn_dec = 22.445[dB]
Irr_atn_noe = 22.445[dB]
Irr_noise = WALTZ
Decoupling = TRUE
Initial_wait = 1[s]
Noe = TRUE
Noe_time = 2[s]
Recvr_gain = 72
Relaxation_delay = 2[s]
Repetition_time = 3.3303808[s]
Temp_get = 20.6[dC]
(Z)-13g
abundance
X : parts per Million : 13C
166.6063
132.2791
129.5018
129.3034
128.6777
128.3039
128.1208
77.3205
77.0000
76.6719
64.8073
28.3742
28.1606
26.4133
137.5364
132.8132
130.3792
126.5337

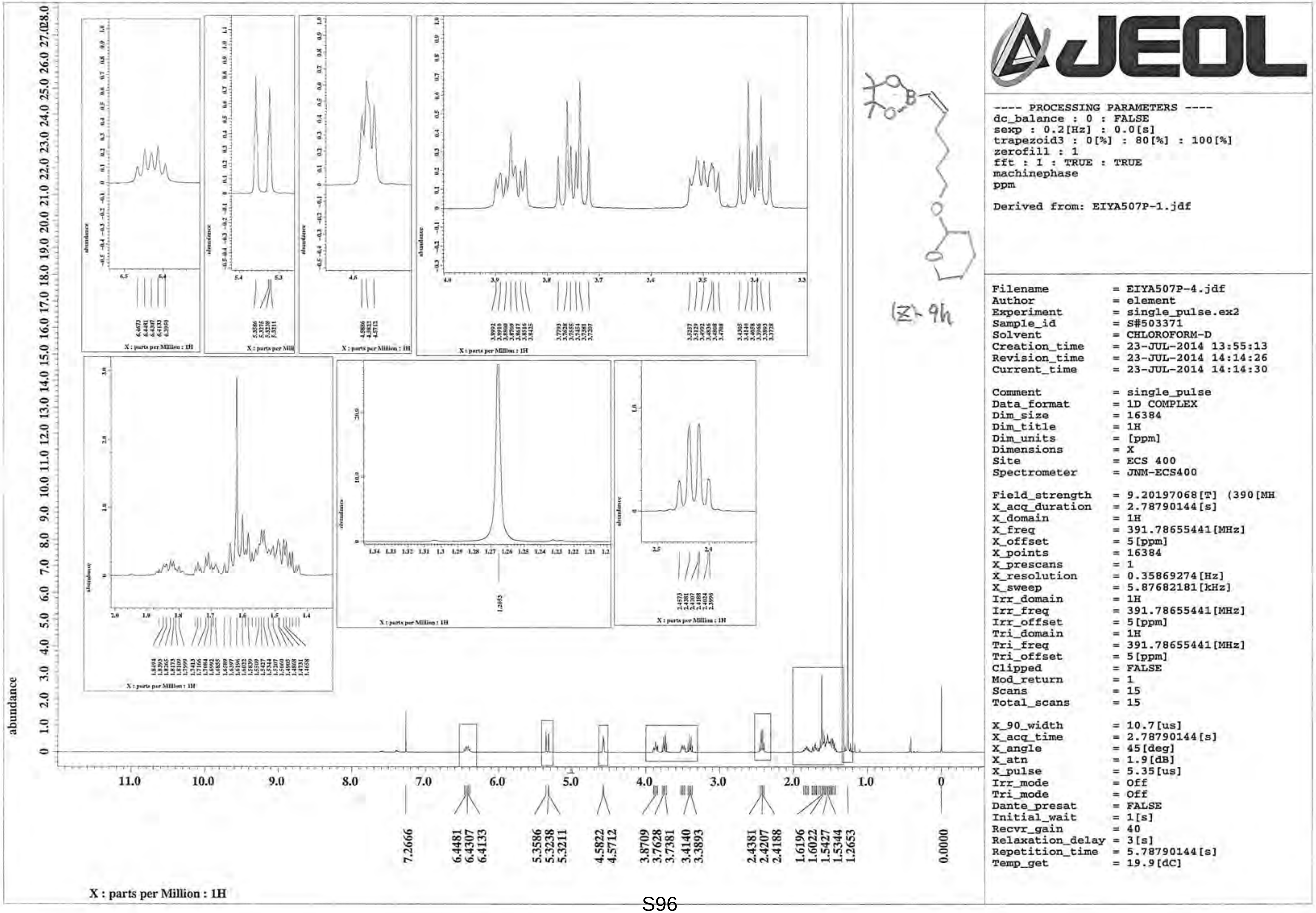
(Z)-9h
JEOL
---- PROCESSING PARAMETERS ----
dc_balance : 0 : FALSE
sexp : 0.2[Hz] : 0.0[s]
trapezoid3 : 0[%] : 80[%] : 100[%]
zerofill : 1
fft : 1 : TRUE : TRUE
machinephase
ppm
Derived from: EIYA507P-1.jdf
Filename = EIYA507P-4.jdf
Author = element
Experiment = single_pulse.ex2
Sample_id = S#503371
Solvent = CHLOROFORM-D
Creation_time = 23-JUL-2014 13:55:13
Revision_time = 23-JUL-2014 14:14:26
Current_time = 23-JUL-2014 14:14:30
Comment = single_pulse
Data_format = 1D COMPLEX
Dim_size = 16384
Dim_title = 1H
Dim_units = [ppm]
Dimensions = X
Site = ECS 400
Spectrometer = JNM-ECS400
Field_strength = 9.20197068[T] (390[MH
X_acq_duration = 2.78790144[s]
X_domain = 1H
X_freq = 391.78655441[MHz]
X_offset = 5[ppm]
X_points = 16384
X_prescans = 1
X_resolution = 0.35869274[Hz]
X_sweep = 5.87682181[kHz]
Irr_domain = 1H
Irr_freq = 391.78655441[MHz]
Irr_offset = 5[ppm]
Tri_domain = 1H
Tri_freq = 391.78655441[MHz]
Tri_offset = 5[ppm]
Clipped = FALSE
Mod_return = 1
Scans = 15
Total_scans = 15
X_90_width = 10.7[us]
X_acq_time = 2.78790144[s]
X_angle = 45[deg]
X_atn = 1.9[dB]
X_pulse = 5.35[us]
Irr_mode = Off
Tri_mode = Off
Dante_presat = FALSE
Initial_wait = 1[s]
Recvr_gain = 40
Relaxation_delay = 3[s]
Repetition_time = 5.78790144[s]
Temp_get = 19.9[dC]
7.2666
6.4481
6.4307
6.4133
5.3586
5.3238
5.3211
4.5822
4.5712
3.8709
3.7628
3.7381
3.4140
3.3893
2.4381
2.4207
2.4188
1.6196
1.6022
1.5427
1.5344
1.2653
0.0000
abundance
X : parts per Million : 1H

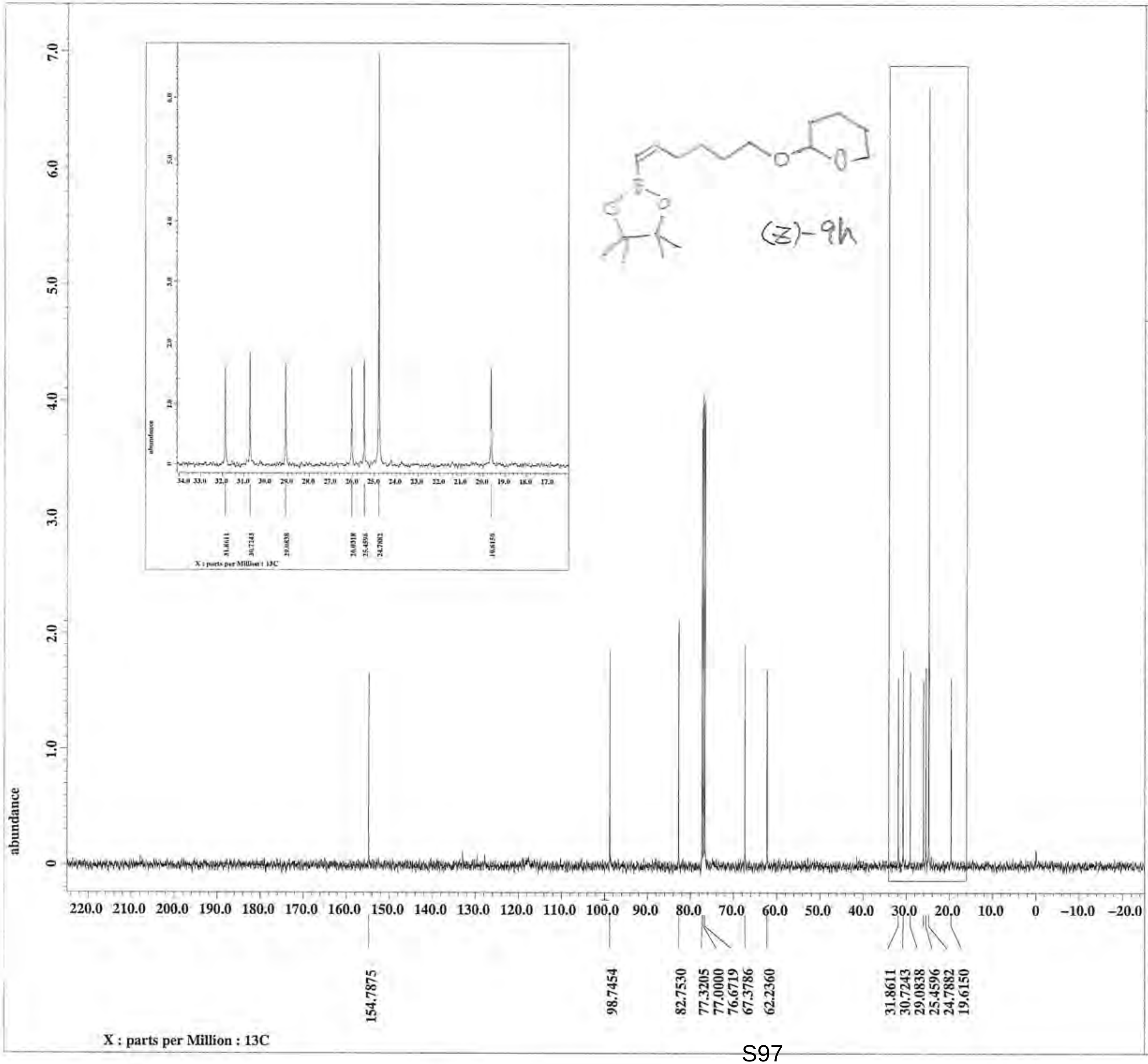

JEOL

---- PROCESSING PARAMETERS ----
dc_balance : 0 : FALSE
sexp : 2.0[Hz] : 0.0[s]
trapezoid3 : 0[%] : 80[%] : 100[%]
zerofill : 1
fft : 1 : TRUE : TRUE
machinephase
ppm

Derived from: EIYA507C_copy-2.jdf

Filename	=	EIYA507C_copy-4.jdf
Author	=	element
Experiment	=	single_pulse_dec
Sample_id	=	S#510109
Solvent	=	CHLOROFORM-D
Creation_time	=	23-JUL-2014 14:10:58
Revision_time	=	23-JUL-2014 14:18:16
Current_time	=	23-JUL-2014 14:19:14
Comment	=	single pulse decouple
Data_format	=	1D COMPLEX
Dim_size	=	32768
Dim_title	=	13C
Dim_units	=	[ppm]
Dimensions	=	X
Site	=	ECS 400
Spectrometer	=	JNM-ECS400
Field_strength	=	9.20197068[T] (390[MH
X_acq_duration	=	1.3303808[s]
X_domain	=	13C
X_freq	=	98.51479726[MHz]
X_offset	=	100[ppm]
X_points	=	32768
X_prescans	=	4
X_resolution	=	0.75166449[Hz]
X_sweep	=	24.63054187[kHz]
Irr_domain	=	1H
Irr_freq	=	391.78655441[MHz]
Irr_offset	=	5[ppm]
Clipped	=	FALSE
Incomplete_copy	=	TRUE
Mod_return	=	1
Scans	=	106
Total_scans	=	106
X_90_width	=	8.8[us]
X_acq_time	=	1.3303808[s]
X_angle	=	30[deg]
X_atn	=	4.9[dB]
X_pulse	=	2.93333333[us]
Irr_atn_dec	=	22.52628[dB]
Irr_atn_noe	=	22.52628[dB]
Irr_noise	=	WALTZ
Decoupling	=	TRUE
Initial_wait	=	1[s]
Noe	=	TRUE
Noe_time	=	2[s]
Recvr_gain	=	72
Relaxation_delay	=	2[s]
Repetition_time	=	3.3303808[s]
Temp_get	=	20[dC]

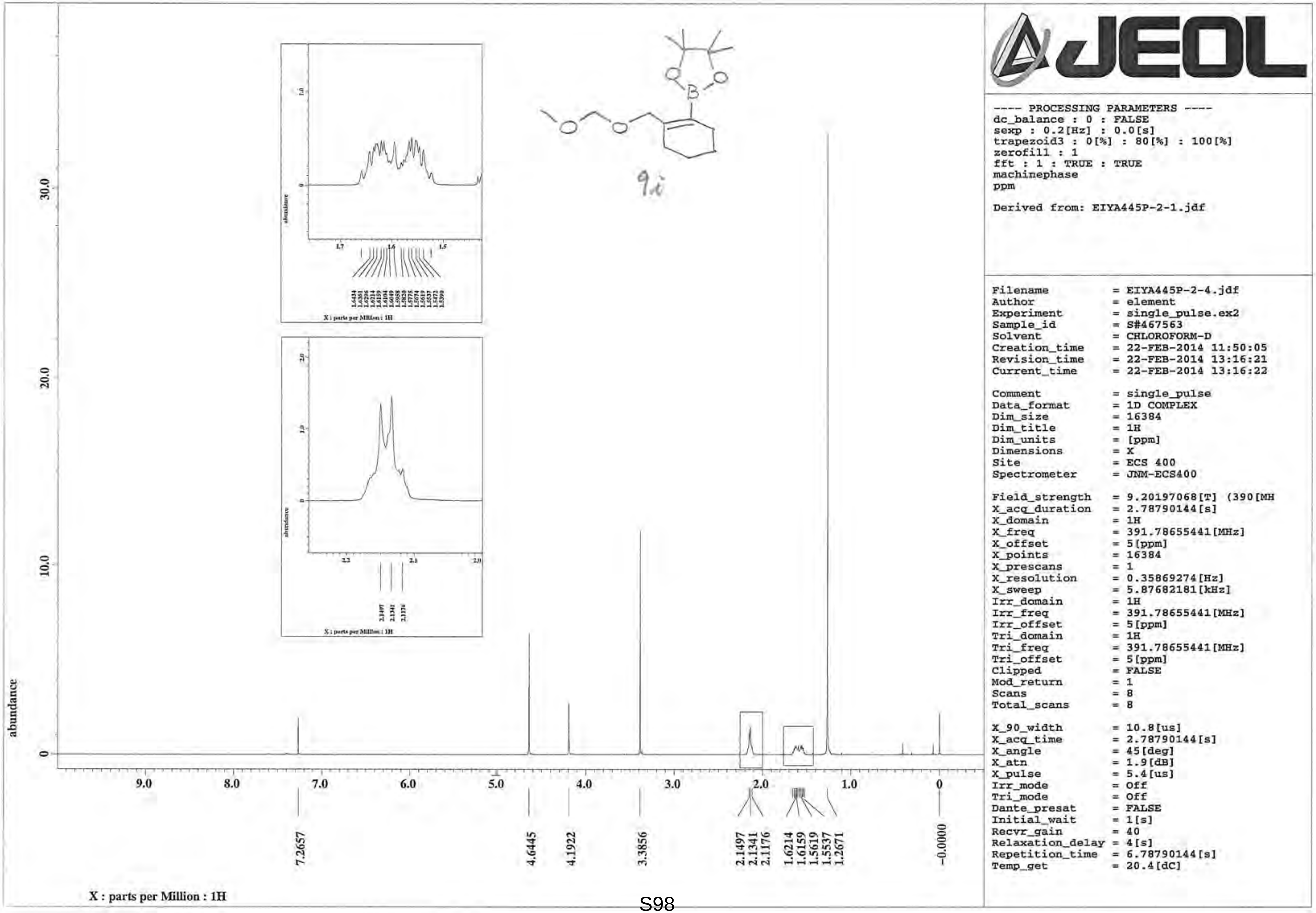
JEOL
---- PROCESSING PARAMETERS ----
dc_balance : 0 : FALSE
sexp : 0.2[Hz] : 0.0[s]
trapezoid3 : 0[%] : 80[%] : 100[%]
zerofill : 1
fft : 1 : TRUE : TRUE
machinephase
ppm
Derived from: EIYA445P-2-1.jdf
Filename = EIYA445P-2-4.jdf
Author = element
Experiment = single_pulse.ex2
Sample_id = S#467563
Solvent = CHLOROFORM-D
Creation_time = 22-FEB-2014 11:50:05
Revision_time = 22-FEB-2014 13:16:21
Current_time = 22-FEB-2014 13:16:22
Comment = single_pulse
Data_format = 1D COMPLEX
Dim_size = 16384
Dim_title = 1H
Dim_units = [ppm]
Dimensions = X
Site = ECS 400
Spectrometer = JNM-ECS400
Field_strength = 9.20197068[T] (390[MH
X_acq_duration = 2.78790144[s]
X_domain = 1H
X_freq = 391.78655441[MHz]
X_offset = 5[ppm]
X_points = 16384
X_prescans = 1
X_resolution = 0.35869274[Hz]
X_sweep = 5.87682181[kHz]
Irr_domain = 1H
Irr_freq = 391.78655441[MHz]
Irr_offset = 5[ppm]
Tri_domain = 1H
Tri_freq = 391.78655441[MHz]
Tri_offset = 5[ppm]
Clipped = FALSE
Mod_return = 1
Scans = 8
Total_scans = 8
X_90_width = 10.8[us]
X_acq_time = 2.78790144[s]
X_angle = 45[deg]
X_atn = 1.9[dB]
X_pulse = 5.4[us]
Irr_mode = Off
Tri_mode = Off
Dante_presat = FALSE
Initial_wait = 1[s]
Recvr_gain = 40
Relaxation_delay = 4[s]
Repetition_time = 6.78790144[s]
Temp_get = 20.4[dC]
9i
abundance
X : parts per Million : 1H
7.2657
4.6445
4.1922
3.3856
2.1497
2.1341
2.1176
1.6214
1.6159
1.5619
1.5537
1.2671
-0.0000

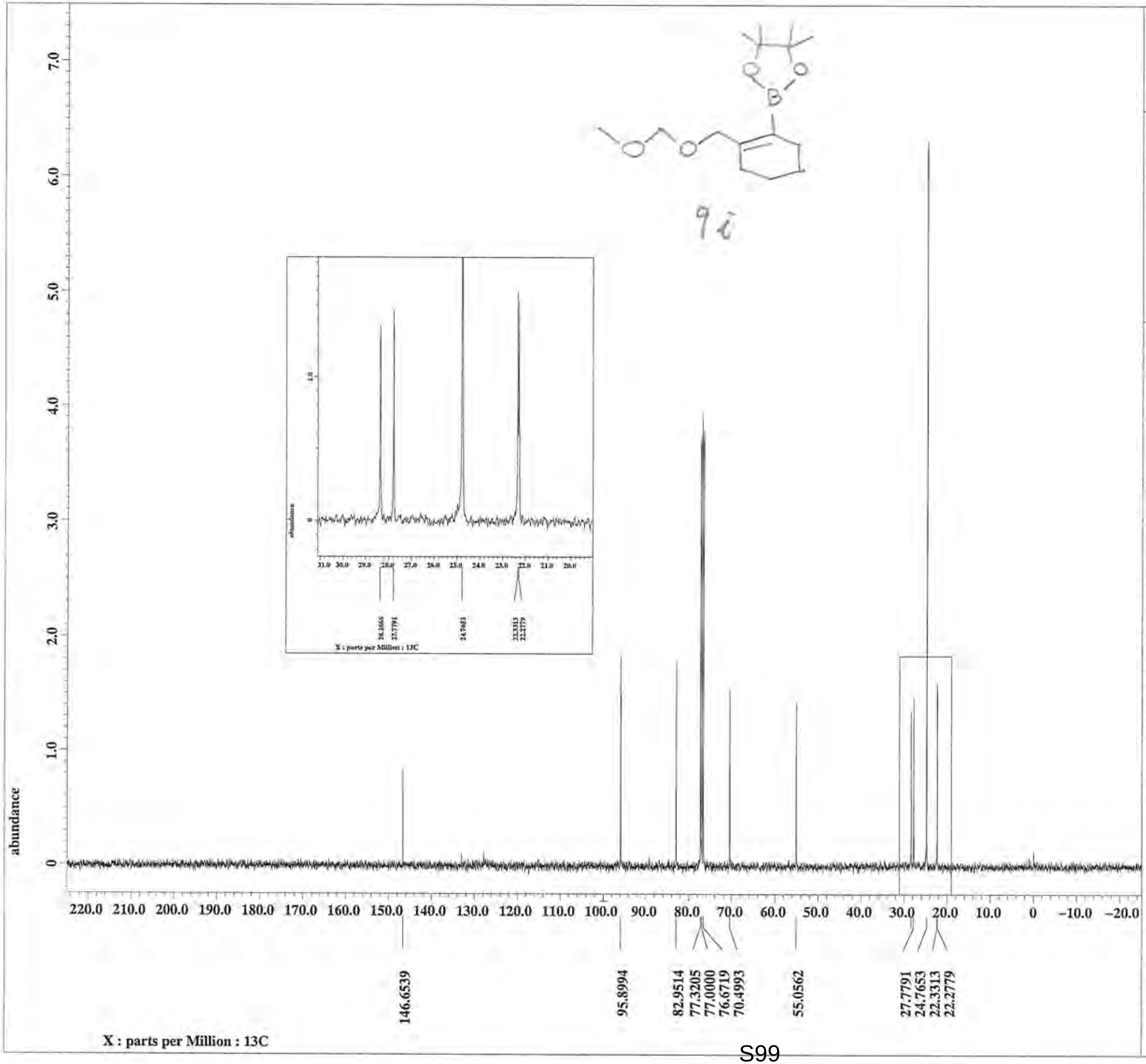

JEOL

---- PROCESSING PARAMETERS ----
dc_balance : 0 : FALSE
sexp : 2.0[Hz] : 0.0[s]
trapezoid3 : 0[%] : 80[%] : 100[%]
zerofill : 1
fft : 1 : TRUE : TRUE
machinephase
ppm

Derived from: EIYA445-2 C_copy-1.jdf

Filename	= EIYA445-2 C_copy-3.jd
Author	= element
Experiment	= single_pulse_dec
Sample_id	= S#472909
Solvent	= CHLOROFORM-D
Creation_time	= 22-FEB-2014 12:05:37
Revision_time	= 22-FEB-2014 13:17:21
Current_time	= 22-FEB-2014 13:18:00
Comment	= single pulse decouple
Data_format	= 1D COMPLEX
Dim_size	= 32768
Dim_title	= 13C
Dim_units	= [ppm]
Dimensions	= X
Site	= ECS 400
Spectrometer	= JNM-ECS400
Field_strength	= 9.20197068[T] (390[MH
X_acq_duration	= 1.3303808[s]
X_domain	= 13C
X_freq	= 98.51479726[MHz]
X_offset	= 100[ppm]
X_points	= 32768
X_prescans	= 4
X_resolution	= 0.75166449[Hz]
X_sweep	= 24.63054187[kHz]
Irr_domain	= 1H
Irr_freq	= 391.78655441[MHz]
Irr_offset	= 5[ppm]
Clipped	= FALSE
Incomplete_copy	= TRUE
Mod_return	= 1
Scans	= 133
Total_scans	= 133
X_90_width	= 8.15[us]
X_acq_time	= 1.3303808[s]
X_angle	= 30[deg]
X_atn	= 4.9[dB]
X_pulse	= 2.71666667[us]
Irr_atn_dec	= 22.445[dB]
Irr_atn_noe	= 22.445[dB]
Irr_noise	= WALTZ
Decoupling	= TRUE
Initial_wait	= 1[s]
Noe	= TRUE
Noe_time	= 2[s]
Recvr_gain	= 72
Relaxation_delay	= 2[s]
Repetition_time	= 3.3303808[s]
Temp_get	= 20.1[dC]

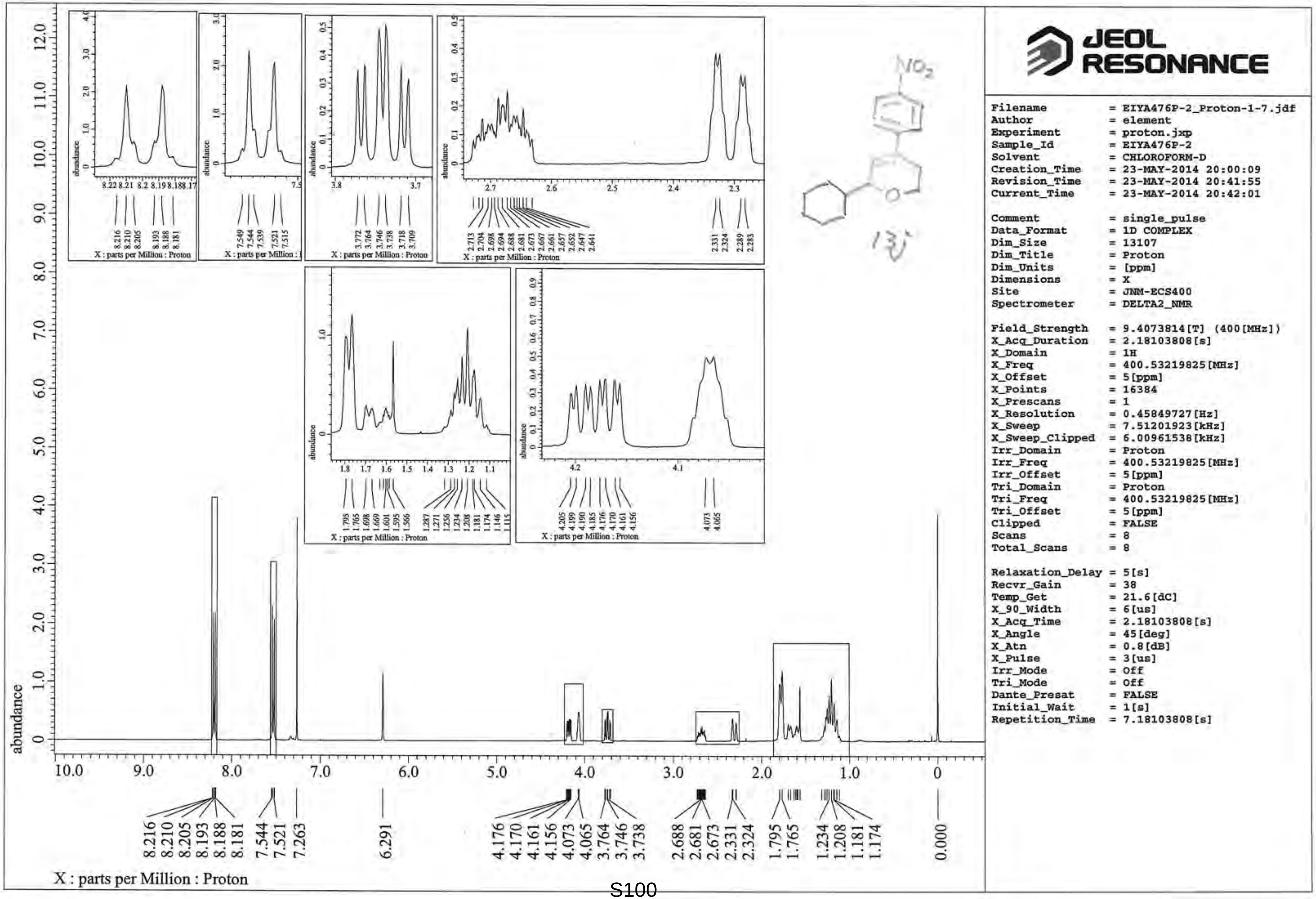
JEOL RESONANCE
Filename = EIYA476P-2_Proton-1-7.jdf
Author = element
Experiment = proton.jxp
Sample_Id = EIYA476P-2
Solvent = CHLOROFORM-D
Creation_Time = 23-MAY-2014 20:00:09
Revision_Time = 23-MAY-2014 20:41:55
Current_Time = 23-MAY-2014 20:42:01
Comment = single_pulse
Data_Format = 1D COMPLEX
Dim_Size = 13107
Dim_Title = Proton
Dim_Units = [ppm]
Dimensions = X
Site = JNM-ECS400
Spectrometer = DELTA2_NMR
Field_Strength = 9.4073814[T] (400[MHz])
X_Acq_Duration = 2.18103808[s]
X_Domain = 1H
X_Freq = 400.53219825[MHz]
X_Offset = 5[ppm]
X_Points = 16384
X_Prescans = 1
X_Resolution = 0.45849727[Hz]
X_Sweep = 7.51201923[kHz]
X_Sweep_Clipped = 6.00961538[kHz]
Irr_Domain = Proton
Irr_Freq = 400.53219825[MHz]
Irr_Offset = 5[ppm]
Tri_Domain = Proton
Tri_Freq = 400.53219825[MHz]
Tri_Offset = 5[ppm]
Clipped = FALSE
Scans = 8
Total_Scans = 8
Relaxation_Delay = 5[s]
Recvr_Gain = 38
Temp_Get = 21.6[dC]
X_90_Width = 6[us]
X_Acq_Time = 2.18103808[s]
X_Angle = 45[deg]
X_Atn = 0.8[dB]
X_Pulse = 3[us]
Irr_Mode = Off
Tri_Mode = Off
Dante_Presat = FALSE
Initial_Wait = 1[s]
Repetition_Time = 7.18103808[s]
NO2
O
13j
abundance
X : parts per Million : Proton
8.216 8.210 8.205 8.193 8.188 8.181 7.544 7.521 7.263 6.291 4.176 4.170 4.161 4.156 4.073 4.065 3.764 3.746 3.738 2.688 2.681 2.673 2.331 2.324 1.795 1.765 1.234 1.208 1.181 1.174 0.000

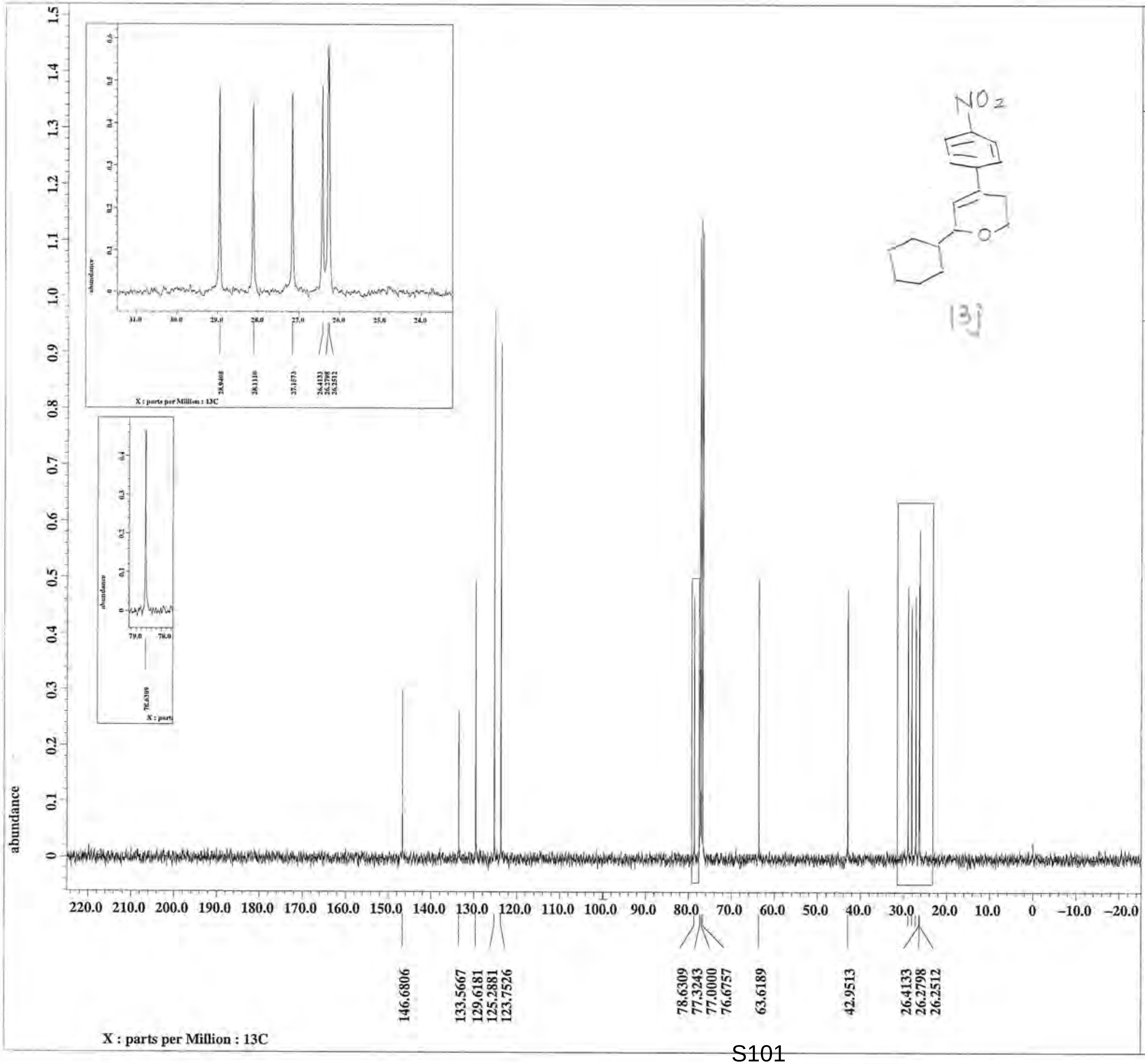

13

JEOL

```
---- PROCESSING PARAMETERS ----
dc_balance : 0 : FALSE
sexp : 2.0[Hz] : 0.0[s]
trapezoid3 : 0[%] : 80[%] : 100[%]
zerofill : 1
fft : 1 : TRUE : TRUE
machinephase
ppm

Derived from: EIYA476C-2-1.jdf

Filename          = EIYA476C-2-4.jdf
Author            = element
Experiment        = single_pulse_dec
Sample_id         = S#781331
Solvent           = CHLOROFORM-D
Creation_time     = 23-MAY-2014 20:27:15
Revision_time     = 23-MAY-2014 21:53:00
Current_time      = 23-MAY-2014 21:56:40

Comment           = single pulse decouple
Data_format       = 1D COMPLEX
Dim_size          = 26214
Dim_title         = 13C
Dim_units         = [ppm]
Dimensions        = X
Site              = ECS 400
Spectrometer      = JNM-ECS400

Field_strength    = 9.20197068[T] (390[MH
X_acq_duration    = 1.06430464[s]
X_domain          = 13C
X_freq            = 98.51479726[MHz]
X_offset          = 100[ppm]
X_points          = 32768
X_prescans        = 4
X_resolution      = 0.93958061[Hz]
X_sweep           = 30.78817734[kHz]
Irr_domain        = 1H
Irr_freq          = 391.78655441[MHz]
Irr_offset        = 5[ppm]
Clipped           = FALSE
Mod_return        = 1
Scans             = 150
Total_scans       = 150

X_90_width        = 8.15[us]
X_acq_time        = 1.06430464[s]
X_angle           = 30[deg]
X_atn             = 4.9[dB]
X_pulse           = 2.71666667[us]
Irr_atn_dec       = 22.445[dB]
Irr_atn_noe       = 22.445[dB]
Irr_noise         = WALTZ
Decoupling        = TRUE
Initial_wait      = 1[s]
Noe               = TRUE
Noe_time          = 2[s]
Recvr_gain        = 60
Relaxation_delay  = 2[s]
Repetition_time   = 3.06430464[s]
Temp_get          = 20.1[dC]
```

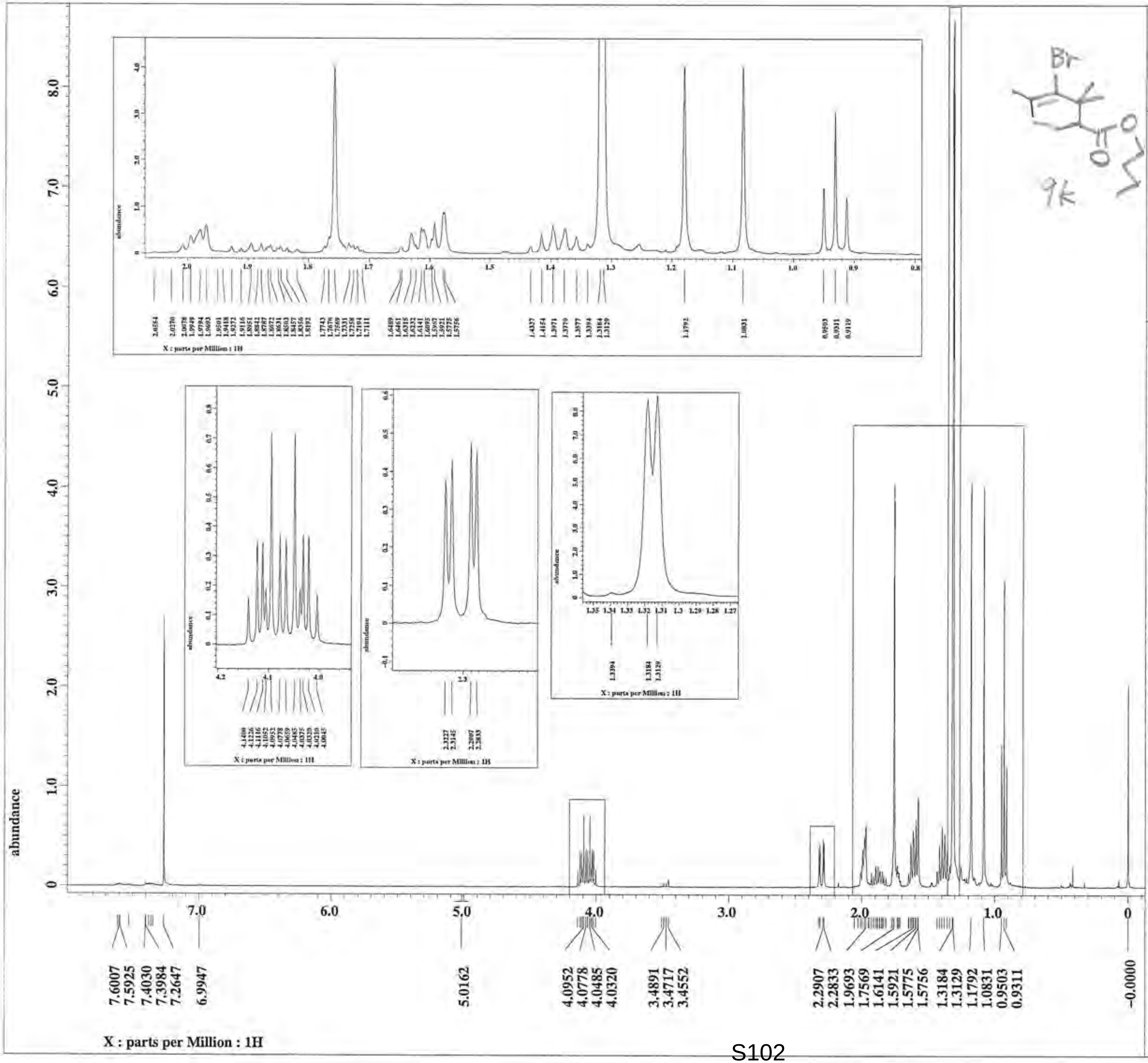

JEOL

---- PROCESSING PARAMETERS ----
dc_balance : 0 : FALSE
sexp : 0.2[Hz] : 0.0[s]
trapezoid3 : 0[%] : 80[%] : 100[%]
zerofill : 1
fft : 1 : TRUE : TRUE
machinephase
ppm

Derived from: EIYA465-2-1.jdf

Filename	= EIYA465-2-4.jdf
Author	= element
Experiment	= single_pulse.ex2
Sample_id	= S#788275
Solvent	= CHLOROFORM-D
Creation_time	= 8-APR-2014 20:38:38
Revision_time	= 8-APR-2014 22:10:59
Current_time	= 8-APR-2014 22:11:06
Comment	= single_pulse
Data_format	= 1D COMPLEX
Dim_size	= 16384
Dim_title	= 1H
Dim_units	= [ppm]
Dimensions	= X
Site	= ECS 400
Spectrometer	= JNM-ECS400
Field_strength	= 9.20197068[T] (390[MH
X_acq_duration	= 2.78790144[s]
X_domain	= 1H
X_freq	= 391.78655441[MHz]
X_offset	= 5[ppm]
X_points	= 16384
X_prescans	= 1
X_resolution	= 0.35869274[Hz]
X_sweep	= 5.87682181[kHz]
Irr_domain	= 1H
Irr_freq	= 391.78655441[MHz]
Irr_offset	= 5[ppm]
Tri_domain	= 1H
Tri_freq	= 391.78655441[MHz]
Tri_offset	= 5[ppm]
Clipped	= FALSE
Mod_return	= 1
Scans	= 8
Total_scans	= 8
X_90_width	= 10.8[us]
X_acq_time	= 2.78790144[s]
X_angle	= 45[deg]
X_atn	= 1.9[dB]
X_pulse	= 5.4[us]
Irr_mode	= Off
Tri_mode	= Off
Dante_presat	= FALSE
Initial_wait	= 1[s]
Recvr_gain	= 44
Relaxation_delay	= 4[s]
Repetition_time	= 6.78790144[s]
Temp_get	= 20.2[dC]

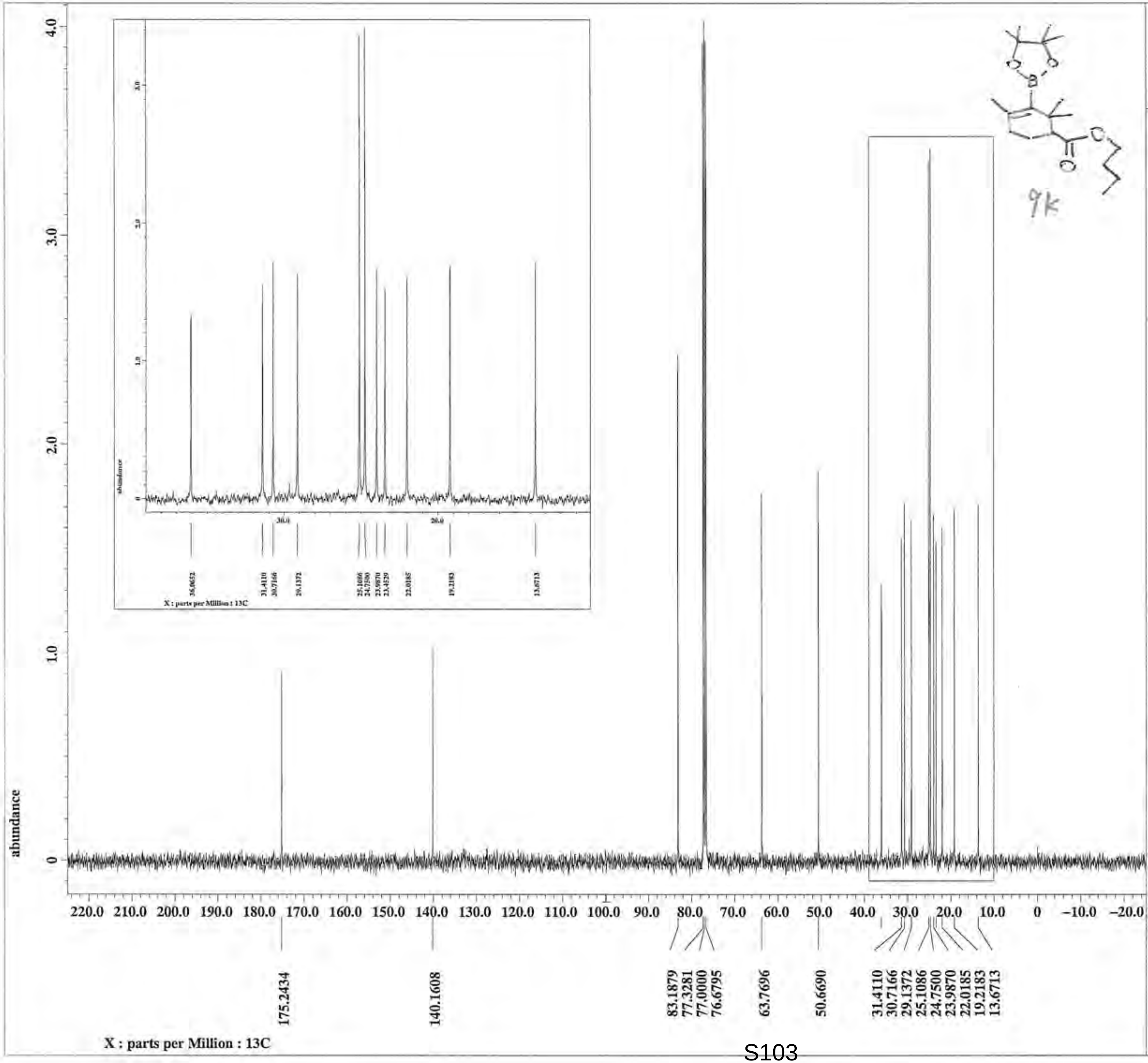


```
---- PROCESSING PARAMETERS ----
dc_balance : 0 : FALSE
sexp : 2.0[Hz] : 0.0[s]
trapezoid3 : 0[%] : 80[%] : 100[%]
zerofill : 1
fft : 1 : TRUE : TRUE
machinephase
ppm

Derived from: EIYA465-2C-1.jdf
```

```
Filename            = EIYA465-2C-3.jdf
Author              = element
Experiment          = single_pulse_dec
Sample_id           = S#793383
Solvent             = CHLOROFORM-D
Creation_time       =  8-APR-2014 20:54:53
Revision_time       =  8-APR-2014 22:13:09
Current_time        =  8-APR-2014 22:13:54

Comment             = single pulse decouple
Data_format         = 1D COMPLEX
Dim_size            = 32768
Dim_title           = 13C
Dim_units           = [ppm]
Dimensions          = X
Site                = ECS 400
Spectrometer        = JNM-ECS400

Field_strength      = 9.20197068[T] (390[MH
X_acq_duration      = 1.3303808[s]
X_domain            = 13C
X_freq              = 98.51479726[MHz]
X_offset            = 100[ppm]
X_points            = 32768
X_prescans          = 4
X_resolution        = 0.75166449[Hz]
X_sweep             = 24.63054187[kHz]
Irr_domain          = 1H
Irr_freq            = 391.78655441[MHz]
Irr_offset          = 5[ppm]
Clipped             = FALSE
Mod_return          = 1
Scans               = 149
Total_scans         = 149

X_90_width          = 8.15[us]
X_acq_time          = 1.3303808[s]
X_angle             = 30[deg]
X_atn               = 4.9[dB]
X_pulse             = 2.71666667[us]
Irr_atn_dec         = 22.445[dB]
Irr_atn_noe         = 22.445[dB]
Irr_noise           = WALTZ
Decoupling          = TRUE
Initial_wait        = 1[s]
Noe                 = TRUE
Noe_time            = 2[s]
Recvr_gain          = 72
Relaxation_delay    = 2[s]
Repetition_time     = 3.3303808[s]
Temp_get            = 20.8[dC]
```
